# Machine learning *vs.* field 3D-QSAR models for serotonin 2A receptor psychoactive substances identification†

**DOI:** 10.1039/d1ra01335a

**Published:** 2021-04-20

**Authors:** Giuseppe Floresta, Vincenzo Abbate

**Affiliations:** Department of Analytical, Environmental and Forensic Sciences, King's College London London UK giuseppe.floresta@kcl.ac.uk vincenzo.abbate@kcl.ac.uk

## Abstract

Serotonergic psychedelics, substances exerting their effects primarily through the serotonin 2A receptor (5HT2AR), continue to comprise a substantial portion of reported new psychoactive substances (NPS). In this paper five quantitative structure–activity relationship (QSAR) models for predicting the affinity of 5-HT2AR ligands have been developed. The resulting models, exploiting the accessibility of the QSAR equations, generate a useful tool for the investigation and identification of unclassified molecules. The models have been built using a set of 375 molecules using Forge software, and the quality was confirmed by statistical analysis, resulting in effective tools with respect to their predictive and descriptive capabilities. The best performing algorithm among the machine learning approaches and the classical field 3D-QSAR model were then combined to produce a consensus model and were exploited, together with a pharmacophorefilter, to explore the 5-HT2AR activity of 523 105 natural products, to classify a set of recently reported 5-HT2AR NPS and to design new potential active molecules. The findings of this study should facilitate the identification and classification of emerging 5-HT2AR ligands including NPS.

## Abstract 2

Five QSAR models for predicting the affinity of 5-HT2AR ligands have been developed. The resulting models generate a useful tool for the investigation and identification of unclassified new psychoactive substances (NPS).

## Graphical abstract

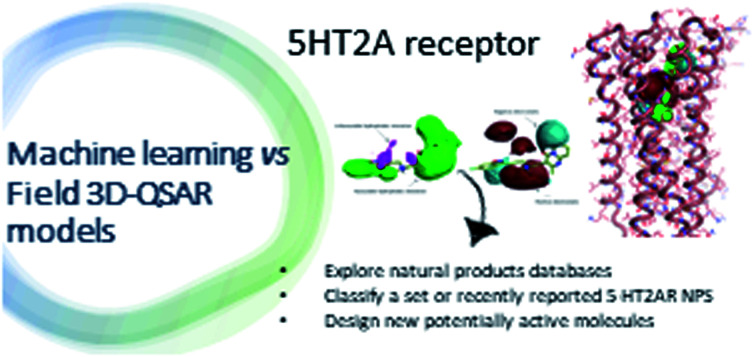

## Introduction

The 5-HT2A receptor (5HT2AR) is a surface G protein-coupled receptor (GPCR) subtype of the 5-HT2 serotonin receptor family. The receptor was first discovered as a target of serotonergic psychedelic drugs such as LSD and psilocybin, and later it was proved to be a mediator of the action of many antipsychotic drugs.^1^ Due to the wide expression of the receptor in the central nervous system (CNS) and other tissues, several physiological processes are mediated by the receptor: neuronal excitation, hallucinations, out-of-body experiences, and fear; its activation in the hypothalamus causes increases in several hormonal levels; activation of the receptor produces potent anti-inflammatory effects in several tissues including cardiovascular; it also has a role in memory and learning, in arthralgia, Alzheimer's disease, sleep paralysis, *etc.*^2–6^

Agonists and antagonists of this receptor are today used or being studied for several clinical application, *e.g.* methysergide (partial agonists) is used in treatment of migraine, AL-34662 (peripherally selective agonists) reduces the pressure inside the eyes without crossing the blood–brain barrier and producing hallucinogenic side effects;^7^ atypical antipsychotic drugs such as risperidone, clozapine, quetiapine and asenapine are relatively potent antagonists of 5-HT2A, and nelotanserin (inverse agonist) is studied for the treatment of insomnia.^8^ 5HT2AR is also targeted for neuroimaging of patients with major depressive disorder using PET imaging.^9^ However, due to the natural expression of this receptor in the CNS, the receptor is also targeted by new psychoactive substances (NPS) designed for recreational use, and every year hundreds of NPS are unearthed on the black market.

**Fig. 1:**
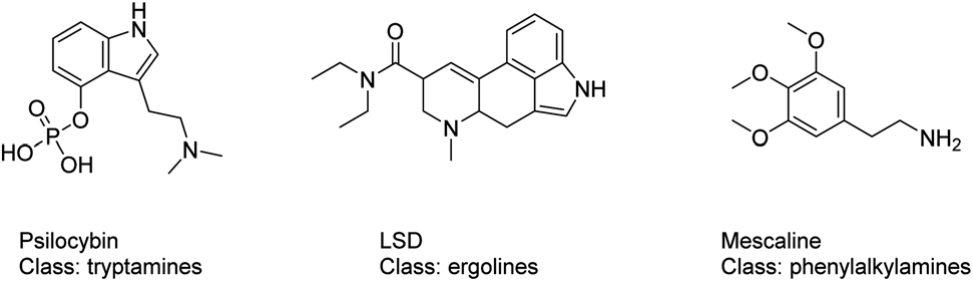
Structures of 5HT2AR psychoactive substances: the tryptamines (*e.g.* psilocybin), the ergolines (*e.g.* lysergic acid diethylamide (LSD)) and the phenylalkylamines (*e.g.* mescaline).

By early 2020, more than 950 NPS had been reported to the United Nations Office on Drugs and Crime (UNODC); in parallel, at the end of 2019 the European Monitoring Centre for Drugs and Drug Addiction (EMCDDA) was monitoring around 790 NPS, 53 of which were reported for the first time in Europe in 2019.^10,11^ Over the time, the group of serotonergic psychedelics constituted a significant proportion of these recently reported NPS.^12^ The term serotonergic psychedelics includes molecules that exert their pharmacological activity mainly by means of interacting with the serotonin 5-HT2A receptor (5-HT2AR).^13^ The class of serotonergic psychedelics is formed by structurally diverse subclasses of compounds: the tryptamines (*e.g.* psilocybin), the ergolines (*e.g.* lysergic acid diethylamide (LSD)) and the phenylalkylamines (*e.g.* mescaline), Fig. 1.^14^ Mystical experiences, empathic feelings, alterations in consciousness, sensory and somatic effects are the main effects looked among users of 5HT2AR NPS ligands. However, severe adverse effects, such as agitation, tachycardia, hyperthermia, rhabdomyolysis, hypertension and seizures, can also frequently occurr.^15,16^ With the more recent group of NBOMes, an emerging subclass of the phenylalkylamines psychedelics, cases requiring hospitalization, suicide attempts, deaths and mass poisoning (with *e.g.* BromoDragonFLY and 2C-E) have been reported.^17–19^

QSAR models are utilized to assist predicting or understanding molecular/drug design within the chemical and natural sciences.^20,21^ Few attempts have been performed to build up QSAR models for the 5HT2AR, however all of them have been produced through a constrained number of compounds with comparable chemical structures and as a result, only the affinities of a restricted class of compounds could be determined.^22–24^

To facilitate the investigation of chemical datasets for 5HT2AR ligands capabilities and to potentially identify emerging or future NPS,^25,26^ here we report the development of five quantitative structure–activity relationship (QSAR) models for predicting the affinity of 5-HT2AR ligands. Details of all the compounds having experimentally determined *K*_i_ values were retrieved from the literature and downloaded from CHEMBL. All models were built using a set of 375 5-HT2AR ligands. Four out of five such models were built by machine learning algorithms (*i.e.* support vector machine (SVM), *k*-nearest neighbors (*k*NN), random forest (RF) and relevance vector machine (RVM)) and one model was built with a field-based methodology.^27,28^ Differently to previously published 5HT2AR QSAR models, the ones reported here include a wide range of chemically different (sub)classes of compounds. Moreover, the best performing tool from the generated machine learning (SVM) and the field based 3D-QSAR models were employed to rank a dataset of recently reported 5HT2AR NPS ligands and to screen a large dataset of natural products to identify potential active molecules against 5-HT2AR.

All the reported QSAR models were developed using the software Forge. Conversely to classical 3D-QSAR modelling, where molecular descriptors are calculated at the interception points of a 3D grid, which surrounds the entire space of the aligned molecules,^29–31^ the modelling calculation in Forge is characterized by the use of probe positions that are defined directly from the field points of the aligned molecules in the training set, and only these positions are then used to describe the volume and the electrostatic potential of each molecule.^32–35^

## Methods

### Biological data & 3D structures generation

The chemical structures of the 375 5-HT2AR ligands were selected from the literature where the experimental *K*_i_ values are reported and retrieved from ChEMBL Database (https://www.ebi.ac.uk/chembl/g/). The biological data were all derived from similar and comparable cellular based experiments. Ki derived from displacement of [3*H*]-ketanserin, [125I]-DOI or [3*H*]INBMeO from human 5HT2AR expressed CHO or HEK293 cells were used. The binding affinity data of the selected dataset were converted into their negative decimal logarithm p*K*_i_ (p*K*_i_ = −log *K*_i_). Collected p*K*_i_ values fall into a range 5.19–10.40. The structures of the studied molecules were built using Marvin 17.21.0, ChemAxon (https://www.chemaxon.com).^36^ The 2D structures were subjected to molecular mechanics energy minimization by Merck molecular force field (MMFF94) using the Marvin Sketch geometrical descriptors plugin.^37^ The protonation states of the molecules were calculated assuming a pH = 7.0. The geometry of the obtained 3D structures was further optimized at semi-empirical level using the parameterized model number 3 (PM3) Hamiltonian as implemented in MOPAC package (vMOPAC2016).^38–40^

### Compound alignment

All the 3D generated molecules, with their respective p*K*_i_ values, were imported into the computational chemistry software Forge (v10.4.2) for setting the machine learnings (SVM, *k*NN; RF and RVM) and the field-based 3D-QSAR model. Out of the 375 ligands for the 5HT2AR, we randomly selected (selection based on activity stratification) 300 molecules (80%) as a training set to build the models, while the remaining 75 compounds (20%) served as test set to evaluate the models.^41^ For both training and test sets, the selected molecules covered a wide range of biological activities: from 10.40 to 5.19 p*K*_i_ for the training set and from 10.00 to 5.34 p*K*_i_ for the test set. All the molecules were aligned to the three co-crystallized ligands of 5HT2AR in their bioactive conformation inside the binding site retrieved from the protein data bank (https://www.rcsb.org/) PDB ID: 6a93, 6a94 and 6wgt.^42,43^ 8NU (risperidone) was the ligand selected for 6a93, ZOT (zotepine) was the ligand selected for 6a94, 7LD ((8alpha)-*N*,*N*-diethyl-6-methyl-9,10-didehydroergoline-8-carboxamide) was the ligand selected for 6wgt.

The field points (used as a descriptor of negative and positive electrostatic, van der Waals shape, and hydrophobic areas)^28^ of each molecule were generated using the extended electron distribution (XED) force field. The molecules in both training set and test set were aligned to the reference compounds by a most common substructure calculation (considering the calculated field points) employing a customized set-up.^44^ The maximum number of conformations produced for each compound was set to 500. The root-mean-square deviation of nuclear positions (RMSD) cutoff for copy conformers was set to 0.5 Å. This parameter controls the likeness which two conformers are accepted indistinguishable. The gradient cutoff for conformer minimization was set to 0.1 kcal mol^−1^. The energy window was set to 2.5 kcal mol^−1^. Conformations that gave a minimized energy outside the energy window were discarded. All the alignments were manually checked to ensure the best possible alignment. All the field points of the molecules used for training the models were exploited to get a gauge invariant set of sampling points, which reduced the number of descriptors that must be considered. The sample values were calculated using a distance of 1 Å between the sample points, ensuring that all areas around the compounds that could contribute to the activity were effectively described. All the software's parameters used for the conformation hunt, alignment, and build model calculations are reported in the ESI.†

## Results and discussion

### Statistical analysis and results

**Fig. 2:**
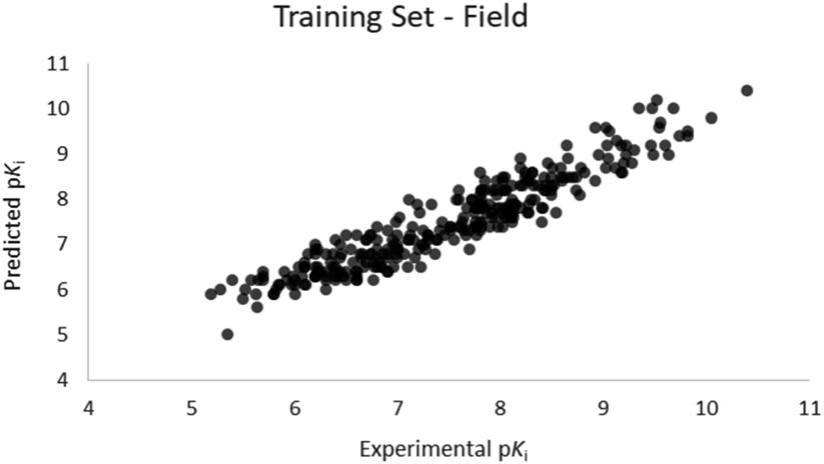
Five component field based QSAR model experimental *vs.* predicted p*K*_i_ of the compounds in the training set.

**Fig. 3:**
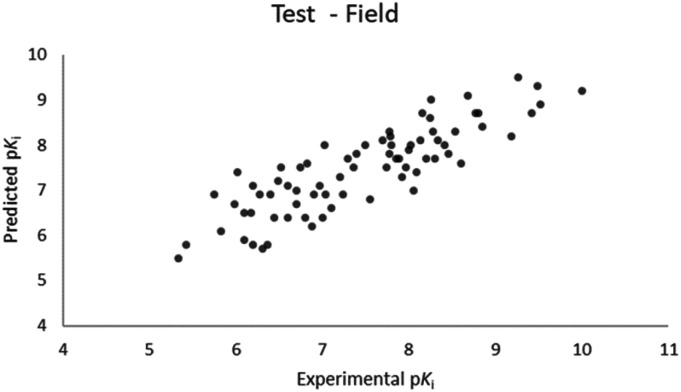
Five component field based QSAR model experimental *vs.* predicted p*K*_i_ of the compounds in the test set.

**Fig. 4:**
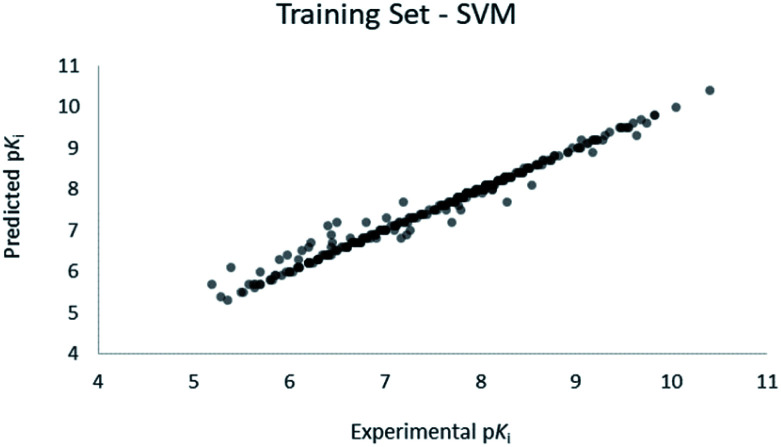
SVM QSAR model experimental *vs.* predicted p*K*_i_ of the compounds in the training set.

**Fig. 5:**
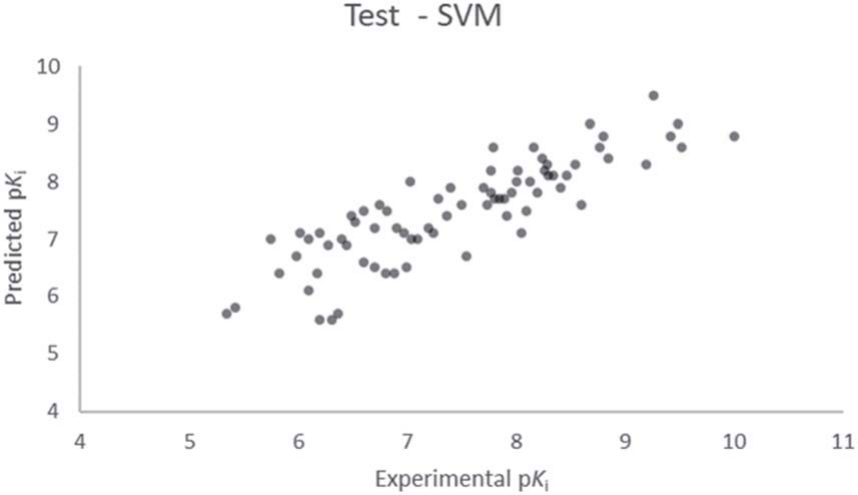
SVM QSAR model experimental *vs.* predicted p*K*_i_ of the compounds in the test set.

For the calculation of the models, a partial least squares (PLS) regression method specifically employing the SIMPLS algorithm was used for the field model, whereas *k*-Nearest Neighbors, Support Vector Machine, Relevance Vector Machine and Random Forest were used in the supervised machine learning models.^45,46^ Detailed information for the assembly and the validation of all the models are reported in the ESI.† The 3D-QSAR models' statistics are reported in Table 1. The 5-component field based model shows both good predictive and descriptive capabilities, demonstrated by the good *r*^2^ (0.88) and *q*^2^ (0.75) values for the training and the cross-validated training set (Fig. 2).^47^ The plots of experimental *vs.* predicted affinities for the compounds in the test set (Fig. 3) show accurate predictions with only a few outliers and an excellent cross-validated *r*^2^ (0.73). Regarding the supervised learning models (SVM, *k*NN, RF and RVM), the *r*^2^ of the models ranked between 0.99 and 0.66, the *q*^2^ between 0.74 and 0.66 and the *r*^2^ test between 0.73 and 0.79 (Table 1). According to these results, the best performing algorithm among the supervised machine learning models is the SVM. The model experimental *vs.* predicted p*K*_i_ of the compounds in the training set and in the test set for the SVM model are reported in Fig. 4 and 5.

**Table 1:** Models statistics

Model	*r* ^2^ training set	*q* ^2^ training set	*r* ^2^ test set	MSE^a^ training set	MSE^a^ test set	MAE^b^ training set	MAE^b^ test set	MAPE^c^ training set	MAPE^c^ test set
3D-field	0.88	0.75	0.73	0.13	0.29	0.31	0.45	4.21	6.24
SVM	0.99	0.74	0.73	0.01	0.30	0.06	0.44	0.84	6.21
*k*NN	0.66	0.66	0.49	0.38	0.56	0.48	0.56	6.75	7.70
RF	0.89	0.64	0.63	0.11	0.41	0.22	0.52	2.96	7.20
RVM	0.89	0.73	0.69	0.12	0.34	0.29	0.40	3.93	5.44

a Mean squared forecast error.

b Mean absolute forecast error.

c Mean absolute percentage forecast error.

**Fig. 6:**
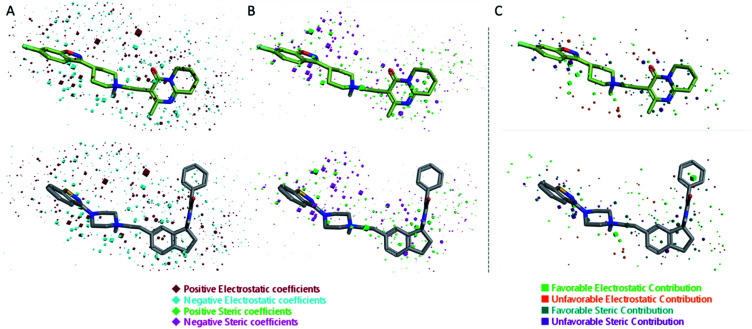
Electrostatic and steric coefficients for the field model. (A) Electrostatic coefficients superimposed to risperidone (used for the alignment, PD BID: 6a93) (up) and the most potent compounds in the training set (down). (B) Steric coefficients superimposed to risperidone (up) and the most potent compounds in the training set (down). (C) Contributions to predicted affinity for risperidone (up) and the most potent compounds in the training set (down).

The 3D visualizations of the field QSAR model is shown in Fig. 6, where the 3D-QSAR coefficients for the two models are superimposed to the most potent molecule in the training set and to risperidone, that was used in the alignment process (PDBID: 6a93). The 3D-QSAR model is portrayed by both steric and electrostatic impacts. The model outlines zones where the equation proposes that the nearby areas have a strong effect on ligand-receptor affinity (Fig. 6). The bigger the points (portrayed as octahedrons), the stronger is the relationship between the electrostatic and steric areas in that position. The higher affinity related to the electrostatic potential is delineated in red for the positive values and in blue for the negative ones. For the steric bulk, the green region leads to higher receptor affinity while the violet zone leads to lower affinity.

**Fig. 7:**
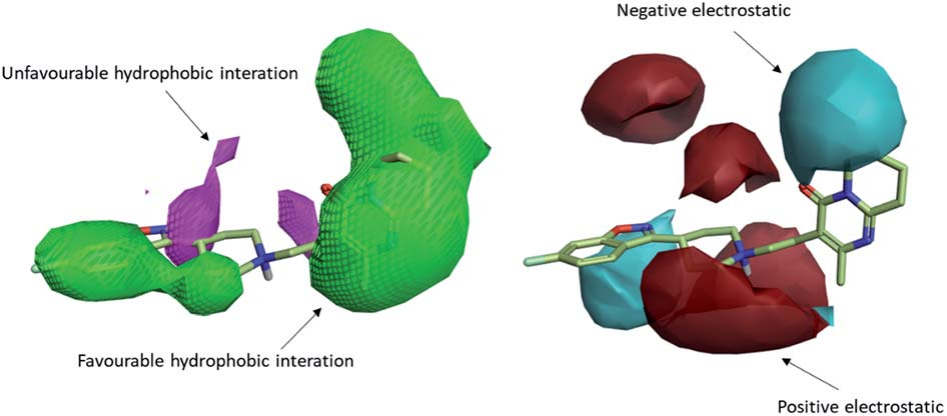
The model map is superimposed to risperidone. Molecular insight of SAR mechanism models, revealing the different lead optimization sites of active compounds. Red color shows positive field region controlling the activity, and blue color the negative ones. Green color shows favorable shape/hydrophobic regions, and purple color the unfavorable ones.

**Fig. 8:**
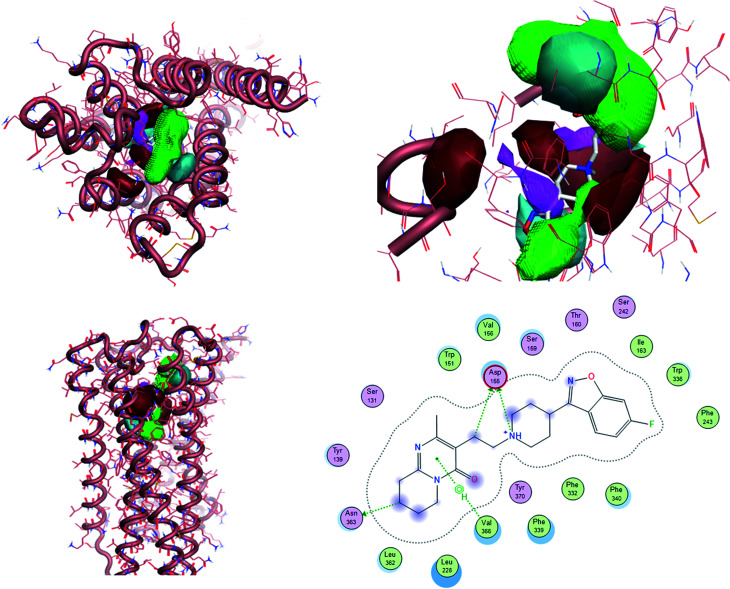
The model AA map is superimposed to risperidone inside 5HT2AR (6a93). 2D interaction between risperidone and 5HT2AR.

To uncover the key highlights of the examined set of compounds against the focused 5HT2AR, a structure–activity relationship (SAR) study was performed through activity-atlas (AA) visualization program. AA is a qualitative strategy valuable for summarizing structure–activity data into 3D maps, which helps within the design and optimization of new compounds. This strategy analyses the SAR of a set of aligned compounds as a function of their electrostatic, hydrophobic, and shape properties through a Bayesian approach to require a global see of the information in a qualitative way. Fig. 7 and 8 show the results of the AA calculations for the 5HT2AR. The model map is superimposed to risperidone in its bioactive conformation retrieved from the crystal structure (6a93). Electrostatic, hydrophobic, and shape features are highlighted by the different colors on the map. In the red area, a more positive electrostatic field increases the receptor-affinity, whereas in the blue region a more negative electrostatic field increases the interaction energies. The violet and the green areas account for the steric and bulk/hydrophobic interactions. In the violet area, a steric/bulk interaction decreases the affinity, whereas in the green area a steric/bulk interaction improves the binding affinity.

Risperidone can be dissected in three different regions: (i) the fluorobenzisoxazol ring; (ii) the pyperidine nucleus; (iii) the tetrahydropyridopyrimidinone ring. Two main hydrophobic areas are described by the AA model for the fluorobenzisoxazol ring and the tetrahydropyridopyrimidinone ring, whereas the pyperidine nucleus is mainly located close to a red area, where a more positive electrostatic field increases the receptor-affinity. The occupancy of both hydrophobic area is fundamental for the affinity; indeed, ligands that do not bear any substituent in this area result in low affinity (*e.g.* (*S*)-1-(2,5-dimethoxyphenyl)propan-2-amine, p*K*_i_ = 5.28, training set; 5-hydroxy-8-(4-methyl-1,4-diazepan-1-yl)-2*H*-benzo[*b*][1,4]oxazin-3(4*H*)-one, p*K*_i_ = 5.19, training set). Inside the binding pocket, risperidone adopts an extended conformation, as showed by the AA model. The basic nitrogen of the pyperidine nucleus forms a salt bridge with Asp155, this interaction being well highlighted in the AA model by the red area surrounding the nitrogen of the pyperidine. This salt bridge is strictly conserved in the structures of aminergic receptors and is thought to be stabilized by the conserved hydrogen bond between Asp155 and Tyr370. The fluorobenzisoxazol ring of risperidone is placed in the bottom hydrophobic cleft represented by a green, favourable hydrophobic interaction, by the AA model. This ring interacts with Ser159 by a CH–π interaction and with Ile163, Phe243 and Phe332 by hydrophobic interactions. As already stated, the occupancy of this area is fundamental for ligand affinity; indeed, mutation of these residues will result in a loss in affinity for 5HT2AR ligands.^42^ The tetrahydropyridopyrimidinone ring is located in the other hydrophobic area of the AA model, interacting with Leu228 and Val366 of the protein.

### NPS identification

Together with synthetic cannabinoids, cathinone derivatives, designer GABA-A/B receptor agonists and novel synthetic opioids, recently emerged and unregulated molecules with high 5HT2AR affinity can be considered NPS. These substances – once identified – are normally screened through cellular *in vitro* tests, that are expensive and time consuming. To exploit the predictive capabilities of our models we selected 29 recently reported NPS (phenethylamines, phenylisopropylamines and *N*-benzylphenethylamines)^48^ with measured EC50, by means of β-arrestin 2 recruitment in 5HT2AR and compared the *in vitro* results with the modelled results of the field based and best machine learning (SVM) based models. Despite the *K*_i_ measures the effective interaction of the molecule with the receptor whereas the EC50 measures the effective concentration at 50% of the total biological effect, the two *K*_i_ and EC50 are held together by the Cheng–Prusoff equation.^49^ This means that for a giving set of agonist or antagonist agents for the 5HT2AR, the most potent agonist or antagonist will have the lowest *K*_i_ (highest p*K*_i_). Of note, the models here reported are not able to distinguish between agonist or antagonist action but are able to rank sets of agonists or antagonists according to their receptor affinity.

**Fig. 9:**
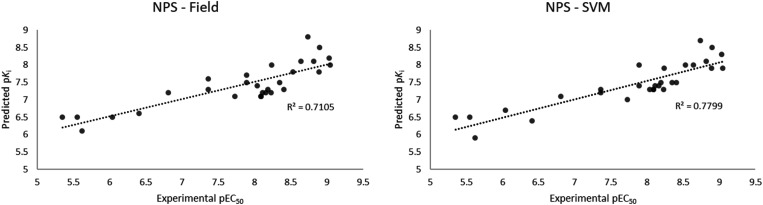
Experimental EC_50_*vs.* calculated p*K*_i_ of the selected 29 NPS with the field (left) and the SVM (right) models.

The results of the experimental EC_50_*vs.* the calculated p*K*_i_ are reported in Fig. 9 and Table S3.† Interestingly, both the field based and the SVM models well performed in the ranking of the 29 compounds, with a slightly better performance for the machine learning model. Both models are able to rank the compounds with a good linearity between the calculated and the experimental values, with *r*^2^ of 0.7105 and 0.7799 for the field and the SVM based models, respectively. More importantly, the models were able to distinctly classify potent compounds from not-potent ones *i.e.* the five most potent compounds (25C-NBOH, 25E-NBOMe, 25D-NBOMe, 25I-NBOMe, 25I-NBOH) in the set were identified among the most potent compounds for 5HT2AR activity by both models. Analogously, the five less potent molecules (25H-NBF, *b-k*-2C-B, 2C-H, DOH, N-Me-2C-H) were also identified as weak binders by both models.

### Finding NPS from natural databases and scaffold hopping analysis

In order to exploit the predicting capability of the models to retrieve novel 5HT2AR binders, the QSAR equations were used to screen for potential active molecules in three databases of natural compounds for a total of 523,105 molecules. Super Natural Product (SNP), ZINC and MolPort Natural (MPN) were selected as a database of natural compounds.^50^ The 523 105 molecules were firstly filtered through a pharmacophore filter using pharmit (http://pharmit.csb.pitt.edu). The chemical database was screened against the pharmacophore models (generated from the three co-cristallyzed ligands of 5HT2AR in their bioactive conformation retrieved from the protein data bank PDB ID: 6a93, 6a94 and 6wgt) to find the best matches in terms of root mean square distance (RMSD) between pharmacophore query features and corresponding ligand points. 910 compounds with the lowest RMSD value (less than 0.75) were selected for further evaluation in our models. The field based and the SVM QSAR models were then used to score the filtered dataset of 910 compounds. With this objective in mind, all of the molecules were aligned to the models as described in the compound alignment paragraph. The six most potent molecules identified by means of the calculated p*K*_i_ from both models are reported in Table 2 and the whole set in Table S4.† Despite molecules 1, 3 and 6 have not been reported as 5HT receptor binders (molecule 1 class was reported for the treatment of cancer^51^ and molecule 6^52^ is derived from pyridyloxy carboxylic acid commonly used as herbicidal whereas no biological activity is reported for molecule 3), molecule 2, 4 and 5 have been tested for their activity against serotoninergic receptors showing the potentiality of the models to find new hits among library of compounds and would deserve further research investigation to better understand the potential 5HT2AR activity of high ranked compounds.^53,54^

**Table 2:** Top 6 ligands from the scoring of natural products

Molecule	Calculated p*K*_i_
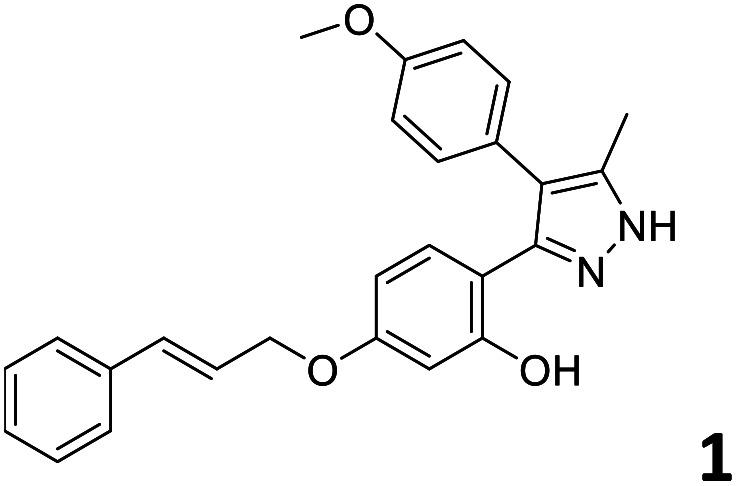	9.15
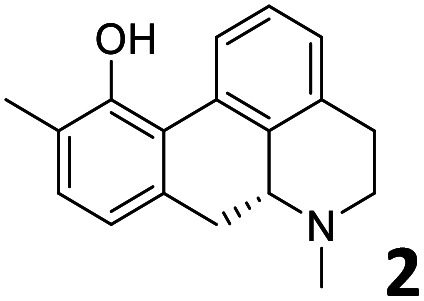	8.95
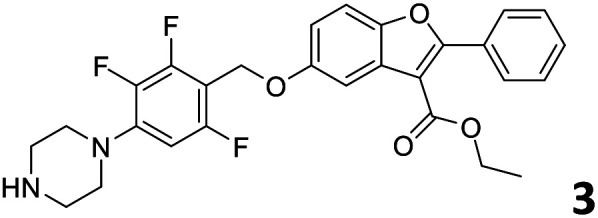	8.95
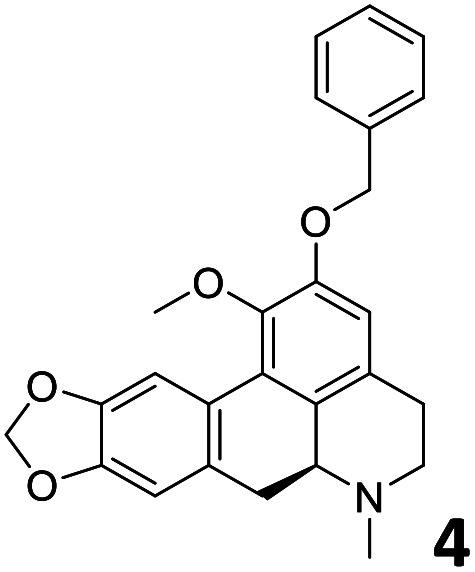	8.90
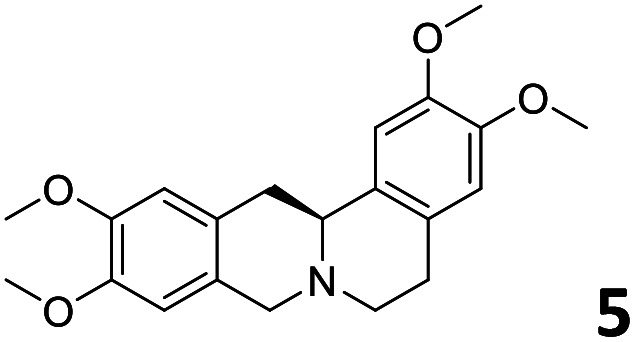	8.90
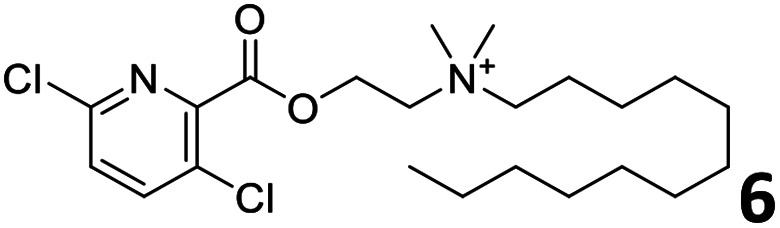	8.75

All the molecules selected as most potent (Table 2) are able to interact with the 5HT2AR correctly inside its binding site. The protonated nitrogen in molecules 2–6 is forming the fundamental salt-bridge with ASP155, moreover the aromatic part of the molecules are accommodated in the two hydrophobic pockets inside the receptor formed by SER131, TYR139, ASN363, LEU362, LEU228, VAL366 or PHE340, PHE243, TRP336, ILE163, SER242, THR160, SER159 gaining stability thought hydrophobic interactions.

**Fig. 10:**
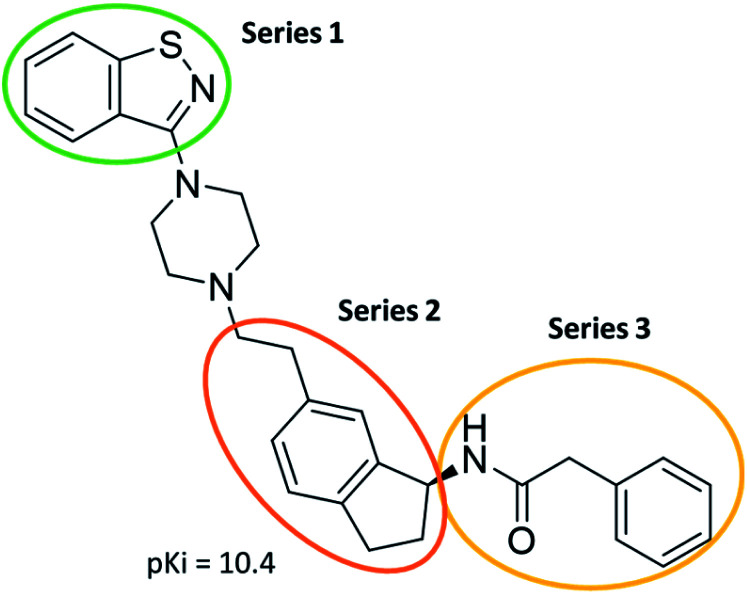
Scaffold hopping series 1–3 derived from the most potent molecule in the training set.

The predictive capabilities of the model were then used for a scaffold-hopping study of the most potent compounds in the training set for the generation of a library of new compounds with high affinity for the receptor. The molecule was divided in three parts and a scaffold hopping analysis was performed as already reported by us (ref. 21, 25 and 26) for each part (Fig. 10). In series 1 the benzo[*d*]isothiazole was substituted, in series 2 the ethyl-2,3-dihydro-1*H*-indene core was substituted and in series 3 the terminal phenylacetamide was substituted. The piperazine nucleus was not replaced due to the important interaction with ASP155. The resulting molecules were then evaluated by the superposition on the 3D-QSAR models. Overall, the results showed in Table 3 (best two molecules for each series) indicate that the scaffold replacement generated new structures with the appropriate chemical features for the binding to the 5HT2AR and the selected compounds resulted more potent than their precursor, showing again the potential of the models to identify new hits among library of compounds and would deserve further research investigation to better understand the potential 5HT2AR activity.

**Table 3:** Top 6 ligands from the scaffold replacement series 1–3. Molecules 7 and 8, series 1. Molecules 9 and 10, series 2. Molecules 11 and 12, series 3

Molecule	Calculated p*K*_i_
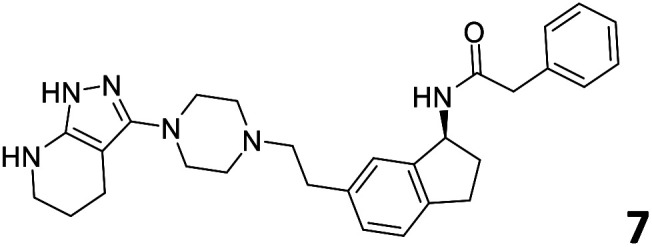	11.2
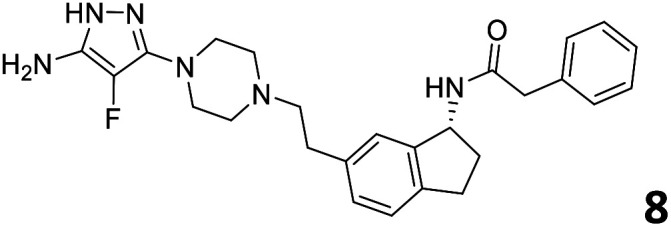	11.1
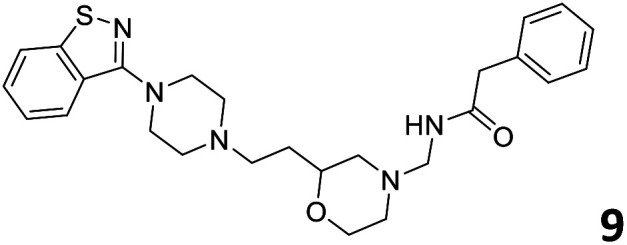	11.3
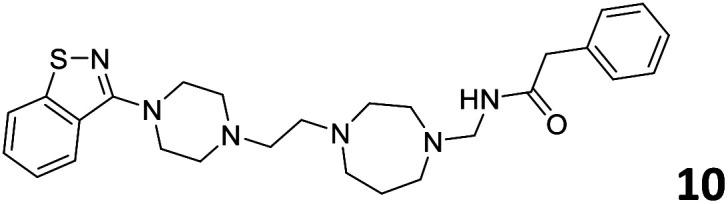	11.3
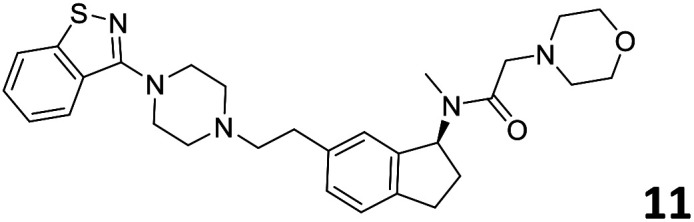	11.1
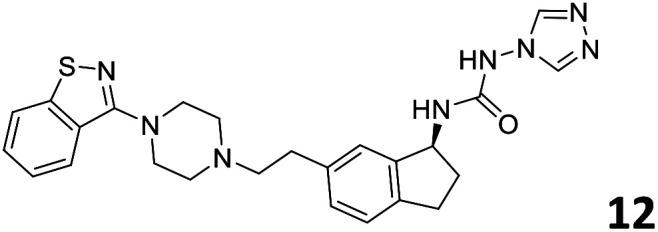	11.0

## Conclusions

The present study deals with the development of different field based and machine learning based 3D-QSAR models for the prediction of the affinity for 5HT2AR ligands and their successive use as a tool for ranking 5HT2AR NPS, the screening of a large dataset of natural molecules and new designed compounds. Forge was used to build a statistically robust 3D-QSAR model using a set of 375 molecules for the 5HT2AR covering a whole range of different chemical classes of the ligands for 5HT2A protein. To the best of our knowledge, this is the first attempt of 3D-QSAR modelling for this subtype of serotoninergic receptor which includes such a wide number and range of molecular structures and accounts for the observed SAR. The 3D-QSAR models showed high statistical quality and robust predictive potential capability, particularly the field based and the SVM which were shown to possess the best statistical quality. Visualization of the model by means of AA allowed processing data in a 3D map format accounting for both steric and electrostatic effects and allow for a rationalization of both potency and selectivity. The field-based model and the SVM-based model were then used to rank a dataset of recently reported 5HT2AR NPS and screen a dataset of natural products for potential active molecules against 5HT2AR. Interestingly, the models were able to rank the experimental EC50 and the calculated *K*_i_ of the NPS in a linear manner and to identify alkaloids already tested for their activity against the 5HT2AR. The 3D-QSAR models here reported will guarantee, prospectively, fruitful applications to speed up the design of novel therapeutic molecules for 5HT2AR as well as the identification/classification of NPS acting on 5HT2AR.^25,26^

## Conflicts of interest

There are no conflicts to declare.

## Supplementary Material

RA-011-D1RA01335A-s001
