# Supplementary material for: Machine learning vs. field 3D-QSAR models for serotonin 2A receptor psychoactive substances identification
Source: RSC Adv. 2021 Apr 20;11(24):14587–95. doi: 10.1039/d1ra01335a (PMC8697832; doi:10.1039/d1ra01335a)
Supplement: RA-011-D1RA01335A-s001 [file RA-011-D1RA01335A-s001.pdf]

Electronic Supplementary Material (ESI) for RSC Advances.
This journal is © The Royal Society of Chemistry 2021

Supporting Information

Machine learning vs Field 3D-QSAR models for serotonin 2A receptor psychoactive substances identification

Giuseppe Floresta [a,*] and Vincenzo Abbate [a,*]

[a] Department of Analytical, Environmental and Forensic Sciences, King's College London, London, UK

Corresponding authors:

E-mail address: giuseppe.floresta@kcl.ac.uk (G. Floresta), vincenzo.abbate@kcl.ac.uk (V. Abbate)

Table of content

Statistical analysis information for the model S3

k-Nearest Neighbors (kNN) S4

Support Vector Machine & Relevance Vector Machine S4

Random Forest S5

Figure S1. Forge's parameters used for the conformation hunt S6

Figure S2. Forge's parameters used for the alignment S6

Figure S3. Forge's parameters used for the build of the field model S7

Figure S4. Forge's parameters used for the build of the kNN model S7

Figure S5. Forge's parameters used for the build of the RF model S7

Figure S6. Forge's parameters used for the build of the RVM model. S8

Figure S7. Forge's parameters used for the build of the SVM model. S8

Figure S8. Model statistics for the 3D-field model. S9

Figure S9. Model statistics for the kNN model. S9

Figure S10. q2 for kNN model. S10

Figure S11. kNN training set experimental vs. calculated K_i S10

Figure S12. kNN test set experimental vs. calculated K_i S11

Figure S13. RF training set experimental vs. calculated K_i S11

Figure S14. RF test set experimental vs. calculated K_i S12

Figure S15. RVM training set experimental vs. calculated K_i S12

Figure S16. RVM test set experimental vs. calculated K_i S13

Figure S17. Pharmit pharmacophore queries for PDB ID: 6a93 (left), 6a94 (centre) and 6wgt (right) S13

Table S1. SMILES, experimental and predicted (field and SVM) pKi values of the molecules in the training set S13

Table S2. SMILES, experimental and predicted (field and SVM) pKi values of the molecules in the test set S20

Table S3. Name of NPS molecules, experimental pEC50 and predicted (field and SVM) pKi values S21

Table S4. Calculated ${}_{field}pK_i$, ${}_{SVM}pK_i$ and ${}_{mean}pK_i$ for the natural products screening S22

Table S1bis. Structures, experimental and predicted (field and SVM) pKi values of the molecules in the training setS43

Table S2bis. Structures, experimental and predicted (field and SVM) pKi values of the molecules in the test set S103

Table S4bis. Calculated ${}_{field}pK_i$, ${}_{SVM}pK_i$ and ${}_{mean}pK_i$ for the natural products screening and structures and structures S120

Statistical analysis information for the models

The following conditions were used to calculate the field 3D-QSAR model (Figure S1-S3). The leave-one-out method was used for the validation of the QSAR model. The maximum number of components to extract from the PLS regression was set to 20. The number of Y scrambles to use was set to 50, this means that in each scramble the activity values are randomly assigned to molecules and the model building process is repeated. More scramble sets provide stronger confirmation of statistical significance. The sample point minimum distance threshold was set to 1 Å. This option checks the sphere exclusion algorithm used to reduce the initial number of field sample positions down to a smaller set. Decrease the value sample point minimum distance increases the number of sample points, which may improve the model at the expense of increasing the probability of over-fitting. A value of 1 Å means that sample points must be at least 1 Å aside from each other. The predictive ability of the generated model was confirmed by different statistical tests. The leave-one-out method was used during the validation of the QSAR model which means that the model is built again but a single molecule left out of the process, this is then repeated leaving out each training set molecule in turn. The predicted activity for each molecule is the value obtained when it was left out of the model building process. The cross-validation regression coefficient (q^2) was calculated based on the prediction error sum of squares (PRESS) and the sum of squares of deviation of the experimental values from their mean (SSY):

$$q^2 = 1 - \frac{PRESS}{SSY} = 1 - \frac{\sum_{i=1}^{n}(Yexp - Ypred)^2}{\sum_{i=1}^{n}(Yexp - Ymean)^2}$$

where *Yexp* is the experimental activity of training set compound, *Ypred* is the predicted activity of training set compound, and *Ymean* is the mean values of the activity of training set compound.

The performance of the model was also validated through the determination of the coefficient in prediction, r^2_{test}, using the following equation:

$$r^2 test = 1 - \frac{\sum_{i=1}^{n}(Ypredtest - Ytest)^2}{\sum_{i=1}^{n}(Ytest - Ymean)^2}$$

where *Ypredtest* is the predicted activity of test set compound by QSAR equation, *Ytest* is the experimental activity of test set compound, and *Ymean* is the mean values of the activity of training set compound.

As an alternative to field QSAR, Forge can use the electrostatic and shape properties of aligned molecules to develop QSAR models using various machine learning methods suitable for regression calculations.

The machine learning methods available in Forge, and used to generate the QSAR models, are k-Nearest Neighbors (kNN), Support Vector Machine, Relevance Vector Machine and Random Forest, all of them are supervised learning methods, i.e. the predictive model is built from training molecules for which we know the activity value or class.

k-Nearest Neighbors (kNN)

The algorithm works as follows:

1. Calculate the distance (use 1 - similarity) between a new compound and all the compounds in the Training Set;
2. Select k compounds in the Training Set most similar to the new compound, according to the distances calculated in step 1;
3. Regression models: predict the activity of the new compound as the weighted average activity of its k nearest neighbors in the Training Set.

The kNN model was built using the 3D (field and shape) similarity (Figure S4). The use of a 3D (field and shape) similarity metric requires the generation of correct alignments for all compounds with the lowest possible degree of noise. The same alignment used for the field 3D-QSAR model was used for the kNN model.

The optimal k value was selected by LOO (Leave-One-Out) cross-validation: each compound in the Training Set was removed in turn from the modelling and its activity was predicted as the average activity of its k nearest neighbors. These Training Set predicted activities are used to calculate a q2 value for the model (Figure S9 and S10). The k value and weighting scheme which gives the highest q2 were then selected as the optimal conditions for the kNN model (Figure S11 and S12).

Support Vector Machine & Relevance Vector Machine

In this method, the 3D descriptors for the Training Set molecules are plotted as points in *n*-dimensional space, where *n* is the number of the descriptors. In binary classification, the aim is to find the (n-1)-dimensional hyperplane in this space that best separates the two classes, by maximising the distances to the nearest points. The subset of data points that define this margin are termed *support vectors*. New molecules from a test or prediction set are then mapped into the same space and classified according to which side of the hyperplane they fall. If the data points cannot be separated linearly, a transformation can be applied to the points so that they can be represented in a higher dimensional space, in which they may be separable. This is known as the *kernel trick* and the functions that perform this operation are known as *kernels*. In Forge, the radial basis function (RBF) kernel is used, as this works well for a wide range of data, is relatively easy to optimise as it has only one hyperparameter and is numerically well-behaved.

SVM regression models (Figure S7)

1. The gamma parameter for the RBF kernel, determines how far the influence of a single training example reaches. Lower values of gamma increase the distance away from the hyperplane at which data points are considered. Higher gamma values allow more complex models at the risk of overfitting.
2. C is a regularisation parameter which controls accuracy vs. generalization. Higher values of C give a more accurate fit to the training data, with a higher likelihood of overfitting. Lower values of C result in a simpler decision boundary that may generalise better to unseen data.
3. The epsilon parameter specifies the tolerance to which the hyperplane is optimised, i.e. how closely the model should attempt to reproduce the data. lower epsilon values lead to a more closely fitted model, but higher susceptibility to noise.

Care was taken to avoid over/underfitting to the training data in selecting these parameters. An effective way to choose the gamma, C and epsilon parameters is through a k-fold cross-validation procedure, where the cross-validation process is repeated k times, each time leaving out 20% of the Training Set compounds. The cross-validation was carried out for various values of these parameters, which were chosen through a global

optimisation procedure, and the final model was built from the parameters which gave the best predictive power during cross-validation, as measured by the Root Mean Squared error from cross-validation.

Relevance Vector Machines (RVMs, Figure S6) have the same functional form as SVMs, but the training process uses Bayesian inference to estimate the parameters for the model. This therefore eliminates the complex process of choosing model parameters such as C and epsilon, although the RBF gamma parameter must still be chosen through a k-fold cross-validation procedure. The quality of the model is then evaluated by how accurate the predictions are. For regression problems, the parameter optimization continues until convergence.

Random Forest

Random Forest (Figure S5) is a regression method based upon decision trees.

To fit the regression problem using a decision tree, several splits were tried for the data for the selected 3D descriptors, and the split that gives the lowest error for the predicted activity value was chosen. This splitting process was continued recursively until the maximum tree depth was reached.

Random forests improve on decision trees by using an ensemble of decision trees, trained with bagging (bootstrap aggregating). Bagging means that each tree is built from a random sample of the original descriptors, which may include duplicate descriptors. The output for a prediction is then the mean prediction from all of the trees in the forest. The Random Forest procedure enhances stability and accuracy relative to using a decision tree and helps to avoid overfitting.

The following parameters were to train Random Forest model:

1. Number of trees. Generally, more trees will result in more accurate predictions.
2. Feature subsampling fraction. To introduce additional randomness, only a randomly selected fraction of the descriptors is considered at each split in the tree.
3. Minimum samples per leaf: defines the minimum node size, which implicitly limits the tree depth, reducing the risk of overfitting.

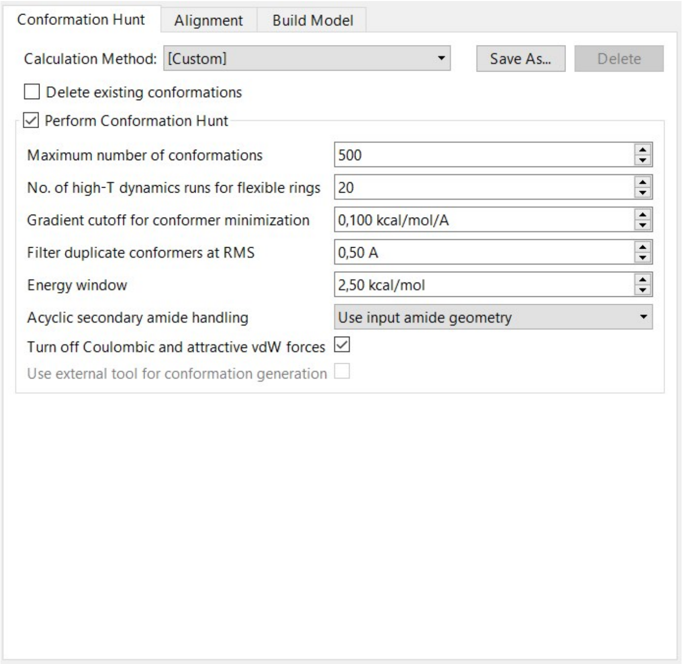

Figure S1. Forge's parameters used for the conformation hunt.

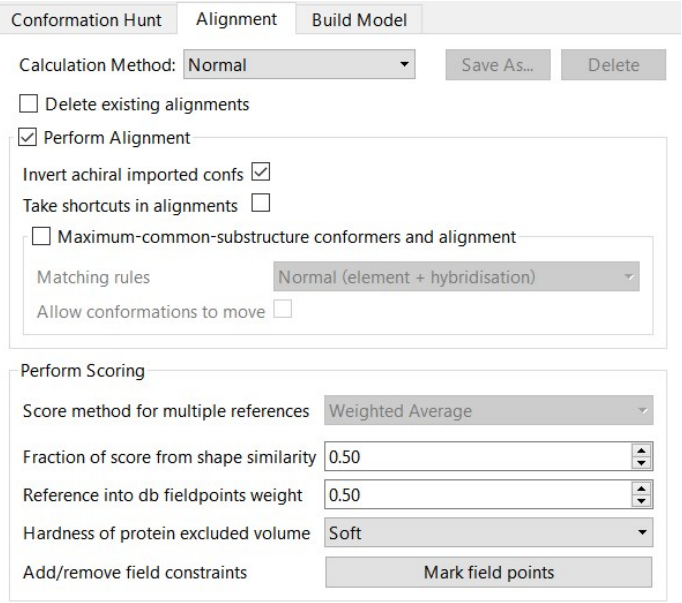

Figure S2. Forge's parameters used for the alignment.

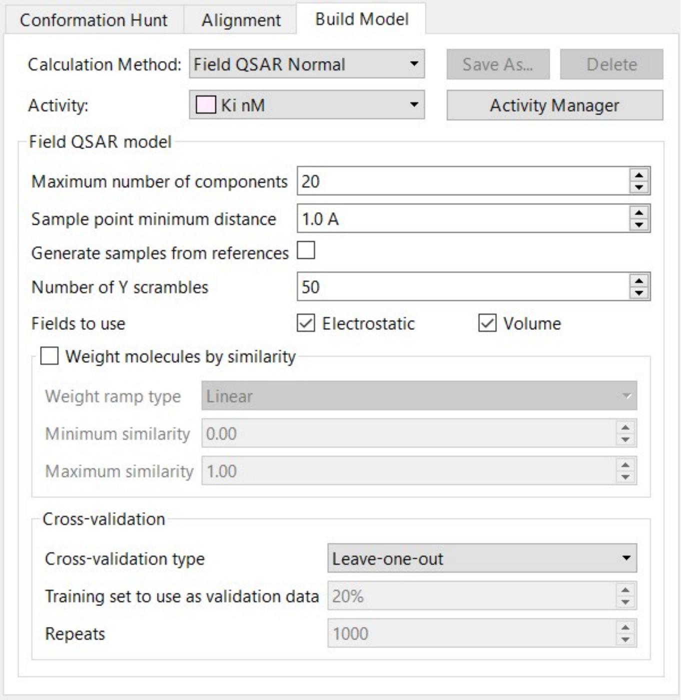

Figure S3. Forge's parameters used for the build of the field model.

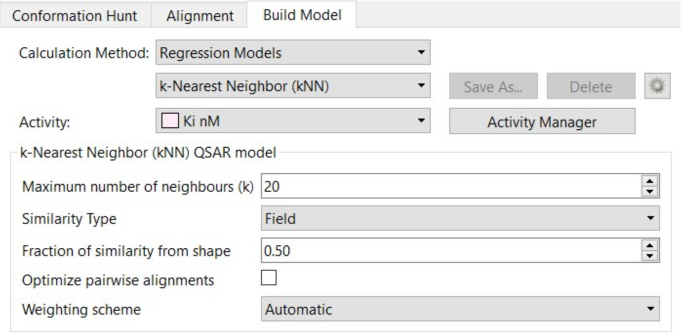

Figure S4. Forge's parameters used for the build of the kNN model.

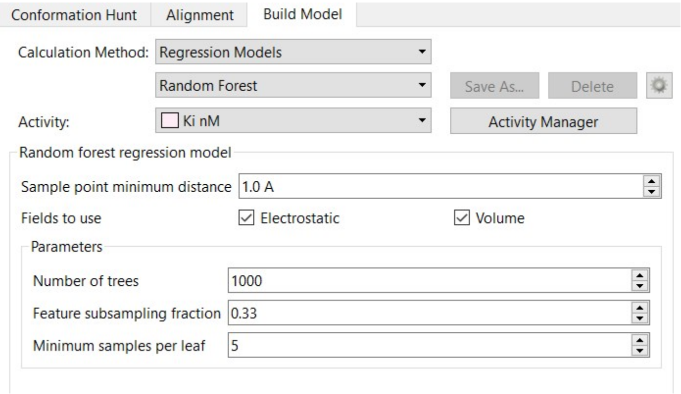

Figure S5. Forge's parameters used for the build of the RF model.

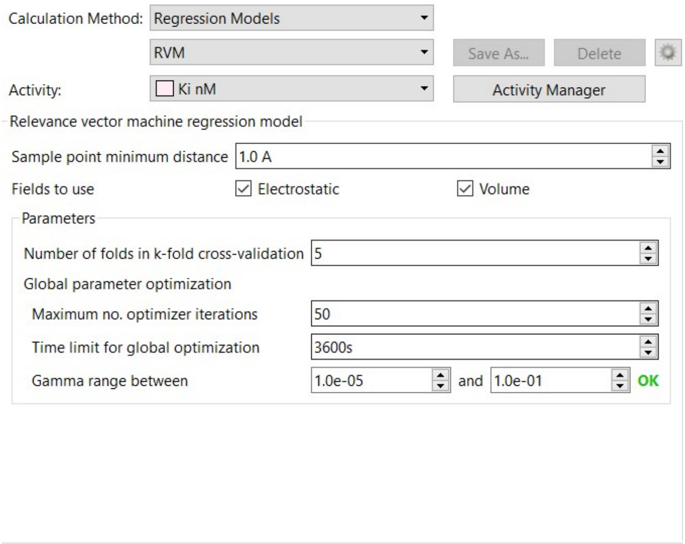

Figure S6. Forge's parameters used for the build of the RVM model.

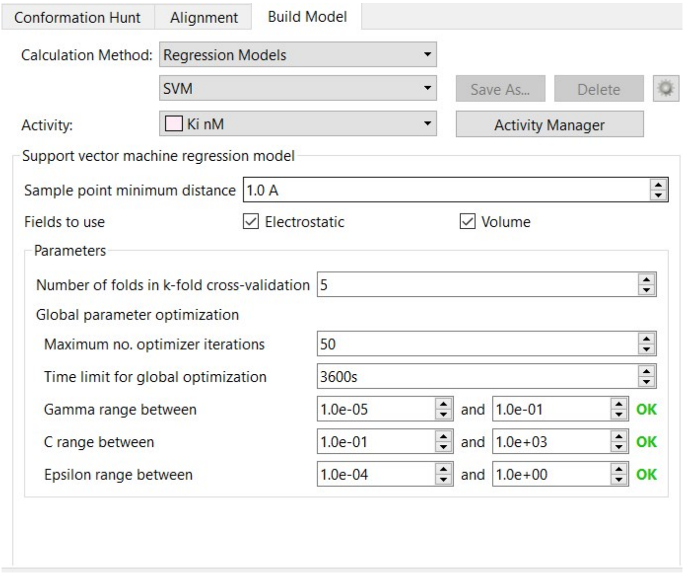

Figure S7. Forge's parameters used for the build of the SVM model.

Comps	R^2	Q^2	Test R^2	RMSE	RMSEpred	Tau	Tau-pred
0	-0.001	-0.007	-0.001	1.071	1.074	0.306	-0.963
1	0.511	0.448	0.398	0.750	0.798	0.514	0.474
2	0.721	0.644	0.653	0.563	0.639	0.647	0.590
3	0.794	0.721	0.678	0.485	0.565	0.705	0.647
4	0.847	0.744	0.695	0.417	0.542	0.751	0.667
5*	0.878	0.751	0.734	0.372	0.535	0.779	0.670
6	0.909	0.746	0.702	0.319	0.540	0.808	0.665
7	0.939	0.742	0.715	0.260	0.544	0.851	0.669
8	0.948	0.738	0.703	0.241	0.548	0.865	0.664
9	0.960	0.734	0.684	0.212	0.553	0.877	0.664
10	0.967	0.730	0.670	0.192	0.557	0.887	0.663
11	0.974	0.726	0.675	0.171	0.560	0.899	0.661
12	0.980	0.719	0.666	0.147	0.568	0.911	0.656
13	0.986	0.708	0.668	0.126	0.579	0.927	0.649
14	0.989	0.702	0.665	0.110	0.586	0.935	0.644
15	0.992	0.699	0.653	0.097	0.588	0.944	0.642
16	0.993	0.696	0.655	0.086	0.592	0.951	0.637
17	0.995	0.693	0.649	0.074	0.595	0.957	0.635
18	0.996	0.691	0.650	0.065	0.597	0.961	0.635
19	0.997	0.691	0.652	0.056	0.597	0.969	0.633
20	0.998	0.691	0.642	0.049	0.597	0.973	0.634

Figure S8. Model statistics for the 3D-field model.

Model statistics:
=================

K	Distance		Q^2	RMSEpred	Tau-pred
	Avg	Std. dev.			
0	0.00	1.00	-0.007	1.075	-0.991
1	0.28	0.09	0.485	0.769	0.557
2	0.29	0.09	0.635	0.647	0.597
3*	0.31	0.08	0.661	0.624	0.611
4	0.32	0.08	0.660	0.625	0.608
5	0.32	0.08	0.668	0.617	0.610
6	0.33	0.07	0.656	0.629	0.605
7	0.34	0.07	0.656	0.628	0.600
8	0.34	0.07	0.651	0.633	0.597
9	0.35	0.07	0.650	0.634	0.598
10	0.35	0.07	0.652	0.632	0.595
11	0.35	0.07	0.645	0.638	0.591
12	0.36	0.06	0.644	0.639	0.590
13	0.36	0.06	0.638	0.645	0.585
14	0.37	0.06	0.634	0.648	0.583
15	0.37	0.06	0.632	0.650	0.586
16	0.37	0.06	0.630	0.652	0.582
17	0.37	0.06	0.628	0.653	0.580
18	0.38	0.05	0.625	0.656	0.580
19	0.38	0.05	0.623	0.658	0.579
20	0.38	0.05	0.615	0.664	0.574

Best value of K: 3

Figure S9. Model statistics for the kNN model.

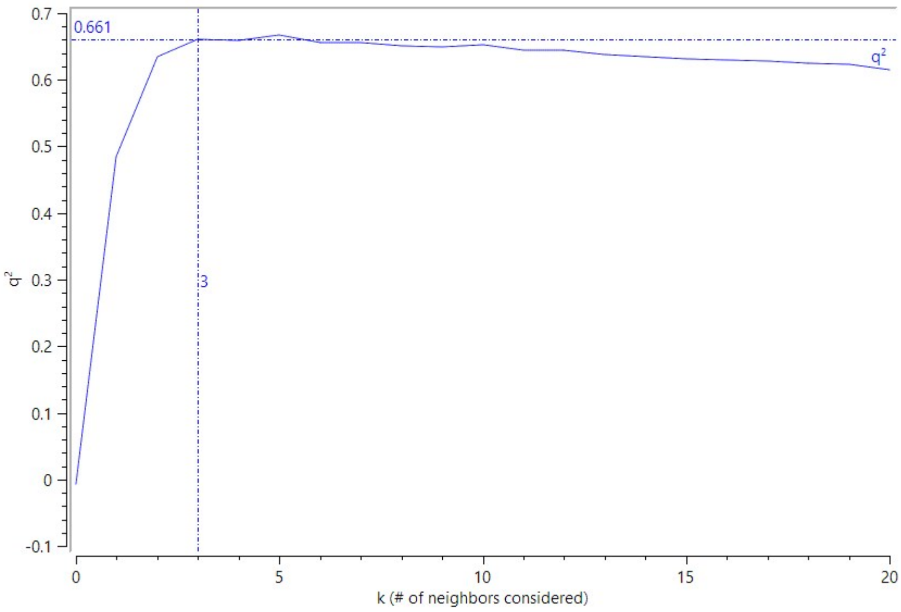

Figure S10. q^2 for kNN model.

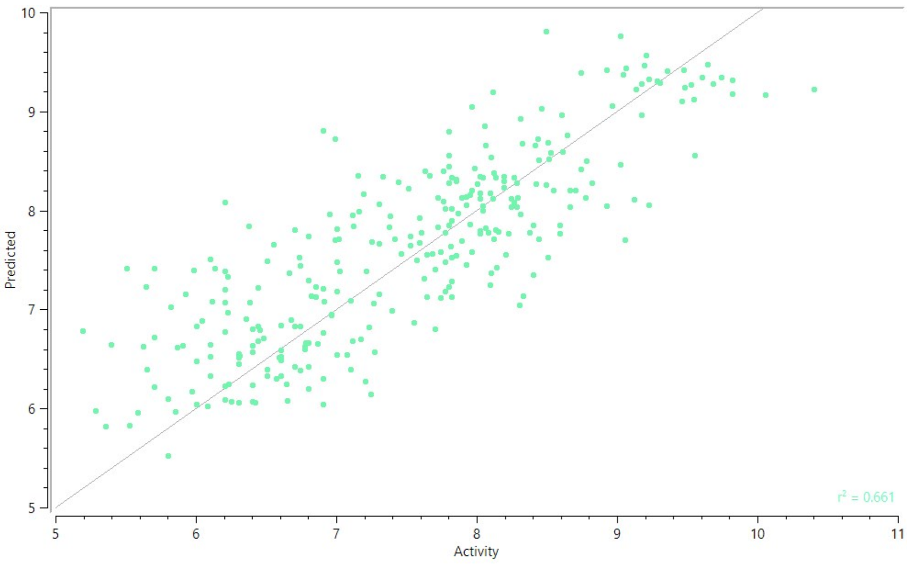

Figure S11. kNN training set experimental vs. calculated K_i

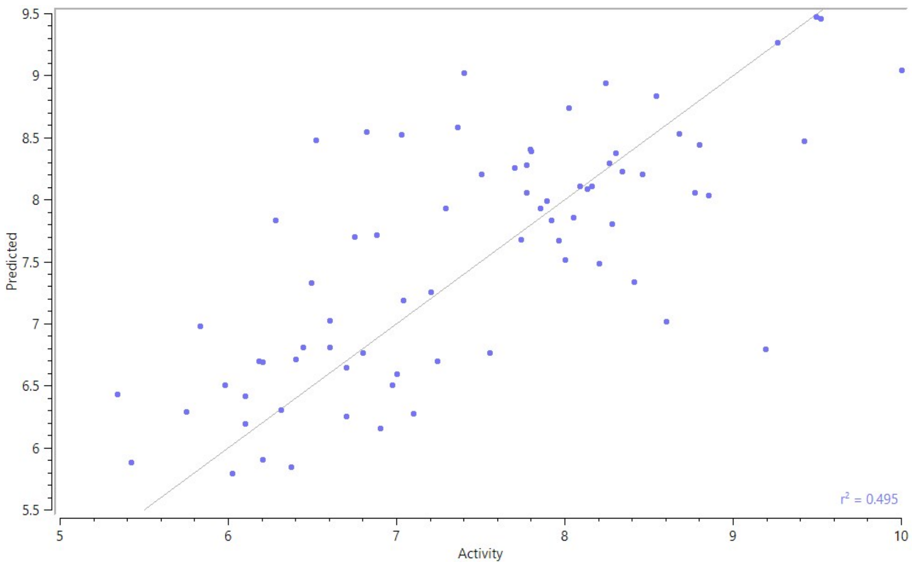

Figure S12. kNN test set experimental vs. calculated K_i

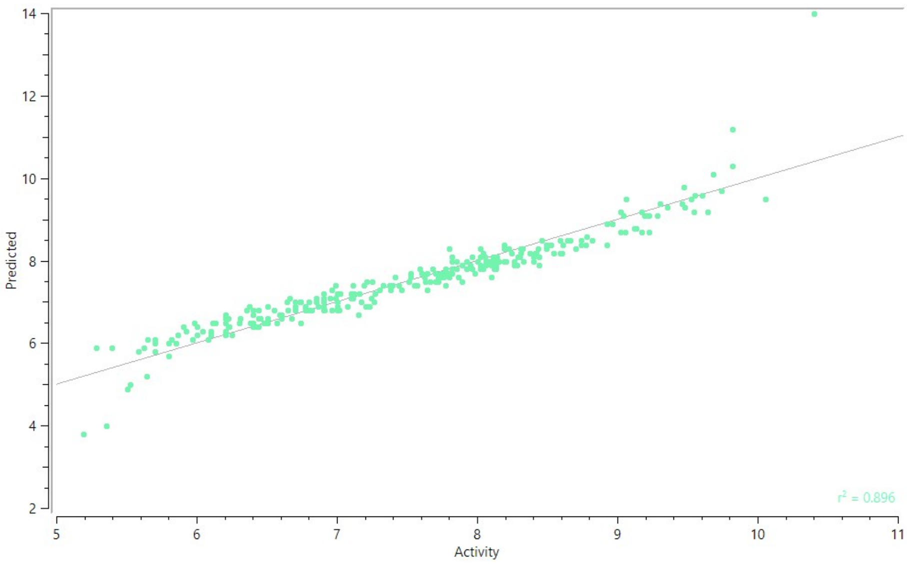

Figure S13. RF training set experimental vs. calculated K_i

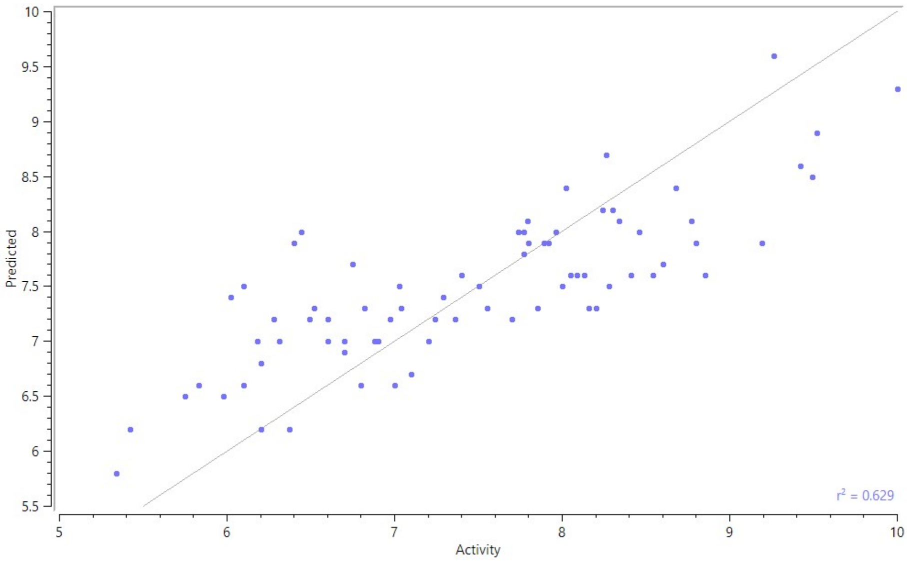

Figure S14. RF test set experimental vs. calculated K_i

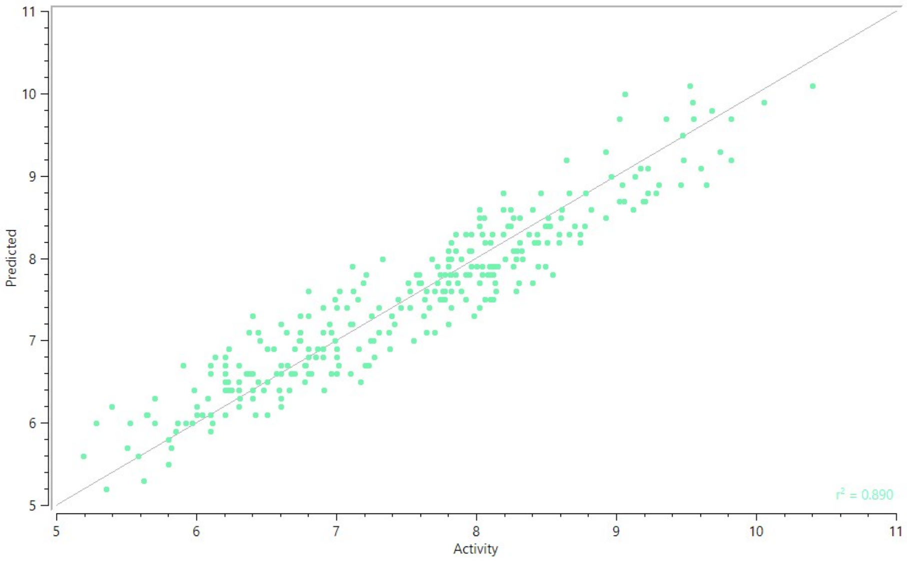

Figure S15. RVM training set experimental vs. calculated K_i

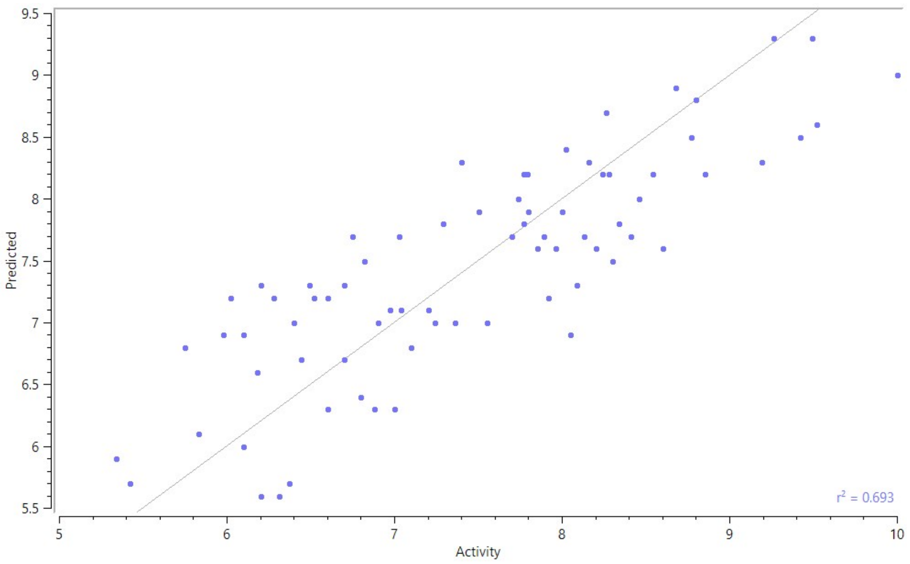

Figure S16. RVM test set experimental vs. calculated K_i

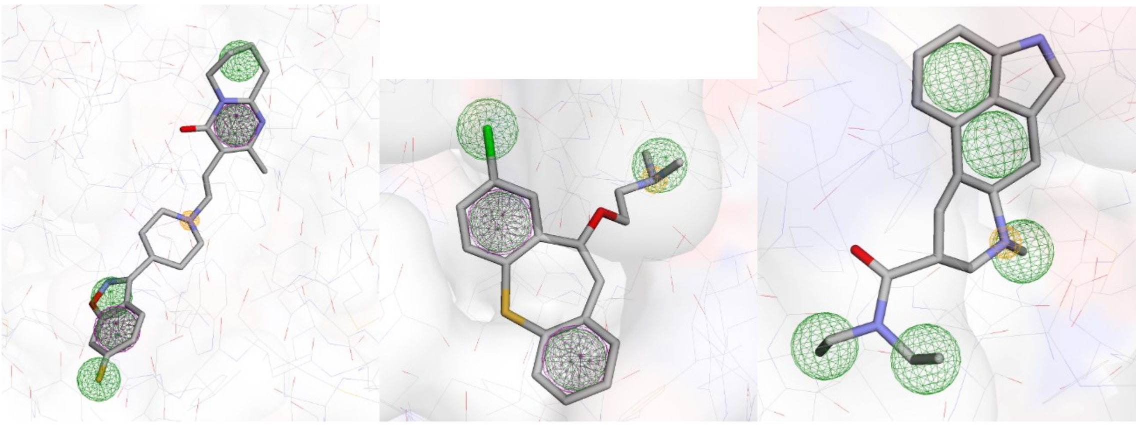

Figure S17. Pharmit pharmacophore queries for PDB ID: 6a93 (left), 6a94 (centre) and 6wgt (right).

Table S1. SMILES, experimental and predicted (field and SVM) pK_i values of the molecules in the training set

n	SMILES	$_{exp}$pK_i	$_{field}$pK_i	$_{SVM}$pK_i
1	O=C(N[C@H]1CCc2ccc(CC[NH+]3CCN(CC3)c4c5ccccc5sn4)cc21)Cc6ccccc6	10.4	10.4	10.4
2	[NH3+][C@@H]1c2cc(CC[NH+]3CCN(CC3)c4c5ccccc5sn4)ccc2CC1	10.1	9.8	10.0
3	FC(F)(F)C(=O)N[C@H]1CCc2cc(CC[NH+]3CCN(CC3)c4c5ccccc5sn4)ccc21	9.8	9.5	9.8
4	O=C(NC1CCC(CC[NH+]2CCN(CC2)c3c4ccccc4sn3)CC1)c5cccs5	9.8	9.4	9.8
5	O=C(OCC)NC1CCC(CC[NH+]2CCN(CC2)c3c4ccccc4sn3)CC1	9.7	9.4	9.6
6	O=C(NC1CCC(CC[NH+]2CCN(CC2)c3c4ccccc4sn3)CC1)Nc5ccccc5	9.7	10.0	9.7
7	O=C(NC1CCC(CC[NH+]2CCN(CC2)c3c4ccccc4sn3)CC1)c5ccco5	9.6	9.0	9.3

8	O=C(N[C@H]1CCc2cc(CC[NH+]3CCN(CC3)c4c5ccccc5sn4)ccc21)CC	9.6	9.2	9.6
9	Fc1ccc(C(=O)C2CC[NH+](CC2)CCN3C(=O)Nc4ccccc4C3=O)cc1	9.6	9.7	9.5
10	O=C(NC1CCC(CC[NH+]2CCN(CC2)c3c4ccccc4sn3)CC1)c5cnccc5	9.5	9.6	9.5
11	O=C(N[C@H]1CCc2ccc(CC[NH+]3CCN(CC3)c4c5ccccc5sn4)cc21)C6CC6	9.5	10.2	9.5
12	O=C(N[C@H]1CCc2cc(CC[NH+]3CCN(CC3)c4c5ccccc5sn4)ccc21)C	9.5	9.0	9.5
13	O=S(=O)(NC1CCC(CC1)CC[NH+]2CCN(CC2)c3c4ccccc4sn3)CCC	9.5	10.0	9.5
14	O=C(NC1CCC(CC[NH+]2CCN(CC2)c3c4ccccc4sn3)CC1)C5CC5	9.5	9.2	9.5
15	O=C(N[C@H]1CCc2ccc(CC[NH+]3CCN(CC3)c4c5ccccc5sn4)cc21)CCC	9.4	10.0	9.4
16	O=C(NC1CCC(CC1)CC[NH+]2CCN(CC2)c3c4ccccc4sn3)N(CC)C	9.3	9.1	9.3
17	O=C(OCC)NC1CCC(CC[NH+]2CCN(CC2)c3c4ccccc4sn3)CC1	9.3	8.8	9.2
18	O=C(NC1CCC(CC1)CC[NH+]2CCN(CC2)c3c4ccccc4sn3)N(C)C	9.2	9.2	9.2
19	Fc1ccc2c(onc2C3CC[NH+](CC3)CCCCNS(=O)(=O)c4cccc5ccccc54)c1	9.2	9.0	9.2
20	FC(F)(F)C(=O)NC1CCC(CC[NH+]2CCN(CC2)c3c4ccccc4sn3)CC1	9.2	8.8	9.2
21	O=C(N[C@H]1CCc2cc(CC[NH+]3CCN(CC3)c4c5ccccc5sn4)ccc21)C(C)C	9.2	8.6	9.2
22	FC1(S(=O)(=O)c2ccccc2)CC[NH+](CC1)CCc3ccc(F)cc3F	9.2	8.6	8.9
23	O=C(N[C@H]1CCc2cc(CC[NH+]3CCN(CC3)c4c5ccccc5sn4)ccc21)c6cnccc6	9.2	9.2	9.2
24	Fc1ccc(C(=O)CCC[NH+]2CC=C(N3C(=O)Nc4ccccc43)CC2)cc1	9.1	9.3	9.1
25	O=C(N[C@H]1CCc2ccc(CC[NH+]3CCN(CC3)c4c5ccccc5sn4)cc21)C(C)C	9.1	8.7	9.1
26	O=C(NC1CCC(CC[NH+]2CCN(CC2)c3c4ccccc4sn3)CC1)CCC	9.1	9.5	9.2
27	O=S(=O)(NCC[NH+]1CCN(CC1)c2c3ccccc3sn2)c4ccc5ccccc5c4	9.1	8.9	9.0
28	O=C(NC1CCC(CC[NH+]2CCN(CC2)c3c4ccccc4sn3)CC1)NCc5ccccc5	9.0	9.2	9.0
29	O=C(N[C@H]1CCc2ccc(CC[NH+]3CCN(CC3)c4c5ccccc5sn4)cc21)c6cnccc6	9.0	8.7	9.0
30	O=C(N[C@H]1CCc2ccc(CC[NH+]3CCN(CC3)c4c5ccccc5sn4)cc21)CCc6ccccc6	9.0	9.6	9.0
31	O=C(NC1CCC(CC[NH+]2CCN(CC2)c3c4ccccc4sn3)CC1)CC	9.0	9.0	9.0
32	O=S(=O)(N(C1CCC(CC1)CC[NH+]2CCN(CC2)c3c4ccccc4sn3)C)c5cccs5	8.9	9.6	8.9
33	Fc1ccc(C(=O)CCC[NH+]2CCC(n3c(O)nc4ccccc43)CC2)cc1	8.9	8.4	8.9
34	Clc1cccc(N2CC[NH+](CCCCOc3ccc4c(NC(=O)CC4)n3)CC2)c1C	8.8	8.6	8.8
35	Fc1ccc(C(=O)N[C@H]2CCc3cc(CC[NH+]4CCN(CC4)c5c6ccccc6sn5)ccc32)cc1	8.8	8.7	8.8
36	Fc1ccc2c(onc2C3CC[NH+](CC3)CCCNS(=O)(=O)c4cccc5ccccc54)c1	8.8	8.1	8.8
37	Fc1ccc2c(onc2C3CC[NH+](CC3)CCNS(=O)(=O)c4ccc5ccccc5c4)c1	8.7	8.2	8.7
38	O=C(NC1CCC(CC[NH+]2CCN(CC2)c3c4ccc([N+]([O-])=O)cc4sn3)CC1)c5ccco5	8.7	8.5	8.7
39	Clc1c(Cl)cccc1N2CC[NH+](CCC2)CCCCOc3ccc4c(NC(=O)CC4)c3	8.7	8.5	8.7
40	O=C(N1CCC(CC[NH+]2CCN(CC2)c3c4ccccc4sn3)CC1)c5ccco5	8.7	8.5	8.7
41	FC(F)(F)Oc1ccc(S(=O)(=O)NCCCC[NH+]2CCN(CC2)c3c4ccccc4sn3)cc1	8.7	8.9	8.7
42	Fc1ccc2c(c(c(S(=O)(=O)NCCC[NH+]3CCC(CC3)c4c5ccc(F)cc5on4)s2)C)c1	8.6	9.2	8.6
43	Fc1ccc(C(=O)N[C@H]2CCc3ccc(CC[NH+]4CCN(CC4)c5c6ccccc6sn5)cc32)cc1	8.6	8.5	8.6
44	O[C@H](C1CC[NH+](CC1)CCc2ccccc2)c3ccccc3	8.6	8.4	8.6
45	Clc1ccc2c(sc(S(=O)(=O)NCCC[NH+]3CCC(CC3)c4c5ccc(F)cc5on4)c2)c1	8.6	8.5	8.6
46	Clc1c(Cl)cccc1N2CC[NH+](CC2)CCCC(=O)Nc3ccn4c(c(cn4)/C=N\O)c3	8.6	8.7	8.6
47	Fc1ccc(C(=O)CCC[NH+]2CC[C@H]3[C@H](C2)c4cccc5CCN3c54)cc1	8.5	7.7	8.1
48	O=C(N[C@H]1CCc2ccc(CC[NH+]3CCN(CC3)c4c5ccccc5sn4)cc21)c6ccc(C)cc6	8.5	8.3	8.5
49	Clc1ccc2c(ccc(S(=O)(=O)NCCC[NH+]3CCC(CC3)c4c5ccc(F)cc5on4)c2)c1	8.5	8.7	8.5
50	O=C1c2cc(OCCC[NH+]3CCN(CC3)c4cccc5CCCc54)ccc2CN1	8.5	8.2	8.5
51	Fc1ccc(OCCC[NH+]2CCC(N3C(=O)Nc4ccccc43)CC2)cc1	8.5	8.3	8.5
52	O=C(N1CCOCC1)NC2CCC(CC2)CC[NH+]3CCN(CC3)c4c5ccccc5sn4	8.5	8.5	8.5
53	Clc1c(Cl)cccc1N2CC[NH+](CC2)CCCCOc3ncc4C=CC(=O)Nc4n3	8.5	8.1	8.5
54	Fc1ccc2c(onc2C3CC[NH+](CCNS(=O)(=O)c4cccc(c4)C)CC3)c1	8.5	8.8	8.5
55	Fc1ccccc1S(=O)(=O)c2cnc(cc2)/C=C/c3ccccc3O	8.4	8.2	8.4
56	Fc1ccc2c(onc2C3CC[NH+](CC3)CCCCNS(=O)(=O)c4ccc(OC(F)(F)F)cc4)c1	8.4	8.3	8.4

57	Fc1ccc2c(onc2C3CC[NH+](CCCNS(=O)(=O)c4cccc(c4)C)CC3)c1	8.4	8.2	8.4
58	Fc1ccc2c(onc2C3CC[NH+](CCCNS(=O)(=O)c4ccc(OC(F)(F)F)cc4)CC3)c1	8.4	8.5	8.4
59	Fc1cc(F)ccc1CCC2CCN(CC2)C(=O)c3ccccc3F	8.4	7.8	8.4
60	FC(F)(F)c1cccc(N2CC[NH+](CCCCNC(=O)c3cc4ccccc4[nH]3)CC2)c1	8.4	7.5	8.4
61	Fc1ccc2c(scc2N3CC[NH+](CC3)CCCCN4C(=O)C5(S[C@H]4C)CCCC5)c1	8.4	7.8	8.4
62	O=C1c2cc(OCCC[NH+]3CCN(CC3)c4cccc5c4CCO5)ccc2CN1	8.4	8.3	8.4
63	Fc1ccc(C(=O)CCC[NH+]2CC[C@H]3[C@H](C2)c4cccc5c4N3CCN5Cc6ccccc6)cc1	8.3	8.0	8.3
64	O=C(N[C@H]1CCc2ccc(CC[NH+]3CCN(CC3)c4c5ccccc5sn4)cc21)c6ccccc6	8.3	8.3	8.3
65	O=C1c2cc(OCCC[NH+]3CCN(CC3)c4cccc5CCCCc54)ccc2CN1	8.3	8.6	8.3
66	Clc1c(Cl)ccc(S(=O)(=O)NCCCC[NH+]2CCC(CC2)c3c4ccc(F)cc4on3)c1	8.3	8.6	8.3
67	Clc1ccc(OCC(F)(F)c2ccccc2)c(OC3C[NH2+]C3)c1	8.3	8.0	8.3
68	Fc1ccc2c(onc2C3CC[NH+](CC3)C[C@H]4CC(=O)c5ccoc5C4)c1	8.3	8.4	8.3
69	Fc1ccc(C(=O)CCC[NH+]2CC[C@H]3[C@H](C2)c4cccc5c4N3C(CN5C)(C)C)cc1	8.3	7.9	8.3
70	Fc1ccc(C(=O)C2CC[NH+](CC2)CCCCc3ccccc3)cc1	8.3	7.7	7.7
71	Fc1ccc2c(onc2C3CC[NH+](CC3)C[C@H]4CC(=O)c5ccoc5C4)c1	8.3	8.4	8.3
72	Fc1ccc(C(=O)CCC[NH+]2CC[C@H]3[C@H](C2)c4cccc5c4N3CCN5)cc1	8.3	8.4	8.3
73	O=C1c2cc(OCCCC[NH+]3CCN(CC3)c4cccc5CCOCc54)ccc2CN1	8.3	7.7	8.3
74	O=C(CCC[NH+]1CC[C@H]2[C@@H](C1)c3cccc4c3N2CCN4C)c5ccccc5	8.2	8.5	8.2
75	Fc1ccc2c(onc2C3CC[NH+](CC3)CCCCNS(=O)(=O)c4csc5ccccc54)c1	8.2	8.6	8.2
76	O=S(=O)(NCCCC[NH+]1CCN(CC1)c2c3ccccc3sn2)c4cc5ccccc5s4	8.2	8.3	8.2
77	O=C(Nc1ccc(OC)c(CCC[NH+](C)C)c1)c2ccc(-c3ccc(cc3C)-c4noc(n4)C)cc2	8.2	7.8	8.2
78	Clc1ccc2c(sc(S(=O)(=O)NCCCC[NH+]3CCN(CC3)c4c5ccccc5sn4)c2)c1	8.2	8.9	8.2
79	Fc1ccc2c(c(c(S(=O)(=O)NCCCC[NH+]3CCN(CC3)c4c5ccccc5sn4)s2)C)c1	8.2	8.7	8.2
80	Fc1ccc(C(=O)C2CC[NH+](CC2)C[C@H]3CC(=O)c4cnc(NC)nc4C3)cc1	8.2	8.3	8.2
81	Clc1c(Cl)cccc1N2CC[NH+](CC2)CCc3cn(nn3)-c4ccn5c(c4)ccn5	8.2	7.9	8.1
82	Clc1c(Cl)cccc1N2CC[NH+](CCCCNC(=O)c3cc4ccccc4o3)CC2	8.1	7.9	8.1
83	Fc1ccc2c(c(c(S(=O)(=O)NCCCC[NH+]3CCN(CC3)c4c5ccccc5on4)s2)C)c1	8.1	7.6	8.1
84	Fc1ccc(/C=C/c2ccc(S(=O)(=O)c3ccccc3)cn2)cc1	8.1	7.7	8.0
85	C[NH+]1CCC(CC1)=C2c3ccsc3CCc4ccccc42	8.1	7.5	8.0
86	O=S(=O)(NCCCC[NH+]1CCN(CC1)c2c3ccccc3sn2)c4cccc5c4ncs5	8.1	7.7	8.1
87	Clc1ccc2c(ccc(S(=O)(=O)NCCCC[NH+]3CCN(CC3)c4c5ccccc5on4)c2)c1	8.1	8.3	8.1
88	Fc1ccc(C(=O)CCC[NH+]2CC[C@@H]3[C@H](C2)c4cccc5CCCN3c54)cc1	8.1	8.0	8.1
89	Fc1ccc2c(scc2N3CC[NH+](CC3)CCCCN4C(=O)C(S[C@H]4C)(C)C)c1	8.1	7.6	8.1
90	Fc1ccc(N2CC[NH+](CC2)CCCCNC(=O)c3cc4ccccc4cn3)cc1	8.1	7.7	8.1
91	O=S(=O)(NCCCC[NH+]1CCN(CC1)c2c3ccccc3sn2)c4cnc5ccccn54	8.1	7.7	8.1
92	Clc1ccc2c(ccc(S(=O)(=O)NCCCC[NH+]3CCN(CC3)c4c5ccccc5sn4)c2)c1	8.1	7.9	8.1
93	O=S(=O)(NCCC[NH+]1CCN(CC1)c2c3ccccc3sn2)c4cccc5c4ncs5	8.1	7.8	8.1
94	O=S(=O)(NCC[NH+]1CCN(CC1)c2c3ccccc3sn2)c4cccc(c4)C	8.1	8.2	8.1
95	Clc1c(Cl)cccc1N2CC[NH+](CC2)CCCCOc3ccc4c(NC(=O)CC4)c3	8.1	7.6	8.1
96	O=S(=O)(N1CCC[C@@H]1CC[NH+]2CCN(CC2)c3cccc4c3ccs4)c5cccc6cnccc65	8.1	8.5	8.1
97	Fc1ccc2c(onc2C3CC[NH+](CCCNS(=O)(=O)c4cccc5c4ncs5)CC3)c1	8.0	7.8	8.0
98	Clc1ccc([C@](OCC[C@@H]2CCC[NH+]2C)(c3ccccc3)C)cc1	8.0	7.6	8.0
99	O=S(=O)(NCCC[NH+]1CCN(CC1)c2c3ccccc3sn2)c4cccs4	8.0	8.2	8.0
100	Fc1ccc2c(onc2C3CC[NH+](CC3)CCCCNS(=O)(=O)c4cccs4)c1	8.0	8.4	8.0
101	Clc1ccc2c(CCc3cccnc3C2=C4CC[NH2+]CC4)c1	8.0	7.4	7.9
102	Fc1ccc2c(onc2C3CC[NH+](CC3)CCCCNS(=O)(=O)c4cnc5ccccn54)c1	8.0	7.7	8.0
103	FC(F)(F)Oc1ccc(S(=O)(=O)NCCC[NH+]2CCN(CC2)c3c4ccccc4sn3)cc1	8.0	8.2	8.0
104	O=C(C1CC[NH+](CC1)CCc2ccccc2)c3ccccc3	8.0	8.5	8.0
105	O=C1c2cc(OCCCC[NH+]3CCN(CC3)c4cccc5CCCc54)ccc2CN1	8.0	7.8	8.0

106	Clc1cccc(N2CC[NH+](CCCCOc3ccc4c(NC(=O)CC4)n3)CC2)c1F	8.0	7.4	8.0
107	O=S(=O)(NCCC[NH+]1CCN(CC1)c2c3ccccc3sn2)c4cc5ccccc5s4	8.0	8.4	8.0
108	Clc1ccc2c(NC(=O)N2C3CC[NH+](CC3)CCCN4C(=O)Nc5ccccc54)c1	8.0	8.1	8.0
109	Clc1ccc2c(sc(S(=O)(=O)NCCCC[NH+]3CCN(CC3)c4c5ccccc5on4)c2)c1	8.0	7.8	8.0
110	O=C1c2cc(OCCC[NH+]3CCN(CC3)c4cccc5CCOCc54)ccc2CN1	8.0	7.6	8.0
111	O=C1c2cc(OCCCC[NH+]3CCN(CC3)c4cccc5COCCc54)ccc2CN1	8.0	8.2	7.9
112	Fc1ccc2c(onc2C3CC[NH+](CC[C@@H]4CCCN4S(=O)(=O)c5cccc6c5cccn6)CC3)c1	7.9	7.6	7.9
113	Clc1c(Cl)ccc(S(=O)(=O)NCCCC[NH+]2CCN(CC2)c3c4ccccc4sn3)c1	7.9	8.2	7.9
114	Clc1c(C(F)(F)F)cccc1N2CC[NH+](CCCCOc3ccc4c(NC(=O)CC4)n3)CC2	7.9	7.7	7.9
115	Fc1ccc2c([C@@H]3[C@H](C[C@H](O3)C[NH+](C)C)c4ccccc4S2)c1	7.9	7.4	7.9
116	O=S(=O)(NCCCC[NH+]1CCN(CC1)c2c3ccccc3on2)c4cnc5ccccn54	7.9	7.9	7.9
117	Fc1cc(OC2CCC(CC2)(C)C)c(F)cc1CNC(=O)[C@@H]3CCC[NH2+]3	7.9	7.7	7.9
118	Fc1ccc2c(c(c(S(=O)(=O)NCCC[NH+]3CCN(CC3)c4c5ccccc5sn4)s2)C)c1	7.9	7.8	7.8
119	Fc1ccc(C(=O)CCC[NH+]2CC[C@H]3[C@H](C2)c4cccc5c4N3CCN5CCCC)cc1	7.9	8.2	7.9
120	Clc1c(F)ccc(S(=O)(=O)NCCC[NH+]2CCN(CC2)c3c4ccccc4sn3)c1	7.9	8.4	7.9
121	O=C(NCCCC[NH+]1CCN(CC1)c2cccc(c2)C)c3cc4ccccc4cn3	7.8	7.4	7.8
122	Fc1ccc(C(=O)CCC[NH+]2CCC(S(=O)(=O)c3ccccc3)CC2)cc1	7.8	7.9	7.8
123	O=S(=O)(C1CC[NH+](CC1)CCCc2ccccc2)c3ccccc3	7.8	8.2	7.8
124	Clc1c(F)ccc(S(=O)(=O)NCCCC[NH+]2CCN(CC2)c3c4ccccc4sn3)c1	7.8	7.5	7.8
125	Clc1c(Cl)cccc1N2CC[NH+](CC2)CCCCc3cn(nn3)-c4ccn5c(c4)c(cn5)C=O	7.8	8.2	7.8
126	O=S(=O)(N1CCC[C@@H]1CC[NH+]2CCN(CC2)c3c4ccccc4sn3)c5cccc6cnccc65	7.8	8.0	7.8
127	Clc1c(Cl)cccc1N2CC[NH+](CC[C@H](O)CNC(=O)c3ccc(-c4ccccn4)cc3)CC2	7.8	7.8	7.8
128	Clc1ccccc1N2CC[NH+](CCCCOc3ccc4c(NC(=O)CC4)n3)CC2	7.8	7.5	7.8
129	O=S(=O)(N1CCC[C@@H]1CC[NH+]2CCN(CC2)c3c4ccccc4sn3)c5cccc6c5cccn6	7.8	8.6	7.8
130	O1c2ccccc2CCC31CC[NH+](CC3)CCc4ccccc4	7.8	8.1	7.8
131	Clc1ccc2c(CCC3(CC[NH+](CC3)CCc4ccccc4)C2=O)c1	7.8	7.9	7.8
132	Fc1cc(F)ccc1CC[NH+]2CCC3(CC2)C=Cc4cc(F)ccc4O3	7.8	7.3	7.5
133	Clc1c(Cl)cccc1N2CC[NH+](CC2)CCCCOc3ccn4c(c3)cc(n4)CO	7.8	7.9	7.8
134	Clc1c(Cl)cccc1N2CC[NH+](CCCCNC(=O)c3cc4ccccc4s3)CC2	7.8	7.8	7.8
135	O=C(NCCCC[NH+]1CCN(CC1)c2cccc(c2)C)c3ccc4ccccc4n3	7.8	7.4	7.8
136	O=C1c2cc(OCCCC[NH+]3CCN(CC3)c4cccc5c4OCCC5)ccc2CN1	7.8	7.4	7.8
137	Fc1ccc(S(=O)(=O)c2cnc(cc2)/C=C/c3ccc(F)cc3)cc1	7.8	7.2	7.6
138	Fc1ccc(C(=O)C2CC[NH+](C[C@@H]3CC(=O)c4cc(oc4C3)-c5ccccc5)CC2)cc1	7.8	7.8	7.8
139	Fc1ccc2c(onc2C3CC[NH+](CC3)C[C@H]4CC(=O)c5cnn(c5C4)C)c1	7.8	7.5	7.8
140	Fc1ccccc1N2CC[NH+](CCCCNC(=O)c3cc4ccccc4o3)CC2	7.7	7.4	7.7
141	Fc1ccc2c([nH]nc2CCC[NH+]3CC[C@H]4[C@@H](C3)c5cccc6c5N4CCN6C)c1	7.7	7.7	7.7
142	Fc1ccc2c(onc2C3CC[NH+](CC3)CC[C@@H]4C(=O)c5cc([N+]([O-])=O)ccc5CC4)c1	7.7	8.0	7.7
143	Fc1ccc(S(=O)(=O)c2ccc(CCc3ccc(F)cc3)cn2)cc1	7.7	8.0	7.7
144	Fc1ccc2c(c(CCC[NH+]3CCN(CC3)c4cccc5c4O[C@@H](C(=O)N5)C)c[nH]2)c1	7.7	7.3	7.7
145	[O-]C(=O)C[C@@H]1CC[NH+]([C@H]2CC[C@]3(C2)c4ccccc4Cc5ccccc5C3)C1	7.7	6.9	7.2
146	O=S(=O)(N1CCC[C@@H]1CC[NH+]2CCN(CC2)c3c4ccccc4sn3)c5cccc6c5cccn6	7.7	7.8	7.7
147	O=C(NCCCC[NH+]1CCN(CC1)c2ccccc2)[C@@H]3CCc4ccccc4N3	7.7	7.2	7.7
148	Clc1c(Cl)cccc1N2CC[NH+](CCCCNC(=O)c3ccc(-c4ccccn4)cc3)CC2	7.6	7.6	7.6
149	[NH3+]C[C@H]1c2ccccc2Cc3cc(OCCCCC)ccc31	7.6	7.3	7.5
150	Clc1ccc(N2CC[NH+](CCC2)CCCC(=O)c3ccc(F)cc3)cc1	7.6	7.5	7.6
151	O=C(Nc1ccccc1SCCC[NH+](C)C)/C=C/c2ccccc2	7.6	7.4	7.6
152	O=C1c2cc(OCCC[NH+]3CCN(CC3)c4cccc5c4OCCC5)ccc2CN1	7.6	7.4	7.6
153	O=C(CCC[NH+]1CCC(OC(c2ccccc2)c3ccccc3)CC1)c4ccc(C(C)(C)C)cc4	7.6	8.2	7.6
154	O=S(=O)(N1CCCCC[C@@H]1CC[NH+]2CCN(CC2)c3c4ccccc4sn3)c5ccnc6ccccc65	7.6	8.0	7.6

155	O=C(NCCCC[NH+]1CCN(CC1)c2cccc([nH+]2)C)c3cc4ccccc4o3	7.6	8.0	7.6
156	Fc1ccc(S(=O)(=O)c2ccc(cn2)/C=C\c3ccc(F)cc3)cc1	7.6	7.1	7.5
157	O=S(=O)(N1CC[C@@H]1CC[NH+]2CCN(CC2)c3c4ccccc4sn3)c5cccc6c5cccn6	7.5	7.3	7.5
158	Fc1ccc(C(=O)C2CC[NH+](CC2)C[C@@H]3CC(=O)c4cnn(c4C3)C)cc1	7.5	7.4	7.5
159	Clc1c(Cl)cccc1N2CC[NH+](CCCCNC(=O)c3cc4ccccc4[nH]3)CC2	7.5	7.4	7.5
160	O=S(=O)(N1CCCC[C@@H]1CC[NH+]2CCN(CC2)c3cccc4c3ccs4)c5cccc6c5cccn6	7.5	7.2	7.5
161	Clc1ccc(N2CC[NH+](CCCc3nc4ccccc4s3)CC2)cc1	7.4	7.5	7.4
162	Oc1ccc(N2CC[NH+](CCCCOc3ccc4c(NC(=O)CC4)c3)CC2)c5cccnc15	7.4	7.3	7.4
163	Brc1ccccc1Cn2c(c(nn2)C(=O)NCCCCC[NH+]3CCN(CC3)c4ccccc4OC)C	7.4	7.1	7.4
164	Clc1c(Cl)cccc1N2CC[NH+](CCCOc3cc(cc4c3C(C)=CC(O4)=O)C)CC2	7.4	7.1	7.4
165	Fc1ccc(C(=O)C2CC[NH+](CC2)C[C@@H]3CC(=O)c4cn[nH]c4C3)cc1	7.4	6.8	7.4
166	Clc1c(Cl)cccc1N2CC[NH+](CC2)C/C=C/CNC(=O)c3ccc(cc3)-c4c(cccn4)C	7.3	7.9	7.3
167	Clc1c(Cl)cccc1N2CC[NH+](CC2)CCc3cn(nn3)-c4ccn5c(c4)c(cn5)C=O	7.3	7.2	7.3
168	O=C(Nc1cc(OCC[NH+](C)C)c2c(CCO2)c1)c3ccc(-c4ccc(cc4C)-c5noc(n5)C)cc3	7.3	7.1	7.3
169	[O-]C(=O)CC1C[NH+]([C@@H]2CC[C@@]3(C2)c4ccccc4Cc5ccccc5C3)C1	7.3	7.2	7.3
170	Clc1cccc(N2CC[NH2+]CC2)c1	7.3	6.9	7.0
171	Fc1ccccc1N2CC[NH+](CCCOc3cc(cc4c3C(C)=CC(O4)=O)C)CC2	7.3	7.0	7.3
172	O=S(=O)(N1CCC[C@@H]1CC[NH+]2CCN(CC2)c3cccc4c3ccs4)c5cccc6c5cccn6	7.3	7.3	7.3
173	[NH3+]CC1c2ccccc2Cc3ccccc31	7.2	7.0	7.2
174	FC(F)(F)c1cccc2c1C(=O)N3CC[NH2+]C[C@@H]23	7.2	6.5	6.9
175	Brc1ccccc1Cn2c(CCC)c(nn2)C(=O)NCCCCC[NH+]3CCN(CC3)c4ccccc4OC	7.2	7.7	7.2
176	FC(F)(F)c1c(OC)cc2c(N(CC2)C(=O)Nc3cncc(-c4cnccc4)c3)c1	7.2	6.9	7.2
177	Fc1ccccc1S(=O)(=O)c2cnc(cc2)/C=C/c3ccc(F)cc3OC	7.2	7.9	7.7
178	FC(F)(F)Oc1cccc2c1C(=O)N3CC[NH2+]C[C@@H]23	7.2	6.7	6.8
179	C[NH+]1CCN2c3c(Cc4ccccc4[C@@H]2C1)ccc[nH+]3	7.2	7.1	7.2
180	Clc1ccc(N2CC[NH+](CCCOc3cc(cc4c3C(C)=CC(O4)=O)C)CC2)cc1	7.2	7.4	7.2
181	O=C(NC/C=C/C[NH+]1CCN(CC1)c2ccccc2OC)c3ccc(-c4ccccn4)cc3	7.1	7.2	7.1
182	O=C(NCCCC[NH+]1CCN(CC1)c2cccc(c2)C)[C@@H]3CCc4ccccc4N3	7.1	8.0	7.1
183	[NH3+]CCc1ccc(CCCCCC)cc1	7.1	7.1	7.1
184	F[C@]1(CCC[NH+]([C@H]2CC[C@@]3(C2)c4ccccc4Cc5ccccc5C3)C1)C([O-])=O	7.1	6.5	7.0
185	[O-]C(=O)C1CC[NH+]([C@H]2CC[C@]3(C2)c4ccccc4Cc5ccccc5C3)CC1	7.1	7.1	7.1
186	Clc1ccc(OC)c2c1C(=O)N3CC[NH2+]C[C@@H]23	7.1	6.8	7.1
187	Fc1ccc2c(CCC3(O2)CC[NH+]([C@@H]4Cc5ccccc5CC4)CC3)c1	7.0	7.6	7.3
188	Clc1c(F)ccc(S(=O)(=O)NCCC[NH+]2CCN(CC2)c3c4ccccc4on3)c1	7.0	6.9	7.0
189	O=C(NCCCC[NH+]1CCN(CC1)c2ccccc2OC)c3cccn4c3ccn4	7.0	7.2	7.0
190	[O-]C(=O)[C@@H]1C[NH+]([C@H]2CC[C@]3(C2)c4ccccc4Cc5ccccc5C3)CCO1	7.0	6.7	7.0
191	FC(F)(F)c1c(OC)cc2c(N(CC2)C(=O)Nc3cnc(Oc4cnccc4)cc3)c1	7.0	6.9	7.0
192	O=C(NCCCC[NH+]1CCN(CC1)c2ccccc2)c3ccccn3	7.0	7.0	7.0
193	Fc1ccc(C(=O)C2CC[NH+](CC2)C[C@H]3CC(=O)c4cnc(SC)nc4C3)cc1	7.0	7.5	7.0
194	Brc1cccc(CCC[NH+](CCc2ccc3c(OCO3)c2)CCc4ccc(OC)c(OC)c4)c1	7.0	6.8	7.0
195	O=S(=O)(N1CCC[C@H]1CC[NH+]2CCN(CC2)c3cccc4c3OCCO4)c5cccc6c5cccn6	7.0	7.0	7.0
196	FC(F)(F)c1cccc2c1C(=O)N3CC[NH2+]C[C@@H]23	7.0	6.5	7.0
197	O=C1C=C(c2c(O1)cc(cc2OCCCC[NH+]3CCN(CC3)c4ccccc4C#N)C)C	7.0	7.1	7.0
198	FC(F)(F)c1cccc2c1C(=O)N3CC[NH2+][C@H]([C@H]23)C	6.9	6.4	6.8
199	Clc1ccc2c(c3C(=O)N(Cc3n2C)c4ccc(OC)c(OCC[NH+]5CCC(CC5)C)c4)c1	6.9	6.9	6.9
200	Fc1ccc2c(onc2C3CC[NH+](C[C@H]4CC(=O)c5cnc(nc5C4)-c6ccccc6)CC3)c1	6.9	7.3	6.9
201	Clc1c(cc2CCN(c2c1)C(=O)Nc3cnc(Oc4cccnc4C)c(c3)C)C	6.9	6.7	6.9
202	N#C[C@@](CCC[NH+](CCc1ccc(OC)c(OC)c1)C)(C(C)C)c2ccc(OC)c(OC)c2	6.9	6.4	6.9
203	C[NH+](CCO[C@@H](c1ccccc1)c2ccccc2C)C	6.9	6.8	6.9

204	Ic1c(F)cc2c(N(CC2)C[C@@H]([NH3+])C)c1	6.9	6.8	6.9
205	O=C(NCCCC[NH+]1CCN(CC1)c2ccccc2OC)c3c(n(nn3)Cc4ccc(OC)cc4)C	6.9	6.5	6.9
206	O=C(NCCCCC[NH+]1CCN(CC1)c2ccccc2OC)c3c(n(nn3)Cc4ccccc4)C	6.9	6.5	6.8
207	Fc1ccccc1S(=O)(=O)c2cnc(cc2)/C=C\c3cccc(F)c3	6.8	6.6	6.8
208	Clc1ccc2c(c3C(=O)N(Cc3n2C)c4ccc(OC)c(OCC[NH+]5CCCCC5)c4)c1	6.8	6.9	6.8
209	[O-]C(=O)CC[NH2+][C@H]1CC[C@]2(C1)c3ccccc3Cc4ccccc4C2	6.8	7.1	6.8
210	FC(F)(F)c1cc2c(CCN2C(=O)Nc3cnc(Oc4cccnc4C)cc3)cc1C	6.8	6.5	6.8
211	Clc1ccc(N2CC[NH+](CCC2)CCCCc3nc4ccccc4s3)cc1	6.8	7.4	7.2
212	Brc1cc2c(CCN2C(=O)Nc3cnc(Oc4cccnc4C)cc3)cc1C	6.8	6.7	6.8
213	Clc1c(F)cc2c(N(CC2)C[C@@H]([NH3+])C)c1	6.8	6.5	6.8
214	Clc1cccc2c1C(=O)N[C@@H]3CC[NH2+]CCN23	6.8	6.2	6.8
215	[NH3+][C@H](CN1c2cc(SC)ccc2CC1)C	6.8	6.6	6.8
216	[NH3+][C@H](CN1c2cc(SCC)ccc2CC1)C	6.7	6.8	6.7
217	O=C1C=C(c2c(O1)cc(cc2OCCCC[NH+]3CCN(CC3)c4cccc(OC)c4)C)C	6.7	6.8	6.7
218	O=Cc1cc2cc(OCCCC[NH+]3CCN(CC3)c4ccccc4OC)ccn2n1	6.7	7.2	6.7
219	Fc1cccc(CCC[NH+](CCc2ccc3c(OCO3)c2)CCc4ccc(OC)c(OC)c4)c1	6.7	7.2	6.7
220	COc1ccc(CC[NH+](CCCc2cccc(OC)c2)CCc3ccc4c(OCO4)c3)cc1OC	6.7	7.1	6.7
221	[NH3+]C[C@H](c1ccc(CCCCCC)cc1)c2ccccc2	6.7	7.1	6.7
222	FC(F)(F)c1cc2c(CCN2C(=O)Nc3cc(cc(CC)c3)-c4cnccc4)cc1OC	6.7	6.6	6.7
223	C[NH+]1CCc2cc(OC)c(OC)c3-c4cc(OC)c(OCc5ccccc5)cc4C[C@H]1c32	6.7	6.5	6.7
224	O=C(NCCCCC[NH+]1CCN(CC1)c2ccccc2OC)c3cc4ccccn4n3	6.7	6.8	6.7
225	Clc1ccc(OCc2csc(n2)C)c(OC3C[NH2+]C3)c1	6.7	6.7	6.7
226	[NH3+][C@H](Cn1ccc2ccc3ccncc3c21)C	6.6	6.8	6.8
227	Clc1ccc2c(n(c3CN(C(=O)c23)c4ccc(OC)c(OCC[NH+]5CCC(CC5)C)c4)C)c1	6.6	6.2	6.6
228	Fc1cccc2c1n(c3CN(c4ccc(OC)c(OCC[NH+]5CCC(CC5)C)c4)C(=O)c23)C	6.6	7.2	6.6
229	FC(F)(F)c1c(SC)cc2c(N(CC2)C(=O)Nc3cnc(Oc4cccnc4C)cc3)c1	6.6	6.4	6.6
230	FC(F)(F)c1c(OC)cc2c(N(CC2)C(=O)Nc3cccc(-c4cnccc4C)c3)c1	6.6	6.3	6.6
231	Clc1c(Cl)cc2c(CCN2C(=O)Nc3cnc(Oc4cccnc4C)cc3)c1	6.6	6.2	6.6
232	C[NH+]1CCc2c(C1)c3cccc4-c5ccccc5CCn2c43	6.6	6.4	6.6
233	O=C1c2ccccc2N3CC[NH2+]CC[C@H]3N1	6.6	6.6	6.6
234	Clc1ccc2c([NH+]=C(NC3CC[NH+](CC3)Cc4ccccc4)c5cccnc5O2)c1	6.6	6.9	6.6
235	Clc1ccc2c(n(c3CN(C(=O)c23)c4ccc(OC)c(OCC[NH+]5CCCCC5)c4)C)c1	6.5	6.4	6.5
236	Clc1ccc2c(N(CC2)C(=O)Nc3cnc(Oc4cccnc4C)cc3)c1	6.5	6.2	6.5
237	Fc1ccc(SCCC[NH+]2Cc3ccccc3CC2)cc1	6.5	7.2	7.2
238	Clc1cccc2c1C(=O)N[C@H]3C[NH2+]CCN23	6.5	6.3	6.5
239	Brc1ccc2c(N(CC2)C[C@@H]([NH3+])C)c1	6.5	6.8	6.7
240	FC(F)(F)c1cccc2c1C(=O)N3[C@H](C[NH2+]C[C@@H]23)C	6.4	6.7	6.6
241	Clc1ccc(CCC[NH+](CCc2ccc3c(OCO3)c2)CCc4ccc(OC)c(OC)c4)cc1	6.4	6.4	6.4
242	Oc1c(c(CC2=[NH+]CCN2)c(cc1C(C)(C)C)C)C	6.4	7.0	6.9
243	Clc1c(Cl)ccc2c1C(=O)N3CC[NH2+]C[C@@H]23	6.4	6.3	6.4
244	Clc1c(Cl)ccc([C@@H]2C(=O)N(CC2)c3ccc(OC)c(OCC[NH+]4CCC(CC4)(C)C)c3)c1	6.4	6.5	6.4
245	Fc1ccc(C=2C(=O)N(c3ccc(OC)c(OCC[NH+]4CCCCC4)c3)CC2)cc1	6.4	6.2	6.4
246	FC(F)(F)c1c(SC)cc2c(N(CC2)C(=O)Nc3cnccc3)c1	6.4	6.3	6.4
247	[NH2+](CC1c2ccccc2Cc3ccccc31)CCc4ccccc4	6.4	7.1	7.1
248	[NH3+][C@H](CN1c2c(CC1)ccc3c2CCCO3)C	6.4	6.4	6.4
249	Brc1ccc(COc2c(OC)cc-3c(C[C@H]4c5c3c(OC)c(OC)cc5CC[NH+]4C)c2)cc1	6.4	6.5	6.4
250	O=C(NCCCC[NH+]1CCN(CC1)c2ccccc2OC)c3cc4ccccn4n3	6.4	6.8	6.4
251	Clc1cccc(Cn2c3c(nc2CC)c(N4CC[NH2+]CC4)ccn3)c1	6.4	6.6	6.4
252	Clc1cccc2c1[C@@H](NC(=[NH+]2)NCC(F)F)C	6.3	6.4	6.3

253	Clc1c(Cl)ccc(N2CC[C@@](C2=O)(c3ccc(OC)c(OCC[NH+]4CCCCC4)c3)C)c1	6.3	6.8	6.3
254	FC(F)(F)c1cc2c(CCN2C(=O)Nc3cccc(-c4cccnc4C)c3)cc1OC	6.3	6.2	6.3
255	FC(F)(F)c1cc2c(c3c1OCC3)CCN2C(=O)Nc4cnc(Oc5cccnc5C)cc4	6.3	6.3	6.3
256	Clc1c(F)cc2c(N(CC2)C(=O)Nc3cnc(Oc4cccnc4C)cc3)c1	6.3	6.0	6.3
257	FC(F)(F)c1cc(cc2c1C(=O)N3CC[NH2+]C[C@@H]23)C	6.3	6.4	6.2
258	O=C1c2cc(OC)c(cc2[C@@H]3C[NH2+]CCN13)CC	6.2	6.9	6.7
259	Oc1ccc(N2CC[NH+](CC2)CCCCOc3ccn4c(c3)c(cn4)/C=N/O)c5c1OCCO5	6.2	6.4	6.2
260	FC(F)(F)c1cccc([C@@H](Oc2ccc(cc2OC3C[NH2+]C3)C(F)(F)F)C)c1	6.2	6.5	6.2
261	FC(F)(F)c1cc(cc2c1C(=O)N3CC[NH2+]C[C@@H]23)CCCC	6.2	6.3	6.2
262	Fc1ccc(C(=O)C2CC[NH+](C[C@@H]3CC(=O)c4cnc(nc4C3)-c5ccccc5)CC2)cc1	6.2	6.9	6.6
263	Clc1cccc(N2C(=O)N(CC[NH+]3Cc4ccccc4C3)CC2)c1	6.2	6.8	6.2
264	[O-]C(=O)CC1CC[NH+]([C@@H]2CC[C@@]3(C2)c4ccccc4Cc5ccccc5C3)CC1	6.2	6.5	6.2
265	FC(F)(F)c1c(OC)cc2c(N(CC2)C(=O)Nc3cnc(Oc4cccnc4C)cc3C)c1	6.2	6.3	6.2
266	O=C1C(=CCN1c2ccc(OC)c(OCC[NH+]3CCC(CC3)C)c2)c4ccc(cc4)C	6.2	7.0	6.2
267	[O-]C(=O)C1[C@H]2C[NH+]([C@H]3CC[C@@]4(C3)c5ccccc5Cc6ccccc6C4)C[C@@H]12	6.2	6.3	6.2
268	C[NH+]1CCc2c(c3cccc4Cc5ccccc5Cn2c43)C1	6.1	6.8	6.5
269	Clc1c(Cl)cc([C@H]2c3ccccc3[C@@H]([NH2+]C)CC2)cc1	6.1	6.1	6.1
270	Clc1cccc(C=2C(=O)N(c3ccc(OC)c(OCC[NH+]4CCC(CC4)C)c3)CC2)c1	6.1	6.1	6.1
271	Clc1cc2c(CCN2C(=O)Nc3cnc(Oc4cccnc4C(C)C)cc3)cc1C	6.1	6.5	6.1
272	Fc1cc2c(CCN2C(=O)Nc3cnc(Oc4cccnc4C)cc3)cc1C	6.1	6.5	6.3
273	FC(F)(F)c1cccc(C=2C(=O)N(c3ccc(OC)c(OCC[NH+]4CCCCC4)c3)CC2)c1	6.1	6.6	6.1
274	FC(F)(F)c1cc(cc2c1C(=O)N3CC[NH2+]C[C@@H]23)C=C	6.1	6.3	6.1
275	COc1ccc(OC)cc1CCC[NH+](CCc2ccc3c(OCO3)c2)CCc4ccc(OC)c(OC)c4	6.0	6.5	6.0
276	O=C1c2c3cc(OC)ccc3n(c2CN1c4ccc(OC)c(OCC[NH+]5CCCCC5)c4)C	6.0	6.1	6.0
277	FC(F)(F)c1c(OC)cc2c(N(CC2)C(=O)Nc3ccc(C(C)(C)C)c(-c4cnccc4)c3)c1	6.0	5.9	6.0
278	Clc1c(Cl)ccc(N2C(=O)N(CC2)c3cnc(OCC[NH+]4CCCCC4)cc3)c1	6.0	6.2	6.0
279	Clc1ccc(N2CC[NH+](CCC2)CCCC(=O)c3nc4ccccc4s3)cc1	6.0	6.3	6.4
280	FC(F)(F)c1cc(CCC)cc2c1C(=O)N3CC[NH2+]C[C@@H]23	6.0	6.1	6.0
281	Clc1cc(Cl)ccc1CO/N=C(/c2c(Cl)cc(Cl)cc2)Cn3cncc3	5.9	6.2	5.9
282	Clc1ccc(NC(=O)N2CCc3cc(OC)c(cc32)C(F)(F)F)cc1-c4cnccc4	5.9	6.4	6.3
283	[NH3+]C[C@H]1c2ccccc2Cc3ccc(OC)cc31	5.9	6.1	5.9
284	FC(F)(F)c1cc(OC)cc2c1C(=O)N3CC[NH2+]C[C@@H]23	5.9	6.1	5.9
285	Oc1ccc(N2CC[NH+](CCC2)CCCCNC(=O)c3ccc(OCCCc4cn(nn4)CCCC)c(OC)c3)c5C=CC(=O)Nc15	5.8	6.0	5.8
286	FC(F)(F)c1ccc(C=2C(=O)N(c3ccc(OC)c(OCC[NH+]4CCC(CC4)C)c3)CC2)cc1	5.8	5.9	5.8
287	O=C(c1c(cc2c(c1OCCCC[NH+]3CCN(CC3)c4cc[nH+]cc4)C(=CC(O2)=O)C)C)C	5.8	5.9	5.8
288	O=C1c2ccccc2N3CC[NH2+]C[C@@H]3N1	5.7	6.3	6.0
289	Fc1cccc(N2C(=O)N(CC2)c3ccc(OC)c(OCC[NH+]4CCCCC4)c3)c1	5.7	6.2	5.7
290	Clc1ccc(N2CC[NH+](CCCC(=O)c3nc4ccccc4s3)CC2)cc1	5.7	6.4	5.7
291	COc1c(OC)cc(CCC[NH+](Cc2ccc3c(OCO3)c2)CCc4ccc(OC)c(OC)c4)cc1OC	5.7	6.2	5.7
292	O=C1C=C(c2c(O1)cc(cc2OCCCC[NH+]3CCN(CC3)c4cnccn4)C)C	5.6	5.6	5.6
293	Clc1ccc(c2c1C(=O)N3CC[NH2+]C[C@@H]23)CC	5.6	5.9	5.7
294	FC(F)(F)c1cc(cc2c1C(=O)N3CC[NH2+]C[C@@H]23)C(C)C	5.6	6.2	5.7
295	O=C(c1c(cc2c(c1OCCC[NH+]3CCN(CC3)c4cnccn4)C(C)=CC(O2)=O)C)C	5.5	6.0	5.5
296	O=C1C2(NC(=O)N1)CC[NH+](CC2)CCCCC(=O)c3c(OC)cc(OC)c(NS(=O)(=O)c4ccccc4)c3	5.5	5.8	5.5
297	FC(F)(F)c1cccc2c1C(=O)N3CC[NH+](C[C@@H]23)C	5.4	6.2	6.1
298	O=C(c1c(cc2c(c1OCCCC[NH+]3CCOCC3)C(=CC(O2)=O)C)C)C	5.4	5.0	5.3

299	[NH3+][C@H](Cc1c(OC)ccc(OC)c1)C	5.3	6.0	5.4
300	Oc1ccc(N2CCC[NH+](CC2)C)c3c1NC(=O)CO3	5.2	5.9	5.7

Table S2. SMILES, experimental and predicted (field and SVM) pK_i values of the molecules in the test set

n	smiles	${}_{exp}pK_i$	${}_{field}pK_i$	${}_{SVM}pK_i$
1	Clc1ccc(C(=O)N[C@H]2CCc3ccc(CC[NH+]4CCN(CC4)c5c6ccccc6sn5)cc32)cc1	10.0	9.2	8.8
2	O=S(=O)(NC1CCC(CC1)CC[NH+]2CCN(CC2)c3c4ccccc4sn3)c5cccs5	9.5	8.9	8.6
3	Clc1ccc2c(snc2N3CC[NH+](CC4CCC(CC4)CNC(=O)c5ccco5)CC3)c1	9.5	9.3	9.0
4	O=C(N(C(C)C)C(C)C)NC1CCC(CC[NH+]2CCN(CC2)c3c4ccccc4sn3)CC1	9.4	8.7	8.8
5	O=C(N[C@H]1CCc2ccc(CC[NH+]3CCN(CC3)c4c5ccccc5sn4)cc21)c6ccco6	9.3	9.5	9.5
6	Clc1ccc(S(=O)(=O)C2(F)CC[NH+](CC2)CCc3ccc(F)cc3F)cc1	9.2	8.2	8.3
7	Clc1cccc(N2CC[NH+](CCCCOc3ccc4c(NC(=O)CC4)n3)CC2)c1C(F)(F)F	8.9	8.4	8.4
8	Fc1ccc2c(onc2C3CC[NH+](CCCNS(=O)(=O)c4csc5ccccc54)CC3)c1	8.8	8.7	8.8
9	Fc1ccc(C(=O)CCC[NH+]2CC[C@H]3c4cccc5c4N([C@H]3CC2)CCN5C)cc1	8.8	8.7	8.6
10	Fc1ccc2c(onc2C3CC[NH+](CC3)CCCCNS(=O)(=O)c4cccc(c4)C)c1	8.7	9.1	9.0
11	[NH3+][C@@H](Cc1c(OC)cc(CCCCCC)c(OC)c1)C	8.6	7.6	7.6
12	Fc1cc(F)ccc1CC[NH+]2CCC3(CC2)Cc4ccccc4CC3	8.5	8.3	8.3
13	Fc1ccc(C(=O)CCC[NH+]2CC[C@H]3[C@H](C2)c4cc(OC)cc5c4N3CCN5C)cc1	8.5	7.8	8.1
14	O=C(CCC[NH+]1Cc2ccccc2CC1)c3ccccc3	8.4	8.0	7.9
15	Fc1ccc(C(=O)CCC[NH+]2CC[C@H]3[C@H](C2)c4cccc5c4N3CCN5C)cc1	8.3	8.1	8.1
16	O=C(Nc1ccc(OC)c(NCC[NH+](C)C)c1)c2ccc(-c3ccc(cc3C)-c4noc(n4)C)cc2	8.3	7.7	8.1
17	Clc1c(Cl)cccc1N2CC[NH+](CC2)CCCc3cn(nn3)-c4ccn5c(c4)ccn5	8.3	8.3	8.3
18	O=S(=O)(NCCCC[NH+]1CCN(CC1)c2c3ccccc3sn2)c4cccs4	8.3	9.0	8.2
19	Fc1ccc(C(=O)CCC[NH+]2CC[C@H]3[C@H](C2)c4cccc5c4N3[C@@H](CN5C)C)cc1	8.2	8.6	8.4
20	Clc1c(Cl)cccc1N2CC[NH+](CC2)CCCC(=O)Nc3ccn4c(c(cn4)C=O)c3	8.2	7.7	7.8
21	Fc1ccc2c(onc2C3CC[NH+](C[C@H]4CC(=O)c5cc(oc5C4)-c6ccccc6)CC3)c1	8.2	8.7	8.6
22	Clc1c(Cl)cccc1N2CC[NH+](CCCCNC(=O)c3c(n(nn3)Cc4ccccc4)C)CC2	8.1	8.1	8.0
23	Fc1ccccc1S(=O)(=O)c2cnc(/C=C/c3ccc(F)cc3C)cc2	8.1	7.4	7.5
24	Clc1c(F)ccc(S(=O)(=O)NCCCC[NH+]2CCC(CC2)c3c4ccc(F)cc4on3)c1	8.1	7.0	7.1
25	Clc1ccc2c(sc(S(=O)(=O)NCCC[NH+]3CCN(CC3)c4c5ccccc5sn4)c2)c1	8.0	8.0	8.2
26	[NH3+][C@@H](Cc1c(OC)cc(CCCc2ccccc2)c(OC)c1)C	8.0	7.9	8.0
27	Fc1cccc(N2CC[NH+](CC2)CCCCNC(=O)c3cc4ccccc4cn3)c1	8.0	7.5	7.8
28	O=S(=O)(NCCC[NH+]1CCN(CC1)c2c3ccccc3sn2)c4cnc5ccccn54	7.9	7.3	7.4
29	Clc1ccc2c(ccc(S(=O)(=O)NCCC[NH+]3CCN(CC3)c4c5ccccc5sn4)c2)c1	7.9	7.7	7.7
30	Fc1ccc(C(=O)CCC[NH+]2Cc3ccccc3CC2)cc1	7.9	7.7	7.7
31	O=C(Nc1ccc(OC)c(N2CC[NH+](CC2)C)c1)c3ccc(-c4ccc(cc4C)-c5noc(n5)C)cc3	7.8	8.0	7.7
32	Fc1ccc(C(=O)C2CC[NH+](C[C@@H]3CC(=O)c4ccc(OC)cc4C3)CC2)cc1	7.8	8.2	8.6
33	O=C(NCCCC[NH+]1CCN(CC1)c2ccc(O)cc2)c3cc4ccccc4cn3	7.8	8.3	8.2
34	Fc1ccc(C(=O)CCC[NH+]2CC[C@H]3c4cccc5c4N([C@H]3CC2)CCN5C)cc1	7.8	7.8	7.8
35	O=S(=O)(N1CCC[C@@H]1CC[NH+]2CCN(CC2)c3c4ccccc4sn3)c5cccc6cnccc65	7.7	7.5	7.6
36	Fc1ccc2c(onc2C3CC[NH+](CC3)C[C@H]4CC(=O)c5c(oc(n5)C)C4)c1	7.7	8.1	7.9
37	Ic1c(OC)cc(C[C@@H]([NH3+])C)c(OC)c1	7.6	6.8	6.7
38	Fc1ccc2c(onc2C3CC[NH+](C[C@@H]4CC(=O)c5ccc(cc5C4)C(=O)C)CC3)c1	7.5	8.0	7.6
39	Fc1ccc2c([nH]cc2C3=CC[NH+](CC3)C[C@@H]4CC(=O)c5c(nc(s5)N6CCCC6)C4)c1	7.4	7.8	7.9
40	Brc1ccccc1Cn2c(CCC)c(nn2)C(=O)NCCCC[NH+]3CCN(CC3)c4ccccc4OC	7.4	7.5	7.4
41	O=C1CCc2ccc(OCCCC[NH+]3CCN(CC3)c4ccccc4C)nc2N1	7.3	7.7	7.7
42	Fc1cc2c(N(CC2)C[C@@H]([NH3+])C)cc1SCC	7.2	6.9	7.1

43	Clc1ccc2c(sc(S(=O)(=O)NCCC[NH+]3CCN(CC3)c4c5ccccc5on4)c2)c1	7.2	7.3	7.2
44	FC(F)(F)c1cc2c(CCN2C(=O)Nc3cccc(c3)-c4cnccc4)cc1SC	7.1	6.6	7.0
45	Clc1ccc2c([NH+]=C(N3CCN(C4CCCCC4)CC3)c5cccnc5O2)c1	7.0	6.9	7.0
46	Clc1ccc(C2(O)CC[NH+](CC2)CCCSc3ccc(F)cc3)cc1	7.0	8.0	8.0
47	Clc1cc2c(CCN2C(=O)Nc3cnc(Oc4c(Cl)nccc4)cc3)cc1C	7.0	6.4	6.5
48	Oc1ccc2c(Cc3ccccc3[C@H]2C[NH3+])c1	7.0	7.1	7.1
49	FC(F)(F)c1c(OC)cc2c(N(CC2)C(=O)Nc3cc(F)cc(-c4cnccc4)c3)c1	6.9	6.9	7.2
50	Clc1ccc2c([NH+]=C(N3CCNCC3)c4cccnc4O2)c1	6.9	6.2	6.4
51	Fc1cc(F)ccc1CC[NH+]2CCC3(CC2)CC(=O)c4cc(ccc4O3)C#N	6.8	7.6	7.5
52	Clc1cc2c(CCN2C(=O)Nc3cnc(Oc4cccnc4C)cc3)cc1C	6.8	6.4	6.4
53	O=C(NCCCC[NH+]1CCN(CC1)c2cccc(OC)c2)c3ccccn3	6.8	7.5	7.6
54	Clc1cc2c(CCN2C(=O)Nc3cnc(Oc4cccnc4CC)cc3)cc1C	6.7	6.7	6.5
55	FC(F)(F)c1cc2c(CCN2C(=O)Nc3cccc(c3)-c4cnccc4)cc1OC	6.7	7.0	7.2
56	OCc1cc2cc(OCCCC[NH+]3CCN(CC3)c4ccccc4OC)ccn2n1	6.6	7.1	7.5
57	Clc1cc(F)ccc1[C@H]2C[C@@H]2C[NH3+]	6.6	6.4	6.6
58	Clc1ccc(C2(O)CC[NH+](CC2)CCCCc3ccc(F)cc3)cc1	6.5	7.5	7.3
59	Fc1ccc(OCCC[NH+]2Cc3ccccc3CC2)cc1	6.5	7.2	7.4
60	O=C1c2ccc(cc2N3CC[NH2+]CC[C@H]3N1)C	6.4	6.4	6.9
61	FC1(CC[NH+]([C@@H]2CC[C@@]3(C2)c4ccccc4Oc5ccccc5C3)CC1)C([O-])=O	6.4	6.9	7.0
62	FC(F)(F)c1cc(cc2c1C(=O)N3CC[NH2+]C[C@@H]23)CC	6.4	5.8	5.7
63	O=S(=O)(n1cc2C=C(Oc3cccc1c32)C[NH+](C)C)c4cccc5ccccc54	6.3	5.7	5.6
64	Clc1ccc(N2CC[NH+](CCCCC(=O)c3nc4ccccc4s3)CC2)cc1	6.3	6.9	6.9
65	Clc1c(Cl)ccc(C2=CCN(c3ccc(OC)c(OCC[NH+]4CCCCC4)c3)C2=O)c1	6.2	5.8	5.6
66	FC(F)(F)c1c(cc2CCN(c2c1)C(=O)Nc3cnc(OCc4ccccn4)cc3)C	6.2	7.1	7.1
67	Brc1ccccc1[C@@H]2C[C@@H]2C[NH3+]	6.2	6.5	6.4
68	Clc1ccc(S(=O)(=O)Nc2c(OC)cc(OC)c(C(=O)CCCC[NH+]3CCC4(CC3)C(=O)NC(=O) N4)c2)cc1	6.1	5.9	6.1
69	O=C1C=C(Oc2c1cccc2-c3ccccc3)N4CCOCC4	6.1	6.5	7.0
70	C[NH+]1CCc2cc(OC)c(OC)c3-c4cc(OC)c(OC)cc4C[C@@H]1c32	6.0	7.4	7.1
71	[NH3+][C@H](Cn1ccc2ccc3cnccc3c21)C	6.0	6.7	6.7
72	Clc1ccc(c2C(=O)N3CC[NH2+]C[C@@H]3c12)C(F)(F)F	5.8	6.1	6.4
73	Clc1c(OC)cc2C(=O)N3CC[NH2+]C[C@H]3c2c1	5.8	6.9	7.0
74	FC(F)(F)c1cc(OCC)cc2c1C(=O)N3CC[NH2+]C[C@@H]23	5.4	5.8	5.8
75	O=C1C=C(c2c(O1)cc(cc2OCCC[NH+]3CCN(CC3)c4cnccn4)C)C	5.3	5.5	5.7

Table S3. Name of NPS molecules, experimental pEC_{50} and predicted (field and SVM) pK_i values.

molecules	pEC_{50} measured[1]	pK_i from Field model	pK_i from SVM model
25C-NBOH	9.05	8.0	7.9
25E-NBOMe	9.03	8.2	8.3
25D-NBOMe	8.90	8.5	8.5
25I-NBOMe	8.89	7.8	7.9
25I-NBOH	8.82	8.1	8.1
25C-NBOMe	8.74	8.8	8.7
25B-NBOMe	8.64	8.1	8.0
BROMODRAGONFLY	8.53	7.8	8.0
DOB	8.41	7.3	7.5
DOI	8.35	7.5	7.5
2C-T-7	8.24	8.0	7.9

2C-I	8.23	7.2	7.3
2C-T-2	8.19	7.3	7.5
2C-E	8.16	7.2	7.4
DOET	8.11	7.2	7.4
DOC	8.09	7.1	7.3
2C-B-FLY	8.09	7.1	7.3
2C-B	8.04	7.4	7.3
25H-NBOH	7.89	7.5	7.4
25H-NBOMe	7.89	7.7	8.0
2C-C	7.73	7.1	7.0
2C-D	7.36	7.6	7.2
DOM	7.36	7.3	7.3
25H-NBMD	6.81	7.2	7.1
25H-NBF	6.41	6.6	6.4
β-k-2CB	6.04	6.5	6.7
2C-H	5.62	6.1	5.9
DOH	5.55	6.5	6.5
N-Me-2C-H	5.35	6.5	6.5

Table S4. Calculated $_{field}pK_i$, $_{SVM}pK_i$ and $_{mean}pK_i$ for the natural products screening

n	name	smiles	$_{field}pK_i$	$_{SVM}pK_i$	$_{mean}pK_i$
1	ZINC08856435	Oc1cc(OC/C=C/c2ccccc2)ccc1-c3c(c([nH]n3)C)-c4ccc(OC)cc4	9.1	9.2	9.15
2	ZINC00006368	Cc1ccc2c(c1O)-c1cccc3c1[C@@H](C2)N(C)CC3	8.9	9.0	8.95
3	ZINC11867629	Fc1c(F)c(COc2ccc3c(c(c(o3)-c4ccccc4)C(OCC)=O)c2)c(F)c(F)c1N5CC[NH2+]CC5	9.0	8.9	8.95
4	ZINC72116881	COc1c(OCc2ccccc2)cc2c3c1-c1cc4c(cc1C[C@@H]3N(C)CC2)OCO4	8.7	9.0	8.90
5	ZINC00607997	COc1cc2c(cc1OC)CN1CCc3cc(OC)c(OC)cc3[C@@H]1C2	9.0	8.8	8.90
6	ZINC68576530	Clc1ccc(Cl)nc1C(OCC[N+](CCCCCCCCCCCC)(C)C)=O	8.7	8.8	8.75
7	ZINC12660071	O=S1(=O)CC[C@@](NC(=O)CCCCCC[C@@H]2C(=O)CC[C@H]2/C=C/[C@@H](O)CCCCC)(C1)C	9.0	8.2	8.60
8	ZINC13690010	O=C(OC)c1c(NC(=O)c2cc3cccc(OC)c3o2)c4ccc(OC)cc4[nH]1	8.9	8.2	8.55
9	ZINC05219478	Oc1cc(OCc2ccccc2)ccc1-c3c(c([nH]n3)C)-c4ccc(OC)cc4	8.7	8.4	8.55
10	ZINC09187792	Clc1ccccc1COc2ccc(-c3c(-c4ccc(OC)c(OC)c4)c([nH]n3)C)c(O)c2	8.6	8.5	8.55
11	12190	Fc1ccccc1C[NH+](C[C@@H]2C=C([C@@H](C[C@H]2C(C)C)Cc3nc4cccc(c4[nH]3)C)C)Cc5ccccc5F	8.9	8.1	8.50
12	ZINC09410825	FC(F)(F)c1c(c(n[nH]1)-c2ccc(OCc3ccc(cc3)C)cc2O)-c4ccc(OC)c(OC)c4	8.5	8.5	8.50
13	MolPort-006-334-884	Clc1ccccc1N2C(=O)C[C@@H](N3CCc4c([nH]c5ccc(OC)cc54)C3)C2=O	8.5	8.5	8.50
14	ZINC39354033	Oc1cc(OCC[NH+]2CCN(CC2)c3ccccc3)ccc1-c4c(-c5ccc(OC)cc5)c(n[nH]4)C	8.5	8.4	8.45
15	ZINC20591088	Oc1cc(OCCN2CC[NH+](CC2)Cc3ccccc3)ccc1-c4c(-c5ccc(OC)cc5)c(n[nH]4)C	8.5	8.4	8.45
16	ZINC09130703	Clc1cccc(F)c1COc2ccc(-c3c(c([nH]n3)C)-c4ccc(OC)cc4)c(O)c2	8.4	8.5	8.45
17	ZINC05343552	Oc1cc(OCC=C(C)C)ccc1-c2c(c([nH]n2)C)-c3ccc(OC)cc3	8.4	8.4	8.40
18	ZINC16036365	O[C@H](CCCCC)/C=C/[C@H]1CCC(=O)[C@H]1CCCCCCC(OCC[NH+]2CCCC[C@H]2c3cnccc3)=O	8.3	8.5	8.40
19	ZINC09187797	Clc1cccc(Cl)c1COc2ccc(-c3c(-c4ccc(OC)c(OC)c4)c([nH]n3)C)c(O)c2	8.4	8.3	8.35
20	157207-88-0	N1(CC[C@H]2c3c(CCN2)c4ccccc4[nH]3)CCCCC=CCCCC1	8.4	8.3	8.35
21	ZINC09418544	Oc1c(-c2c(c([nH]n2)C)-c3ccc(OC)cc3)ccc(OCC[NH+]4CCC(CC4)C(=O)N)c1C	8.3	8.4	8.35
22	ZINC09130113	Brc1ccc(COc2ccc(-c3c(c([nH]n3)C)-c4ccc(OC)cc4)c(O)c2)cc1	8.3	8.4	8.35
23	MolPort-002-530-410	OC1=C([C@H]2c3c(CCN2)c4ccccc4[nH]3)C(=O)N(Cc5ccc(OC)cc5)C(=O)N1	8.5	8.2	8.35

24	SN00009552	O=C(c1c(OC)cc(OC)cc1OC)/C=C\c2ccc(OCc3ccccc3)c(OC)c2	8.5	8.2	8.35
25	ZINC09130734	Oc1cc(OCc2ccc(cc2)C=C)ccc1-c3c(-c4ccc(OC)c(OC)c4)c([nH]n3)C	8.4	8.2	8.30
26	ZINC09409677	Oc1cc(OCc2ccccc2C)ccc1-c3c(-c4ccc(OC)c(OC)c4)c([nH]n3)C	8.4	8.2	8.30
27	ZINC08791003	O=C(NCCC[NH+](C)C)CCC=1C(Oc2c(C1C)ccc(OC)c2C)=O	8.4	8.2	8.30
28	ZINC09130659	FC(F)(F)c1c(-c2ccc(OC)cc2)c(n[nH]1)-c3ccc(OCc4ccccc4F)cc3O	8.4	8.1	8.25
29	6932	O[C@@H]1C[C@@H]([NH+](C1)Cc2cc3ccccc3o2)c4nc(no4)-c5cccc(c5)C	8.3	8.2	8.25
30	ZINC08972988	Clc1ccc(COc2ccc(-c3c(c([nH]n3)C)-c4ccc(OC)cc4)c(O)c2)cc1	8.3	8.2	8.25
31	SN00149601	Oc1cc(OCc2ccc(cc2)C=C)ccc1-c3c(-c4ccc(OC)c(OC)c4)c(no3)C	8.3	8.2	8.25
32	SN00061787	O=C(Nc1ccc2c(c3ccccc3n2CC)c1)CCC=4C(Oc5cc6c(cc5C4C)c(C)co6)=O	8.3	8.1	8.20
33	ZINC09129618	Clc1ccc(COc2ccc(-c3c(c([nH]n3)C)-c4ccc(OC)cc4)c(O)c2)cc1Cl	8.3	8.1	8.20
34	ZINC08973292	FC(F)(F)c1c(-c2ccc(OC)cc2)c(n[nH]1)-c3ccc(OCC(C)=C)cc3O	8.2	8.2	8.20
35	ZINC09187780	FC(F)(F)c1c(-c2ccc(OC)cc2)c(n[nH]1)-c3ccc(OCc4ccccc4)cc3O	8.2	8.2	8.20
36	ZINC68572101	Clc1ccc(Cc2c(/N=C/c3ccc(cc3)C)n[nH]c2/N=C/c4ccc(cc4)C)cc1	8.2	8.2	8.20
37	MolPort-001-016-686	O=C(Nc1c(OC)cc2c(oc3ccccc32)c1)c4cc(OC)cc(OC)c4	8.1	8.3	8.20
38	ZINC08973452	Fc1ccc(COc2ccc(-c3c(c([nH]n3)C)-c4ccc(OC)cc4)c(O)c2)cc1	8.1	8.3	8.20
39	MolPort-002-535-647	O=C1c2c(OC)c(OC)ccc2-c3c4c(c5cc(O[C@H](C(=O)Nc6c(OC)ccc(OC)c6)C)ccc5n14)ccn3	8.3	8.0	8.15
40	ZINC09410789	FC(F)(F)c1c(c(n[nH]1)-c2ccc(OCc3ccccc3F)cc2O)-c4ccc(OC)c(OC)c4	8.1	8.2	8.15
41	MolPort-027-852-775	Clc1ccc(-c2c([C@H](NC(=O)c3cc(n(n3)C)C)C)c(on2)C(O)=O)cc1	8.1	8.2	8.15
42	ZINC09409653	Brc1ccc(COc2ccc(-c3c(-c4ccc(OC)c(OC)c4)c([nH]n3)C)c(O)c2)cc1	8.1	8.2	8.15
43	MolPort-001-738-042	O=C1CN(C(=O)[C@@H]2Cc3c4ccccc4[nH]c3[C@@H](N12)c5ccccc5OC)CCc6ccc(OC)c(OC)c6	8.1	8.2	8.15
44	SN00148291	Oc1cc(OCc2ccccc2)ccc1-c3c(-c4ccc(OC)c(OC)c4)c(no3)C	7.9	8.3	8.10
45	ZINC20466733	Fc1cccc(C(=O)N[C@@H]2CC[C@@]3(CC[C@H]([C@H](O)[C@@H]3[C@H]2C)[C@H](C(=O)N4CCCC4)C)C)c1	8.1	8.1	8.10
46	SN00147982	Oc1cc(OCc2ccc(cc2)C)ccc1-c3c(-c4ccc(OC)cc4)c(no3)C	8.0	8.2	8.10
47	ZINC04235438	OC=1c2ccccc2N(C(=O)C1C(=O)C[C@H]([NH2+]C=3C(=O)N(N(C3C)C)c4ccccc4)c5ccc(N(C)C)cc5)C	8.2	7.9	8.05
48	ZINC02098147	O=C1C2=C([C@@H](N1CCc3ccc(OC)c(OC)c3)c4ccc(O)cc4)C(=O)c5cc(c(cc5O2)C)C	8.0	8.1	8.05
49	ZINC20411284	O[C@@H](CCCCC)/C=C/[C@@H]1CCC(=O)[C@H]1CCCCCCC(=O)NCC[NH+]2CCOCC2	8.0	8.1	8.05
50	ZINC09410809	FC(F)(F)c1c(c(n[nH]1)-c2ccc(OCc3ccc(F)cc3)cc2O)-c4ccc(OC)c(OC)c4	8.1	7.9	8.00
51	ZINC09421984	O=C1c2ccc(OC(=O)c3cccs3)c(c2O/C1=C/c4cn(c5ccccc45)CC)C	8.1	7.9	8.00
52	ZINC70672992	Clc1ccc(OCC#C[C@@H]2c3c(OC)c4c(OCO4)cc3CC[NH+]2C)c(c1)C=C5C(=O)N(C(=O)N(C5=O)C)C	8.0	8.0	8.00
53	ZINC11866356	Clc1cccc(F)c1COc2ccc(-c3c(-c4ccc(OC)cc4)c(no3)C)c(O)c2	8.0	8.0	8.00
54	ZINC01780045	Clc1cccc(COc2ccc3c(OC(=C(c4ccccc4OC)C3=O)C(F)(F)F)c2)c1	7.9	8.1	8.00
55	ZINC11866343	Fc1ccccc1COc2ccc(-c3c(-c4ccc(OC)c(OC)c4)c(no3)C)c(O)c2	7.8	8.2	8.00
56	ZINC09186350	Clc1ccc(COc2ccc(-c3c(-c4ccc(OC)c(OC)c4)c(no3)C)c(O)c2)cc1	7.8	8.2	8.00
57	MolPort-002-511-872	O=C1C2=C(N(C(=O)N1C)C)N=C3c4ccccc4C(OC(=O)CC)=C3[C@@H]2c5ccc(OC)cc5	8.0	7.9	7.95
58	ZINC02122370	O=C(NCCc1ccc(OC)cc1)c2cc3c4ccccc4[nH]c3c(n2)-c5ccc(OC)c(OC)c5	8.0	7.9	7.95
59	ZINC09409699	Clc1cc(F)ccc1COc2ccc(-c3c(c([nH]n3)C)-c4ccc(OC)cc4)c(O)c2	8.0	7.9	7.95
60	ZINC02429597	Fc1c(F)c(F)c(F)c(COc2ccc3c(c(c(n3C)C)C(OCCOC)=O)c2)c1F	7.9	8.0	7.95
61	MolPort-000-424-288	Brc1cc2c(c(c(o2)C)C(OCCOC)=O)cc1OCc3ccc(cc3)C=C	7.9	8.0	7.95
62	ZINC11866321	Clc1cccc(F)c1COc2ccc(-c3c(-c4ccc(OC)c(OC)c4)c(no3)C)c(O)c2	7.9	8.0	7.95
63	ZINC08972974	Oc1cc(OCC)ccc1-c2c(c([nH]n2)C)-c3ccc(OC)cc3	7.9	8.0	7.95
64	ZINC12898513	O=C(NCC(C[NH+](C)C)(C)C)CC=1C(Oc2cc3c(c(co3)C)cc2C1C)=O	8.1	7.8	7.95
65	147293-04-7	O[C@@H]([C@H]1[C@@H](O1)CCCCCCCC(OC)=O)/C=C\C/C=C/CC	8.1	7.8	7.95
66	SN00031093	Fc1ccc(-c2cc(n(n2)C)[C@H]3C[NH+]4CC[C@H]3C[C@H]4C[NH2+]Cc5ccccn5)cc1	8.1	7.8	7.95
67	ZINC02149559	O=C1c2c(OC)c(OC)ccc2C=NN1CCOc3cccc(OC)c3	7.8	8.1	7.95
68	ZINC03840589	O[C@H]1[C@@H](CC[C@]2(Cc3c(nc(s3)Nc4c(OC)ccc(OC)c4)[C@H]([C@H]12)C)C)[C@H](C)C(=O)N(CC=C)C	7.8	8.1	7.95
69	MolPort-002-518-881	O=S(=O)(N[C@H](CCC)C(Oc1ccc-2c(OC(=O)c3cc(OC)ccc32)c1)=O)c4ccc(cc4)C	7.7	8.2	7.95

70	ZINC05190540	Oc1cc(OCc2ccccc2)ccc1-c3c(Oc4ccc(OC)cc4)c[nH]n3	8.1	7.7	7.90
71	MolPort-009-649-103	O=C(Nc1ccc2c([nH]c(n2)COC)c1)CCCc3c[nH]c4ccccc43	8.0	7.8	7.90
72	ZINC02319891	O=C(OCC)c1c2cc(OCc3c(C)ccc(c3)C)ccc2oc1C(C)(C)C	8.0	7.8	7.90
73	ZINC05572691	Fc1ccccc1COc2ccc(-c3c(-c4ccc(OC)cc4)cnc(n3)N)c(O)c2	7.9	7.9	7.90
74	ZINC08845645	O=C(Nc1c(oc2ccccc21)C(=O)Nc3cccc(c3)C)c4cc(n[nH]4)-c5ccc(cc5O)C	7.6	8.2	7.90
75	ZINC02412441	Clc1cccc(COc2ccc3c(OC(=C(c4ccc(OC)cc4)C3=O)C(F)(F)F)c2)c1	8.0	7.7	7.85
76	ZINC09129491	FC(F)(F)c1c(-c2ccc(OC)cc2)c(n[nH]1)-c3ccc(OCc4ccc(F)cc4)cc3O	7.9	7.8	7.85
77	ZINC01322966	O=C1c2ccc(OC(=O)c3cccc(OC)c3)cc2OC(=C1c4ccc(OC)c(OC)c4)C(OCC)=O	7.9	7.8	7.85
78	ZINC09409930	FC(F)(F)c1c(c(n[nH]1)-c2ccc(OCc3ccccc3)cc2O)-c4ccc(OC)c(OC)c4	7.9	7.8	7.85
79	ZINC08791787	O=C1c2ccccc2[C@@H]3N1[C@@H](C(S3)(C)C)C(=O)N[C@@H](C(C)C)C(=O)NCCCc4ccccc4	7.9	7.8	7.85
80	ZINC48057077	Oc1cccc(OC)c1C(=O)/C=C\c2cccc(OC)c2OC	7.8	7.9	7.85
81	ZINC04267171	OC=1c2ccccc2N(C(=O)C1C(=O)C[C@@H]([NH2+]C=3C(=O)N(N(C3C)C)c4ccccc4)c5ccc6c(OCO6)c5)C	7.8	7.9	7.85
82	ZINC20466528	O[C@H]1[C@@H](CC[C@]2(CC[C@H](NC(=O)C3CCCCC3)[C@@H]([C@@H]12)C)C)[C@H](C(=O)NC4CC4)C	7.8	7.9	7.85
83	ZINC01792699	Fc1c(F)c(F)c(F)c(COc2ccc3C=C(c4ccc5ccccc5n4)C(Oc3c2)=O)c1F	7.8	7.9	7.85
84	ZINC11866287	Oc1cc(OCc2ccccc2C)ccc1-c3c(-c4ccc(OC)c(OC)c4)cnc(n3)N	7.8	7.9	7.85
85	ZINC08970919	Clc1cccc(Cl)c1COc2ccc(-c3c(-c4ccc(OC)c(OC)c4)c(no3)C)c(O)c2	7.7	8.0	7.85
86	SN00149108	Clc1cccc(COc2ccc(-c3c(-c4ccc(OC)cc4)c(no3)C)c(O)c2)c1	7.7	8.0	7.85
87	ZINC47284531	Oc1c(c(OCC[NH+]2CCN(CC2)c3ccccc3)ccc1-c4c(-c5ccc(OC)cc5)c(n[nH]4)C)C	7.6	8.1	7.85
88	ZINC08996172	Clc1cccc(COc2ccc(-c3c(Oc4ccc(OC)cc4)c[nH]n3)c(O)c2)c1	7.7	7.9	7.80
89	ZINC03983996	O[C@@]1(CC[C@H]2[C@H]3CC[C@@H]4CC(=O)CC[C@]4([C@@H]3CC[C@@]12C)C)CSc5nccn5C	7.7	7.9	7.80
90	ZINC12900696	O=C1C(=C(c2c(OC)cc3c(CCC(O3)(C)C)c2O1)C)CC(=O)NCCc4c[nH]c5ccc(OC)cc54	8.1	7.5	7.80
91	ZINC04267149	Clc1ccc([C@H]([NH2+]C=2C(=O)N(N(C2C)C)c3ccccc3)CC(=O)C=4C(=O)N(c5ccccc5C4O)C)cc1	8.0	7.6	7.80
92	MolPort-035-700-739	Oc1cc(C2=CC(=O)c3c(O)cc4c([C@H](CC(O4)=O)C=5C(=O)Nc6ccccc6C5)c3O2)ccc1OC	7.8	7.8	7.80
93	ZINC20759596	OC1=C([C@H]2c3c(C[C@H](N2)CC)c4ccccc4[nH]3)C(=O)NC(=O)N1Cc5ccc(OC)cc5	7.8	7.8	7.80
94	ZINC11866347	Oc1cc(OCc2c(C)ccc(c2)C)ccc1-c3c(-c4ccc(OC)c(OC)c4)c(no3)C	7.8	7.8	7.80
95	ZINC13571467	O=C1C(/C(=N/CCc2ccc(OC)c(OC)c2)CC)=C([O-])CC(C1)(C)C	7.8	7.8	7.80
96	ZINC09575415	Brc1ccc(OC)c(/C=C\2C(=O)c3ccc(O)c(C[NH+](CC)CC)c3O2)c1	7.8	7.8	7.80
97	ZINC09410298	Brc1ccc(COc2ccc(-c3c(-c4ccc(OC)c(OC)c4)c(no3)C)c(O)c2)cc1	7.6	8.0	7.80
98	ZINC02158608	O=C1C(=C(c2cc3c(OCN(c4ccccc4C)C3)c(c2O1)C)C)Cc5ccccc5	7.6	8.0	7.80
99	ZINC20760972	O=C(N[C@H]([C@H](CC)C)C(OC)=O)CN1C=Nc2cc(OC)c(OC)cc2C1=O	7.5	8.1	7.80
100	SN00149302	Clc1c(Cl)ccc(COc2ccc(-c3c(-c4ccc(OC)c(OC)c4)c(no3)C)c(O)c2)c1	7.4	8.2	7.80
101	ZINC02107196	O=C1[C@H](c2c3ccccc3n(CCCC)c2)CC(=O)N1c4cccc(OC)c4	7.9	7.6	7.75
102	MolPort-002-517-334	O=C(N[C@@H](Cc1c[nH]c2ccccc21)C(O)=O)COc3ccc4c(OC(=O)C=5CCCCC45)c3	7.9	7.6	7.75
103	ZINC09130740	FC(F)(F)c1c(c(n[nH]1)-c2ccc(OCc3ccccc3C)cc2O)-c4ccc(OC)c(OC)c4	7.8	7.7	7.75
104	MolPort-027-852-794	Fc1ccc(-c2c([C@H](NC(=O)c3cc(n(n3)C)C)C)c(on2)C(O)=O)cc1	7.8	7.7	7.75
105	ZINC19893649	Clc1ccc(Cc2c(n[nH]c2/N=C/c3ccc(F)cc3)/N=C/c4ccc(F)cc4)cc1	7.8	7.7	7.75
106	ZINC01280062	O=C(c1c(cc2C([C@@H](C(c2c1)(C)C)C)(C)C)C)C[C@@H]3c4c(OC)c5c(OCO5)cc4CC[N+]3(C)C	7.7	7.8	7.75
107	ZINC09130747	FC(F)(F)c1c(c(n[nH]1)-c2ccc(OCC(C)=C)cc2O)-c3ccc(OC)c(OC)c3	7.7	7.8	7.75
108	MolPort-046-594-356	Oc1c(OC)cc2CCN([C@H](Cc3ccc(O)c(Oc4c(OC)cc5CCN([C@@H](c5c4)Cc6ccc(OC)cc6)C)c3)c2c1)C	7.7	7.8	7.75
109	ZINC02667596	Clc1c(Cl)cc(COc2ccc3c(OC(=C(c4ccccc4OC)C3=O)C)c2)cc1	7.7	7.8	7.75
110	MolPort-000-826-974	O=C(Nc1c(OC)cc2c(oc3ccccc32)c1)c4cccc(OC)c4	7.6	7.9	7.75
111	ZINC02114178	O=C1[C@@H]2Cc3c4ccccc4[nH]c3[C@@H](N2C(=O)CN1CCCOC)c5ccccc5C	7.6	7.9	7.75
112	SN00382560	Oc1c(OC)cc2CCN([C@H]3Cc4ccc(O)c(c4)-c5c(OC)ccc(C[C@@H]6c7cc(Oc1c23)c(OC)cc7CCN6C)c5)C	7.6	7.9	7.75
113	SN00151289	Clc1cc(F)ccc1COc2ccc(-c3c(-c4ccc(OC)c(OC)c4)c(no3)C)c(O)c2	7.6	7.9	7.75
114	ZINC02147505	O=C(OCC)CCC=1C(Oc2c(c(OCc3cccc(c3)C)ccc2C1C)C)=O	7.5	8.0	7.75
115	SN00147957	Fc1ccc(COc2ccc(-c3c(-c4ccc(OC)cc4)c(no3)C)c(O)c2)cc1	7.5	8.0	7.75

116	ZINC08792424	O=C1c2c(OC)c(OC)ccc2-c3c4c(c5cc(OCc6cccc(c6)C)ccc5n14)ccn3	7.7	7.7	7.70
117	ZINC08789180	O=C1CN(CCCOC)C(=O)C2(c3c([C@H](CN12)c4cccc(OC)c4OCC)c5ccccc5[nH]3)C	7.7	7.7	7.70
118	ZINC70669913	O=C1C=CC=C2[C@@H]3C[C@@H](C[NH+](CC4(Cc5cc(OC)c(OC)cc5OC)C(=O)N(C(=O)N(C4=O)C)C)C3)CN12	7.7	7.7	7.70
119	ZINC20611836	OC1=C([C@@H]2c3c(c4ccccc4[nH]3)CCN2C)C(=O)NC(=O)N1c5ccc(OC)cc5	7.5	7.9	7.70
120	ZINC05452205	Fc1ccccc1COc2ccc(-c3c(-c4ccc(OC)cc4)c(no3)C)c(O)c2	7.5	7.9	7.70
121	ZINC02155319	O=C1C2=C([C@H](N1CCc3ccc(OC)cc3)c4cccc(OC)c4)C(=O)c5ccccc5O2	7.8	7.6	7.70
122	MolPort-002-528-757	O=C(N[C@@H](Cc1c[nH]c2ccc(O)cc21)C(O)=O)[C@@H](Oc3ccc4c(OC(=O)C=C4CC)c3)C	7.8	7.6	7.70
123	MolPort-001-737-511	O=C1CN(CCCN(C)C)C(=O)[C@H]2Cc3c4ccccc4[nH]c3[C@H](N12)c5ccc(C(C)C)cc5	7.8	7.6	7.70
124	20164	Clc1ccc(S(=O)(=O)Cc2nc3c(s2)C[C@@]4(CC[C@@H]([C@H](O)[C@H]4[C@H]3C)[C@@H](C(=O)N5CCOCC5)C)C)cc1	7.6	7.8	7.70
125	MolPort-000-812-834	Clc1cc2c(OC(=O)C(Cc3ccccc3)=C2C)cc1O[C@H](C(=O)N[C@@H](Cc4c[nH]c5ccc(O)cc54)C(O)=O)C	7.6	7.8	7.70
126	ZINC03846610	O=C(C[C@@H](Nc1cnccc1)c2ccc(N(C)C)cc2)C=3C(=O)c4ccccc4N(C3[O-])C	7.6	7.8	7.70
127	8159	Fc1c(F)ccc(C[NH+]2CCC[C@H]2c3nc(no3)-c4ccc(F)cn4)c1	7.6	7.8	7.70
128	ZINC13181151	Oc1c(c(OCC[NH+]2C[C@@H](O[C@@H](C2)C)C)ccc1-c3c(-c4ccc(OC)cc4)c(n[nH]3)C)C	7.9	7.4	7.65
129	ZINC48057072	Oc1cccc(OC)c1C(=O)/C=C/c2cc(OC)c(OC)cc2OC	7.8	7.5	7.65
130	MolPort-002-524-694	O=C(NCc1ccc(OC)cc1)c2cc3c4ccccc4[nH]c3c(n2)-c5cccc(OC)c5	7.8	7.5	7.65
131	MolPort-002-520-663	O=C(N[C@H](Cc1c[nH]c2ccccc21)C(O)=O)CC=3C(Oc4c(c5c(cc4C3C)c(c(o5)C)C)C)=O	7.8	7.5	7.65
132	ZINC31170105	O[C@@](CC/C=C(/CO)C)(/C=C/C/C(C)=C/COc1ccc2C=CC(Oc2c1)=O)C	7.7	7.6	7.65
133	11064	O[C@@H]1[C@H](O)[C@H]2[C@H](SC(=N2)Nc3ccccc3)[C@H](C1)C(=O)NCc4ccccc4OC	7.6	7.7	7.65
134	ZINC09410850	Oc1cc(OCC[NH+]2CCC(CC2)C)ccc1-c3c(-c4ccc(OC)c(OC)c4)c([nH]n3)C	7.6	7.7	7.65
135	ZINC17058595	Oc1cc(OCC[NH+]2C[C@@H](O[C@@H](C2)C)C)ccc1-c3c(-c4ccc(OC)c(OC)c4)c(n[nH]3)C	7.6	7.7	7.65
136	ZINC00083643	O=C(OCC)c1c(oc2ccc(OCc3c(C)ccc(c3)C)cc21)C	7.6	7.7	7.65
137	ZINC08790710	O=C(NCCC[NH+](C)C)CCC=1C(Oc2c(c3c(cc2C1C)c(c(o3)C)C)C)=O	7.6	7.7	7.65
138	ZINC05156892	Oc1cc(OCC)ccc1-c2c(-c3ccc(OC)c(OC)c3)c([nH]n2)C	7.5	7.8	7.65
139	MolPort-021-745-971	O=C(N1CCc2c(C1)c3ccccc3[nH]2)COc4ccc5c(OC(=O)C(=C5C)C)c4	7.5	7.8	7.65
140	ZINC04236981	O[C@@H]1CC[C@@H]2[C@@]([C@@H](O)CC[C@@]2([C@@H]1CC(=O)N(CCCC)CCCC)C)(CO)C	7.4	7.9	7.65
141	ZINC04090087	O=C(NCCCOC(C)C)CC=1C(Oc2cc(c3c(c(oc3c2C1C)C)C)C)=O	7.9	7.3	7.60
142	MolPort-002-530-479	Clc1c(OCC(=O)N[C@@H](Cc2c[nH]c3ccc(O)cc32)C(O)=O)cc4c(C=5CCCC5C(O4)=O)c1	7.7	7.5	7.60
143	ZINC08993461	O=S(Oc1ccc2C(=O)/C(Oc2c1C)=C\c3cn(c4ccccc34)CC)(=O)c5ccc(OC)cc5	7.6	7.6	7.60
144	ZINC09059912	Oc1c(-c2c(c([nH]n2)C)-c3ccc(OC)cc3)ccc(OCC[NH+]4CCCCC4)c1C	7.6	7.6	7.60
145	ZINC20466291	O[C@@H]1[C@H](CC[C@]2(CC[C@@H](NC(=O)c3ccccc3)[C@@H]([C@@H]12)C)C)[C@H](C(=O)N(CCC#N)C)C	7.6	7.6	7.60
146	ZINC01321188	Brc1ccccc1C(Oc2ccc3C(=O)C(c4ccc(OC)c(OC)c4)=C(Oc3c2)C(OCC)=O)=O	7.5	7.7	7.60
147	ZINC79188720	O=C(NCCc1c[nH]c2ccccc21)Cc3cn(c4ccccc34)CCOC	7.5	7.7	7.60
148	ZINC09128884	FC(F)(F)c1c(-c2ccc(OC)cc2)c(on1)-c3ccc(OCc4ccc(F)cc4)cc3O	7.5	7.7	7.60
149	ZINC00627450	O=S(=O)(N[C@H]([C@H](CC)C)C(Oc1ccc2c(OC(=O)C=3CCCC23)c1)=O)c4ccc(cc4)C	7.5	7.7	7.60
150	ZINC02147037	O=S(=O)(N[C@H]([C@@H](CC)C)C(Oc1cc(cc2c1C(=C(C(O2)=O)C)C)C)=O)c3ccc(cc3)C	7.5	7.7	7.60
151	ZINC08789652	O=C1CN(C(=O)[C@@]2(c3c([C@H](CN12)c4ccc(OCCC)c(OC)c4)c5ccccc5[nH]3)C)CCCOC(C)C	7.5	7.7	7.60
152	19285	Clc1ccc(-c2c([C@H](NC(=O)c3cc(n(n3)C)C)C)c(on2)C([O-])=O)cc1	7.4	7.8	7.60
153	MolPort-039-057-114	Oc1ccc2c(oc3c2nc4c(c3-c5cc(OC)c(OC)c(OC)c5)cnn4C)c1C	7.3	7.9	7.60
154	MolPort-002-518-975	O=C1[C@H]2Cc3c4ccccc4[nH]c3C(N2C(=O)N1c5c(OC)cc6c(c7ccccc7o6)c5)(C)C	7.7	7.4	7.55
155	ZINC15675098	Clc1cc(F)ccc1Cc2noc(n2)[C@@H]3C[C@H](O)C[NH+]3Cc4ccc(N(C)C)cc4	7.7	7.4	7.55
156	20512	Fc1cc(F)cc(CNC(=O)[C@@H]([C@H]2CC[C@]3(CC[C@@H](NC(=O)Cc4ccccc4)[C@H]([C@@H]3[C@H]2O)C)C)C)c1	7.4	7.7	7.55
157	ZINC02316741	O=C(OCC)c1c2cc(OCc3c(C)ccc(c3)C)ccc2oc1-c4ccc(OC)cc4	7.4	7.7	7.55
158	SN00147842	Oc1cc(OCc2ccc(cc2)C=C)ccc1-c3c(-c4ccc(OC)cc4)c(no3)C	7.4	7.7	7.55
159	SN00149300	Fc1ccc(COc2ccc(-c3c(-c4ccc(OC)c(OC)c4)c(no3)C)c(O)c2)cc1	7.2	7.9	7.55
160	ZINC72324873	O=C1C(=C(c2c(OC)cc(OC)cc2O1)C)CC(=O)Nc3cccc4c3cc[nH]4	7.6	7.5	7.55

161	SN00151276	Oc1cc(OCc2ccccc2C)ccc1-c3c(-c4ccc(OC)c(OC)c4)c(no3)C	7.6	7.5	7.55
162	144369-45-9	O=C1C([C@@H]2CC[C@@H]([C@]2(CCOC(=O)C)C)[C@@H](/C=C/C(C)C)C)=C[C@@H]3[C@]4(O3)C[C@@H](OC(=O)C)CC[C@@]14C	7.5	7.6	7.55
163	ZINC68592313	O=P(OCC)(OCC)c1ccccc1OCCOS(=O)(=O)c2ccc(cc2)C	7.5	7.6	7.55
164	6915	Fc1ccc(C[NH+]2C[C@@H](O)C[C@H]2c3nc(no3)-c4cccc(c4)C)cc1	7.5	7.6	7.55
165	ZINC02284233	Fc1c(F)c(F)c(F)c(COc2ccc3c(c(c(o3)C(C)(C)C)C(OCC)=O)c2)c1F	7.5	7.6	7.55
166	3205	O[C@]1(C(=C[C@@H](OC(=O)CCCCCCC)[C@@H]2C(CC[C@@H](O)[C@]12C)(C)C)CO)CO	7.5	7.6	7.55
167	ZINC09128858	Clc1cc(Cl)ccc1COc2ccc(-c3c(-c4ccc(OC)c(OC)c4)c([nH]n3)C)c(O)c2	7.3	7.8	7.55
168	SN00149096	Clc1ccc(COc2ccc(-c3c(-c4ccc(OC)cc4)c(no3)C)c(O)c2)cc1	7.3	7.8	7.55
169	ZINC08918384	O[C@@]12CCN(C[C@H]2CCCC1)C(=O)CCC=3C(Oc4c(c(OCc5cccc(c5)C)ccc4C3C)C)=O	7.7	7.3	7.50
170	ZINC01322258	O=C1c2ccc(OC(=O)c3ccccc3C)cc2OC(=C1c4ccc(OC)c(OC)c4)C(OCC)=O	7.6	7.4	7.50
171	ZINC72324469	O=C1C(=C(c2ccc(OC)c(OC)c2O1)C)CC(=O)Nc3cccc4c3ccn4CCOC	7.6	7.4	7.50
172	ZINC03846553	Oc1ccccc1[C@H](Nc2ccc(OC)cc2)CC(=O)C=3C(=O)c4ccccc4N(C3[O-])C	7.6	7.4	7.50
173	SN00278139	O=C1c2ccc(O)c(-c3c(OC)ccc(C[C@H]4c5c(Oc6c(OC)cc7CCN([C@@H]1c7c6)C)c(O)c(OC)cc5CCN4C)c3)c2	7.5	7.5	7.50
174	ZINC48057026	Oc1cccc(OC)c1C(=O)/C=C/c2cc(OC)ccc2OC	7.5	7.5	7.50
175	467419-73-4	Brc1ccc2c([nH]c3c(nccc23)CCN(CCc4c5c(c6ccc(Br)cc6[nH]5)ccn4)C)c1	7.5	7.5	7.50
176	SN00149688	Clc1cc(Cl)ccc1COc2ccc(-c3c(-c4ccc(OC)c(OC)c4)c(no3)C)c(O)c2	7.5	7.5	7.50
177	ZINC04171591	O=C(OC(C)C)c1c(oc2ccc(OCc3c(C)ccc(c3)C)cc21)C	7.5	7.5	7.50
178	ZINC02096175	O=C1C(=C(c2ccc(OCc3cccc(c3)C)cc2O1)C)CC(OC)=O	7.4	7.6	7.50
179	ZINC01782009	Clc1cccc(COc2ccc3c(OC=C(c4ccccc4OC)C3=O)c2)c1	7.3	7.7	7.50
180	ZINC08994386	Oc1cc(OCc2ccc(cc2)C)ccc1-c3c(c([nH]n3)C)-c4ccc(OC)cc4	7.3	7.7	7.50
181	ZINC06719437	Oc1cc(OCc2ccccc2)ccc1-c3c(-c4ccc(OC)cc4)c(no3)C	7.3	7.7	7.50
182	ZINC12865161	O[C@]12CCN(C[C@@H]2CCCC1)C(=O)CCC=3C(Oc4c(c(OCc5cc(cc(c5)C)C)ccc4C3C)C)=O	7.5	7.4	7.45
183	ZINC01321752	O=C1[C@H](c2c3ccccc3n(Cc4ccccc4)c2)CC(=O)N1c5cccc(OC)c5	7.4	7.5	7.45
184	ZINC09129980	Clc1cccc(Cl)c1COc2ccc(-c3c(-c4ccc(OC)cc4)cnc(n3)N)c(O)c2	7.4	7.5	7.45
185	ZINC72325204	O=C(Nc1cnn(c1)C)CCCc2cn(c3ccccc32)C(C)C	7.4	7.5	7.45
186	SN00009552	O=C(c1c(OC)cc(OC)cc1OC)/C=C/c2ccc(OCc3ccccc3)c(OC)c2	7.4	7.5	7.45
187	ZINC08764447	O[C@H]([C@H](N(C(=O)CC=1C(Oc2c(c(OCC(C)=C)ccc2C1C)C)=O)C)C)c3ccccc3	7.4	7.5	7.45
188	ZINC03840748	O[C@H]1[C@@H](CC[C@]2(Cc3c(nc(s3)NC(=O)CC(C)(C)C)[C@H]([C@H]12)C)C)[C@@H](C(=O)N4CCCC4)C	7.4	7.5	7.45
189	MolPort-002-516-108	O=C(N[C@@H](Cc1c[nH]c2ccccc21)C(O)=O)COc3ccc4c(OC(=O)C(=C4C)C)c3	7.2	7.7	7.45
190	SN00149009	Clc1ccccc1COc2ccc(-c3c(-c4ccc(OC)c(OC)c4)c(no3)C)c(O)c2	7.2	7.7	7.45
191	ZINC02148733	O=S(=O)(N[C@@H]([C@@H](CC)C)C(Oc1ccc2C(C)=C(C(Oc2c1C)=O)C)=O)c3ccc(cc3)C	7.2	7.7	7.45
192	ZINC01799152	Fc1c(F)c(F)c(F)c(COc2ccc3C=C(c4ccccn4)C(Oc3c2)=O)c1F	7.2	7.7	7.45
193	ZINC20591923	Oc1cc(OCC[NH+]2CCOCC2)ccc1-c3c(-c4ccc(OC)c(OC)c4)c(n[nH]3)C	7.2	7.7	7.45
194	ZINC08764397	O=C(N[C@@H](Cc1c[nH]c2ccccc21)C)CC=3C(Oc4c(c5c(cc4C3C)c(c(o5)C)C)C)=O	7.6	7.3	7.45
195	SN00149119	Clc1ccc(COc2ccc(-c3c(-c4ccc(OC)cc4)c(no3)C)c(O)c2)cc1Cl	7.6	7.3	7.45
196	MolPort-027-852-800	Fc1ccc(-c2c([C@H](NC(=O)c3ccccn3)C)c(on2)C(O)=O)cc1	7.3	7.6	7.45
197	MolPort-027-852-111	Fc1ccc(-c2cnc3CO[C@@H](Cn23)CNCc4c(cccn4)C)cc1	7.3	7.6	7.45
198	ZINC20592055	Oc1cc(OCC[NH+]2CCOCC2)ccc1-c3c(c([nH]n3)C)-c4ccc(OC)cc4	7.3	7.6	7.45
199	ZINC04026460	O[C@@H]([C@H](O)C[C@@H](O)CCCCCC)CCCCCCCC([O-])=O	7.1	7.8	7.45
200	SN00245703	Clc1c(O)c(c(OC)c(c1C)C(Oc2c(c(c(c(OC)c2C)C(OC)=O)C)C)=O)C	7.1	7.8	7.45
201	ZINC01124363	Fc1ccccc1COc2ccc3C(=O)C(c4ccc(OC)c(OC)c4)=C(Oc3c2)C(OCC)=O	7.5	7.3	7.40
202	ZINC11867170	OC12CCN(C[C@@H]2CCCC1)C(=O)CC=3C(Oc4c(c(OCc5cccc(c5)C)ccc4C3C)C)=O	7.5	7.3	7.40
203	ZINC02401695	Fc1c(F)c(F)c(F)c(COc2ccc3C(=O)C(c4ccc5c(OCCO5)c4)=C(Oc3c2)C(OCC)=O)c1F	7.5	7.3	7.40
204	ZINC13569111	O=C(Nc1nc(cs1)C)CCC=2C(Oc3c(C2C)c(OC)cc4c3CCC(O4)(C)C)=O	7.5	7.3	7.40
205	MolPort-002-514-023	O=C(N[C@H](Cc1c[nH]c2ccccc21)C(O)=O)[C@H](Oc3ccc4c(OC(=O)C=5CCCCC45)c3)C	7.4	7.4	7.40
206	141544-63-0	O=C1c2c3c(c4ccccc4nc3C([C@H](OC)CNC(=O)/C(C)=C\C)=C1)ccn2	7.4	7.4	7.40
207	ZINC04090560	O=C(N[C@@H](Cc1c[nH]c2ccccc21)C(OC)=O)CCCc3c[nH]c4ccccc43	7.4	7.4	7.40

208	ZINC17058595	Oc1cc(OCC[NH+]2C[C@H](O[C@@H](C2)C)C)ccc1-c3c(-c4ccc(OC)c(OC)c4)c(n[nH]3)C	7.4	7.4	7.40
209	MolPort-001-745-794	O=C(NC[C@H]1C[C@H]2CCN1C[C@H]2c3cc(nn3C)-c4cccs4)c5ccc(OC)cc5	7.3	7.5	7.40
210	ZINC12875010	O=C1CN(C(=O)[C@@]2(c3c([C@H](CN12)c4ccccc4OC)c5ccccc5[nH]3)C)Cc6ccccc6OC	7.3	7.5	7.40
211	ZINC12865161	O[C@@]12CCN(C[C@@H]2CCCC1)C(=O)CCC=3C(Oc4c(c(OCc5cc(cc(c5)C)C)ccc4C3C)C)=O	7.3	7.5	7.40
212	MolPort-002-536-594	O=C(Nc1ccc2c(c1)C=CC(O2)=O)COc3cc(O)c4C(=O)C=C(Oc4c3)c5ccccc5	7.3	7.5	7.40
213	20208	Clc1ccc(S(=O)(=O)Nc2nc3c(s2)C[C@@]4(CC[C@H]([C@@H](O)[C@@H]4[C@H]3C)[C@H](C)C(=O)N(CC#C)C)C)cc1	7.3	7.5	7.40
214	ZINC02115645	O=C1C(=C(c2cc3c(OCN(c4ccccc4CC)C3)c(c2O1)C)C)Cc5ccccc5	7.3	7.5	7.40
215	ZINC02149058	Clc1c(Cl)ccc(COc2ccc3c(OC(=O)C(CCC(OCC)=O)=C3C)c2)c1	7.3	7.5	7.40
216	ZINC09130539	FC(F)(F)c1c(c(n[nH]1)-c2ccc(OCC(=O)N[NH3+])cc2O)-c3ccc(OC)c(OC)c3	7.2	7.6	7.40
217	ZINC03150514	O=C(Nc1cc(nc2ccccc21)C)CCCCCN3C(=O)c4ccccc4C3=O	7.2	7.6	7.40
218	ZINC02359452	Fc1c(F)c(F)c(F)c(F)c1OCC(Oc2ccc3c(c(o3)C)C(OCCOC)=O)c2)=O	7.2	7.6	7.40
219	ZINC01321303	Clc1ccccc1C(Oc2ccc3C(=O)C(c4ccc(OC)c(OC)c4)=C(Oc3c2)C(OCC)=O)=O	7.5	7.2	7.35
220	ZINC02094744	Clc1cccc(COc2ccc3c(OC(=O)C(=C3C)CCC(OCC)=O)c2)c1	7.5	7.2	7.35
221	ZINC08789240	O[C@@]1(CC[C@H]2[C@]1(CC[C@@H]3[C@]4(CC/C(=N\OCC(=O)N[C@H](c5ccccc5)C)C=C4CC[C@H]32)C)C)C#C	7.4	7.3	7.35
222	MolPort-042-647-983	Clc1ccc(C(=O)c2ccc3c([C@@H](c4cc(OC)c(OC)c(OC)c4)CC(O3)=O)c2O)cc1	7.4	7.3	7.35
223	SN00359873	O=C1c2c(O)cc(OC)c(c2O[C@H](C1)c3ccc(OC)cc3)-c4c(O)ccc([C@H]5CC(=O)c6c(O)cc(OC)cc6O5)c4	7.4	7.3	7.35
224	ZINC20466656	O[C@H]1[C@@H](CC[C@@]2(CC[C@@H](NC(=O)C3CC3)[C@H]([C@H]12)C)C)[C@@H](C(=O)NCc4ccc(OC)cc4)C	7.4	7.3	7.35
225	ZINC02417488	Clc1cccc(COc2ccc3C(=O)C(Oc4ccc(OC)cc4)=C(Oc3c2)C)c1	7.4	7.3	7.35
226	ZINC02416730	Clc1cccc(COc2ccc3C(=O)C(c4ccccc4)=C(Oc3c2)C(F)(F)F)c1	7.4	7.3	7.35
227	ZINC85879450	O=C(NC[C@@H]1CCC[NH+]2CCCC[C@H]12)CCCC3=Nc4ccccc4C(=O)N3	7.3	7.4	7.35
228	ZINC02148395	O=C1C(CCC(OCC)=O)=C(c2ccc(OCc3cc(cc(c3)C)C)cc2O1)C	7.3	7.4	7.35
229	ZINC08764768	O=C(OC)c1c(NC(=O)CC=2C(Oc3cc(OC)ccc3C2C)=O)c4c(OC)cccc4[nH]1	7.3	7.4	7.35
230	121979-39-3	O[C@@H]([C@@H](O)/C=C/C=C\CCCCC(O)=O)/C=C\CCCCC	7.3	7.4	7.35
231	ZINC70701314	Clc1ccccc1CN2CC(=O)N3C[C@H](c4c5ccccc5[nH]c4[C@@]3(C2=O)C)c6ccc(OC(C)C)c(OC)c6	7.3	7.4	7.35
232	ZINC48057026	Oc1cccc(OC)c1C(=O)/C=C\c2cc(OC)ccc2OC	7.3	7.4	7.35
233	ZINC03875039	O=C1C(CCCC)(COC(=O)CCC([O-])=O)C(=O)N(N1c2ccccc2)c3ccccc3	7.2	7.5	7.35
234	ZINC02148180	O=S(=O)(N[C@@H](Cc1c[nH]c2ccccc21)C(Oc3ccc4C(C)=CC(Oc4c3)=O)=O)c5ccc(cc5)C	7.2	7.5	7.35
235	ZINC02419763	Fc1c(F)c(F)c(F)c(F)c1COc2ccc3c(OC(=C(c4ccc(OC)cc4)C3=O)C)c2	7.2	7.5	7.35
236	ZINC02432591	Clc1cccc(COc2ccc3C(=O)C(Oc4ccccc4OCC)=C(Oc3c2)C)c1	7.1	7.6	7.35
237	ZINC18068961	Oc1cc(OCC(C)=C)ccc1-c2c(-c3ccc(OC)c(OC)c3)c(n[nH]2)C	7.1	7.6	7.35
238	ZINC01322844	Clc1cccc(COc2ccc3c(OC(=C(c4ccccc4OC)C3=O)C)c2)c1	7.0	7.7	7.35
239	ZINC12603405	Fc1ccc(-n2c(c([C@H]3C=C[C@H]([NH2+]Cc4ccc(C(C)C)cc4)C3)c(n2)C)C)cc1	7.4	7.2	7.30
240	MolPort-010-738-591	Fc1ccc-2c(NC3(CCN(CC3)C(=O)Cc4cccc(OC)c4)c5cccn25)c1	7.4	7.2	7.30
241	114820-25-6	O=C1C=C2CC[C@H]3[C@H]([C@@]2(CC1)C)CC[C@]4([C@H]([C@H]([C@@H]5CC(=C(C(O5)=O)C)C)C)CC[C@H]34)C	7.4	7.2	7.30
242	ZINC00947089	O=C(Oc1ccc2C(=O)C(Oc3ccccc3)=COc2c1)COc4ccc5C(C)=CC(Oc5c4)=O	7.4	7.2	7.30
243	SN00009667	O=C(c1c(OC)cccc1OC)/C=C/c2cc(OC)c(OC)c(OC)c2	7.4	7.2	7.30
244	ZINC12897517	Oc1cc(cc2c1C(=C(CC(=O)N[C@@H](Cc3c[nH]c4ccccc43)C)C(O2)=O)C)C	7.4	7.2	7.30
245	MolPort-038-386-307	O=C(N1CCN(CC1)c2ccccc2OC)c3c(noc3[C@H](O)C)-c4ccc(OC)cc4	7.2	7.4	7.30
246	105377-88-6	O[C@]([C@H]1CC[C@]([C@H](C1)C(C)=C)(C)C=C)(/C=C/C=C(C)C)C	7.2	7.4	7.30
247	ZINC05427801	FC(F)(F)c1c(-c2ccc(OC)cc2)c(on1)-c3ccc(OCC(C)=C)cc3O	7.2	7.4	7.30
248	ZINC02316868	Fc1c(F)c(F)c(F)c(F)c1OCC(Oc2ccc3c(c(o3)C)C(OCC)=O)c2)=O	7.2	7.4	7.30
249	MolPort-028-854-802	Oc1ccc2c(oc3c2nc4c(c3-c5ccc(OC)c(O)c5)cnn4C(C)C)c1C	7.2	7.4	7.30
250	MolPort-002-533-316	O=C(NCCc1ccc(OC)c(OC)c1)CC=2C(Oc3cc(c4c(C)coc4c3C2C)C)=O	7.2	7.4	7.30
251	SN00345792	O=C1C=C(Oc2cc(OC)c(C3=CC(=O)C(OC)=C(O3)C)c(OC)c12)c4ccccc4	7.2	7.4	7.30
252	MolPort-002-518-869	O=S(=O)(N[C@@H]([C@@H](CC)C)C(Oc1ccc-2c(OC(=O)c3ccccc32)c1)=O)c4ccc(cc4)C	7.2	7.4	7.30

253	ZINC01797720	Fc1c(F)c(F)c(F)c(COc2ccc3C=C(c4nc5ccccc5s4)C(Oc3c2)=O)c1F	6.9	7.7	7.30
254	MolPort-029-886-452	Oc1c(O)c2c([C@@H](c3cccc(OC)c3OCCc4ccc(OC)cc4)CC(O2)=O)c5c1C(=O)C=C(O5)c6ccccc6	7.3	7.3	7.30
255	ZINC79210340	O=S(=O)(Nc1cc(OC)c(OC)cc1Cc2c3cc(OC)c(OC)cc3ccn2)c4ccc5c(c4)C(=O)N(C(=O)N5CC)CC	7.3	7.3	7.30
256	9853	Fc1ccc(C(=O)N2Cc3c(ncn3Cc4ccc(OC)cc4)C[C@H]2C(OC)=O)cc1	7.3	7.3	7.30
257	ZINC68573697	FC(F)(C(F)(F)O[C@@](F)(C(F)(F)F)C(Oc1cc(cc(c1C)C)C)=O)C(F)(F)F	7.3	7.3	7.30
258	MolPort-000-813-035	O=C(N[C@@H](Cc1c[nH]c2ccc(O)cc21)C(O)=O)COc3ccc4c(OC(=O)C(=C4C)C)c3	7.3	7.3	7.30
259	MolPort-028-855-502	Oc1ccc2c(oc3c2nc4c(c3-c5cc(OC)c(OC)c(OC)c5)cnn4C(C)C)c1C	7.3	7.3	7.30
260	ZINC12865161	O[C@@]12CCN(C[C@H]2CCCC1)C(=O)CCC=3C(Oc4c(c(OCc5cc(cc(c5)C)C)ccc4C3C)C)=O	7.3	7.3	7.30
261	ZINC20622826	Oc1c(-c2c(c([nH]n2)C)-c3ccc(OC)cc3)ccc(OCC[NH+]4CCOCC4)c1C	7.3	7.3	7.30
262	ZINC17858262	Oc1cc(OCC(C)=C)ccc1-c2c(-c3ccc(OC)cc3)c(n[nH]2)C	7.3	7.3	7.30
263	ZINC02147827	O=C1c2c(OC(=C1c3ccc4c(OCCCO4)c3)C)cc(OCc5ccc(cc5)C)c(CC)c2	7.3	7.3	7.30
264	SN00149094	Brc1ccc(COc2ccc(-c3c(-c4ccc(OC)cc4)c(no3)C)c(O)c2)cc1	7.3	7.3	7.30
265	ZINC13124861	Brc1cc([C@H]2CC(=N[NH2+]2)c3cc(Cl)ccc3O)c4c(COCO4)c1	7.3	7.3	7.30
266	MolPort-002-520-217	O=C(N[C@H](Cc1c[nH]c2ccccc21)C(O)=O)CC3=CC(Oc4cc(OC)ccc43)=O	7.1	7.5	7.30
267	MolPort-002-519-718	O=C(N[C@@H]([C@@H](CC)C)C(O)=O)[C@H](Oc1ccc2c(OC(=O)C(=C2C)C)c1C)C	7.1	7.5	7.30
268	ZINC02115916	O=C1C(C2=CC(Oc3c(c(OCc4cc(cc(c4)C)C)ccc23)C)=O)=Cc5ccccc5O1	7.1	7.5	7.30
269	ZINC06755178	Oc1cc(OCC(C)=C)ccc1-c2c(-c3ccc(OC)c(OC)c3)c(no2)C	7.1	7.5	7.30
270	ZINC09130710	Fc1ccccc1COc2ccc(-c3c(-c4ccc(OC)c(OC)c4)c([nH]n3)C)c(O)c2	7.1	7.5	7.30
271	SN00098390	O=C1c2c(OC)c(OC)ccc2[C@@H](O1)c3cc(OC)c(OC)cc3Cc4c5cc(OC)c(OC)cc5ccn4	7.1	7.5	7.30
272	ZINC70692199	O=C1CN(CCC[NH+](CC)CC)C(=O)[C@@]2(c3c([C@@H](CN12)c4ccc(OCCC)c(OC)c4)c5ccccc5[nH]3)C	7.1	7.5	7.30
273	MolPort-002-535-528	O=C1c2ccccc2-c3c4c(c5cc(OCC(=O)NCCc6ccc(OC)c(OC)c6)ccc5n14)ccn3	7.0	7.6	7.30
274	SN00379562	O=C1C=C[C@]2([C@H](C(CC[C@H]2C1(C)C)=C)COc3c(OC)cc4C=CC(Oc4c3OC)=O)C	7.0	7.6	7.30
275	SN00351686	O=C1c2ccc(OC)c(-c3c(OC)ccc(C[C@H]4c5c(Oc6c(OC)cc7CCN([C@@H]1c7c6)C)c(O)c(OC)cc5CCN4C)c3)c2	7.4	7.1	7.25
276	SN00168965	Oc1c(OC)cc2CC[N+](=C3Cc4ccc(OC)c(Oc5ccc(C[C@@H]6c7cc(Oc1c23)c(OC)cc7CCN6C)cc5)c4)C	7.4	7.1	7.25
277	ZINC20466317	O[C@H]1[C@H](CC[C@@]2(CC[C@H](NC(=O)c3ccccc3)[C@@H]([C@H]12)C)C)[C@@H](C(=O)N4CCCC4)C	7.4	7.1	7.25
278	ZINC02112486	O=C1c2ccccc2-c3cc4c(OCN(C4)c5ccccc5CC)c(c3O1)C	7.3	7.2	7.25
279	6895	Fc1c(C)ccc(-c2noc(n2)[C@@H]3C[C@@H](O)C[NH+]3Cc4ccc(cc4)C)c1	7.3	7.2	7.25
280	MolPort-029-885-299	O=C(N[C@@H](C(C)C)c1nc2ccccc2[nH]1)CC=3C(Oc4c(c5c(cc4C3C)c(co5)C)C)=O	7.3	7.2	7.25
281	MolPort-002-536-261	O=C(NCCc1c[nH]c2cc(OC)ccc21)CC=3C(Oc4c(c5c(cc4C3C)c(c(o5)C)C)C)=O	7.3	7.2	7.25
282	ZINC12296352	O=C(NC[C@H]1CCC[NH+]2CCCC[C@H]12)Cc3csc(n3)C	7.3	7.2	7.25
283	MolPort-038-386-306	O=C(N1CCC[C@@H]1COC)c2c(noc2[C@@H](O)C)-c3ccc(OC)cc3	7.3	7.2	7.25
284	ZINC13653587	Oc1ccc2C(=O)/C(Oc2c1C[NH+]3CCCC[C@H]3C)=C/c4ccc(OC)cc4OC	7.2	7.3	7.25
285	ZINC04061925	O=C1[C@@H](C[C@H](C(C(C)C)(C)C)C[C@@H]1[C@H]2c3ccc(OC)c(OC)c3C(O2)=O)C	7.2	7.3	7.25
286	MolPort-002-532-941	O=C1c2ccccc2OC(NC(=O)c3ccc(OC)cc3)=C1c4cccc(OC)c4	7.1	7.4	7.25
287	ZINC08879630	O=C(Nc1cccc2c1cccn2)CCC=3C(Oc4cc(OCC(C)=C)ccc4C3C)=O	7.1	7.4	7.25
288	ZINC30879908	Fc1cccc(C(=O)NC[C@@H]2C=C([C@@H](C[C@@H]2C(C)C)CC(=O)N3CCN(CC3)C(=O)C)C)c1	7.1	7.4	7.25
289	ZINC03846576	O=C(C[C@H](Nc1cccc(N)c1)c2ccc3c(OCO3)c2)C=4C(=O)c5ccccc5N(C4[O-])C	7.1	7.4	7.25
290	ZINC00083661	O=C(OC)c1c(oc2ccc(OCc3c(C)ccc(c3)C)cc21)C	7.1	7.4	7.25
291	ZINC02096181	O=S(=O)(N[C@H]([C@H](CC)C)C(Oc1ccc2c(OC(=O)C=C2C)c1)=O)c3ccc(cc3)C	6.8	7.7	7.25
292	ZINC11867175	Clc1c(Cl)ccc(COc2ccc3c(OC(=O)C(CCC(=O)N4CC[C@]5(O)CCCC[C@H]5C4)=C3C)c2)c1	7.4	7.0	7.20
293	MolPort-028-855-910	O=C(NCCN1C(=Nc2c(C1=O)cccn2)C)CCCc3c[nH]c4ccccc43	7.2	7.2	7.20
294	ZINC02817963	COc1ccc([C@H]2c3c(CC[NH2+]2)c4ccccc4[nH]3)cc1CSc5nc6ccccc6s5	7.2	7.2	7.20
295	ZINC62001394	O=C1C(=C(c2c(OC)cc(OC)cc2O1)C)CC(=O)Nc3cccc4c3ccn4C	7.2	7.2	7.20
296	ZINC02092024	O=S(=O)(N[C@H]([C@H](CC)C)C(Oc1ccc2C(CC)=CC(Oc2c1C)=O)=O)c3ccc(cc3)C	7.2	7.2	7.20
297	ZINC08791608	O=C(NCCCOC(C)C)CC=1C(Oc2cc3c(c(-c4ccccc4)co3)cc2C1C)=O	7.2	7.2	7.20

298	8166	Fc1cnc(-c2noc(n2)[C@@H]3CCC[NH+]3Cc4ccccc4OC)cc1	7.2	7.2	7.20
299	ZINC02105206	O=C1CN(C(=O)[C@H]2Cc3c4ccccc4[nH]c3[C@H](N12)c5cccc(OC)c5)CCOC	7.0	7.4	7.20
300	MolPort-005-910-295	O=C(N[C@@H](Cc1c[nH]c2ccccc21)C(O)=O)COc3ccc4c(C(=O)N5C(=N4)CCC5)c3	7.0	7.4	7.20
301	ZINC04025067	C[C@@H]1CC(=C[C@H](C21COC(OC2)CCCCCC)C)C	7.0	7.4	7.20
302	ZINC09130732	Clc1c(Cl)ccc(COc2ccc(-c3c(-c4ccc(OC)c(OC)c4)c([nH]n3)C)c(O)c2)c1	7.0	7.4	7.20
303	ZINC02092577	O=S(=O)(N[C@@H](CC(C)C)C(Oc1cc(cc2c1C(=C(C(O2)=O)C)C)C)=O)c3ccc(cc3)C	6.9	7.5	7.20
304	SN00268509	Oc1ccc2cc1Oc3ccc(C[C@H]4c5c(Oc6c(OC)cc7CCN([C@H](c7c6)C2)C)c(OC)c(OC)c(OC)c5CCN4C)cc3	6.9	7.5	7.20
305	ZINC12603350	Clc1cccc(C[NH2+][C@@H]2C=C[C@@H](C2)c3c(nn(c3C)-c4ccc(F)cc4)C)c1	7.3	7.1	7.20
306	ZINC30883993	O[C@H]1[C@H](CC[C@@]2(CC[C@H](NC(=O)c3cnccn3)[C@@H]([C@H]12)C)C)[C@@H](C(=O)N(CCC#N)C)C	7.3	7.1	7.20
307	MolPort-001-742-517	O=C(c1c(OC)cc(OC)cc1OC)/C=C\c2ccc(OC)c(OC)c2	7.3	7.1	7.20
308	ZINC02115467	Clc1cccc(-c2c3c(c4ccccc4[nH]3)cc(n2)C(=O)NCCc5ccc(OC)cc5)c1	7.3	7.1	7.20
309	MolPort-002-517-246	O=C1C=C(c2ccc(OCc3cccc(OC)c3)c(c2O1)COc4cccc(OC)c4)c5ccccc5	7.1	7.3	7.20
310	ZINC02107236	O=S(Oc1ccc2C(=O)C(c3ccc(OC)c(OC)c3)=C(Oc2c1)C(OCC)=O)(=O)C	7.1	7.3	7.20
311	ZINC68601377	O=C(N[NH2+]/C=C(/C=1C(Oc2ccccc2C1)=O)C)CCC(=O)N/N=C(/C=3C(Oc4ccccc4C3)=O)C	7.1	7.3	7.20
312	ZINC70692423	Clc1cc(Cl)ccc1CN2CC(=O)N3C[C@H](c4c5ccccc5[nH]c4[C@]3(C2=O)C)c6ccc(CC)cc6	7.1	7.3	7.20
313	MolPort-044-544-310	O=C1C=C(c2cc(cc(c2O1)C)C)C3=Cc4c(OC)c(OC)c5c6cc(OC)c(OC)cc6ccc5c4CCN3C	7.1	7.3	7.20
314	ZINC04387787	Fc1c(OCC)c(F)c(F)c(COc2ccc3c(c(c(o3)C)C([O-])=O)c2)c1F	7.1	7.3	7.20
315	1877	Oc1c(c(O)cc(c1C(Oc2c(c(OC)c(c(c2C)C)C(Oc3c(c(OC)c(c(c3C)C)C([O-])=O)C)=O)C)=O)C)C	7.1	7.3	7.20
316	ZINC08479128	O=C(Nc1ccccc1OCC)CC=2C(Oc3cc(c4c(C)coc4c3C2C)C)=O	7.1	7.3	7.20
317	ZINC04100775	Clc1ccccc1/C=C\C(=O)c2c(OC)cc(OC)cc2OC	7.1	7.3	7.20
318	ZINC01111774	Clc1c(Cl)ccc(COc2ccc3c(OC(=O)C(=C3C)CC(OC)=O)c2)c1	7.1	7.3	7.20
319	ZINC01118985	Clc1cccc(COc2ccc3c(OC(=O)C(=C3C)CCC(OCC)=O)c2C)c1	7.1	7.3	7.20
320	SN00063216	O=C1c2cc(ccc2OC=3C(=O)N([C@@H](C13)c4ccc(O)c(OC)c4)CCc5ccc(OC)cc5)C	7.4	6.9	7.15
321	ZINC02156156	O=C(N[C@H]([C@H](CC)C)C([O-])=O)CC=1C(Oc2c(c3c(cc2C1C)c(c(o3)C)C)C)=O	7.4	6.9	7.15
322	ZINC02091864	O=C1C(=C(c2ccc(OCc3cc(cc(c3)C)C)cc2O1)C)CC(OC)=O	7.4	6.9	7.15
323	MolPort-029-886-141	O=C1[C@H](NC(=O)N1Cc2ccc(OC)cc2)CC(=O)NCCc3c[nH]c4ccc(OC)cc43	7.4	6.9	7.15
324	ZINC35425888	COc1c(OC)c(OC)cc(C[NH+]2CCC[C@H]2c3nc(no3)-c4cnc(OC)cc4)c1	7.3	7.0	7.15
325	SN00155517	Oc1c(c(OC)cc(OC)c1CC=C(C)C)C(=O)/C=C/c2c(OC)cc(OC)cc2OC	7.2	7.1	7.15
326	ZINC20611253	O=C(N[C@@H]([C@@H](CC)C)C([O-])=O)[C@H](N1C(=O)Nc2ccccc2C1=O)CC(C)C	7.2	7.1	7.15
327	ZINC08764272	O=C(N[C@@H](Cc1c[nH]c2ccccc21)C)CC=3C(Oc4cc5c(cc4C3C)c(C)co5)=O	7.2	7.1	7.15
328	ZINC03840780	Fc1cccc(F)c1C(=O)Nc2nc3c(s2)C[C@]4(CC[C@@H]([C@H](O)[C@@H]4[C@H]3C)[C@H](C(=O)N(CC=C)C)C)C	7.2	7.1	7.15
329	ZINC04252739	O=C(c1c(OC)cccc1OC)/C=C\c2cc(OC)c(OC)c(OC)c2	7.2	7.1	7.15
330	ZINC20757263	OC1=C([C@@H]2c3c(c4ccccc4[nH]3)CCN2C)C(=O)N(C(=O)N1c5ccc(OC)cc5)C	7.1	7.2	7.15
331	SN00077518	O=C1[C@H-]([C@@H](N(CCOC)C1=O)c2ccc(OC)c(OC)c2)C(=O)c3ccc(OC)cc3	7.1	7.2	7.15
332	ZINC12891809	O=C(Nc1ccc2c(c1)cc[nH]2)CC=3C(Oc4cc(OCC(C)=C)ccc4C3C)=O	7.1	7.2	7.15
333	ZINC02094302	O=C1c2ccccc2-c3ccc(OCC(=O)c4c(n(c5ccccc54)C)C)c(c3O1)C	7.1	7.2	7.15
334	ZINC70666401	Brc1ccc2ccn(CCC(=O)Nc3cccc4c3cc[nH]4)c2c1	7.1	7.2	7.15
335	ZINC13127298	Oc1cc(OC)ccc1-c2c(-c3ccc(OC)c(OC)c3)c([nH]n2)C	7.0	7.3	7.15
336	ZINC79189085	O=C(NCCn1ccc2c(OC)cccc21)C3=CC(=O)N(c4ccccc34)C	7.0	7.3	7.15
337	ZINC01111387	O=C1C(=C(c2ccc(OCc3cccc(c3)C)c(c2O1)C)C)CC(OC)=O	7.0	7.3	7.15
338	ZINC02159445	O=C1C=C(CCC)c2ccc3c(CN(CO3)c4c(C)ccc(c4)C)c2O1	7.0	7.3	7.15
339	MolPort-044-830-921	O[C@@H]1[C@@H](n2cnc3cc(OC)c(OC)cc32)[C@@H]4OC[C@@H](O4)[C@H]1NCc5c(noc5C)C	7.0	7.3	7.15
340	ZINC70687971	Brc1cccc(COc2ccc3C(=C(CC(=O)N4CC[C@@]5(O)CCCC[C@H]5C4)C(Oc3c2C)=O)C)c1	7.0	7.3	7.15
341	SN00151294	FC(F)(F)c1c(c(on1)-c2ccc(OCC(C)=C)cc2O)-c3ccc(OC)c(OC)c3	6.9	7.4	7.15
342	MolPort-009-649-059	O=C1C[C@@H](c2c(N1)c(cn2-c3c(OC)ccc(OC)c3)C(O)=O)c4ccc(OC)c(O)c4	6.9	7.4	7.15
343	ZINC01322799	O=C1c2ccc(OCc3cccc(OC)c3)cc2OC(=C1c4ccc(OC)c(OC)c4)C(OCC)=O	6.9	7.4	7.15

344	ZINC12901891	O=C1C(=C(c2c(OC)cc3c(CCC(O3)(C)C)c2O1)C)CC(=O)Nc4cc(OC)cc(OC)c4	6.9	7.4	7.15
345	12169	CC([C@@H]1C[C@@H](C(=C[C@H]1C[NH+](C)C)C)Cc2nc3ccc(OC)nc3[nH]2)C	7.2	7.0	7.10
346	ZINC02093820	O=C(c1c2cc(OC)ccc2n(c1C)C)COc3ccc4C(=C(Cc5ccccc5)C(Oc4c3C)=O)C	7.2	7.0	7.10
347	ZINC01447881	Clc1ccc(COc2ccc3C(=O)C(Oc4ccc(cc4)C(OC)=O)=COc3c2)c1	7.2	7.0	7.10
348	ZINC09266649	Clc1c(Cl)ccc(COc2ccc3C(=O)C(c4ccc5c(OCCO5)c4)=COc3c2)c1	7.2	7.0	7.10
349	ZINC12881699	O=C1c2c(OC)c(OC)ccc2[C@@H]3c4c([C@@H](CN13)c5ccc(OCC)c(OC)c5)c6ccccc6[nH]4	7.1	7.1	7.10
350	78518-73-7	O[C@H]1CC[C@]2([C@H](OC1(C)C)CC[C@](O)([C@@H]2CC[C@H]3C(=CC[C@H]4[C@H](C3(C)C)CC[C@]4(O)C)C)C)C	7.1	7.1	7.10
351	ZINC20467354	Fc1cccc(NC(=O)N[C@@H]2CC[C@@]3(CC[C@H]([C@H](O)[C@@H]3[C@H]2C)[C@@H](C(=O)N4CCCC4)C)C)c1	7.1	7.1	7.10
352	MolPort-028-855-487	Oc1ccc2c(oc3c2nc4c(c3-c5ccc(OC)c(OC)c5)cnn4C(C)C)c1C	7.1	7.1	7.10
353	ZINC04252699	O=C(c1c(OC)cc(OC)cc1OC)/C=C/c2ccc(OC)c(OC)c2	7.1	7.1	7.10
354	ZINC01797677	O=C(OCCOC)c1c(oc2ccc(OCc3c(C)ccc(c3)C)cc21)C	7.1	7.1	7.10
355	ZINC02122276	O=C1C=C(CCCC)c2ccc3c(CN(CO3)c4c(C)ccc(c4)C)c2O1	7.0	7.2	7.10
356	ZINC08993442	Clc1ccccc1C(Oc2ccc3C(=O)/C(Oc3c2C)=C\c4cn(c5ccc(OC)cc45)CC)=O	6.9	7.3	7.10
357	SN00151259	Fc1ccccc1COc2ccc(-c3c(-c4ccc(OC)cc4)c(no3)C(F)(F)F)c(O)c2	6.9	7.3	7.10
358	ZINC02122006	Brc1ccc(N2COc3c(c4c(cc3C2)C(=C(Cc5ccccc5)C(O4)=O)C)C)c(C)c1	6.9	7.3	7.10
359	ZINC08739938	FC(F)(F)c1c(c(n[nH]1)-c2ccc(O)cc2O)-c3ccc(OC)c(OC)c3	6.9	7.3	7.10
360	ZINC02149345	Clc1c(Cl)ccc(COc2ccc3C(=C(CCC([O-])=O)C(Oc3c2C)=O)C)c1	6.8	7.4	7.10
361	ZINC20111812	Fc1ccc(C(=O)C[C@H]2c3c(OC)c4c(OCO4)cc3CC[NH+]2C)cc1	7.2	6.9	7.05
362	20391	Fc1cc(F)cc(CNC(=O)[C@H]([C@@H]2CC[C@]3(CC[C@@H](NC(=O)CC(C)(C)C)[C@@H]([C@H]3[C@@H]2O)C)C)C)c1	7.2	6.9	7.05
363	ZINC15674342	Fc1c(C)ccc(-c2noc(n2)[C@H]3C[C@H](O)C[NH+]3Cc4cc5ccccc5o4)c1	7.2	6.9	7.05
364	MolPort-001-910-598	OC(=O)CCc1ccc(n1-c2ccc(OC)cc2OC)-c3ccc(OC)cc3	6.9	7.2	7.05
365	MolPort-002-525-441	O=C(N[C@@H](Cc1c[nH]c2ccccc21)C(O)=O)[C@H](Oc3cc(cc4c3C5=C(CCC5)C(O4)=O)C)C	6.9	7.2	7.05
366	MolPort-001-838-316	Oc1cc(OC)c(CCC)cc1-c2c(-c3ccc4c(OCCCO4)c3)c[nH]n2	6.9	7.2	7.05
367	ZINC01448111	Clc1cccc(COc2ccc3C(=O)C(Oc4ccccc4OC)=COc3c2)c1	6.9	7.2	7.05
368	19304	Fc1ccc(-c2c([C@H](NC(=O)c3cc(n(n3)C)C)C)c(on2)C([O-])=O)cc1	6.7	7.4	7.05
369	169340-01-6	O[C@]1(CC[C@H]2[C@](C[C@@H](OC(=O)C)[C@H]3C(CCC[C@]23C)(C)C)(C1)C)[C@H]4CC(=O)C(=C[C@H]4O)C	6.7	7.4	7.05
370	ZINC30883259	CC([C@H]1C[C@@H](C(=C[C@@H]1C[NH+]2CCN(CC2)C)C)Cc3nnc(o3)-c4ccc(OC)cc4)C	7.3	6.8	7.05
371	ZINC85874833	OC=1C(=O)C=C(N(C1C[NH+]2[C@@H]3CC(C[C@@](C3)(C2)C)(C)C)C)C	7.1	7.0	7.05
372	ZINC02341762	O=C(OCCOC)c1c(n(c2ccc(OC(=O)c3cccs3)cc21)C)C	7.1	7.0	7.05
373	ZINC05646268	FC(F)(F)c1c(c(n[nH]1)-c2ccc(OC)cc2O)-c3ccc(OC)c(OC)c3	7.1	7.0	7.05
374	MolPort-002-525-094	O=C(NCc1ccccc1)c2cc3c4ccccc4[nH]c3c(n2)-c5ccc(OCC)c(OC)c5	7.1	7.0	7.05
375	SN00142352	O=C1C(=O)c2c(O)c(c(OC)c(OC)c2[C@]3(CCCC([C@@H]13)(C)C)C)[C@@H](COC)C	7.1	7.0	7.05
376	SN00309254	Oc1ccc2cc1Oc3ccc(C[C@H]4c5c(Oc6c(OC)cc7CCN[C@H](c7c6)C2)c(OC)c(OC)cc5CCN4C)cc3	7.0	7.1	7.05
377	ZINC08991161	O=S(Oc1ccc2C(=O)/C(Oc2c1C)=C\c3cn(c4ccccc34)CC)(=O)c5ccccc5	7.0	7.1	7.05
378	ZINC09129150	FC(F)(F)c1c(-c2ccc(OC)cc2)c(on1)-c3ccc(OCc4ccccc4)cc3O	7.0	7.1	7.05
379	SN00265756	O=C(c1c(OC)cc2c(OCO2)c1OC)/C=C/c3cc(OC)c(OC)c(OC)c3OC	7.0	7.1	7.05
380	SN00379562	O=C1C=C[C@]2([C@H](C(CC[C@@H]2C1(C)C)=C)COc3c(OC)cc4C=CC(Oc4c3OC)=O)C	7.0	7.1	7.05
381	ZINC01463420	O=C(OCC)c1c2cc(OCc3c(C)ccc(c3)C)ccc2n(c1C)C	7.0	7.1	7.05
382	ZINC03846558	Oc1ccccc1[C@H](Nc2cccc3cccc(N)c32)CC(=O)C=4C(=O)c5ccccc5N(C4[O-])C	6.8	7.3	7.05
383	ZINC20611343	O=C(N[C@@H]([C@@H](CC)C)C([O-])=O)[C@H](N1C(=O)Nc2ccccc2C1=O)Cc3ccccc3	6.8	7.3	7.05
384	6277-14-1	OC(=O)[C@@]1(CC[C@@]2(CC[C@]3(C([C@H]2C1)=CC(=O)[C@@H]4[C@]3(CC[C@@H]5C([C@@H](OC(=O)C)CC[C@]54C)(C)C)C)C)C)C	6.8	7.3	7.05
385	MolPort-000-852-534	O=C(NCCc1c[nH]c2ccccc21)c3ccccc3NC(=O)c4ccc(OC)cc4	7.2	6.8	7.00
386	ZINC08790706	O=C(N[C@H](CNC(=O)[C@@]12CC[C@@](C1(C)C)(C(O2)=O)C)C)[C@]34CC[C@](C3(C)C)(C(O4)=O)C	7.1	6.9	7.00
387	ZINC03999931	O=C1CN2c3ccccc3C([C@]2(N1)/C=C/c4ccccc4OCCCCCC)(C)C	7.1	6.9	7.00
388	163597-60-2	O[C@@]1(C2=CC[C@H]([C@@]1(CCC(=CCC[C@]3([C@H](O3)C2=O)C)C)C)C)C	7.0	7.0	7.00

389	MolPort-002-536-237	O=C(Nc1c2ccccc2oc1C3=CC(Oc4c(cc(cc43)C)C)=O)c5ccccc5OC	7.0	7.0	7.00
390	SN00083390	O=C(Nc1cc(OC)c(OC)cc1Cc2c3cc(OC)c(OC)cc3ccn2)c4ccc(OCCCC)cc4	7.0	7.0	7.00
391	23212	O[C@@H]1CC[C@@H]2[C@@]3(CO[C@H](O[C@H]3CC[C@]2([C@@H]1CC(=O)NCc4ccc(OC)cc4)C)COc5ccccc5)C	7.0	7.0	7.00
392	ZINC02401244	Clc1c(Cl)cc(COc2ccc3C(=O)C(Oc4ccccc4OC)=COc3c2)cc1	7.0	7.0	7.00
393	ZINC12660555	Clc1ccc2c(c(NC(=O)C[NH+]3CCc4cc(OC)c(OC)cc4[C@@H]3C)c([nH]2)C(OC)=O)c1	7.0	7.0	7.00
394	MolPort-002-509-346	Oc1cc(O)c(-c2c(-c3ccc(OC)cc3)c[nH]n2)cc1CC	6.9	7.1	7.00
395	ZINC05646116	FC(F)(F)c1c(c(n[nH]1)-c2ccc(OCC)cc2O)-c3ccc(OC)c(OC)c3	6.9	7.1	7.00
396	ZINC02094353	O=C1C(CCC(OCC)=O)=C(c2ccc(OCc3cccc(c3)C)cc2O1)C	6.9	7.1	7.00
397	ZINC03846618	O=C(C[C@@H](Nc1ccc(N)cc1)c2ccc(N(C)C)cc2)C=3C(=O)c4ccccc4N(C3[O-])C	6.9	7.1	7.00
398	ZINC09129706	Clc1cccc(Cl)c1COc2ccc(-c3c(-c4ccc(OC)c(OC)c4)cncn3)c(O)c2	6.9	7.1	7.00
399	ZINC20113162	FC(F)(F)c1cccc(N2C(=O)NC(=O)C([C@H]3c4c(OC)c5c(OCO5)cc4CCN3C)=C2O)c1	6.9	7.1	7.00
400	ZINC02119961	Clc1cccc(COc2ccc3c(OC(=O)C=C3C=4C(Oc5ccccc5C4)=O)c2)c1	6.9	7.1	7.00
401	ZINC06662659	FC(F)(F)c1c(c(on1)-c2ccc(OC)cc2O)-c3ccc(OC)c(OC)c3	6.8	7.2	7.00
402	ZINC00129480	O=C(Oc1ccc2C(C)=CC(Oc2c1)=O)COc3ccc(CC)cc3	6.8	7.2	7.00
403	ZINC02283570	Fc1c(F)c(F)c(F)c(COc2ccc3c(c(c(o3)C)C(OCC)=O)c2)c1F	6.8	7.2	7.00
404	MolPort-039-195-602	Clc1ccc(C(=O)c2ccc3c([C@H](c4cc(OC)c5c(OCO5)c4)CC(O3)=O)c2O)cc1	6.8	7.2	7.00
405	ZINC05484338	Oc1cc(OCc2cc(ccc2C)C)ccc1-c3c(-c4ccc(OC)cc4)cncn3	6.8	7.2	7.00
406	ZINC06669439	FC(F)(F)c1c(c(on1)-c2ccc(OCC)cc2O)-c3ccc(OC)c(OC)c3	6.7	7.3	7.00
407	19317	Fc1ccc(-c2c([C@@H](NS(=O)(=O)c3ccc(OC)cc3)C)c(on2)C([O-])=O)cc1	6.7	7.3	7.00
408	ZINC68591815	Oc1c(OC)cc([C@@H](Nc2nc(c(s2)C)C)CC(=O)C=3C(=O)c4ccccc4N(C3[O-])C)cc1OC	6.7	7.3	7.00
409	6883	Fc1c(C)ccc(-c2noc(n2)[C@@H]3C[C@H](O)C[NH+]3Cc4ccc(F)cc4)c1	7.2	6.7	6.95
410	ZINC70687765	FC(F)(F)c1cccc(COc2ccc3C(=C(CCC(=O)N4CC[C@]5(O)CCCC[C@H]5C4)C(Oc3c2C)=O)C)c1	7.0	6.9	6.95
411	ZINC02112065	Clc1c(OCC(=O)c2c(n(c3ccc(OC)cc23)C)cc4c(C(=C(CCC(OCC)=O)C(O4)=O)C)c1	7.0	6.9	6.95
412	ZINC02111331	O=C(c1c2ccccc2n(c1C)C)COc3cc4c(cc3CC)C(C)=CC(O4)=O	7.0	6.9	6.95
413	12308	Fc1cccc(C(=O)NC[C@@H]2C=C([C@@H](C[C@@H]2C(C)C)CC(=O)NCCOC)C)c1	7.0	6.9	6.95
414	149355-76-0	O=C1c2c(c(c3-c4cc(OC)c(O)cc4CCn23)-c5ccc(OC)c(OC)c5)-c6cc(OC)c(O)cc6O1	7.0	6.9	6.95
415	SN00309240	Oc1c(OC)c(OC)nc(C/C=C(/C/C=C\C(=C\[C@@H]([C@H](OC)[C@@]2([C@@H](O2)C)C)C)C)C)c1C	6.9	7.0	6.95
416	MolPort-001-626-086	O=C(Nc1cc(OC)c(OC)cc1Cc2c3cc(OC)c(OC)cc3ccn2)c4ccc(cc4)C	6.9	7.0	6.95
417	ZINC01321302	O=C1c2ccc(OC(=O)c3c(oc4ccc(OC(=O)C)cc43)C)cc2OC(=C1c5ccc(OC)c(OC)c5)C(OCC)=O	6.9	7.0	6.95
418	ZINC16364038	Clc1ccc(N[C@H](CC(=O)C=2C(=O)c3ccccc3N(C2[O-])C)c4ccc(N(C)C)cc4)cc1	6.9	7.0	6.95
419	ZINC04278011	Fc1ccc(N2C(=O)N[C@H]3CCN([C@H]3C2=O)C(=O)c4ccc5ccccc5c4)cc1	6.9	7.0	6.95
420	MolPort-044-180-963	Clc1ccccc1-c2c(nc(s2)C)C(=O)N(C[C@@H]3CCCN4CCCC[C@H]34)CCOC	6.9	7.0	6.95
421	ZINC01280062	O=C(c1c(cc2C([C@H](C(c2c1)(C)C)C)(C)C)C)C[C@H]3c4c(OC)c5c(OCO5)cc4CC[N+]3(C)C	6.9	7.0	6.95
422	ZINC18166941	FC(F)(F)c1c(c([nH]n1)-c2ccc(O)cc2O)-c3ccc(OC)cc3	6.7	7.2	6.95
423	SN00353994	O[C@H]1CC[C@]2([C@@H](C(CC[C@H]2C1(C)C)=C)COc3c(OC)cc4C=CC(Oc4c3OC)=O)C	6.7	7.2	6.95
424	15714	Clc1cc(C(=O)Nc2ccc([C@@H](O)[C@H]3COCC(=O)N3Cc4ccccn4)cc2)ccn1	6.7	7.2	6.95
425	MolPort-035-701-757	Oc1cc(C2=CC(=O)c3c(O)cc4c([C@@H](c5ccc(OC)c(OCC(OC)=O)c5)CC(O4)=O)c3O2)ccc1OC	6.7	7.2	6.95
426	ZINC04045543	O=C1C(CCC([O-])=O)=C(c2ccc(OCc3cc(cc(c3)C)C)c(c2O1)C)C	6.5	7.4	6.95
427	MolPort-035-701-683	O=C(N1CCN(CC1)C)C[C@H](c2c(O)c(O)c(O)c3C(=O)C=C(Oc32)c4ccccc4)c5cccc(OC)c5	6.8	7.1	6.95
428	ZINC09423424	O=C1c2ccc(OC(=O)c3ccccc3)c(c2O/C1=C/c4cn(c5ccccc45)CC)C	6.8	7.1	6.95
429	SN00073713	O=C1c2ccccc2OC(/N=C(\O)c3ccc(OC)cc3)=C1c4cccc(OC)c4	6.8	7.1	6.95
430	ZINC02281027	Fc1c(F)c(F)c(F)c(COc2ccc3c(c(c(o3)C)C(OC(C)C)=O)c2)c1F	6.8	7.1	6.95
431	ZINC01663395	Cc1cc(nn1CN(Cn2c(cc(n2)C)C)c3ccc(N(Cn4c(cc(n4)C)C)Cn5c(cc(n5)C)C)cc3)C	6.8	7.1	6.95
432	ZINC01463853	Clc1cccc(COc2ccc3c(OC(=C(c4ccc(OC)cc4)C3=O)C)c2)c1	6.8	7.1	6.95
433	SN00173499	O[C@H]1c2cc(OC)ccc2O[C@H]([C@@H]1[C@@H]3COc4c(Cc5ccc(CC)c(O)c5)cc(OC)cc4[C@H]3O)CCCOC	6.8	7.1	6.95
434	MolPort-000-823-233	OC(=O)[C@H](NC(=O)COc1ccc-2c(OC(=O)c3ccccc32)c1C)Cc4c[nH]c5ccccc54	6.6	7.3	6.95

435	ZINC01940964	Clc1c(Cl)ccc(COc2ccc3C(=O)C(Oc4ccc(OC)cc4)=COc3c2)c1	6.6	7.3	6.95
436	MolPort-002-532-272	O=C1[C@]2([C@]3([C@H]([C@H]4CCCN42)C(=O)c5cccc(OC)c5)C(=O)Nc6ccccc63)c7cccc(c7N1)C	7.1	6.7	6.90
437	MolPort-001-737-843	O=C1CN(C(=O)[C@@H]2Cc3c4ccccc4[nH]c3[C@@H](N12)c5cc(OC)c(OC)c(OC)c5)CCc6ccc(OC)c(OC)c6	7.1	6.7	6.90
438	15331	O=C(N1CCCCCC1)[C@@H]2Cc3cn(c4cccc([C@@H]([NH2+]2)CC(C)C)c43)Cc5cc(on5)C	7.1	6.7	6.90
439	MolPort-008-348-482	O=C1COc2ccc(NC(=O)C(=O)NCCc3c[nH]c4ccc(OC)cc43)cc2N1C	7.1	6.7	6.90
440	13183	CC([C@@H]1C[C@@H](Cc2nc3cccc(c3[nH]2)C)C(=C[C@H]1C[NH2+]Cc4cnccc4)C)C	7.0	6.8	6.90
441	ZINC68572030	Brc1cc(Br)cc(-c2nnc(SCC=C)n2[NH3+])c1O	7.0	6.8	6.90
442	ZINC00945051	Clc1cccc(COc2ccc3C(=O)C(Oc4ccc(OC)cc4)=COc3c2)c1	7.0	6.8	6.90
443	189083-79-2	Oc1c(OC)ccc(-c2c3-c4cc(OC)c(O)cc4OC(=O)c3n5CCc6cc(OC)c(OC)cc6-c25)c1	7.0	6.8	6.90
444	ZINC49543314	O=C1C[C@@H](c2c(N1)c(cn2-c3ccc(OC)cc3OC)C([O-])=O)c4ccc(OC)c(OC)c4	6.9	6.9	6.90
445	ZINC62001460	Oc1c2C(OCc2c(c(OC)c1C/C=C(/CCC(=O)Nc3nc(c(s3)C)C)C)C)=O	6.9	6.9	6.90
446	MolPort-002-675-581	Clc1ccc2c(c3CCN[C@@H](c3[nH]2)C4=C(O)NC(=O)N(C4=O)Cc5ccc(OC)cc5)c1	6.9	6.9	6.90
447	ZINC70701314	Clc1ccccc1CN2CC(=O)N3C[C@@H](c4c5ccccc5[nH]c4[C@]3(C2=O)C)c6ccc(OC(C)C)c(OC)c6	6.9	6.9	6.90
448	ZINC02289379	Fc1c(F)c(F)c(F)c(COc2ccc3c(c(o3)C)C(OC)=O)c2)c1F	6.9	6.9	6.90
449	ZINC00323509	O[C@]([C@@H]1Cc2c(OC)c3ccccc3nc2O1)(COC(=O)C)C	6.9	6.9	6.90
450	ZINC02433600	Fc1c(F)c(F)c(F)c(COc2ccc3C(=O)C(c4ccc(OC)c(OC)c4)=C(Oc3c2)C)c1F	6.9	6.9	6.90
451	115982-19-9	Oc1c(OC)c(OC)cc-2c1CCn3c4C(Oc5cc(O)c(OC)cc5-c4c(c23)-c6ccc(OC)c(O)c6)=O	6.9	6.9	6.90
452	ZINC02118774	O=C1C=C(CCC)c2c(O1)c(c3c(CN(c4c(cccc4C(C)C)C)CO3)c2)C	6.9	6.9	6.90
453	131917-09-4	O[C@@H]([C@H](O)[C@H](O)/C=C/C/C=C/C/C=C\CCCC(O)=O)C/C=C/CC	6.8	7.0	6.90
454	ZINC09409633	Brc1ccc(-c2c(nc(nc2-c3ccc(OCC(C)=C)cc3O)N)C(F)(F)F)cc1	6.8	7.0	6.90
455	MolPort-002-516-123	O=C(N[C@@H](Cc1c[nH]c2ccccc21)C(O)=O)COc3cc(cc4c3C5=C(CCCC5)C(O4)=O)C	6.8	7.0	6.90
456	ZINC40312612	O=C1C[C@H](c2c(N1)c(cn2-c3ccc(OC)cc3)C([O-])=O)c4ccc(OC)c(OC)c4OC	6.7	7.1	6.90
457	ZINC11867078	O[C@]12CCN(C[C@H]2CCCC1)C(=O)CC=3C(Oc4c(c(OCc5cc(cc(c5)C)C)ccc4C3C)C)=O	6.7	7.1	6.90
458	ZINC02125245	O=C1C=C(c2ccc3c(CN(CO3)c4c(C)ccc(c4)C)c2O1)CC	6.7	7.1	6.90
459	MolPort-023-275-183	Fc1ccccc1CN[C@@H]2CO[C@H]([C@H]2O)CNS(=O)(=O)c3ccc(cc3)C	6.6	7.2	6.90
460	ZINC02091635	O=C1C(=C(c2ccc(OCc3cc(cc(c3)C)C)c(c2O1)C)C)CC(OC)=O	7.1	6.6	6.85
461	ZINC01799114	Clc1c(Cl)ccc(COc2ccc3C=C(c4nc5ccccc5s4)C(Oc3c2)=O)c1	7.1	6.6	6.85
462	MolPort-009-759-056	O=C(NCCN1C(=Nc2ccccc2C1=O)C)CCCc3c[nH]c4ccccc43	6.9	6.8	6.85
463	ZINC04222564	Fc1cc(F)cc(CNC(=O)C[C@@H]2c3c(sc(n3)NCC=C)C[C@H]4[C@]([C@@H](O)CC[C@]24C)(CO)C)c1	6.9	6.8	6.85
464	MolPort-002-522-484	O=C(N[C@H](Cc1c[nH]c2ccccc21)C(O)=O)CCC=3C(Oc4c(c5c(c6c(o5)CCCC6)cc4C3C)C)=O	6.9	6.8	6.85
465	SN00267894	O=C1c2ccc3c(c2OC(c4cc(OC)cc(OC)c4)=C1OC)cco3	6.9	6.8	6.85
466	ZINC08791835	O=C(N[C@H]([C@@H](CC)C)C(=O)NCCCOC)[C@H]1C(S[C@@H]2c3ccccc3C(=O)N21)(C)C	6.9	6.8	6.85
467	ZINC20466689	O[C@H]1[C@@H](CC[C@]2(CC[C@H](NC(=O)C3CC3)[C@@H]([C@@H]12)C)C)[C@@H](C(=O)N4CCCC4)C	6.9	6.8	6.85
468	SN00073847	[O+]=C1c2ccccc2OC(NC(=O)c3ccco3)=C1c4ccc(OC)c(OC)c4	6.9	6.8	6.85
469	ZINC38564389	O=C1CC[C@@]2([C@@H](C1(C)C)CC[C@@]3([C@H]2C(=O)C=C4[C@]3(CCC5(CC[C@](C[C@H]45)(C)C(OC)=O)C)C)C)C	6.8	6.9	6.85
470	7338	O=C(N[C@H]1C[C@H]([NH+](C1)C)c2nc(no2)-c3ccc4ccccc4c3)c5ccccn5	6.8	6.9	6.85
471	ZINC02150013	Fc1cccc(COc2ccc3c(OC(=O)C(=C3C)CCC(OCC)=O)c2)c1	6.8	6.9	6.85
472	ZINC20411593	OC=1C(=O)N(CCN(C)C)[C@@H](c2c(OC)ccc(OC)c2)C1C(=O)c3cc4cccc(OC)c4o3	6.8	6.9	6.85
473	MolPort-007-673-275	Clc1ccc(CCNC(=O)c2nccn2CC(=O)c3ccc(OC)cc3)cc1	6.8	6.9	6.85
474	ZINC01648045	Brc1ccc2c(c3c([nH]2)C(=O)N(CC3)Cc4ccc(Cl)cc4)c1	6.8	6.9	6.85
475	ZINC02442084	Clc1cccc(COc2ccc3C(=O)C(Oc4ccccc4OC)=C(Oc3c2)C)c1	6.8	6.9	6.85
476	6277	Clc1c(F)ccc(C(=O)N[C@@H]2C=C[C@H](c3c(nn(Cc4ccccc4)c3C)C)C2)c1	6.7	7.0	6.85
477	ZINC13729784	O=C1c2ccccc2N=CN1CC(=O)Nc3ccc4c(c(o4)C(=O)c5ccccc5)C)c3	6.7	7.0	6.85
478	MolPort-028-856-572	O=C(c1ccc(OC)c(OC)c1)COc2ccc3c(c(-c4cc(OC)c(OC)c(OC)c4)co3)c2	6.7	7.0	6.85
479	ZINC18046579	FC(F)(F)c1c(c([nH]n1)-c2ccc(OCC(=O)N[NH3+])cc2O)-c3ccc(OC)cc3	6.7	7.0	6.85

480	SN00147976	FC(F)(F)c1c(-c2ccc(OC)cc2)c(on1)-c3ccc(OCC)cc3O	6.7	7.0	6.85
481	SN00283805	O=C(c1c(OC)cc(OC)cc1OC)/C=C/c2cc(OC)c(OC)cc2OC	6.7	7.0	6.85
482	163768-83-0	Clc1c(c(Cl)c(O)c(C(=O)c2c(c(O)cc(O)c2CC=C(C)C)C=O)c1O)C	6.7	7.0	6.85
483	ZINC40309002	Brc1cccc2c1ccn2CCC(=O)N[C@H]([C@H](CC)C)C([O-])=O	6.7	7.0	6.85
484	SN00084157	O=C1C(=O)[C@H-]([C@@H](N1CCOC)c2ccc(O)c(OC)c2)C(=O)c3ccc(o3)C	6.7	7.0	6.85
485	ZINC02092165	Fc1cccc(COc2ccc3c(OC(=O)C(=C3C)CCC(OCC)=O)c2C)c1	6.6	7.1	6.85
486	ZINC02151660	O=C1C(=C(c2ccc(OCc3cccc(c3)C)c(c2O1)C)C)CC([O-])=O	6.5	7.2	6.85
487	ZINC06407719	Oc1cc(OC)cc(OC)c1C(=O)/C=C/c2ccccc2OC	6.7	6.9	6.80
488	ZINC31157673	Oc1c(c(cc(O)c1Cc2c(O)cc(c(c2O)C=O)C)C)C	6.7	6.9	6.80
489	MolPort-005-950-245	O=C(N1Cc2c([nH]cn2)C[C@H]1c3nc(no3)-c4ccc5ccccc5c4)Cc6ccc(OC)cc6	6.7	6.9	6.80
490	SN00145601	Brc1cc2c(c(c(o2)C)C(OCCOC)=O)cc1OCc3cccc([N+]([O-])=O)c3	6.7	6.9	6.80
491	ZINC40313355	O=C(c1c2ccccc2n(c1C)C)CN3C(=O)C=CC(=N3)c4ccco4	6.7	6.9	6.80
492	ZINC11866426	O=C(Nc1cc(ccc1OC)C)c2c(oc3ccc(OC)cc23)C	6.7	6.9	6.80
493	MolPort-002-535-447	O=C1c2c(OC)c(OC)ccc2-c3c4c(c5cc(O[C@H](C(=O)Nc6ccc(OC)c(OC)c6)C)ccc5n14)ccn3	6.7	6.9	6.80
494	ZINC02319078	Fc1c(F)c(F)c(F)c(F)c1OCC(Oc2ccc3c(c(c(o3)C)C(OC)=O)c2)=O	6.7	6.9	6.80
495	ZINC09410228	Clc1cc(F)ccc1COc2ccc(-c3c(-c4ccc(OC)cc4)c(no3)C)c(O)c2	6.7	6.9	6.80
496	ZINC15674316	Fc1c(C)ccc(-c2noc(n2)[C@@H]3C[C@@H](O)C[NH+]3Cc4c[nH]c5ccccc54)c1	6.8	6.8	6.80
497	ZINC02122012	O=C1C=C(CCC)c2c(O1)c(c3c(CN(CO3)c4c(C)ccc(c4)C)c2)C	6.8	6.8	6.80
498	ZINC08740407	Brc1ccc(OCc2ccccn2)c([C@@H]3c4c(C[C@@H](N3)C(O)=O)c5ccccc5[nH]4)c1	6.8	6.8	6.80
499	SN00147906	FC(F)(F)c1c(c(on1)-c2ccc(O)cc2O)-c3ccc(OC)cc3	6.6	7.0	6.80
500	ZINC03846578	O=C(C[C@@H](Nc1ccc(N)cc1)c2ccc3c(OCO3)c2)C=4C(=O)c5ccccc5N(C4[O-])C	6.6	7.0	6.80
501	SN00290874	Oc1c(OC)cc2CCN([C@H]3Cc4ccc(O)c(c4)-c5c(OC)ccc(CC6c7cc(Oc1c23)c(OC)cc7CCN6)c5)C	6.6	7.0	6.80
502	ZINC02126974	O=C(NCCc1ccc(OC)cc1)c2cc3c4ccccc4[nH]c3c(n2)-c5cc(OC)ccc5OC	6.6	7.0	6.80
503	ZINC05389290	Oc1cc(OCC)ccc1-c2c(-c3ccc(OC)c(OC)c3)c(no2)C	6.5	7.1	6.80
504	MolPort-002-535-319	O=C(NCCc1ccc(OC)cc1)[C@@H](Oc2ccc3c(c4ccnc5-c6ccccc6C(=O)n3c54)c2)C	6.5	7.1	6.80
505	ZINC35466154	O[C@H]([C@H](CCC/C(=C/Cc1c(OC)c(c2COC(=O)c2c1O)C)C)C)CC([O-])=O	6.5	7.1	6.80
506	ZINC31164529	O[C@@H](C/C=C(/CCC=C(C)C)C)/C(=C/Cc1c(O)ccc(c1)C([O-])=O)C	6.5	7.1	6.80
507	MolPort-005-948-910	O[C@@H]1C[C@H](N(C1)Cc2ccc(OC)cc2)c3nc(no3)CS(=O)(=O)c4ccc(cc4)C	6.5	7.1	6.80
508	ZINC13623573	Fc1ccc(C(=O)Nc2c3ccccc3oc2C4=CC(Oc5ccc(C(C)C)cc54)=O)cc1	6.9	6.6	6.75
509	MolPort-002-526-623	Clc1cccc(CNC(=O)c2cc3c4ccccc4[nH]c3c(n2)-c5ccc(OCC)c(OC)c5)c1	6.9	6.6	6.75
510	ZINC02200212	Fc1c(F)c(F)c(F)c(COc2cc3c(cc2CC)C(=O)C(c4ccc5c(OCCO5)c4)=C(O3)C)c1F	6.9	6.6	6.75
511	ZINC01799112	Clc1cccc(COc2ccc3C=C(c4nc5ccccc5s4)C(Oc3c2)=O)c1	6.9	6.6	6.75
512	ZINC02153244	O=C1C2=C([C@H](N1CCc3ccc(OC)c(OC)c3)c4ccc(O)c(OC)c4)C(=O)c5ccccc5O2	6.8	6.7	6.75
513	SN00017085	O=S(=O)(NC[C@@H]1C[C@@H]2CC[NH+]1C[C@H]2c3cc(nn3C)-c4ccc(OC)c(OC)c4)Cc5ccccc5	6.8	6.7	6.75
514	MolPort-009-649-082	O=C1C[C@H](c2c(N1)c(cn2-c3ccc(OC)cc3OC)C(O)=O)c4ccc(OC)c(O)c4	6.8	6.7	6.75
515	MolPort-002-516-371	S=C1N(C(=O)C([C@@H]2c3cc(OC)c(OC)cc3CCN2C(=O)C)=C(O)N1c4ccccc4)c5ccccc5	6.7	6.8	6.75
516	ZINC04237030	O[C@H]1CCC2([C@@H](c3c(sc(n3)NC(=O)CCCCC)C[C@H]2[C@@]1(CO)C)CC(=O)N(CC=C)C)C	6.7	6.8	6.75
517	ZINC15676415	O=S(=O)(N1Cc2c(ncn2Cc3ccc(OC)cc3)C[C@H]1C(OC)=O)c4cccc5cccnc54	6.7	6.8	6.75
518	ZINC02118808	O=C1C(=C(c2cc3c(OCN(C3)c4ccccc4CC)c(c2O1)C)C)C	6.7	6.8	6.75
519	ZINC01799111	Fc1c(F)c(F)c(F)c(F)c1OCC(Oc2ccc3c(c(o3)C)C(OC(C)C)=O)c2)=O	6.7	6.8	6.75
520	SN00332856	O=C1C=Cc2c(O1)cc(OC)c([C@H]3C=C(CC[C@]3(/C=C/c4c(OC)cc5c(c4)C=CC(O5)=O)C)C)c2	6.7	6.8	6.75
521	ZINC02667598	Clc1c(Cl)ccc(COc2ccc3C(=O)C(c4ccc(OC)c(OC)c4)=C(Oc3c2)C)c1	6.7	6.8	6.75
522	ZINC12860017	S=C(N(CCc1cc2c(OCO2)c(OC)c1/C=C/3C(=O)NC(=O)N(CCCCCC)C3=O)C)NC	6.7	6.8	6.75
523	20464	O[C@H]1[C@@H](CC[C@]2(CC[C@H](NC(=O)COC)[C@H]([C@@H]12)C)C)[C@H](C(=O)N(Cc3ccccc3)C)C	6.6	6.9	6.75
524	ZINC68575016	FC(F)(F)C(F)(C(=O)Nc1cc(ccc1OC)C(F)(F)F)C(F)(F)F	6.6	6.9	6.75
525	ZINC68574667	FC(F)(O[C@](F)(C(=O)Nc1cc(ccc1OC)C(F)(F)F)C(F)(F)F)C(F)(F)C(F)(F)F	6.6	6.9	6.75

526	SN00148474	FC(F)(F)c1c(-c2ccc(OC)cc2)c(on1)-c3ccc(OCc4ccc([N+]([O-])=O)cc4)cc3O	6.6	6.9	6.75
527	ZINC04270713	Fc1cccc(C(=O)N2C[C@H](N(S(=O)(=O)C)CC2)C(OC)=O)c1	6.6	6.9	6.75
528	ZINC70707164	Fc1ccc(CNC(=O)c2ccccc2N3C(=O)N4[C@@H](c5c(C[C@H]4C3=O)c6ccccc6[nH]5)c7ccc(OC)cc7)c c1	6.6	6.9	6.75
529	ZINC68589892	O=C1C(=C([O-])N(c2ccccc12)c3ccccc3)/C(=N/c4ccc(O)cc4C)C	6.6	6.9	6.75
530	MolPort-044-810-768	Brc1c2c(OCO2)c(OC)c3C=C(N(CCc13)C)C4=CC(Oc5ccc(OC)cc54)=O	6.6	6.9	6.75
531	19205	Fc1ccc(C(=O)NCC2(C[C@H](O)[C@@H](O)C2)COC)cc1	6.5	7.0	6.75
532	ZINC04222564	Fc1cc(F)cc(CNC(=O)C[C@@H]2c3c(sc(n3)NCC=C)C[C@H]4[C@]([C@@H](O)CCC24C)(CO)C)c1	6.5	7.0	6.75
533	MolPort-035-700-267	O=C(N1CCN(CC1)C)C[C@@H](c2c(O)c(O)c(O)c3C(=O)C=C(Oc32)c4ccccc4)c5ccc(OC)c(OC)c5	6.5	7.0	6.75
534	MolPort-002-511-055	Nc1c(c(nc(n1)Cc2ccc(OC)c(OC)c2)Cc3ccc(OC)c(OC)c3)-c4ccc(OC)c(OC)c4	6.5	7.0	6.75
535	ZINC02135653	O=C1C(=C(c2ccc(OC)c(OC)c2O1)C)CC(=O)N[C@@H](Cc3c[nH]c4ccc(O)cc43)C([O-])=O	6.5	7.0	6.75
536	MolPort-000-487-182	O=C1C2=C(OC(N)=C([C@H]2c3ccc(OC)c(OC)c3OC)C#N)C=C(N1Cc4ccc(OC)c(OC)c4)C	6.4	7.1	6.75
537	MolPort-028-856-091	O=C(N1CCN(CC1)c2cccc(OC)c2)Cn3c4ccccc4c5CCN6C(=O)c7ccccc7N=C6c53	6.9	6.5	6.70
538	ZINC02666313	Clc1cccc(COc2ccc3C(=O)C(Oc4ccc(cc4)C(OCC)=O)=COc3c2)c1	6.9	6.5	6.70
539	MolPort-035-700-717	O=C(c1ccc(OC)c(OC)c1)COc2ccc3c(c(-c4ccc(OC)c(OC)c4)co3)c2	6.7	6.7	6.70
540	MolPort-002-528-533	O=C(N[C@@H](Cc1c[nH]c2ccc(O)cc21)C(O)=O)COc3ccc4C(CCC)=CC(Oc4c3C)=O	6.7	6.7	6.70
541	MolPort-035-699-980	O=C(N1CCc2c([nH]c3ccccc32)C1)C[C@@H](C4=COc5ccc(OC)cc5C4=O)C6=C(O)C(=O)C=C(O6) COC	6.7	6.7	6.70
542	MolPort-021-746-036	Fc1ccc2c(c3CN(CCc3[nH]2)C(=O)COc4ccc5c(OC(=O)C(=C5C)C)c4)c1	6.7	6.7	6.70
543	SN00075071	O=C1C(C=2C[C@@H](Sc3ccccc3N2)c4cccc(OC)c4)=C([O-])C=C(O1)C	6.5	6.9	6.70
544	ZINC05163437	FC(F)(F)c1c(-c2ccc(OC)cc2)c(n[nH]1)-c3ccc(OCC)cc3O	6.5	6.9	6.70
545	MolPort-028-856-192	O=C1C([C@@H](CC(OC)=O)c2cc(OC)c(O)c(OC)c2)=C(NN1)Cc3ccc(OC)cc3	6.5	6.9	6.70
546	18636	O=C(N1CCOC[C@H](Oc2cccc(n2)C)C1)c3cc(nn3C)C	6.5	6.9	6.70
547	MolPort-044-811-170	Brc1c2c(OCO2)c(OC)c3C=C(N(CCc13)C)C4=CC(Oc5ccc(cc54)C)=O	6.5	6.9	6.70
548	200936-84-1	O[C@H](CCCCCC(=O)C=1C(O[C@]2(C1C(=C3C=C(OC=C3[C@@H]2O)C)C=O)C)=O)C	6.4	7.0	6.70
549	SN00306304	O=C1[C@H](OC(=O)C)C[C@]2([C@@H](C(CC[C@@H]2C1(C)C)=C)COc3c(OC)cc4C=CC(Oc4c3 OC)=O)C	6.4	7.0	6.70
550	ZINC02122747	O=C1C(=C(c2cc3c(OCN(c4c(cc(cc4C)C)C)C3)c(c2O1)C)C)Cc5ccccc5	6.4	7.0	6.70
551	ZINC04267149	Clc1ccc([C@@H]([NH2+]C=2C(=O)N(N(C2C)C)c3ccccc3)CC(=O)C=4C(=O)N(c5ccccc5C4O)C)cc1	6.4	7.0	6.70
552	ZINC08918342	Clc1cccc(COc2ccc3C(=C(CCC(=O)N4CC[C@]5(O)CCCC[C@H]5C4)C(Oc3c2C)=O)C)c1	6.8	6.6	6.70
553	ZINC02146856	O=C(c1c2cc(OC)ccc2n(c1C)C)COc3ccc4-c5ccc(OC)cc5C(Oc4c3C)=O	6.8	6.6	6.70
554	MolPort-000-846-216	O=C(NCCc1c[nH]c2cc(OC)ccc21)CC=3C(Oc4cc5c(c(c(o5)C)C)cc4C3C)=O	6.8	6.6	6.70
555	ZINC02094262	O=C1c2c(OC(=C1c3ccc4c(OCCO4)c3)C)cc(OCc5ccc(cc5)C)c(CC)c2	6.8	6.6	6.70
556	MolPort-002-518-378	O=S(=O)(N[C@@H](CC(C)C)C(Oc1ccc2-c3ccccc3C(Oc2c1C)=O)=O)c4ccc(cc4)C	6.6	6.8	6.70
557	ZINC09033642	O[C@H](CCCCC)/C=C/[C@@H]1CCC(=O)[C@@H]1CCCCCCC(=O)NCc2cccc(OC)c2OC	6.6	6.8	6.70
558	MolPort-002-524-112	O=C(NCc1ccccc1OC)c2cc3c4ccccc4[nH]c3c(n2)-c5cccc(OC)c5	6.6	6.8	6.70
559	ZINC06755168	FC(F)(F)c1c(c(on1)-c2ccc(O)cc2O)-c3ccc(OC)c(OC)c3	6.6	6.8	6.70
560	ZINC20467205	O[C@H]1[C@H](CC[C@]2(CC[C@H](NC(=O)c3ccccn3)[C@@H]([C@H]12)C)C)[C@@H](C(=O) N4CCCC4)C	6.6	6.8	6.70
561	ZINC00487450	O=C1C(Oc2ccccc2OC)=COc3c1ccc(OC(=O)C)c3C	6.6	6.8	6.70
562	MolPort-035-701-927	Oc1cc(C2=CC(=O)c3c(O)cc4c([C@H](CC(O4)=O)c5ccc(OC)c(OC(C)C)c5)c3O2)ccc1OC	6.6	6.8	6.70
563	ZINC11865237	Clc1cccc(COc2ccc3C(=O)C(Oc4cccc(OC)c4)=C(Oc3c2)C)c1	6.6	6.8	6.70
564	ZINC03851657	Cc1ccc(N2/C(SC(OC)=N2)=C/C=3c4cc(OC)c(OC)cc4CCN3)cc1	6.6	6.8	6.70
565	149355-75-9	Oc1cc2c(-c3c(n4CCc5c(OC)c(OC)c(OC)cc5-c4c3-c6ccc(OC)c(OC)c6)C(O2)=O)cc1OC	6.6	6.8	6.70
566	ZINC12892682	O=C(N[C@@H](Cc1c[nH]c2ccccc21)C)CC=3C(Oc4c(c5c(cc4C3C)c(co5)C)C)=O	6.9	6.4	6.65
567	ZINC02149868	Clc1cc2c(OC(=O)C(=C2C)C)cc1OC(=O)[C@H](NS(=O)(=O)c3ccc(cc3)C)[C@H](CC)C	6.8	6.5	6.65
568	ZINC13609475	Clc1c(Cl)cc(COc2ccc3c(OC([NH3+])=C(c4ccc(OC)cc4)C3=O)c2)cc1	6.8	6.5	6.65
569	SN00241623	Oc1c(OC)cc2c3c1Oc4c(OC)cc5CCN[C@@H](c5c4)Cc6ccc(Oc7c(OC)ccc(C[C@@H]2N(CC3)C)c7)c	6.7	6.6	6.65

		c6			
570	SN00101930	Clc1ccc([C@H]([NH2+]CC[C@H](c2ccc(OC(C)C)cc2)c3ccccc3OC)C)cc1	6.7	6.6	6.65
571	MolPort-028-856-171	O=C(NCCN(C)C)Cn1c2ccccc2c3CCN4C(=O)c5ccccc5N=C4c31	6.7	6.6	6.65
572	ZINC02667544	Brc1ccc(C2=COc3cc(OCc4cccc(Cl)c4)ccc3C2=O)cc1	6.7	6.6	6.65
573	ZINC02217015	FC(F)(F)C(NC(=O)NC=1C(=O)N(N(C1C)C)c2ccccc2)(C(F)(F)F)C	6.7	6.6	6.65
574	ZINC12603403	Fc1ccc(-n2c(c([C@H]3C=C[C@H]([NH2+]Cc4ccco4)C3)c(n2)C)C)cc1	6.6	6.7	6.65
575	ZINC02152082	Fc1cccc(COc2ccc3c(OC(=O)C(=C3C)CC(OC)=O)c2)c1	6.6	6.7	6.65
576	ZINC03846418	O=C(O[C@H]1C[C@H](CC[C@H]1C(C)C)C)C(CCC)(C)C(=O)C	6.6	6.7	6.65
577	MolPort-002-518-659	O=C(Nc1ccc2c(c3ccccc3n2CC)c1)COc4ccc5c(OC(=O)C=C5c6ccccc6)c4	6.6	6.7	6.65
578	143682-17-1	Oc1cccc2c3c4CNC(=O)c4c5c6ccccc6n7c5c3n([C@]8([C@H](OC)[C@@H](NC)C[C@H]7O8)C)c12	6.5	6.8	6.65
579	ZINC20728784	Clc1cccc(COc2ccc3c(noc3c2)Cc4ccc(Cl)cc4)c1	6.5	6.8	6.65
580	ZINC04023868	O[C@]1(CC[C@H]2C(CCC[C@]2([C@@H]1CC[C@@](O)(CC)C)C)(C)C)C	6.5	6.8	6.65
581	SN00052039	Brc1cccc(N2C(=O)c3ccccc3N4C(=O)c5c(OC)c(OC)ccc5[C@@H]24)c1	6.5	6.8	6.65
582	MolPort-002-518-297	O=C(N1Cc2c(C[C@H]1C(OC)=O)c3ccccc3[nH]2)COc4ccc5c(OC(=O)C(CCCCCC)=C5C)c4	6.5	6.8	6.65
583	121819-68-9	O=C1C(Nc2nc(c(n2C)Cc3ccc(OC)c(O)c3)Cc4ccc(OC)cc4)=NC(=O)N1C	6.4	6.9	6.65
584	SN00074949	Oc1ccc([C@H]2CC(=Nc3ccccc3S2)C=4C(OC(=CC4[O-])C)=O)cc1OC	6.4	6.9	6.65
585	SN00049419	O=C1C2=C(N(C(=O)N1C)C)N(C=3c4ccccc4C(=O)C3[C@H]2c5ccc(OC(=O)C)c(OC)c5)C(=O)C	6.4	6.9	6.65
586	MolPort-002-533-130	O=C1c2ccccc2OC(NC(=O)c3cccc(OC)c3)=C1c4ccc(OC)c(OC)c4	6.3	7.0	6.65
587	ZINC11867349	Clc1c(Cl)ccc(COc2ccc3C(=C(CC(=O)N4CC[C@@]5(O)CCCC[C@@H]5C4)C(Oc3c2C)=O)C)c1	6.7	6.5	6.60
588	ZINC11867170	O[C@]12CCN(C[C@H]2CCCC1)C(=O)CC=3C(Oc4c(c(OCc5cccc(c5)C)ccc4C3C)C)=O	6.6	6.6	6.60
589	MolPort-028-855-419	O=C(N1CCN(CC1)c2ccccc2OC)Cn3c4ccccc4c5CCN6C(=O)c7ccccc7N=C6c53	6.6	6.6	6.60
590	ZINC02124948	O=C1C=C(CC)c2c(O1)c(c3c(CN(CO3)c4c(C)ccc(c4)C)c2)C	6.6	6.6	6.60
591	ZINC04252491	Oc1cc(OC)cc(OC)c1C(=O)/C=C/c2ccc(OCc3ccccc3)c(OC)c2	6.6	6.6	6.60
592	MolPort-002-151-076	O=C1c2ccccc2C=3[C@@H]1[C@@H](c4ccc(OC)c(OC)c4)C=5C(=O)N(C(=O)N(C5N3)C)C	6.5	6.7	6.60
593	ZINC05389044	FC(F)(F)c1c(c(on1)-c2ccc(OC)cc2O)-c3ccc(OC)cc3	6.5	6.7	6.60
594	ZINC02159827	O=C1C=C(CCCC)c2c(O1)c(c3c(CN(CO3)c4cccc(c4C)C)c2)C	6.5	6.7	6.60
595	ZINC02093059	O=C(OCC)CCC=1C(Oc2c(c(OCc3cc(cc(c3)C)C)ccc2C1C)C)=O	6.5	6.7	6.60
596	ZINC04046749	O=C1C(=C(c2ccc(OCc3cccc(c3)C)cc2O1)C)CC([O-])=O	6.5	6.7	6.60
597	SN00050294	Fc1ccc(C(=O)Nc2cc(OC)c(OC)cc2Cc3c4cc(OC)c(OC)cc4ccn3)cc1	6.5	6.7	6.60
598	SN00348754	O=C1C=Cc2ccc(OC)c([C@H]3C=C(CC[C@@]3(C)/C=C/c4c(OC)ccc5C=CC(Oc54)=O)C)c2O1	6.5	6.7	6.60
599	ZINC02122508	O=C1C=C(CCCC)c2c(O1)c(c3c(CN(CO3)c4c(C)ccc(c4)C)c2)C	6.5	6.7	6.60
600	SN00151021	Clc1ccccc1COc2cc(OC)ccc2-c3c(-c4ccc(OC)cc4)c(no3)C	6.5	6.7	6.60
601	ZINC20762168	O=C(N1CCc2c([nH]cn2)[C@H]1c3c(OC)ccc(OC)c3)N[C@H](C)C(OC)=O	6.4	6.8	6.60
602	ZINC79189259	O=C(OCC)[C@@H]1[C@@H](c2ccc(OC)c3ccccc32)C=4C(Oc5ccccc5C4O1)=O	6.4	6.8	6.60
603	ZINC02094763	Fc1cccc(COc2ccc3c(OC(=O)C(=C3C)CC(OC)=O)c2C)c1	6.4	6.8	6.60
604	ZINC02148915	O=C1C(CCC([O-])=O)=C(c2ccc(OCc3cc(cc(c3)C)C)cc2O1)C	6.4	6.8	6.60
605	ZINC02133746	O=C1C=C(c2ccc(OC/C=C(/CCC=C(C)C)C)cc2O1)C	6.4	6.8	6.60
606	ZINC03846610	O=C(C[C@H](Nc1cnccc1)c2ccc(N(C)C)cc2)C=3C(=O)c4ccccc4N(C3[O-])C	6.4	6.8	6.60
607	MolPort-027-852-780	Fc1ccc(-c2c([C@@H](NC(=O)c3c(nn(n3)-c4ccccc4)C)C)c(on2)C(O)=O)cc1	6.4	6.8	6.60
608	ZINC01444304	O=C(N1c2ccccc2CO[C@@H]1c3ccc(OC)cc3)c4cccc(OC)c4	6.4	6.8	6.60
609	ZINC02333037	Clc1cccc(COc2ccc3C(=O)C(Oc4ccccc4OCC)=COc3c2)c1	6.4	6.8	6.60
610	ZINC14492226	O[C@H](C=C(C)C)C(=O)/C(=C/CC/C(C)=C/CC=1C(Oc2cccc(c2C1[O-])C)=O)C	6.3	6.9	6.60
611	ZINC02118316	O=C1C=2CCCC2c3cc4c(OCN(C4)c5ccccc5C)c(c3O1)C	6.3	6.9	6.60
612	MolPort-028-856-432	Oc1cc(O)c2C(=O)C=C(Oc2c1[C@@H](c3ccc4c(nccn4)c3)CC(OC)=O)c5ccccc5	6.3	6.9	6.60
613	ZINC02092152	O=S(=O)(N[C@@H](CCCC)C(Oc1cc(cc2c1C(=C(C(O2)=O)C)C)C)=O)c3ccc(cc3)C	6.3	6.9	6.60
614	SN00084180	O=C1C(=O)[C@H-]([C@@H](N1CCOC)c2cc(OC)c(OC)c(OC)c2)C(=O)c3ccco3	6.3	6.9	6.60
615	SN00077068	Oc1ccc(OC)cc1[C@H]2CC(=Nc3ccccc3S2)C=4C(OC(=CC4[O-])C)=O	6.2	7.0	6.60

616	ZINC02277837	Fc1c(F)c(F)c(F)c(COc2ccc3c(c(c(o3)C)C(OCCOC)=O)c2)c1F	6.2	7.0	6.60
617	ZINC68591828	Oc1c(OC)cc([C@@H]2CC(=Nc3ccccc3S2)C=4C(=O)c5ccccc5N(C4[O-])C)cc1OC	6.2	7.0	6.60
618	ZINC38143767	O[C@@H]1C[C@@]2([C@@]([C@H]1[C@](O)(C(=O)CCC(O)(C)C)C)(CC(=O)[C@]3([C@@H]4C[C@H](O)C(=O)C(C4=CC[C@H]32)(C)C)C)C)C	6.7	6.4	6.55
619	SN00048103	Oc1cc(OC)c(CCC)cc1-c2c(-c3ccc4c(OCCCO4)c3)c(no2)C	6.4	6.7	6.55
620	20512	Fc1cc(F)cc(CNC(=O)[C@H]([C@H]2CC[C@]3(CC[C@H](NC(=O)Cc4ccccc4)[C@@H]([C@H]3[C@@H]2O)C)C)C)c1	6.4	6.7	6.55
621	ZINC02094396	Fc1cccc(COc2ccc3c(OC(=O)C(=C3C)CCC([O-])=O)c2C)c1	6.4	6.7	6.55
622	ZINC02148668	O=S(=O)(N[C@@H]([C@@H](CC)C)C(Oc1cc(cc2c1C(CCCC)=CC(O2)=O)C)=O)c3ccc(cc3)C	6.4	6.7	6.55
623	ZINC02147761	O=S(=O)(N[C@@H](CC(C)C)C(Oc1ccc2c(OC(=O)C(=C2C)C)c1)=O)c3ccc(cc3)C	6.4	6.7	6.55
624	ZINC02109980	O=C(c1c2cc(OC)ccc2n(c1C)C)COc3cc4c(C5=C(CCC5)C(O4)=O)cc3CCCCCC	6.4	6.7	6.55
625	ZINC02666276	Clc1c(Cl)ccc(COc2ccc3C(=O)C(c4ccccc4)=C(Oc3c2)C)c1	6.4	6.7	6.55
626	10245-81-5	Brc1c(Br)c(Br)[nH]c1-c2cc(Br)cc(Br)c2O	6.4	6.7	6.55
627	SN00267570	O=C([C@@H]1[C@@H]([C@@H]([C@H]1C(=O)c2c(O)cc(O)cc2OC)c3ccc(OC)cc3)c4ccc(OC)cc4)c5c(O)cc(O)cc5OC	6.4	6.7	6.55
628	ZINC12530252	Fc1ccc(C(=O)N2Cc3c(ncn3Cc4ccccc4)C[C@H]2C(OC)=O)cc1	6.6	6.5	6.55
629	MolPort-002-527-363	O=C(NCc1cnccc1)c2cc3c4ccccc4[nH]c3c(n2)-c5cccc(OC)c5	6.6	6.5	6.55
630	115982-21-3	Oc1c(OC)cc-2c(CCn3c4C(Oc5cc(OC)c(O)cc5-c4c(c23)-c6ccc(OC)c(O)c6)=O)c1	6.6	6.5	6.55
631	115982-20-2	Oc1c(OC)c(OC)cc-2c1CCn3c4C(Oc5cc(O)c(OC)cc5-c4c(c23)-c6ccc(OC)c(OC)c6)=O	6.6	6.5	6.55
632	MolPort-008-347-150	O=C(NCCc1c[nH]c2ccc(OC)cc21)Cn3ccc4cc(OC)ccc43	6.5	6.6	6.55
633	ZINC48057066	Oc1cccc(OC)c1C(=O)/C=C/c2ccccc2OC	6.5	6.6	6.55
634	7138	Fc1ccc(-c2noc(n2)[C@@H]3C[C@@H](O)C[NH+]3Cc4ccc(OC)c5ccccc54)cc1	6.5	6.6	6.55
635	12504	Fc1cccc(NC(=O)NC[C@@H]2C=C([C@H](C[C@H]2C(C)C)CC(=O)N(Cc3cnccc3)C)C)c1	6.5	6.6	6.55
636	ZINC20466733	Fc1cccc(C(=O)N[C@H]2CC[C@@]3(CC[C@@H]([C@@H](O)[C@@H]3[C@H]2C)[C@@H](C(=O)N4CCCC4)C)C)c1	6.5	6.6	6.55
637	MolPort-002-525-564	O=C(NCc1ccc(cc1)C)c2cc3c4ccccc4[nH]c3c(n2)-c5cc(OC)c(OC)c(OC)c5	6.5	6.6	6.55
638	ZINC02148701	O=C(OC(C)(C)C)N[C@@H](Cc1c[nH]c2ccccc21)C(Oc3ccc4C(c5ccc(OC)cc5)=CC(Oc4c3C)=O)=O	6.5	6.6	6.55
639	MolPort-001-683-717	O=C(OC(C)(C)C)N[C@@H](Cc1c[nH]c2ccccc21)C(Oc3ccc4c(OC(=O)C(Cc5ccccc5)=C4C)c3)=O	6.3	6.8	6.55
640	MolPort-009-649-155	O=C1C[C@H](c2c(N1)c(cn2-c3ccc(OC)cc3OC)C(O)=O)c4ccc(OC)cc4OC	6.3	6.8	6.55
641	ZINC02124986	O=C1C(=C(c2cc3c(OCN(C3)c4cccc(c4C)C)c(c2O1)C)C)C	6.3	6.8	6.55
642	MolPort-002-520-410	O=C(N[C@@H](Cc1c[nH]c2ccccc21)C(O)=O)[C@H](Oc3ccc4c(OC(=O)C=C4CCCC)c3C)C	6.1	7.0	6.55
643	ZINC02423589	Clc1cccc(COc2ccc3C(=O)C(c4ccccc4)=COc3c2)c1	6.6	6.4	6.50
644	ZINC20467109	O[C@H]1[C@@H](CC[C@]2(CC[C@H](NC(=O)CCOC)[C@H]([C@H]12)C)C)[C@H](C(=O)N(CCC#N)C)C	6.5	6.5	6.50
645	MolPort-042-647-992	Clc1ccc(C(=O)c2ccc3c([C@H](c4cccc(OC)c4OCCc5ccc(OC)cc5)CC(O3)=O)c2O)cc1	6.5	6.5	6.50
646	ZINC02424621	Clc1cccc(COc2ccc3C(=O)C(c4ccccc4)=C(Oc3c2)C)c1	6.5	6.5	6.50
647	ZINC11865561	O=C(NCCCCCC([O-])=O)CCC=1C(Oc2c(C1C)cc3c(occ3C(C)(C)C)c2C)=O	6.4	6.6	6.50
648	ZINC09233757	O=C(N1c2ccccc2NC=3C[C@@H](CC(=O)C3[C@@H]1c4ccc(cc4)C(OC)=O)c5ccco5)CCCCC	6.4	6.6	6.50
649	ZINC02091929	O=C(c1c2cc(OC)ccc2n(c1C)C)COc3cc(cc4c3C(CCCC)=CC(O4)=O)C	6.4	6.6	6.50
650	ZINC13727702	O=C(Nc1ccc2c(c(o2)C(=O)c3ccc(OC)cc3)C)c1)CCc4cc(OC)no4	6.3	6.7	6.50
651	MolPort-002-518-545	O=C(NCCc1ccc(OC)c(OC)c1)CCC=2C(Oc3cc4c(cc3C2C)c(C)co4)=O	6.3	6.7	6.50
652	SN00396633	O=C1[C@@H](OC(=O)C)C[C@]2([C@H](C(CC[C@@]2(C1(C)C)C)=C)COc3c(OC)cc4C=CC(Oc43OC)=O)C	6.3	6.7	6.50
653	ZINC02148113	O=C1C(CCC([O-])=O)=C(c2ccc(OCc3cccc(c3)C)cc2O1)C	6.3	6.7	6.50
654	ZINC02116736	O=C1C=C(c2ccccc2)c3c(O1)c(c4c(CN(CO4)c5c(C)ccc(c5)C)c3)C	6.3	6.7	6.50
655	ZINC68569482	ClS(=O)(=O)c1ccc(OCC)c(C2=Nc3c(nn(c3C(=O)N2)C)C(C)(C)C)c1	6.3	6.7	6.50
656	SN00077982	O=C1[C@@H-]([C@H](N(CCOC)C1=O)c2ccc(OC)c(OC)c2)C(=O)c3ccco3	6.2	6.8	6.50
657	MolPort-028-854-753	Oc1ccc2C(=O)C(c3ccc(O)cc3)=COc2c1[C@H](c4cc(OC)c(OC)cc4OC)CC(OC)=O	6.2	6.8	6.50
658	ZINC00480646	O=C1C2=C(N(C(=O)C[C@@H]2c3ccc(OC)c(OC)c3)C)CC(C1)(C)C	6.2	6.8	6.50
659	ZINC11866575	O=C1C=2CCCC2c3c(O1)cc(OCc4cc(OC)c(OC)c(OC)c4)c(CCCCCC)c3	6.1	6.9	6.50
660	3037	Oc1c(c(OC)c(c(c1C)C)C(Oc2c(c(OC)c(c(c2C)C)C([O-])=O)C)=O)C	6.1	6.9	6.50

661	ZINC04046044	Clc1c(Cl)ccc(COc2ccc3c(OC(=O)C(CCC([O-])=O)=C3C)c2)c1	6.1	6.9	6.50
662	ZINC02148012	FC(F)(F)c1cccc(COc2ccc3C(=C(CCC(OCC)=O)C(Oc3c2C)=O)C)c1	6.5	6.4	6.45
663	ZINC09357294	FC(F)(F)c1c(c(n[nH]1)-c2ccc(OC)cc2O)-c3ccc(OC)cc3	6.4	6.5	6.45
664	SN00092731	O=C(c1ccc(OC)c(c1)C)[C@@H-]2C(=O)C(=O)N([C@H]2c3cc(OC)c(OC)c(OC)c3)CCOC	6.4	6.5	6.45
665	ZINC04222564	Fc1cc(F)cc(CNC(=O)C[C@H]2c3c(sc(n3)NCC=C)C[C@H]4[C@]([C@@H](O)CC[C@]24C)(CO)C)c1	6.4	6.5	6.45
666	SN00107363	Brc1ccc(C(=O)Nc2cc(OC)c(OC)cc2Cc3c4cc(OC)c(OC)cc4ccn3)cc1	6.4	6.5	6.45
667	ZINC02151928	O=C1[C@@H]2Cc3c4ccccc4[nH]c3[C@H](N2C(=O)CN1CCCOC)c5cc(OC)c(OC)c(OC)c5	6.4	6.5	6.45
668	MolPort-044-180-902	Oc1c(c2c(C(c3ccccc3)=CC(O2)=O)cc1CN(C[C@@H]4CCCN5CCCC[C@H]45)CCCOC)C	6.2	6.7	6.45
669	SN00080681	O=C1c2ccccc2OC(NC(=O)c3cccc(OC)c3)=C1c4cc(OC)c(OC)c(OC)c4	6.2	6.7	6.45
670	ZINC02120791	Clc1ccc(-c2coc3cc4c(C(=C(CC(=O)N[C@@H]([C@H](CC)C)C([O-])=O)C(O4)=O)C)cc32)cc1	6.2	6.7	6.45
671	SN00376520	O[C@H]1[C@@H](NC)C[C@H]2n3c4ccc(O)cc4c5c6C(=O)NCc6c7c8ccccc8n([C@]1(O2)C)c7c53	6.2	6.7	6.45
672	SN00333183	O=C1CC[C@@]2([C@H](C(CC[C@@]2(C1(C)C)C)=C)COc3c(OC)cc4C=CC(Oc4c3OC)=O)C	6.2	6.7	6.45
673	189083-78-1	Oc1c(OC)ccc(-c2c3-c4cc(OC)c(O)cc4OC(=O)c3n5CCc6c(OC)c(OC)c(OC)cc6-c25)c1	6.2	6.7	6.45
674	ZINC02152224	O=C(c1c2cc(OC)ccc2n(c1C)C)COc3ccc4c(OC(=O)C=C4CCCC)c3C	6.2	6.7	6.45
675	SN00294537	Oc1ccc2cc1-c3c(OC)ccc(C[C@@H]4c5cc(Oc6c(OC)c(OC)cc7CCN([C@H](c67)C2)C)c(OC)cc5CCN4)c3	6.2	6.7	6.45
676	ZINC02092882	Clc1cccc(COc2ccc3c(OC(=O)C(=C3C)CCC([O-])=O)c2C)c1	6.2	6.7	6.45
677	MolPort-044-543-486	OC=1C(=O)C=C(OC1[C@@H](CC(=O)NCCN(C)C)c2ccc(O)c(OC)c2)C	6.0	6.9	6.45
678	ZINC02148765	O=C1C(=C(c2ccc(OCc3cc(cc(c3)C)C)c(c2O1)C)C)CC([O-])=O	5.9	7.0	6.45
679	SN00333183	O=C1CC[C@]2([C@H](C(CC[C@@]2(C1(C)C)C)=C)COc3c(OC)cc4C=CC(Oc4c3OC)=O)C	6.3	6.6	6.45
680	ZINC06058691	O=S(=O)(Nc1cc(OC)c(OC)cc1Cc2c3cc(OC)c(OC)cc3ccn2)c4ccc(OC)c5ccccc54	6.3	6.6	6.45
681	ZINC68575391	FC(F)(C(F)(F)O[C@@](F)(C(F)(F)F)/C(O)=N/c1c(F)c(F)c(F)c(F)c1F)C(F)(F)F	6.3	6.6	6.45
682	ZINC06075836	Clc1cc(Cl)ccc1CCNC(=O)c2cc3c4ccccc4[nH]c3c(n2)C	6.3	6.6	6.45
683	SN00366689	O[C@@H]1CC[C@]2([C@@H](C(=CC[C@H]2C1(C)C)C)COc3c(OC)cc4C=CC(Oc4c3OC)=O)C	6.3	6.6	6.45
684	ZINC02095090	O=S(=O)(N[C@@H]([C@@H](CC)C)C(Oc1ccc2c(OC(=O)C=3CCCCC23)c1)=O)c4ccc(cc4)C	6.3	6.6	6.45
685	MolPort-027-718-092	O=C(NCCN(C)C)CCn1c2ccccc2c3CCN4C(=O)c5ccccc5N=C4c31	6.3	6.6	6.45
686	ZINC30882130	Fc1ccc(C(=O)NC[C@@H]2C=C([C@@H](C[C@@H]2C(C)C)CC(=O)Nc3ccccn3)C)cc1	6.1	6.8	6.45
687	ZINC20466245	O[C@H]1[C@H](CC[C@@]2(CC[C@H](NC(=O)c3ccc(OC)cc3)[C@@H]([C@@H]12)C)C)[C@H](C(=O)N4CCCC4)C	6.5	6.3	6.40
688	ZINC02125435	Brc1ccc(N2COc3c(c4c(cc3C2)C(C)=C(C(O4)=O)C)C)c(C)c1	6.4	6.4	6.40
689	ZINC02227306	Clc1cccc(COc2ccc3C(=O)C(c4ccc(OC)cc4)=COc3c2)c1	6.4	6.4	6.40
690	MolPort-038-386-398	Clc1ccc(-c2c(c(on2)[C@H](O)C)C(=O)N(CCc3ccccn3)C)cc1	6.4	6.4	6.40
691	MolPort-028-855-303	O=C1C([C@H](CC(OC)=O)C2=COc3ccccc3C2=O)=C(NN1)Cc4ccc(OC)cc4	6.4	6.4	6.40
692	MolPort-002-513-842	O=C(N[C@H](Cc1c[nH]c2ccccc21)C(O)=O)[C@H](Oc3ccc4c(OC(=O)C=C4CC)c3)C	6.3	6.5	6.40
693	MolPort-000-847-367	O=C(NCCc1c[nH]c2cc(OC)ccc21)COc3ccc4c(OC(=O)C(=C4C)C)c3	6.3	6.5	6.40
694	MolPort-001-573-006	Clc1ccc(C(=O)Nc2cc(OC)c(OC)cc2Cc3c4cc(OC)c(OC)cc4ccn3)cc1	6.3	6.5	6.40
695	MolPort-028-855-404	O=C1C[C@H](c2c(O1)cc(OC)c3C(=O)C=C(Oc32)c4ccccc4)c5cc(OC)c(OC)c(OC)c5	6.2	6.6	6.40
696	ZINC04236557	Fc1cc(F)cc(CNC(=O)C[C@H]2[C@@H](O)CC[C@@H]3[C@@]([C@@H](OC(=O)NCC)CC[C@@]23C)(CO)C)c1	6.2	6.6	6.40
697	ZINC20761201	O=C(N1CCc2c([nH]cn2)[C@H]1c3cccc(OC)c3)N[C@H]([C@@H](CC)C)C(OC)=O	6.2	6.6	6.40
698	62008-00-8	Br[C@H]1CCC([C@H](C1(C)C)Cc2c(O)cc(Br)c(OC)c2)=C	6.2	6.6	6.40
699	ZINC01902806	FC(F)(F)c1cccc(COc2ccc3c(OC(=O)C=C3c4ccc(OC)cc4)c2)c1	6.2	6.6	6.40
700	ZINC35466060	O=C1C2=C([C@H](OC)[C@@H](OC(=O)/C=C\C=C\C=C\C)[C@@H]3C(CCC[C@]23C)(C)C)CN1[C@@H](C)C([O-])=O	6.1	6.7	6.40
701	SN00074324	O=C1c2ccccc2C=3[C@H]1[C@@H](c4ccc(OC)c(OC)c4)C=5C(=O)N(C(=O)N(C5N3)C)C	6.1	6.7	6.40
702	ZINC30880470	Fc1ccc(CNC(=O)C[C@H]2C(=C[C@H]([C@H](C2)C(C)C)CNC(=O)CCC([O-])=O)C)cc1	6.1	6.7	6.40
703	SN00092731	O=C(c1ccc(OC)c(c1)C)[C@@H-]2C(=O)C(=O)N([C@@H]2c3cc(OC)c(OC)c(OC)c3)CCOC	6.0	6.8	6.40
704	ZINC02093739	O=C(c1c2ccccc2n(c1C)C)COc3ccc4c(OC(=O)C=5CCCCC45)c3C	6.0	6.8	6.40
705	ZINC03999825	OCCCN1C(=O)c2ccccc2N=C1/C=C/c3c4ccccc4n(c3)C	5.9	6.9	6.40

706	MolPort-000-797-746	OC1(C=C(C[C@]([C@@H]1C(=O)c2ccc(OC)cc2)(c3ccc(OC)cc3)C)c4ccc(OC)cc4)c5ccc(OC)cc5	5.9	6.9	6.40
707	ZINC72325705	O=C(NCc1c(nn(c1C)C)C)CCC=2C(Oc3c(c4c(cc3C2C)c(c(o4)C)C)C)=O	6.5	6.2	6.35
708	ZINC02345008	Clc1cccc(COc2ccc3C(=O)C(c4ccc(Cl)cc4)=COc3c2)c1	6.5	6.2	6.35
709	SN00372645	O=C(O[C@@H](Cc1cocc-2c(ccc21)C=O)/C=C(/CCC=C(C)C)C)C	6.3	6.4	6.35
710	ZINC06624183	O=C(N[C@@H]([C@H](CC)C)C([O-])=O)CC=1C(Oc2cc(c3c(C)coc3c2C1C)C)=O	6.3	6.4	6.35
711	SN00102958	O=C1C[C@H](c2c(N1)c(cn2-c3c(OC)ccc(OC)c3)C([O-])=O)c4ccc(OC)c(O)c4	6.3	6.4	6.35
712	MolPort-001-737-182	Clc1ccc(C(=O)Nc2cc(OC)c(OC)cc2Cc3c4cc(OC)c(OC)cc4ccn3)cc1Cl	6.2	6.5	6.35
713	169181-25-3	O=C(N([C@@H](C(C)C)/C=C(\C)C(O)=O)C)[C@H](NC(=O)[C@H](NC)C(c1c[nH]c2ccccc21)(C)C)C(C)C	6.2	6.5	6.35
714	SN00295762	O=C1C=C(Oc2c1ccc3c2cco3)c4c(OC)ccc(OC)c4	6.2	6.5	6.35
715	SN00379562	O=C1C=C[C@]2([C@@H](C(CC[C@H]2C1(C)C)=C)COc3c(OC)cc4C=CC(Oc4c3OC)=O)C	6.2	6.5	6.35
716	ZINC20611253	O=C(N[C@@H]([C@@H](CC)C)C([O-])=O)[C@@H](N1C(=O)Nc2ccccc2C1=O)CC(C)C	6.0	6.7	6.35
717	SN00282943	O=C1C(Nc2nc([C@@H](OC)c3ccc(OC)cc3)c(n2C)Cc4ccc(OC)cc4)=NC(=O)N1C	6.4	6.2	6.30
718	ZINC12896984	O=C(Nc1ccc2c(c1)C=CC(O2)=O)CC=3C(Oc4cc(cc(OCC(C)=C)c4C3C)C)=O	6.2	6.4	6.30
719	ZINC38139997	O=C(N1[C@H](CCC1)C([O-])=O)[C@H](NC(=O)[C@H](N2C(=O)Nc3ccccc3C2=O)Cc4ccccc4)[C@H](CC)C	6.2	6.4	6.30
720	SN00083504	O=C1C(=O)[C@@H-]([C@@H](N1CCCOC)c2cc(OC)c(OC)c(OC)c2)C(=O)c3ccc(o3)C	6.2	6.4	6.30
721	ZINC02145888	O=C1C2=C(OC([NH3+])=C([C@@H]2c3cc(OC)c4c(OCO4)c3)C#N)C=C(N1CCc5ccc(OC)c(OC)c5)C	6.2	6.4	6.30
722	MolPort-009-649-148	O=C1C[C@H](c2c(N1)c(cn2-c3cc(OC)ccc3OC)C(O)=O)c4ccc(OC)cc4	6.2	6.4	6.30
723	SN00138838	FC(F)(F)C(C(=O)Nc1cc(ccc1OC)C(F)(F)F)C(F)(F)F	6.2	6.4	6.30
724	ZINC68575413	FC(F)(O[C@](F)(C(=O)/N=C\1C=CC=C(N1)C)C(F)(F)F)[C@](F)(OC(F)(F)C(F)(F)C(F)(F)F)C(F)(F)F	6.2	6.4	6.30
725	ZINC04273464	S=C(NCC=C)N/N=C(/C=1C(=O)c2ccccc2N(CC)C1[O-])C	6.2	6.4	6.30
726	ZINC02151831	O=S(=O)(N[C@@H](CCCC)C(Oc1ccc2C(c3ccc(OC)cc3)=CC(Oc2c1C)=O)=O)c4ccc(cc4)C	5.9	6.7	6.30
727	ZINC11865226	Clc1c(Cl)cc(COc2ccc3C(=O)C(c4ccc(OC)cc4)=COc3c2)cc1	6.5	6.1	6.30
728	SN00061024	O=C1C2=C(OC(N)=C([C@H]2c3cc(OC)c(OC)c(OC)c3)C#N)C=C(N1Cc4ccc(OC)c(OC)c4)C	6.3	6.3	6.30
729	MolPort-028-855-479	O=C(NCCCOC)Cn1c2ccccc2c3CCN4C(=O)c5ccccc5N=C4c31	6.3	6.3	6.30
730	ZINC06075844	Clc1c(Cl)ccc(CNC(=O)c2cc3c4ccccc4[nH]c3c(n2)C)c1	6.3	6.3	6.30
731	ZINC20466733	Fc1cccc(C(=O)N[C@H]2CC[C@@]3(CC[C@@H]([C@@H](O)[C@@H]3[C@H]2C)[C@H](C(=O)N4CCCC4)C)C)c1	6.3	6.3	6.30
732	23230	Fc1cc(F)cc(CNC(=O)C[C@@H]2[C@@H](O)CC[C@@H]3[C@@]4(CO[C@H](O[C@@H]4CC[C@@]23C)CNS(=O)(=O)C)C)c1	6.1	6.5	6.30
733	ZINC71386405	Clc1ccccc1COc2ccc(-c3c(-c4ccc(OC)c(OC)c4)c(nc(n3)N)C(F)(F)F)c(O)c2	6.1	6.5	6.30
734	ZINC48057069	Oc1cccc(OC)c1C(=O)/C=C/c2cccc(OC)c2	6.1	6.5	6.30
735	SN00258775	O=C1C=Cc2ccc(OC)c(C[C@H]3C(O[C@@H](O3)C(Cc4c(OC)ccc5C=CC(Oc54)=O)(C)C)(C)C)c2O1	6.1	6.5	6.30
736	ZINC02092390	O=C(c1c2cc(OC)ccc2n(c1C)C)COc3cc(cc4c3C(C)=CC(O4)=O)C	6.1	6.5	6.30
737	MolPort-009-649-144	O=C1C[C@@H](c2c(N1)c(cn2-c3ccc(OC)cc3OC)C(O)=O)c4ccc(OC)c(OC)c4	6.0	6.6	6.30
738	SN00073804	O=C1c2ccccc2OC(/N=C(\O)c3ccccc3)=C1c4cccc(OC)c4	6.0	6.6	6.30
739	SN00083510	O=C1C2=C(N(C(=O)N1C)C)NC=3c4ccccc4C(=O)C3[C@H]2c5cc(OC)c(OC)c(OC)c5	6.0	6.6	6.30
740	ZINC02114015	O=C(N[C@@H]([C@@H](CC)C)C([O-])=O)CC=1C(Oc2c(OC)c(OC)ccc2C1C)=O	5.8	6.8	6.30
741	ZINC11865162	O=C(Oc1cc(OC)ccc1-c2c(-c3ccc(OC)cc3)c(no2)C)c4ccco4	6.4	6.1	6.25
742	MolPort-002-525-974	Clc1cc(Cl)ccc1CCN2COc3c(c4c(C(=C(C(O4)=O)C)C)cc3C2)C	6.2	6.3	6.25
743	ZINC02096348	O=C(c1c2cc(OC)ccc2n(c1C)C)COc3cc(cc4c3C(=C(Cc5ccccc5)C(O4)=O)C)C	6.2	6.3	6.25
744	MolPort-005-950-564	Fc1ccc(S(=O)(=O)N2Cc3c(ncn3Cc4ccc(OC)cc4)C[C@H]2C(OC)=O)cc1	6.2	6.3	6.25
745	ZINC79189935	O=C1C(=C(c2c(OCC(=O)NC[C@@H]3CCC[NH+]4CCCC[C@H]34)cc5c(CCC(O5)(C)C)c2O1)C)Cc6ccccc6	6.2	6.3	6.25
746	MolPort-001-745-897	Fc1ccc(-c2cc(n(n2)C)[C@H]3CN4CC[C@@H]3C[C@@H]4CNS(=O)(=O)c5c(OC)ccc(OC)c5)cc1	6.1	6.4	6.25
747	ZINC17198153	S=C1NC(O)=C([C@@H]2c3c(CCN2)c4ccccc4[nH]3)C(=O)N1CCCCCC	6.1	6.4	6.25
748	MolPort-039-195-378	Clc1ccc(C(=O)c2ccc3c([C@@H](c4cccc(OC)c4OC)CC(O3)=O)c2O)cc1	6.1	6.4	6.25
749	ZINC20467354	Fc1cccc(NC(=O)N[C@H]2CC[C@@]3(CC[C@@H]([C@@H](O)[C@@H]3[C@H]2C)[C@H](C(=O)N4CCCC4)C)C)c1	6.1	6.4	6.25

750	SN00053349	O=C1c2c(OC)c(OC)ccc2[C@H]3N(c4cc(cc(c4)C)C)C(=O)c5ccccc5N13	6.1	6.4	6.25
751	ZINC49543301	O=C1C[C@H](c2c(N1)c(cn2-c3ccc(OC)cc3OC)C([O-])=O)c4cccc(OC)c4	6.0	6.5	6.25
752	ZINC02095669	O=S(=O)(N[C@H]([C@@H](CC)C)C(Oc1ccc2C(c3ccc(OC)cc3)=CC(Oc2c1C)=O)=O)c4ccc(cc4)C	6.0	6.5	6.25
753	143682-18-2	Oc1cccc2c1n3c4c2c5CNC(=O)c5c6c7cc(O)ccc7n([C@@H]8C[C@@H](NC)[C@H](OC)[C@]3(O8)C)c64	6.0	6.5	6.25
754	129623-30-9	O=C1c2c(c3c4ccccc4n5c3c6c2c7ccccc7n6[C@@H]8C[C@H](N(C)C)[C@@H](OC)[C@]5(O8)C)CN1	6.0	6.5	6.25
755	MolPort-007-564-138	O=C(NCCc1c[nH]c2ccccc21)/C(NC(=O)c3cccc(c3)C)=C/c4ccc(OC)cc4	6.0	6.5	6.25
756	ZINC04046667	Clc1cccc(COc2ccc3c(OC(=O)C(=C3C)CCC([O-])=O)c2)c1	6.0	6.5	6.25
757	ZINC72324754	O=C(Nc1cccc2c1ccn2C)CCn3ccc4c(OC)cccc43	6.0	6.5	6.25
758	MolPort-005-910-869	O=C(NCCc1ccc(OC)c(OC)c1)c2ccccc2CC3=Cc4ccccc4C(O3)=O	5.9	6.6	6.25
759	MolPort-008-348-717	O=C1C[C@H](c2c(N1)c(cn2-c3ccc(OC)cc3)C(O)=O)c4cc(OC)c(O)c(OC)c4	5.9	6.6	6.25
760	SN00077983	O=C1[C@@H-]([C@@H](N(CCOC)C1=O)c2ccc(OC)c(OC)c2)C(=O)c3ccco3	5.9	6.6	6.25
761	ZINC04062053	O=C(NCCCCCC([O-])=O)COc1ccc2c(OC(=O)C=C2CCCC)c1C	5.9	6.6	6.25
762	MolPort-000-813-035	O=C(N[C@H](Cc1c[nH]c2ccc(O)cc21)C(O)=O)COc3ccc4c(OC(=O)C(=C4C)C)c3	5.8	6.7	6.25
763	ZINC11865225	Clc1c(Cl)ccc(COc2ccc3C(=O)C(c4ccc(OC)c(OC)c4)=COc3c2)c1	6.4	6.0	6.20
764	MolPort-039-194-841	O=C(NCc1ccccc1OC)C[C@@H](c2ccc(OC)c(OC)c2OC)C=3C(OC(=CC3O)C)=O	6.2	6.2	6.20
765	MolPort-044-810-910	O=C(NCCc1c[nH]cn1)Cc2c(oc3C(=O)C=C(Oc32)C)-c4ccc(OC)cc4	6.2	6.2	6.20
766	SN00161478	O=C1C=C2C=3C(Oc4cc(OC)c5C=C[C@H](Oc5c4C3C=C(O2)c6ccccc6)c7ccccc7)=C1C	6.2	6.2	6.20
767	ZINC08918194	Clc1cccc(COc2ccc3C(=C(CC(=O)N4CC[C@@]5(O)CCCC[C@@H]5C4)C(Oc3c2C)=O)C)c1	6.2	6.2	6.20
768	ZINC05817910	Oc1cc(OC)ccc1-c2c(-c3ccc(OC)c(OC)c3)c[nH]n2	6.0	6.4	6.20
769	ZINC12899640	O=C(Nc1ccc(OC)c(OC)c1)CC=2C(Oc3c(C2C)c(OC)cc4c3CCC(O4)(C)C)=O	6.0	6.4	6.20
770	ZINC38549848	O=C1C=C2C3=C[C@@](CC[C@@]3(CC[C@]2([C@]4(CC[C@H]5C([C@@H](OCO)CC[C@]5([C@H]14)C)(C)C)C)C)(C)C([O-])=O	6.0	6.4	6.20
771	ZINC02119925	Clc1cccc([C@H]2c3c(C[C@H]4C(=O)N(CCCOC)CC(=O)N42)c5ccccc5[nH]3)c1	6.0	6.4	6.20
772	MolPort-009-649-155	O=C1C[C@@H](c2c(N1)c(cn2-c3ccc(OC)cc3OC)C(O)=O)c4ccc(OC)cc4OC	6.0	6.4	6.20
773	MolPort-002-524-289	O=C(NCc1cnccc1)c2cc3c4ccccc4[nH]c3c(n2)-c5cc(OC)c(OC)c(OC)c5	6.0	6.4	6.20
774	ZINC49543327	O=C1C[C@H](c2c(N1)c(cn2-c3cc(OC)ccc3OC)C([O-])=O)c4ccc(OC)cc4	5.7	6.7	6.20
775	155233-31-1	O=C([C@@H](NC(=O)C[C@H](NC(=O)/C=C\C=C/C)c1ccccc1)C(C)C)[C@H]2C(=O)NC(=O)[C@H]2C	6.3	6.1	6.20
776	181423-71-2	O=C1c2c(c(c3-c4cc(OC)c(OC)cc4CCn23)-c5ccc(OC)c(OC)c5)-c6cc(OC)c(OC)cc6O1	6.3	6.1	6.20
777	ZINC04046180	Fc1cccc(COc2ccc3c(OC(=O)C(=C3C)CC([O-])=O)c2)c1	6.1	6.3	6.20
778	MolPort-028-856-366	Oc1ccc2c(oc3c2nc4c(c3-c5ccc(OC)cc5)cnn4C(C)C)c1C	6.1	6.3	6.20
779	MolPort-007-902-409	O=C(NCc1ccccc1OC)c2ccc3c(nc(c(n3)-c4cc(OC)c(OC)c(OC)c4)-c5cc(OC)c(OC)c(OC)c5)c2	6.1	6.3	6.20
780	ZINC02120633	O=C(N[C@H]([C@@H](CC)C)C([O-])=O)CC=1C(Oc2cc3c(c(C(C)(C)C)co3)cc2C1C)=O	5.8	6.6	6.20
781	12190	Fc1ccccc1C[NH+](C[C@H]2C=C([C@@H](C[C@H]2C(C)C)Cc3nc4cccc(c4[nH]3)C)C)Cc5ccccc5F	6.3	6.0	6.15
782	99457-91-7	O[C@@H]1C(=O)C(=C(O)C(=O)[C@@H]([C@@H]2C=C(CC[C@@H]1C(C)=C)C(O2)=O)C(C)=C)C	6.2	6.1	6.15
783	ZINC04089321	O=C(NCCCOC(C)C)CC=1C(Oc2c(c3c(c4c(o3)CCCC4)cc2C1C)C)=O	6.1	6.2	6.15
784	ZINC02149278	Clc1c(Cl)ccc(COc2ccc3C(=C(CC(OC)=O)C(Oc3c2C)=O)C)c1	6.1	6.2	6.15
785	ZINC30881458	Fc1cccc(C(=O)NC[C@@H]2C=C([C@@H](C[C@@H]2C(C)C)CC(=O)NCC#C)C)c1	6.0	6.3	6.15
786	MolPort-019-936-025	Fc1ccc(-c2cc(no2)CC3(NCc4ccc(OC)cc4O)COC3)cc1	5.9	6.4	6.15
787	ZINC02104075	O=C(N[C@@H]([C@@H](CC)C)C([O-])=O)CC=1C(Oc2cc3c(c4c(o3)CCCC4)cc2C1C)=O	5.8	6.5	6.15
788	MolPort-002-533-189	O=C1c2ccccc2OC(NC(=O)c3ccco3)=C1c4ccc(OC)c(OC)c4	5.7	6.6	6.15
789	ZINC02092427	Clc1c(Cl)ccc(COc2ccc3C(=C(CC([O-])=O)C(Oc3c2C)=O)C)c1	5.7	6.6	6.15
790	MolPort-028-854-697	O=C1C=C(c2cc3c(CNCc4ccc(OC)c(OC)c4)cnc(c3o2)C)c5ccc(c(c5O1)C)C	6.2	6.0	6.10
791	ZINC19927379	OC1=C(C(=O)c2cccc3c2N1CCC3)C(=O)NCCCN(C)C	6.1	6.1	6.10
792	SN00060965	O=C1C2=C(OC(N)=C([C@@H]2c3ccc(OC)c(OC)c3OC)C#N)C=C(N1Cc4ccc(OC)c(OC)c4)C	6.1	6.1	6.10
793	SN00062839	O=C1C2=C([C@H](N1CCc3ccc(OC)cc3)c4ccc(O)c(OC)c4)C(=O)c5ccccc5O2	6.0	6.2	6.10

794	ZINC12875599	Clc1cccc(F)c1CNC(=O)[C@H](N2C(=O)N3CCc4c5ccccc5[nH]c4[C@]3(C2=O)C)CC(C)C	6.0	6.2	6.10
795	MolPort-028-856-471	O=C(NCCCN(C)C)Cn1c2ccccc2c3CCN4C(=O)c5ccccc5N=C4c31	6.0	6.2	6.10
796	ZINC08791756	O=C(N[C@H]([C@H](CC)C)C(=O)NCc1cccc(OC)c1)[C@H]2C(S[C@@H]3c4ccccc4C(=O)N32)(C)C	6.0	6.2	6.10
797	MolPort-009-649-145	O=C1C[C@@H](c2c(N1)c(cn2-c3ccc(OC)cc3OC)C(O)=O)c4cccc(OC)c4OC	5.9	6.3	6.10
798	SN00073864	O=C1c2ccccc2OC(/N=C(\O)c3cccc(OC)c3)=C1c4cccc(OC)c4	5.9	6.3	6.10
799	MolPort-002-087-773	Oc1cc(OC)ccc1-c2c(-c3ccc(OC)cc3)c([nH]n2)C	5.9	6.3	6.10
800	ZINC03846616	O=C(C[C@@H](Nc1cccc(N)c1)c2ccc(N(C)C)cc2)C=3C(=O)c4ccccc4N(C3[O-])C	5.8	6.4	6.10
801	MolPort-035-873-330	O=C1C([C@H](c2ccccc2OCCc3ccc(OC)cc3)CC(OC)=O)=C(O)C=C(N1CCc4ccc(O)cc4)C	5.8	6.4	6.10
802	MolPort-009-649-145	O=C1C[C@H](c2c(N1)c(cn2-c3ccc(OC)cc3OC)C(O)=O)c4cccc(OC)c4OC	5.8	6.4	6.10
803	MolPort-035-702-025	O=C(N1CCN(CC1)C)C[C@H](c2c(O)c(O)c(O)c3C(=O)C=C(Oc32)c4ccccc4)c5cc(OC)c(OC)c(OC)c5	5.7	6.5	6.10
804	MolPort-005-950-438	O=C(NCc1ccc(OC)cc1)[C@H]2Cc3c(nc[nH]3)CN2Cc4cc5ccccc5o4	5.7	6.5	6.10
805	MolPort-002-533-108	O=C1c2ccccc2OC(NC(=O)c3ccccc3)=C1c4cccc(OC)c4	5.6	6.6	6.10
806	MolPort-002-529-261	OC(=O)[C@@H](NC(=O)CCC=1C(Oc2cc3c(c(c(o3)C)C)cc2C1C)=O)Cc4c[nH]c5ccc(O)cc54	5.6	6.6	6.10
807	ZINC02148665	Fc1cccc(COc2ccc3c(OC(=O)C(=C3C)CC([O-])=O)c2C)c1	5.5	6.7	6.10
808	ZINC30881020	Fc1cccc(C(=O)NC[C@@H]2C=C([C@@H](C[C@@H]2C(C)C)CC(=O)N3CCOCC3)C)c1	6.2	5.9	6.05
809	ZINC02095349	O=C(c1c2ccccc2n(c1C)C)COc3ccc4C(c5ccc(OC)cc5)=CC(Oc4c3C)=O	5.9	6.2	6.05
810	MolPort-000-814-627	O=C(NCCc1ccc(OC)c(OC)c1)COc2cc(cc3c2C(=C(C)C(O3)=O)C)C	5.9	6.2	6.05
811	MolPort-039-057-703	Oc1ccc2c(oc3c2nc4c(c3-c5ccc(OC)c(O)c5)cnn4C)c1C	5.9	6.2	6.05
812	MolPort-008-348-717	O=C1C[C@@H](c2c(N1)c(cn2-c3ccc(OC)cc3)C(O)=O)c4cc(OC)c(O)c(OC)c4	5.7	6.4	6.05
813	MolPort-000-792-600	O=C(N[C@H](C(=O)N[C@@H](Cc1c[nH]c2ccccc21)C(O)=O)C)CCC=3C(Oc4cc5c(c(o5)C)C)cc4C3C)=O	5.7	6.4	6.05
814	ZINC04023594	O=C(OCC1([C@@H](C=C(C[C@H]1C)C)C)COC(=O)CCCC)CCCC	6.1	6.0	6.05
815	MolPort-002-518-223	O=S(=O)(N[C@H](CC)C(Oc1ccc2-c3ccc(OC)cc3C(Oc2c1C)=O)=O)c4ccc(cc4)C	6.0	6.1	6.05
816	MolPort-002-523-304	Clc1cccc(CNC(=O)[C@@H](N2C(=O)N3CCc4c5ccccc5[nH]c4[C@]3(C2=O)C)C)c1	6.0	6.1	6.05
817	SN00243902	O=C(O[C@H]1CC[C@]2([C@H](C(CC[C@@H]2C1(C)C)=C)COc3c(OC)cc4C=CC(Oc4c3OC)=O)C)C	6.0	6.1	6.05
818	149378-57-4	O=C1c2c(c(c3-c4cc(OC)c(O)cc4CCn23)-c5ccc(OC)c(O)c5)-c6cc(OC)c(O)cc6O1	6.0	6.1	6.05
819	ZINC35466060	O=C1C2=C([C@@H](OC)[C@H](OC(=O)/C=C\C=C\C=C\C)[C@@H]3C(CCC[C@]23C)(C)C)CN1[C@@H](C)C([O-])=O	5.8	6.3	6.05
820	9574	Clc1ccc(-c2noc(n2)[C@@H]3Cc4c(nc[nH]4)CN3C(=O)c5ccc(F)cc5)cc1	5.8	6.3	6.05
821	ZINC02125058	O=C(NCC(=O)N[C@H]([C@@H](CC)C)C([O-])=O)CCC=1C(Oc2c(C1C)cc3c(occ3C(C)(C)C)c2C)=O	6.1	5.9	6.00
822	ZINC38143767	O[C@@H]1C[C@@]2([C@@]([C@H]1[C@@](O)(C(=O)CCC(O)(C)C)C)(CC(=O)[C@]3([C@H]4C[C@H](O)C(=O)C(C4=CC[C@H]32)(C)C)C)C)C	6.1	5.9	6.00
823	MolPort-002-518-797	Fc1ccccc1NC(=O)CCC=2C(Oc3cc4c(c(o4)C)C)cc3C2C)=O	5.9	6.1	6.00
824	ZINC12899873	O=C1c2c(OC)c(OC)ccc2[C@@H]3N1[C@H](C(S3)(C)C)C(=O)Nc4ccc5c(c4)C=CC(O5)=O	5.9	6.1	6.00
825	SN00344408	O[C@H]1c2cc(OC)c(OC)c(OC)c2-c3c(OC(=O)[C@H](CC)C)c(OC)c(OC)cc3C[C@H]([C@@H]1C)C	5.9	6.1	6.00
826	ZINC06167362	Clc1cc(Cl)ccc1CNC(=O)c2cc3c4ccccc4[nH]c3c(n2)C	5.8	6.2	6.00
827	SN00335313	O=C1C=C[C@]2([C@H](C(=CC[C@H]2C1(C)C)C)COc3c(OC)cc4C=CC(Oc4c3OC)=O)C	5.7	6.3	6.00
828	152273-84-2	Oc1c(OC)c(OC)c(O)c(Cc2c(nc(n2C)NC=3C(=O)N(C)C(=O)N3)Cc4ccc(OC)cc4)c1	5.7	6.3	6.00
829	ZINC02123590	O=C(NCCCCCC([O-])=O)CC=1C(Oc2cc(OC)cc(OC)c2C1C)=O	5.5	6.5	6.00
830	MolPort-002-518-554	Fc1cc(F)ccc1NC(=O)CCC=2C(Oc3cc4c(c(o4)C)C)cc3C2C)=O	5.9	6.0	5.95
831	MolPort-038-386-405	Clc1ccc(-c2c(c(on2)[C@H](O)C)C(=O)NCCOC)cc1	5.9	6.0	5.95
832	ZINC02095346	O=S(=O)(N[C@H](CCCC)C(Oc1ccc2c(OC(=O)C=C2CCCC)c1)=O)c3ccc(cc3)C	5.9	6.0	5.95
833	ZINC02667597	Clc1c(Cl)cc(COc2ccc3c(OC=C(c4ccccc4OC)C3=O)c2)cc1	5.9	6.0	5.95
834	ZINC17146927	O=C(C1=C([O-])C(=O)N([C@H]1c2cc(OC)c(OC)c(OC)c2)CCOC)c3ccc(OC)c(c3)C	5.7	6.2	5.95
835	ZINC02157897	O=C(NCCCCCC([O-])=O)CC=1C(Oc2c(c3c(cc2C1C)c(c(o3)C)C)C)=O	5.5	6.4	5.95

836	MolPort-039-194-689	O=C1C[C@@H](c2cc(OC)c(OC)c(OC)c2)c3c(O1)ccc4C(=O)C(c5ccc(OC)c(OC)c5)=COc43	5.5	6.4	5.95
837	ZINC08790722	Clc1cccc(COc2ccc3C=C(C4=CC(Oc5c(OC)c(OC)ccc45)=O)C(Oc3c2)=O)c1	5.5	6.4	5.95
838	ZINC02117069	O=C1C=2CCCC2c3cc4c(OCN(C4)c5c(C)ccc(c5)C)c(c3O1)C	5.8	6.1	5.95
839	ZINC02125535	O=C1c2ccccc2-c3cc4c(OCN(C4)c5cccc(c5C)C)c(c3O1)C	5.8	6.1	5.95
840	ZINC79192837	O=C1c2ccccc2C(C(=O)NC[C@H]3CCC[NH+]4CCCC[C@@H]34)=CN1Cc5ccccc5	5.8	6.1	5.95
841	MolPort-009-649-144	O=C1C[C@H](c2c(N1)c(cn2-c3ccc(OC)cc3OC)C(O)=O)c4ccc(OC)c(OC)c4	5.6	6.3	5.95
842	SN00051121	N/C(=[NH+]\c1cc2cc(OC)c(OC)cc2c(n1)Cc3ccc(OC)c(OC)c3)Cc4ccc(OC)c(OC)c4	6.0	5.8	5.90
843	ZINC31168056	O[C@H](C(O)(C)C)[C@H](O)C/C(=C/CC/C(=C\COc1ccc2C=CC(Oc2c1)=O)C)C	5.8	6.0	5.90
844	MolPort-039-194-968	OC=1C(=O)C=C(OC1[C@H](c2ccc(SC)cc2)CC(OC)=O)CN3Cc4cc(OC)c(OC)cc4CC3	5.7	6.1	5.90
845	ZINC12296372	O=C1c2c(cc3c(c2O[C@H](c4cccc(OC)c4OC)C1)C(=C(C(O3)=O)C)C)C	5.7	6.1	5.90
846	ZINC02123120	O=C(N[C@@H](CCCC)C([O-])=O)CC=1C(Oc2cc3c(c(-c4ccccc4)c(o3)C)cc2C1C)=O	5.7	6.1	5.90
847	ZINC68589300	ClCCCC(=O)Nc1c2c(nc3CCCCc31)CCC2	5.6	6.2	5.90
848	SN00102579	O=C1C[C@@H](c2c(N1)c(cn2-c3ccc(OC)cc3)C([O-])=O)c4cccc(OC)c4OC	5.5	6.3	5.90
849	SN00305462	O[C@]12c3c(OC)cc(OC)cc3O[C@@]([C@@H]([C@@H]2C(=O)N4CCC[C@H]4NC(=O)/C(C)=C\C)c5ccccc5)([C@@H]1O)c6ccc(OC)cc6	5.4	6.4	5.90
850	MolPort-007-733-445	O=C1c2ccccc2C(=CN1c3cccc(OC)c3)C(=O)NCCc4ccc(OCC)cc4	5.9	5.8	5.85
851	17917	O=S(=O)(c1ccc(-c2c[nH]c(n2)[C@@H]3COCC[NH+]3Cc4ccc(OC)cc4)cc1)C	5.8	5.9	5.85
852	MolPort-001-999-765	O=C1N(C(=O)c2c(N1C)nc(Oc3cc(cc(c3)C)C)n2Cc4ccc(OC)cc4)C	5.8	5.9	5.85
853	SN00103065	O=C1C[C@H](c2c(N1)c(cn2-c3ccc(OC)cc3OC)C([O-])=O)c4ccc(OC)c(OC)c4	5.7	6.0	5.85
854	ZINC38143767	O[C@H]1C[C@@]2([C@@]([C@H]1[C@](O)(C(=O)CCC(O)(C)C)C)(CC(=O)[C@]3([C@H]4C[C@H](O)C(=O)C(C4=CC[C@H]32)(C)C)C)C)C	5.7	6.0	5.85
855	SN00254960	O=C1c2c(c(c3-c4cc(OC)c(OC)cc4CCn23)-c5ccc(OC)c(OC)c5)-c6cc(OC)c(O)cc6O1	5.7	6.0	5.85
856	SN00103017	O=C1C[C@H](c2c(N1)c(cn2-c3ccc(OC)cc3OC)C([O-])=O)c4ccc(O)c(OC)c4	5.6	6.1	5.85
857	MolPort-044-810-666	O=C(N[C@H](CCSC)c1nnc2ccccn21)Cc3c(oc4C(=O)C=C(Oc43)C)-c5ccc(OC)cc5	5.5	6.2	5.85
858	MolPort-042-674-422	Fc1cc(F)ccc1-c2ccc3c(O[C@H]4CN(C[C@H]4N(C3=O)C)CCCc5ccccc5)c2	5.9	5.7	5.80
859	MolPort-007-787-617	O=C(NCc1ccc(OC)cc1OC)c2c(nnc3ccccc32)-c4ccccc4	5.7	5.9	5.80
860	ZINC30883812	O[C@@H]1[C@H](CC[C@]2(CC[C@H](NC(=O)COC)[C@@H]([C@@H]12)C)C)[C@H](C(=O)N3CCCC3)C	5.7	5.9	5.80
861	MolPort-044-544-159	O=C(NCC(OC)OC)Cc1c(oc2C(=O)C=C(Oc21)C)-c3ccc(OC)cc3	5.4	6.2	5.80
862	MolPort-002-533-223	O=C1c2ccccc2OC(NC(=O)c3cccc(OC)c3)=C1c4cccc(OC)c4	5.4	6.2	5.80
863	ZINC05783367	ClCCCCC(=O)Nc1cc(nc2ccccc21)C	5.6	6.0	5.80
864	MolPort-002-184-761	O=C1C2=C(N(C(=O)N1C)C)NC=3c4ccccc4C(=O)C3[C@H]2c5ccc(O)c(OC)c5	5.6	6.0	5.80
865	ZINC02095836	O=S(=O)(N[C@@H](Cc1c[nH]c2ccccc21)C(Oc3ccc4c(OC(=O)C=C4CC)c3)=O)c5ccc(cc5)C	5.6	6.0	5.80
866	MolPort-001-683-688	O=C(OC(C)(C)C)N[C@@H](Cc1c[nH]c2ccccc21)C(Oc3ccc4C(=C(Cc5ccccc5)C(Oc4c3C)=O)C)=O	5.6	6.0	5.80
867	200936-84-1	O[C@@H](CCCCCC(=O)C=1C(O[C@]2(C1C(=C3C=C(OC=C3[C@@H]2O)C)C=O)C)=O)C	5.5	6.1	5.80
868	MolPort-001-906-348	O=C1C2=C(N(C(=O)N1C)C)NC=3c4ccccc4C(=O)C3[C@@H]2c5c(OC)ccc(OC)c5	5.5	6.1	5.80
869	ZINC02114872	O=C(NCCCCCC([O-])=O)[C@H](Oc1cc(cc2c1C3=C(CCCC3)C(O2)=O)C)C	5.3	6.3	5.80
870	189083-80-5	O[C@@H]1Cc2c(OC)c(OC)c(OC)cc2-c3c(c4-c5cc(OC)c(O)cc5OC(=O)c4n13)-c6ccc(OC)c(O)c6	5.5	6.0	5.75
871	ZINC70687176	Clc1ccc([C@@H]2[C@H]3CCCCC3(O)CCN2C(=O)COc4cc(cc5c4C(CCCC)=CC(O5)=O)C)cc1	5.4	6.1	5.75
872	SN00061023	O=C1C2=C(OC(N)=C([C@@H]2c3cc(OC)c(OC)c(OC)c3)C#N)C=C(N1Cc4ccc(OC)c(OC)c4)C	5.4	6.1	5.75
873	MolPort-008-348-069	O=C1C[C@H](c2c(N1)c(cn2-c3ccc(OC)cc3)C(O)=O)c4ccc(OC)cc4OC	5.5	5.9	5.70
874	19310	Fc1ccc(-c2c([C@@H](NC(=O)c3ccccn3)C)c(on2)C([O-])=O)cc1	5.2	6.2	5.70
875	MolPort-001-728-081	Fc1ccc(C(=O)N2CCc3cc(OC)c(OC)cc3[C@@H]2Cc4ccc(OC)c(OC)c4)cc1	5.6	5.8	5.70
876	MolPort-008-348-079	O=C1C[C@H](c2c(N1)c(cn2-c3ccc(OC)cc3)C(O)=O)c4cc(OC)c(OC)c(OC)c4	5.6	5.8	5.70
877	MolPort-044-543-703	O=C(NC[C@@H](N(C)C)c1ccco1)Cc2c(oc3C(=O)C=C(Oc32)C)-c4ccc(OC)cc4	5.3	6.1	5.70
878	MolPort-009-649-107	O=C1C[C@H](c2c(N1)c(cn2-c3ccc(OC)cc3OC)C(O)=O)c4ccc(O)c(OC)c4	5.3	6.1	5.70

879	ZINC70705416	O=C(N[C@H](c1ccccc1)C([O-])=O)[C@@H](NC(=O)[C@@H](N2C(=O)Nc3ccccc3C2=O)Cc4ccccc4)[C@@H](CC)C	5.3	6.1	5.70
880	MolPort-001-907-854	Clc1c(Cl)ccc(Cn2c3C(=O)N(C(=O)N(c3nc2NCCCOC)C)C)c1	5.3	6.1	5.70
881	ZINC02125325	O=C1C(C2=CC(Oc3cc(OCc4c(c(c(c(c4C)C)C)C)C)ccc32)=O)=Cc5cccc(OC)c5O1	5.3	6.1	5.70
882	MolPort-002-277-679	Clc1cc(Cl)ccc1Cn2c(nc3c2C(=O)N(C(=O)N3C)C)NCCOC	5.5	5.8	5.65
883	ZINC02115373	O=C(NCCCCCC([O-])=O)CC=1C(Oc2cc3c(c(C(C)(C)C)co3)cc2C1C)=O	5.3	6.0	5.65
884	ZINC04046886	O=C1C(CCC([O-])=O)=C(c2ccc(OCc3cccc(OC)c3)c(c2O1)C)C	5.2	6.1	5.65
885	ZINC85879780	OC=1C(=O)C=C(N(C1C(c2ccc(OC)c(OC)c2OC)C3=C(O)C(=O)C=C(N3C)C)C)C	5.4	5.8	5.60
886	SN00063749	Clc1ccc(C[C@@H](NC(=O)[C@@H](NC(=O)CC=2C(Oc3cc4c(c(c(o4)C)C)cc3C2C)=O)C)C([O-])=O)cc1	4.9	6.3	5.60
887	SN00098390	O=C1c2c(OC)c(OC)ccc2[C@H](O1)c3cc(OC)c(OC)cc3Cc4c5cc(OC)c(OC)cc5ccn4	5.4	5.7	5.55
888	MolPort-009-649-139	O=C1C[C@@H](c2c(N1)c(cn2-c3ccc(OC)cc3OC)C(O)=O)c4cccc(OC)c4	5.4	5.7	5.55
889	MolPort-002-510-470	OC=1C=C(OC(=O)C1C=2C[C@@H](Sc3ccccc3N2)c4cc(OC)c(OC)c(OC)c4)C	5.2	5.9	5.55
890	MolPort-005-307-707	O=C1C=C(c2ccc(OCc3nnnn3-c4ccc(OC)cc4)cc2O1)C	5.2	5.9	5.55
891	MolPort-008-348-309	O=C1C[C@@H](c2c(N1)c(cn2-c3ccc(OC)cc3)C(O)=O)c4cccc(OC)c4	5.3	5.8	5.55
892	SN00102409	O=C1C[C@H](c2c(N1)c(cn2-c3ccc(OC)cc3)C([O-])=O)c4cc(OC)c(OC)cc4OC	5.1	6.0	5.55
893	SN00103067	O=C1C[C@H](c2c(N1)c(cn2-c3ccc(OC)cc3OC)C([O-])=O)c4cccc(OC)c4OC	5.0	6.1	5.55
894	ZINC05452024	Oc1cc(OCC)ccc1-c2c(-c3ccc(OC)cc3)c(no2)C	5.3	5.7	5.50
895	MolPort-007-796-366	O=C(NCc1ccc(OC)c(OC)c1)CCc2cn(nc2-c3ccc(cc3)C)-c4ccccc4	5.2	5.8	5.50
896	MolPort-001-973-554	O=C1C2=C(N(C(=O)N1C)C)NC=3c4ccccc4C(=O)C3[C@@H]2c5cc(OC)c(OC)c(OC)c5	5.1	5.8	5.45
897	SN00102531	O=C1C[C@H](c2c(N1)c(cn2-c3ccc(OC)cc3)C([O-])=O)c4ccc(OC)c(OC)c4	4.9	5.9	5.40
898	MolPort-008-348-587	O=C1C[C@H](c2c(N1)c(cn2-c3ccc(OC)cc3)C(O)=O)c4ccc(OC)c(OC)c4OC	5.2	5.5	5.35
899	ZINC06786347	O=C1C([O-])=C([C@@H](N1CCOC)c2ccc(O)c(OC)c2)C(=O)c3ccccc3	4.9	5.8	5.35
900	MolPort-035-701-663	O=C(N1CCc2c3ccccc3[nH]c2C1)C[C@H](c4ccc(OC)c(OC)c4)C5=C(O)C(=O)C=C(O5)C	5.1	5.5	5.30
901	MolPort-007-846-702	O=C(NCCc1ccc(OC)c(OC)c1)C[C@H](n2cccc2)c3ccccc3C	5.1	5.5	5.30
902	MolPort-002-524-175	Clc1ccc(C[C@@H](NC(=O)COc2cc(cc3c2C(C)=CC(O3)=O)C)C(O)=O)cc1	4.8	5.8	5.30
903	MolPort-002-528-359	O=C(N[C@@H](Cc1c[nH]c2ccc(O)cc21)C(O)=O)[C@H](Oc3ccc4C=CC(Oc4c3)=O)C	4.9	5.6	5.25
904	SN00102042	O=C1C[C@H](c2c(N1)c(cn2-c3ccc(OC)cc3)C([O-])=O)c4ccc(OC)cc4OC	4.6	5.8	5.20
905	MolPort-002-571-308	O=C1c2c(nc(n2Cc3cccc(c3)C)NCCN(C)C)N(C)C(=O)N1	4.7	5.4	5.05
906	ZINC13409784	S=C1NN=C(N1/N=C\c2ccc(OC)cc2)CCn3c(nc4ccccc43)C	4.5	5.2	4.85
907	SN00102051	O=C1C[C@H](c2c(N1)c(cn2-c3ccc(OC)cc3)C([O-])=O)c4cc(OC)c(OC)c(OC)c4	4.2	5.5	4.85
908	SN00102958	O=C1C[C@@H](c2c(N1)c(cn2-c3c(OC)ccc(OC)c3)C([O-])=O)c4ccc(OC)c(O)c4	4.1	5.6	4.85
909	ZINC20467021	O[C@H]1[C@H](CC[C@@]2(CC[C@H](NC(=O)c3ccncc3)[C@@H]([C@H]12)C)C)[C@H](C(=O)N(CCC#N)C)C	4.5	5.0	4.75
910	SN00086102	OC=1C=C(OC(=O)C1C=2C[C@H](Sc3ccccc3N2)c4cc(OC)c(OC)c(OC)c4)C	4.0	5.3	4.65

Table S1bis. Structures, experimental and predicted (field and SVM) pKi values of the molecules in the training set

n	Structure	$_{exp}pK_i$	$_{field}pK_i$	$_{SVM}pK_i$
1	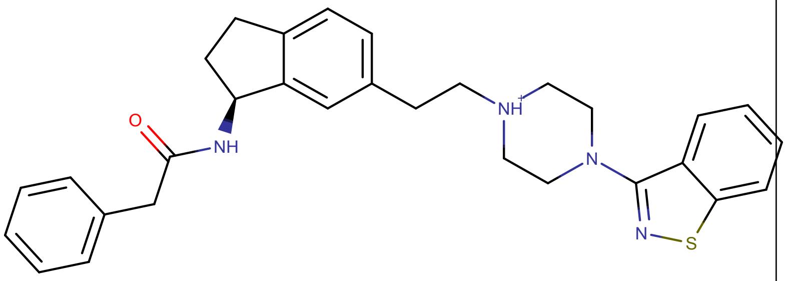	10.4	10.4	10.4
2	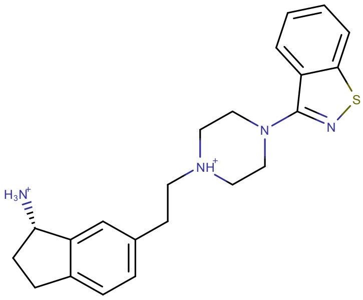	10.1	9.8	10
3	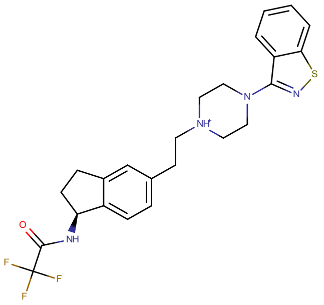	9.8	9.5	9.8
4	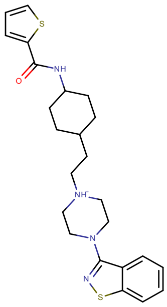	9.8	9.4	9.8

5	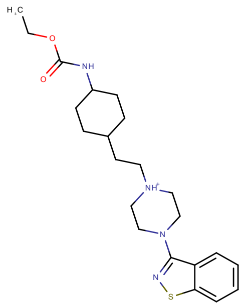	9.7	9.4	9.6
6	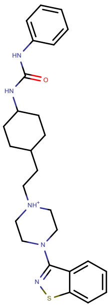	9.7	10	9.7
7	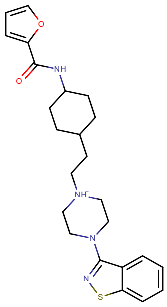	9.6	9	9.3
8	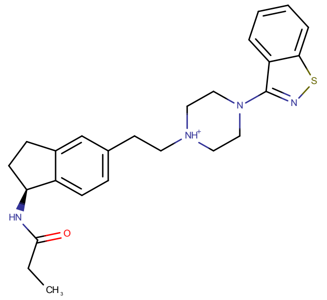	9.6	9.2	9.6
9	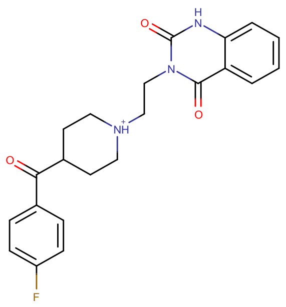	9.6	9.7	9.5

10	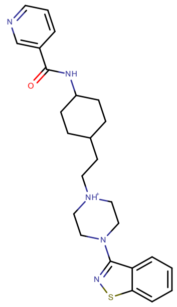	9.5	9.6	9.5
11	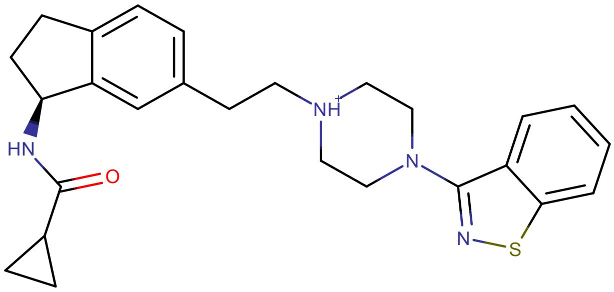	9.5	10.2	9.5
12	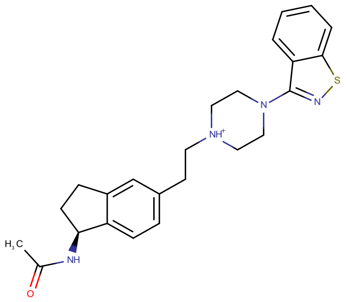	9.5	9	9.5
13	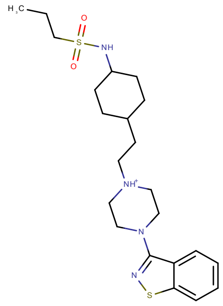	9.5	10	9.5
14	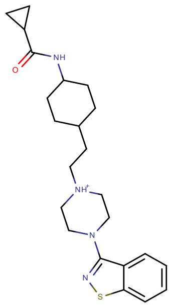	9.5	9.2	9.5

15	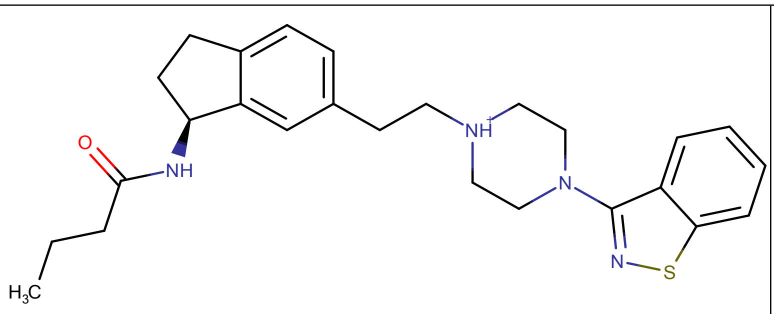	9.4	10	9.4
16	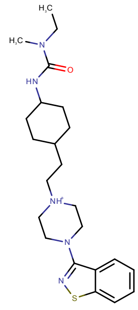	9.3	9.1	9.3
17	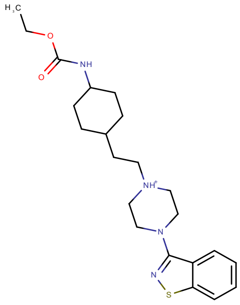	9.3	8.8	9.2
18	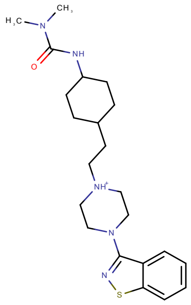	9.2	9.2	9.2
19	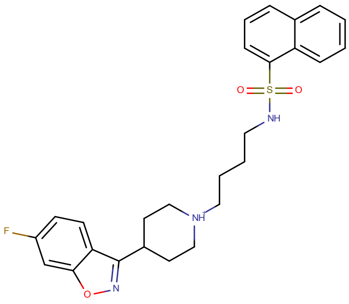	9.2	9	9.2

20	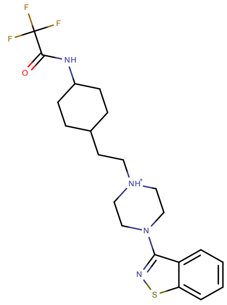	9.2	8.8	9.2
21	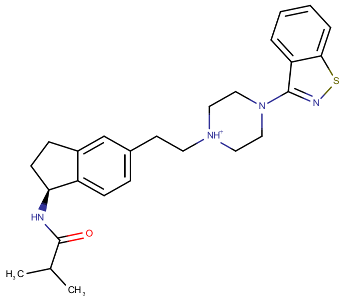	9.2	8.6	9.2
22	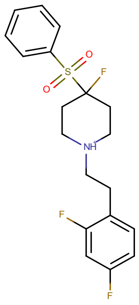	9.2	8.6	8.9
23	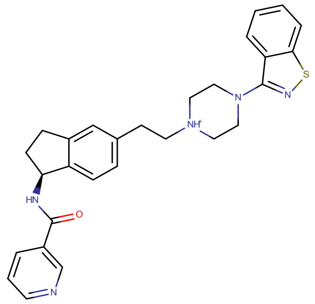	9.2	9.2	9.2
24	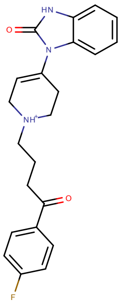	9.1	9.3	9.1

25	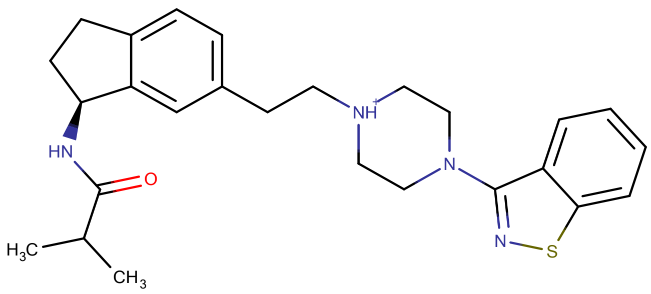	9.1	8.7	9.1
26	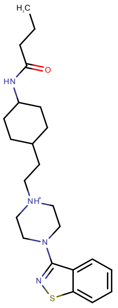	9.1	9.5	9.2
27	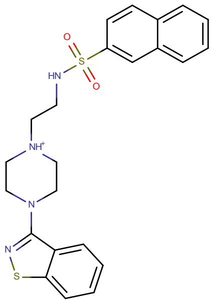	9.1	8.9	9
28	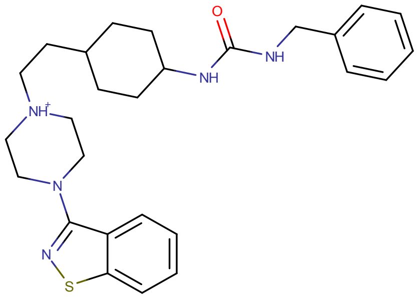	9	9.2	9
29	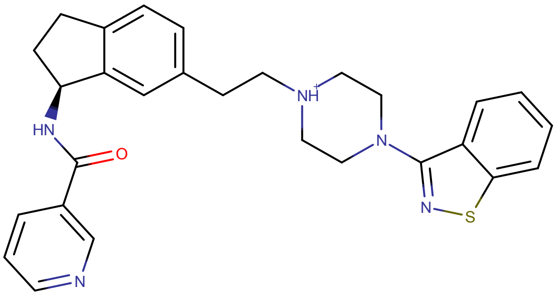	9	8.7	9

30	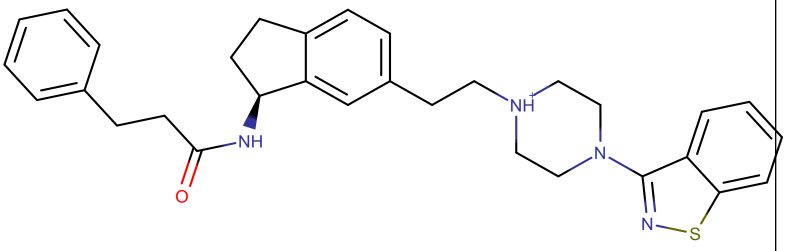	9	9.6	9
31	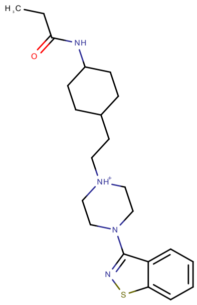	9	9	9
32	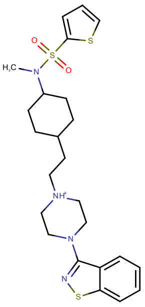	8.9	9.6	8.9
33	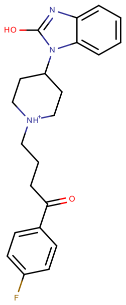	8.9	8.4	8.9
34	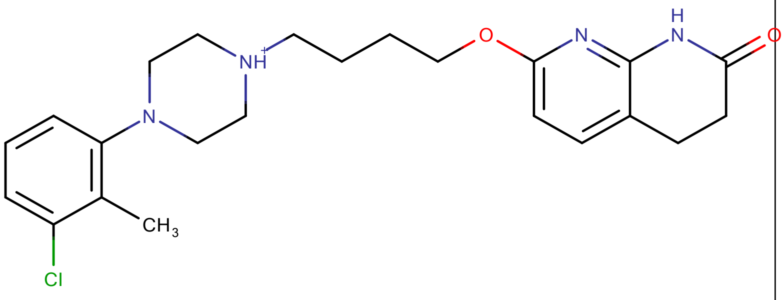	8.8	8.6	8.8

35	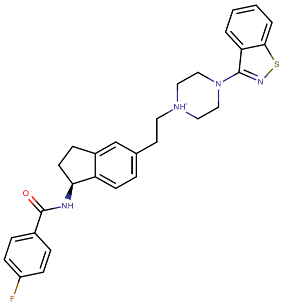	8.8	8.7	8.8
36	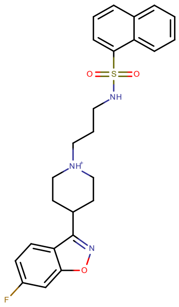	8.8	8.1	8.8
37	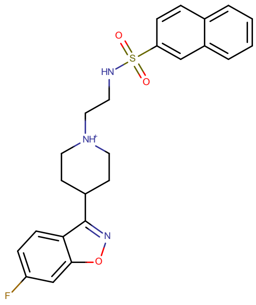	8.7	8.2	8.7
38	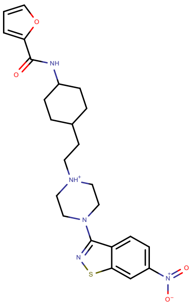	8.7	8.5	8.7
39	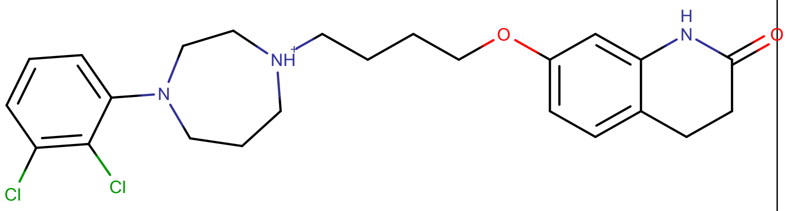	8.7	8.5	8.7

40	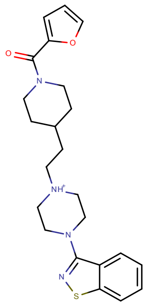	8.7	8.5	8.7
41	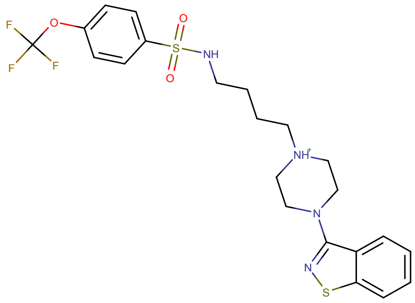	8.7	8.9	8.7
42	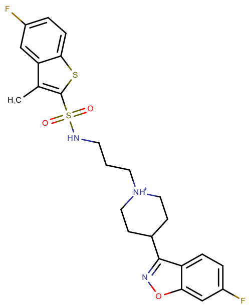	8.6	9.2	8.6
43	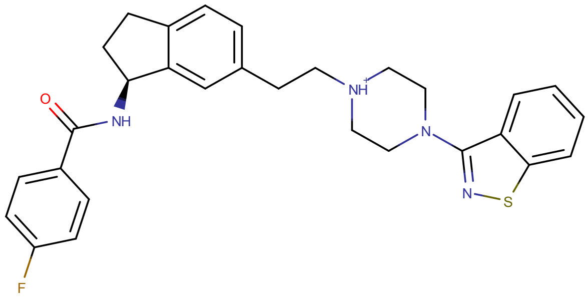	8.6	8.5	8.6
44	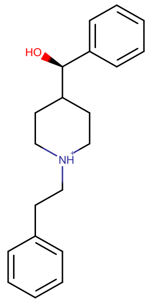	8.6	8.4	8.6

45	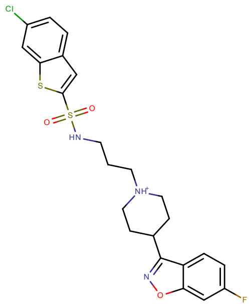	8.6	8.5	8.6
46	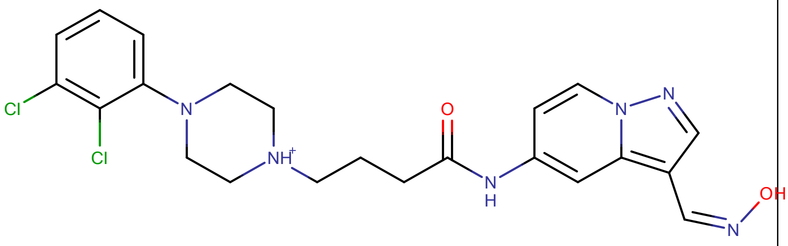	8.6	8.7	8.6
47	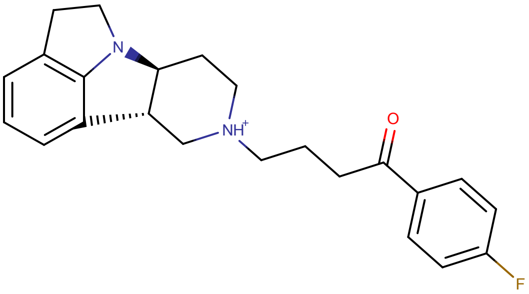	8.5	7.7	8.1
48	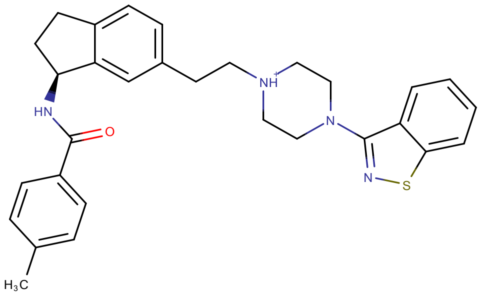	8.5	8.3	8.5
49	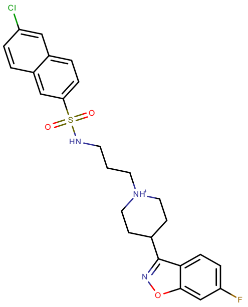	8.5	8.7	8.5

50	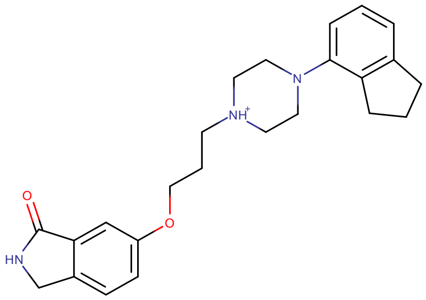	8.5	8.2	8.5
51	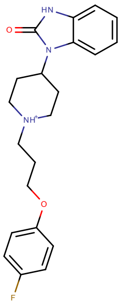	8.5	8.3	8.5
52	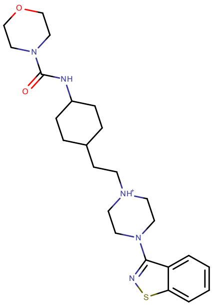	8.5	8.5	8.5
53	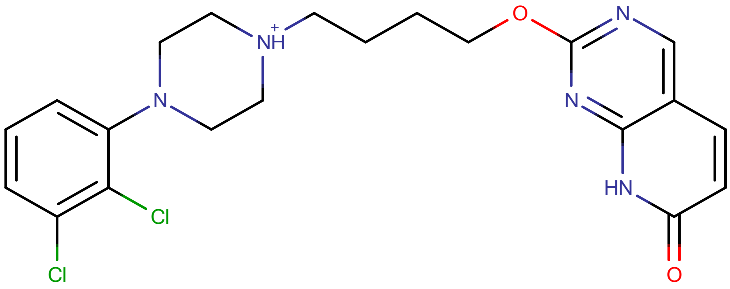	8.5	8.1	8.5
54	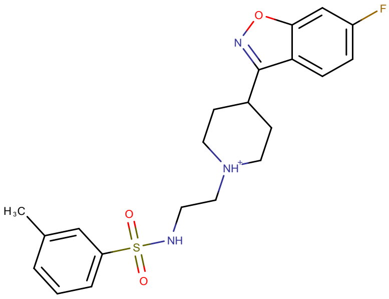	8.5	8.8	8.5

55	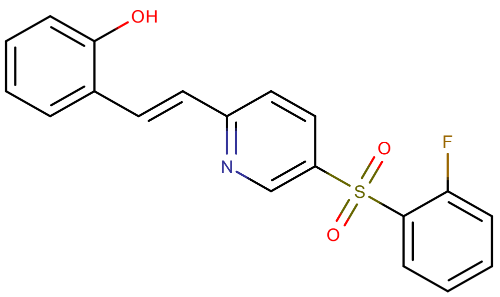	8.4	8.2	8.4
56	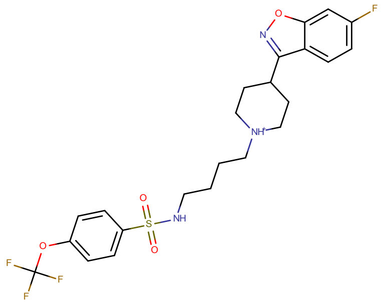	8.4	8.3	8.4
57	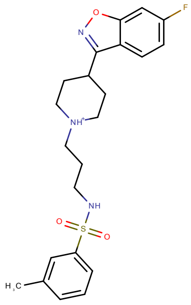	8.4	8.2	8.4
58	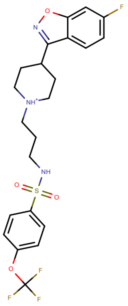	8.4	8.5	8.4
59	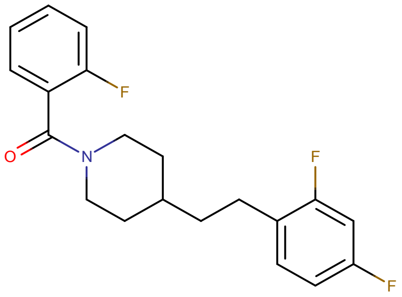	8.4	7.8	8.4

60	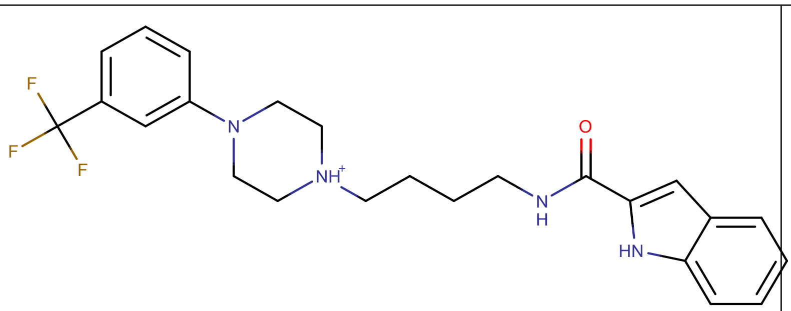	8.4	7.5	8.4
61	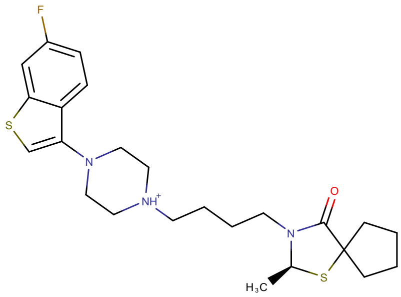	8.4	7.8	8.4
62	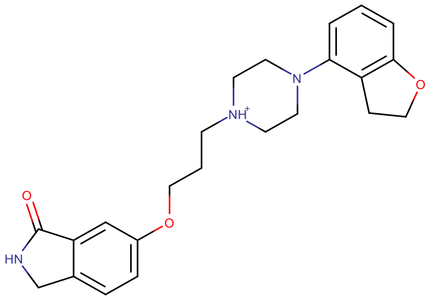	8.4	8.3	8.4
63	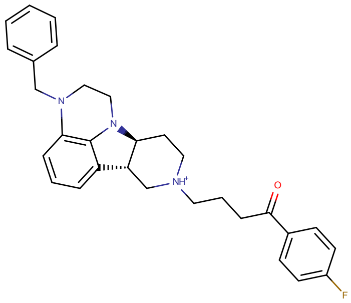	8.3	8	8.3
64	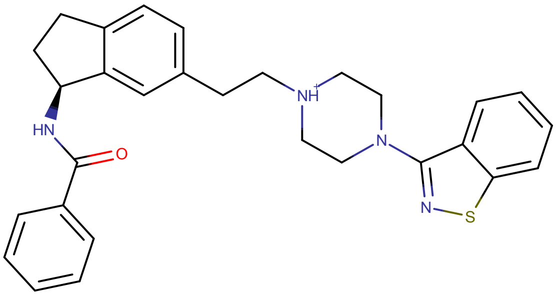	8.3	8.3	8.3

65	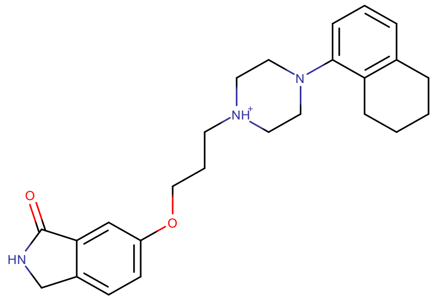	8.3	8.6	8.3
66	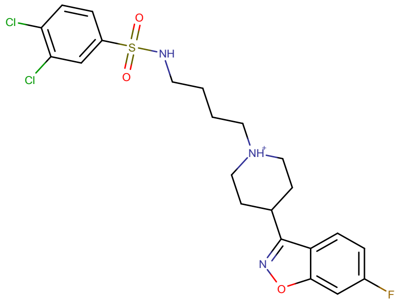	8.3	8.6	8.3
67	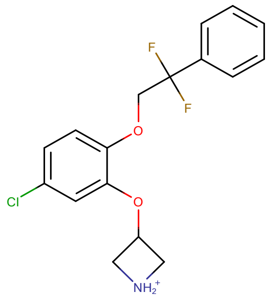	8.3	8	8.3
68	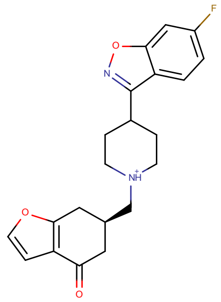	8.3	8.4	8.3
69	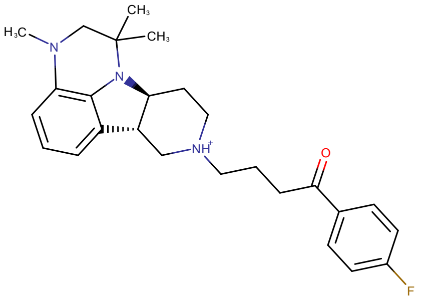	8.3	7.9	8.3

70	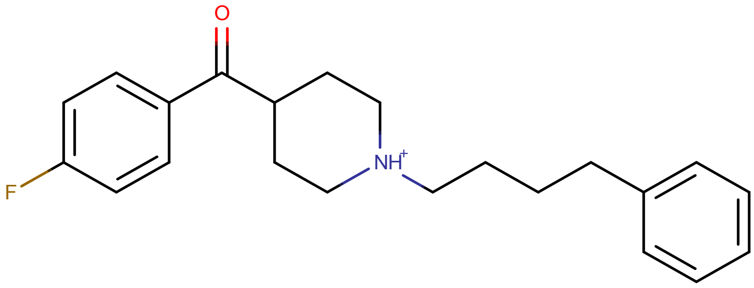	8.3	7.7	7.7
71	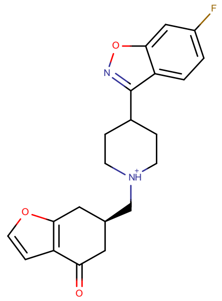	8.3	8.4	8.3
72	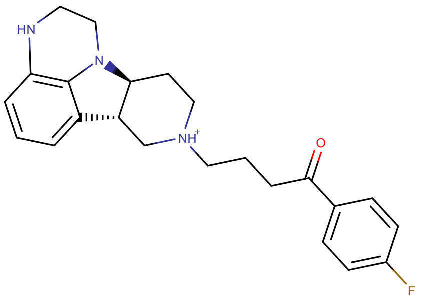	8.3	8.4	8.3
73	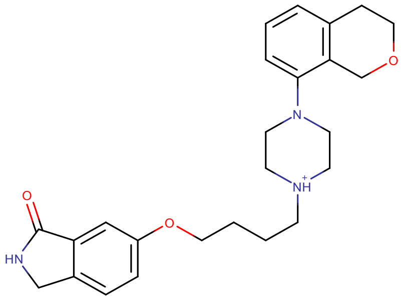	8.3	7.7	8.3
74	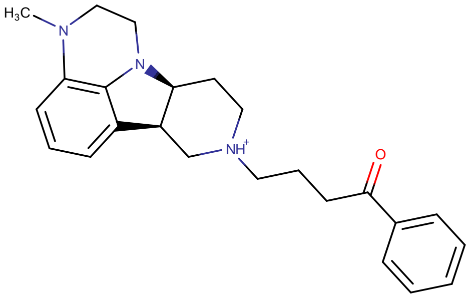	8.2	8.5	8.2

75	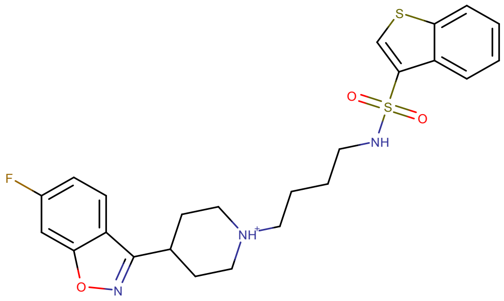	8.2	8.6	8.2
76	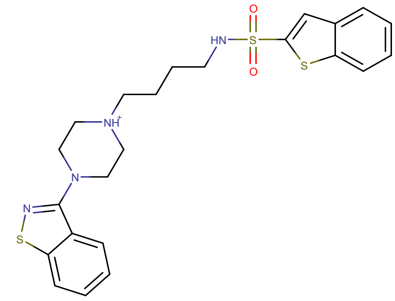	8.2	8.3	8.2
77	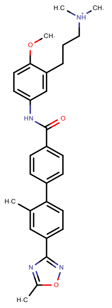	8.2	7.8	8.2
78	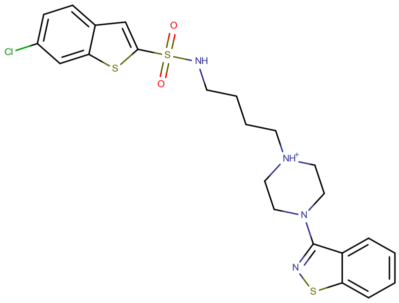	8.2	8.9	8.2
79	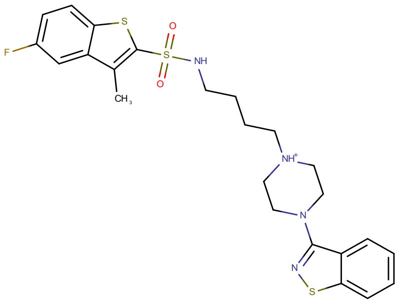	8.2	8.7	8.2

80	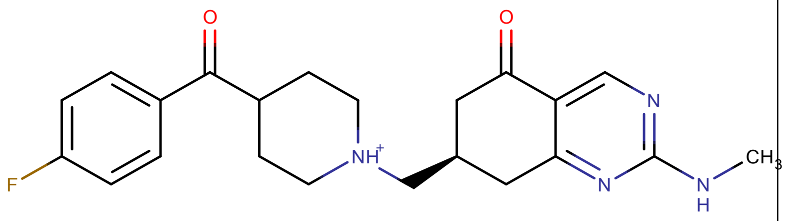	8.2	8.3	8.2
81	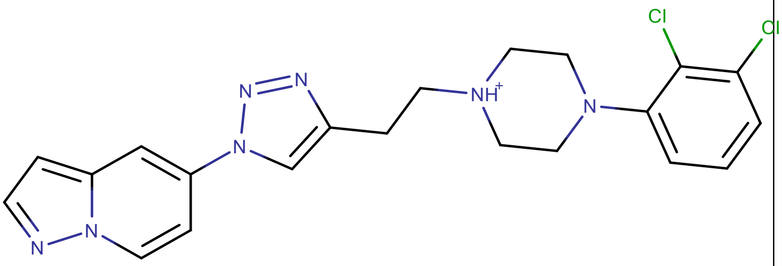	8.2	7.9	8.1
82	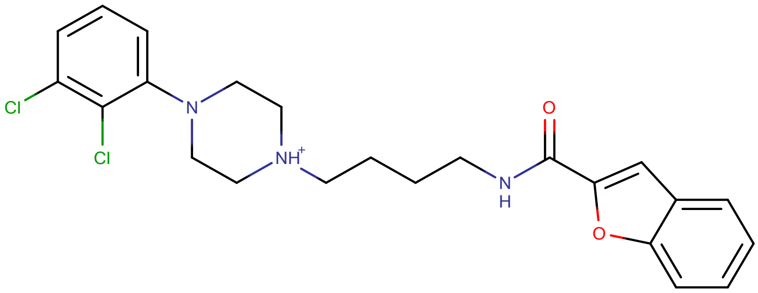	8.1	7.9	8.1
83	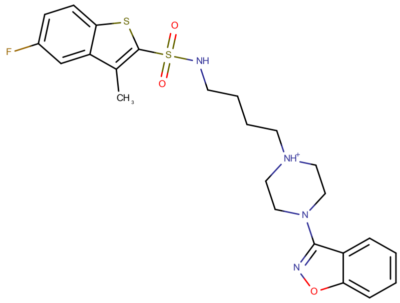	8.1	7.6	8.1
84	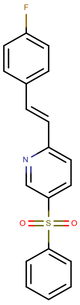	8.1	7.7	8

85	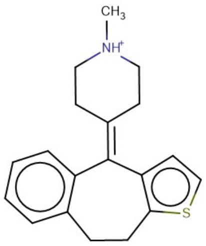	8.1	7.5	8
86	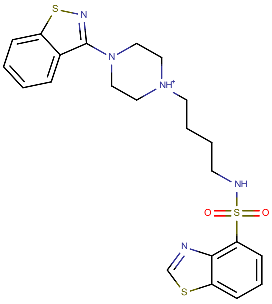	8.1	7.7	8.1
87	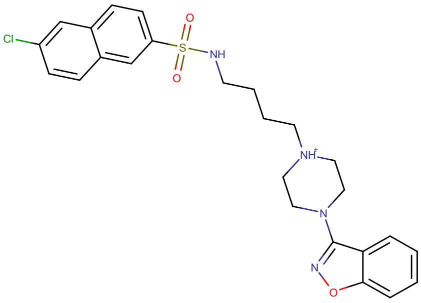	8.1	8.3	8.1
88	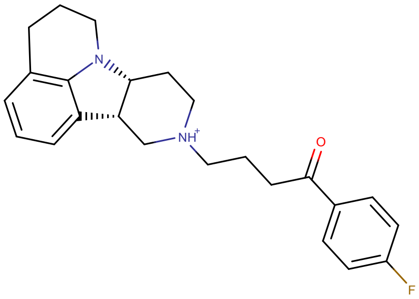	8.1	8	8.1
89	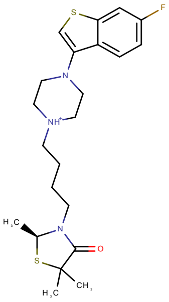	8.1	7.6	8.1

90	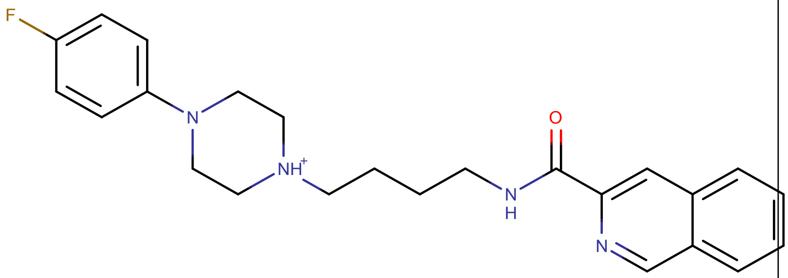	8.1	7.7	8.1
91	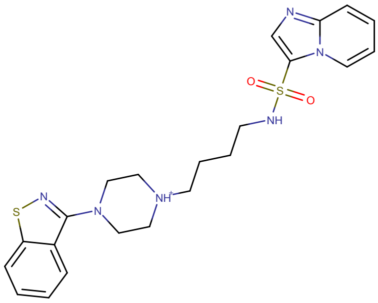	8.1	7.7	8.1
92	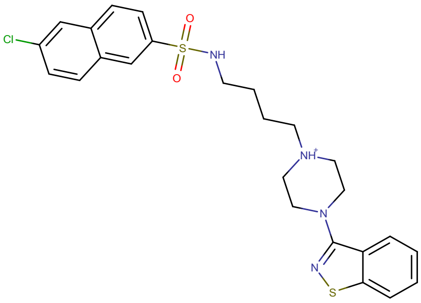	8.1	7.9	8.1
93	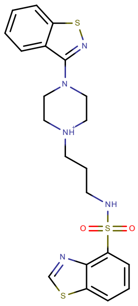	8.1	7.8	8.1
94	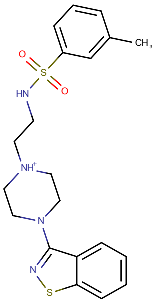	8.1	8.2	8.1

95	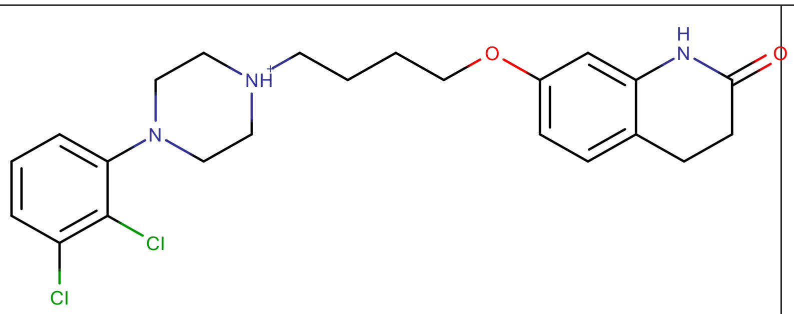	8.1	7.6	8.1
96	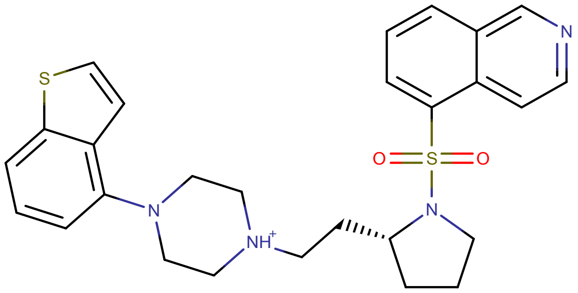	8.1	8.5	8.1
97	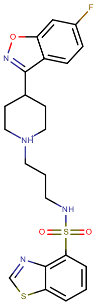	8	7.8	8
98	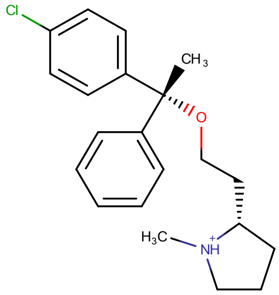	8	7.6	8
99	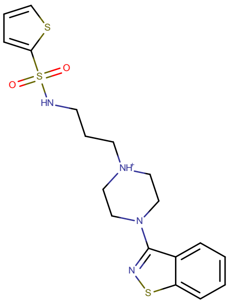	8	8.2	8

100	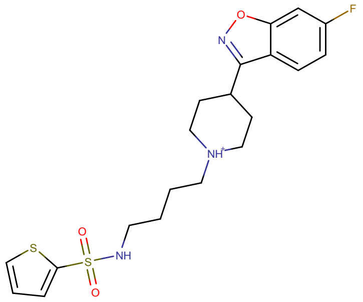	8	8.4	8
101	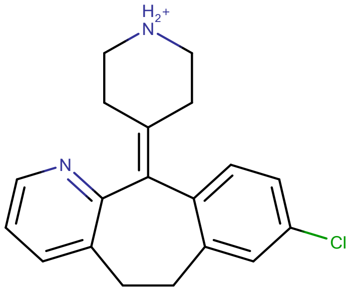	8	7.4	7.9
102	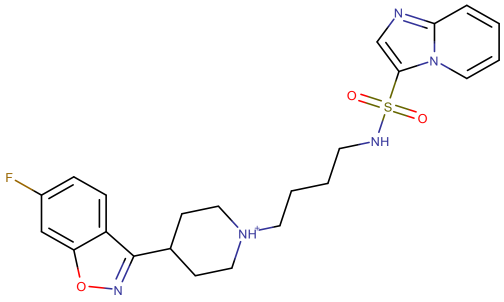	8	7.7	8
103	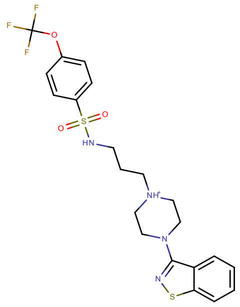	8	8.2	8
104	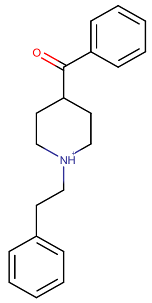	8	8.5	8

105	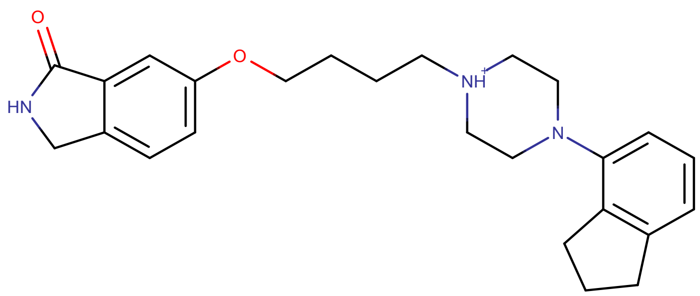	8	7.8	8
106	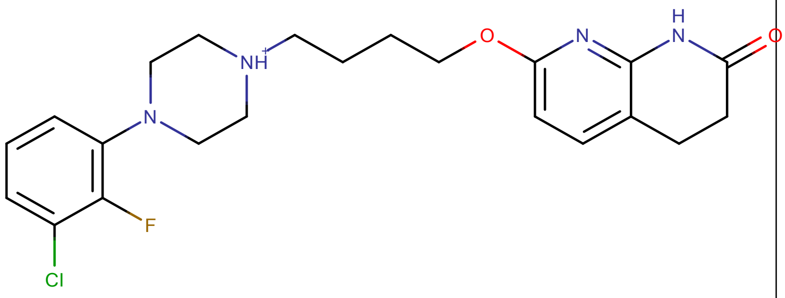	8	7.4	8
107	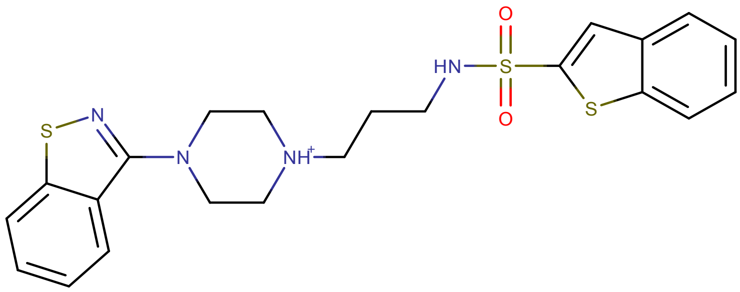	8	8.4	8
108	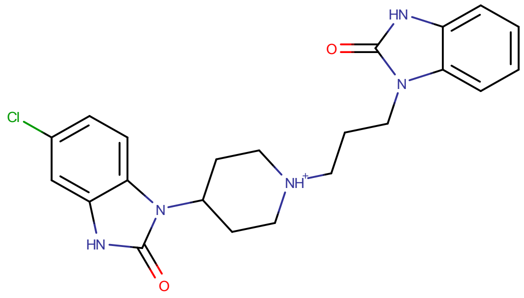	8	8.1	8
109	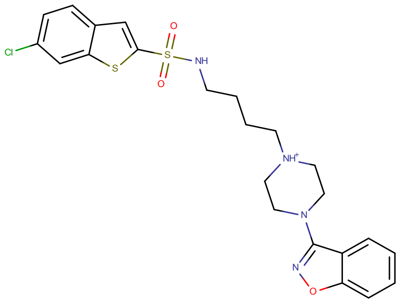	8	7.8	8

110	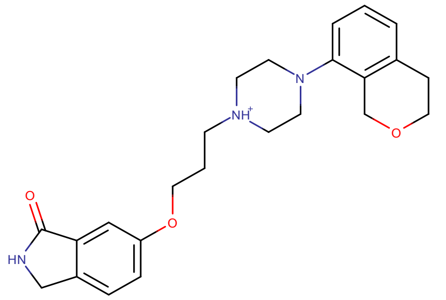	8	7.6	8
111	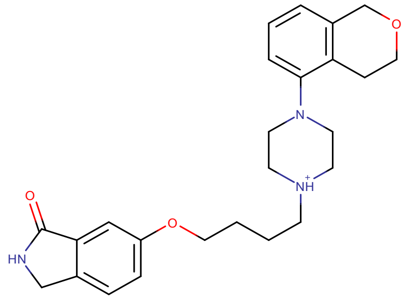	8	8.2	7.9
112	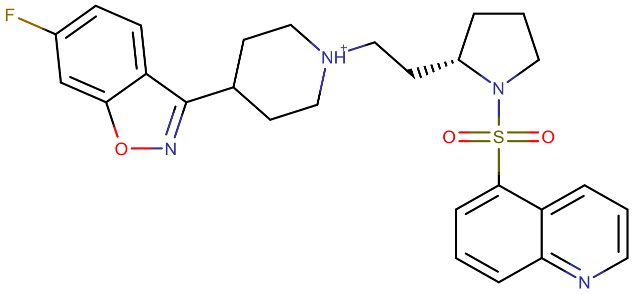	7.9	7.6	7.9
113	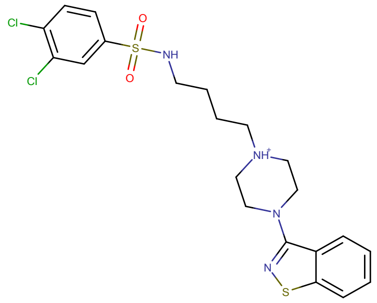	7.9	8.2	7.9
114	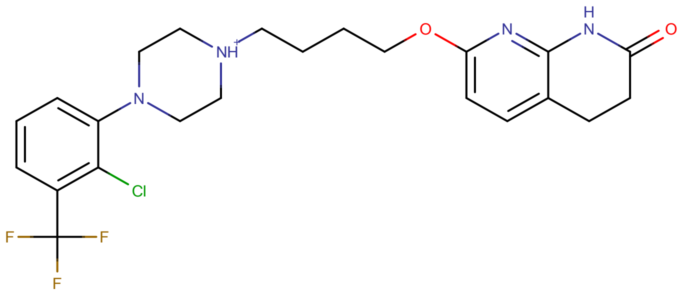	7.9	7.7	7.9

115	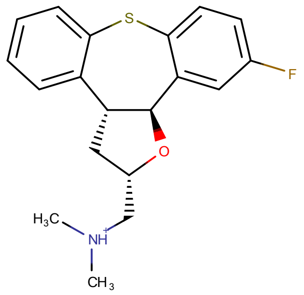	7.9	7.4	7.9
116	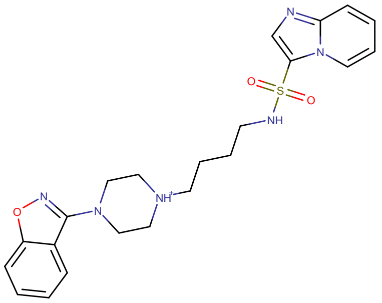	7.9	7.9	7.9
117	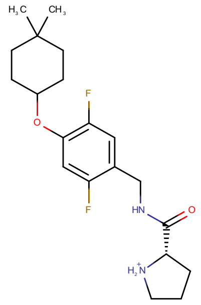	7.9	7.7	7.9
118	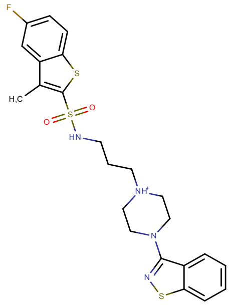	7.9	7.8	7.8
119	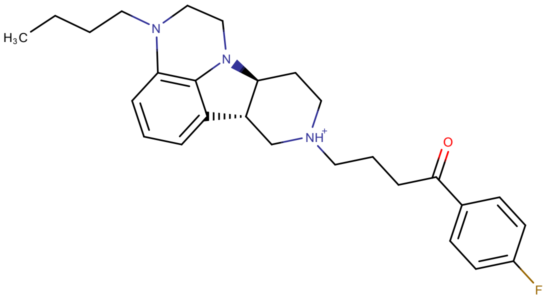	7.9	8.2	7.9

120	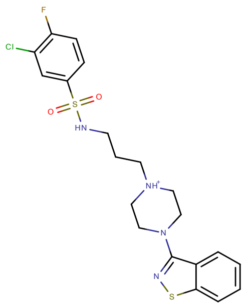	7.9	8.4	7.9
121	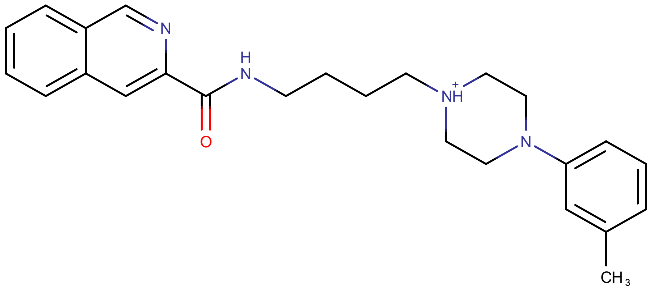	7.8	7.4	7.8
122	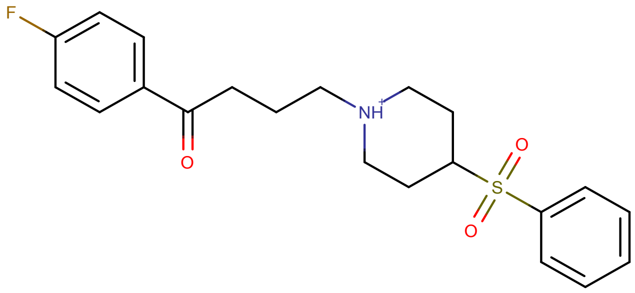	7.8	7.9	7.8
123	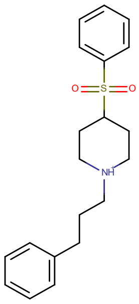	7.8	8.2	7.8
124	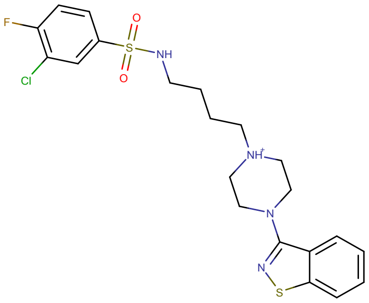	7.8	7.5	7.8

125	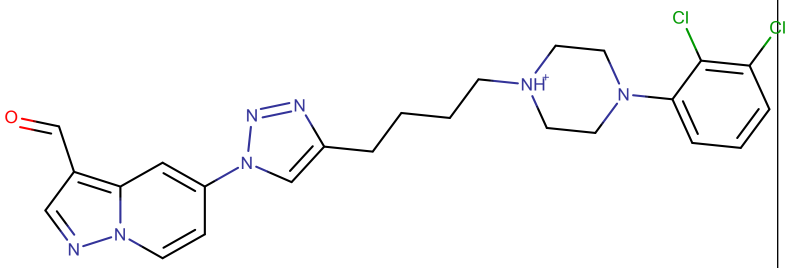	7.8	8.2	7.8
126	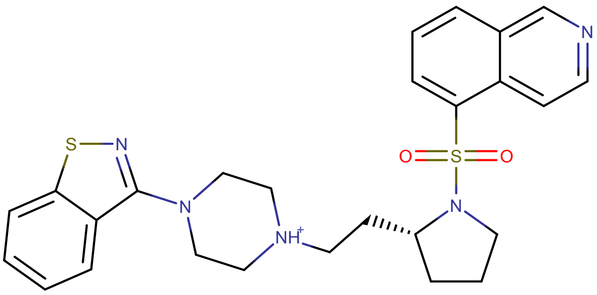	7.8	8	7.8
127	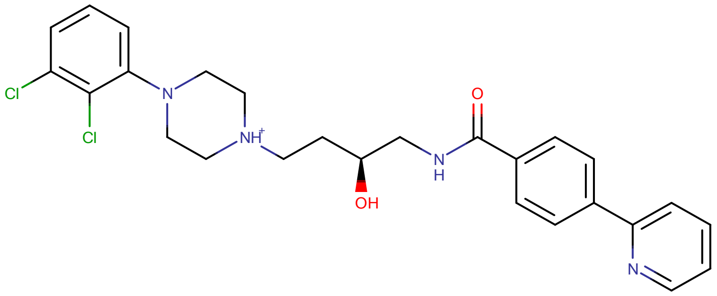	7.8	7.8	7.8
128	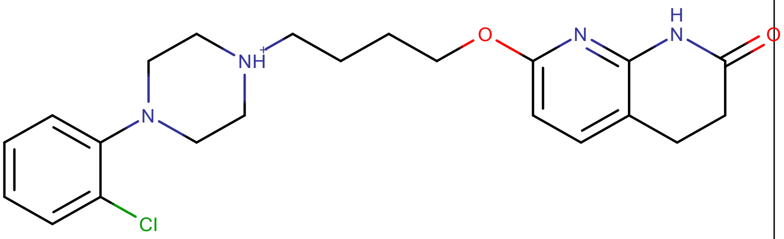	7.8	7.5	7.8
129	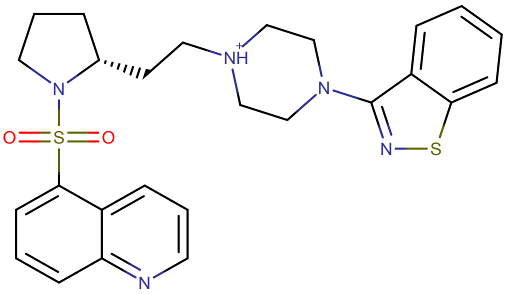	7.8	8.6	7.8

130	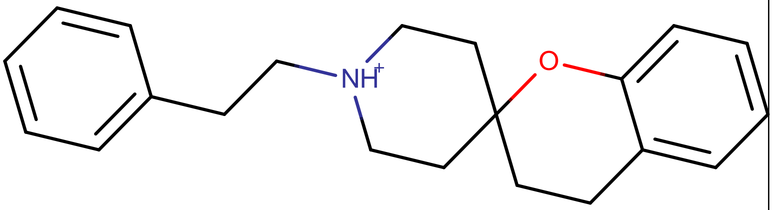	7.8	8.1	7.8
131	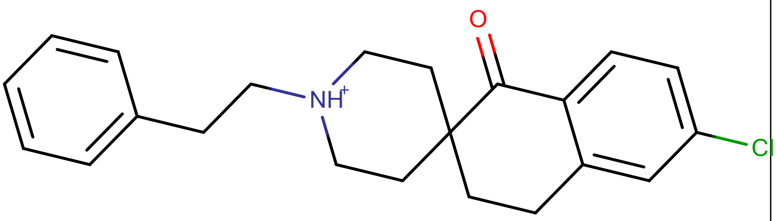	7.8	7.9	7.8
132	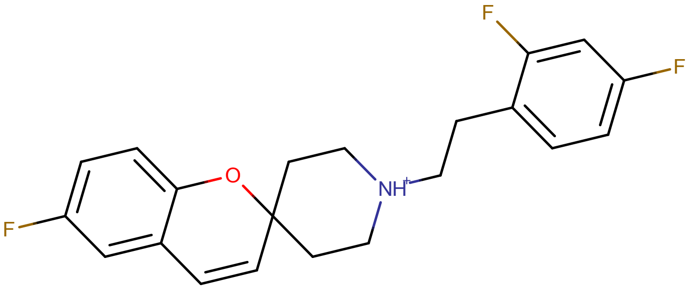	7.8	7.3	7.5
133	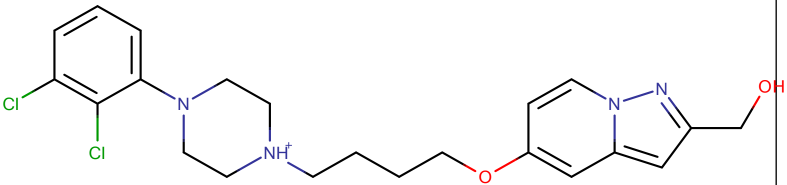	7.8	7.9	7.8
134	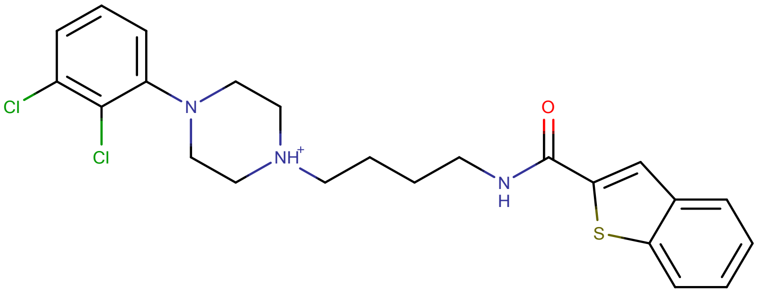	7.8	7.8	7.8

135	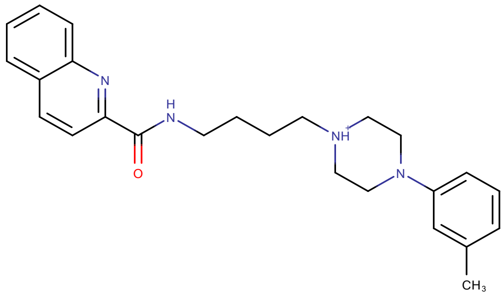	7.8	7.4	7.8
136	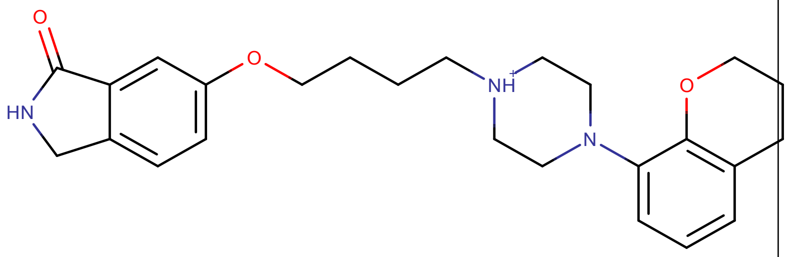	7.8	7.4	7.8
137	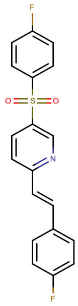	7.8	7.2	7.6
138	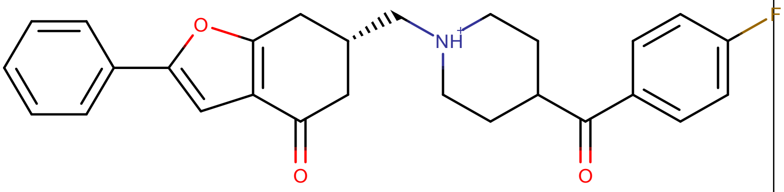	7.8	7.8	7.8
139	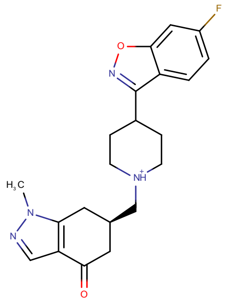	7.8	7.5	7.8

140	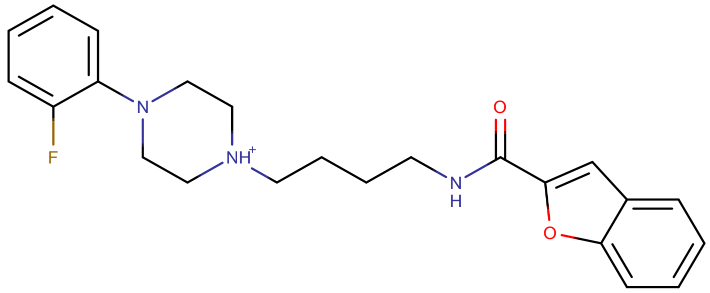	7.7	7.4	7.7
141	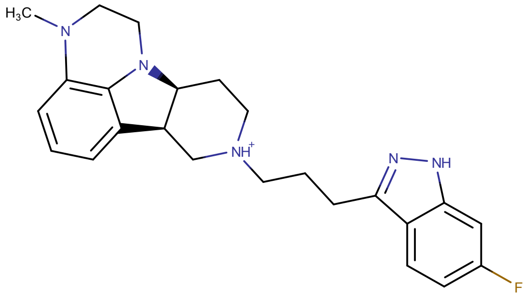	7.7	7.7	7.7
142	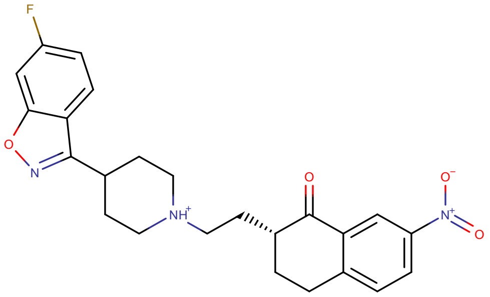	7.7	8	7.7
143	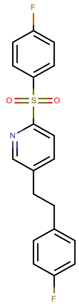	7.7	8	7.7
144	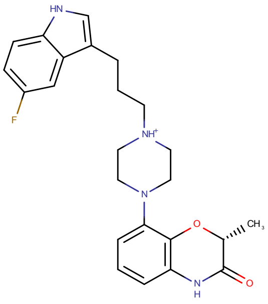	7.7	7.3	7.7

145	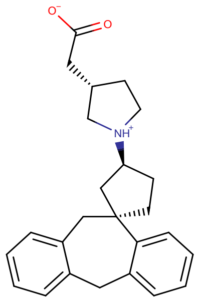	7.7	6.9	7.2
146	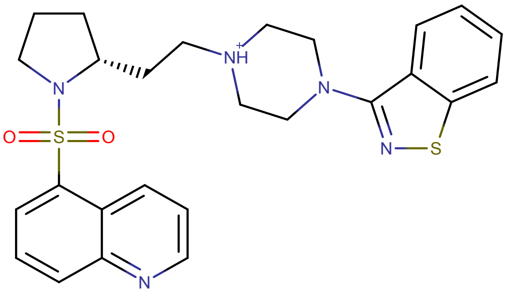	7.7	7.8	7.7
147	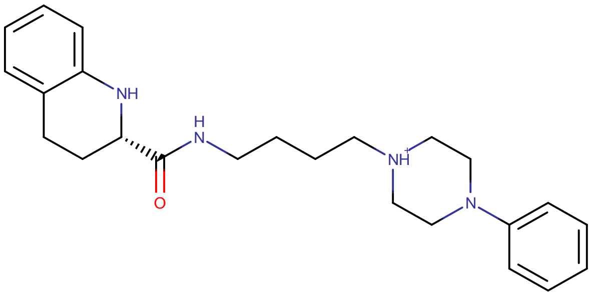	7.7	7.2	7.7
148	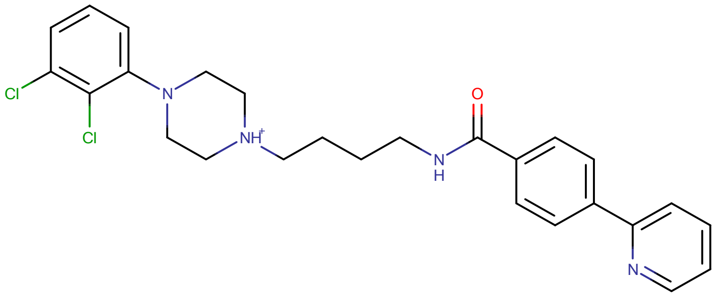	7.6	7.6	7.6
149	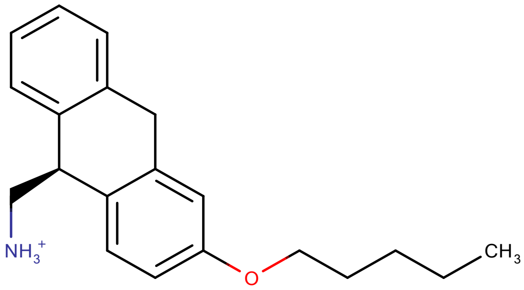	7.6	7.3	7.5

150	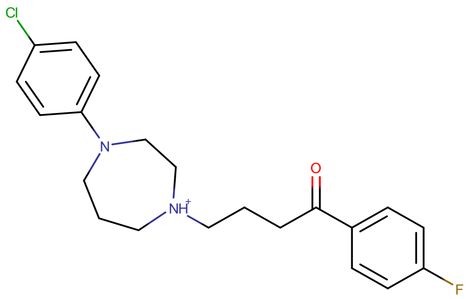	7.6	7.5	7.6
151	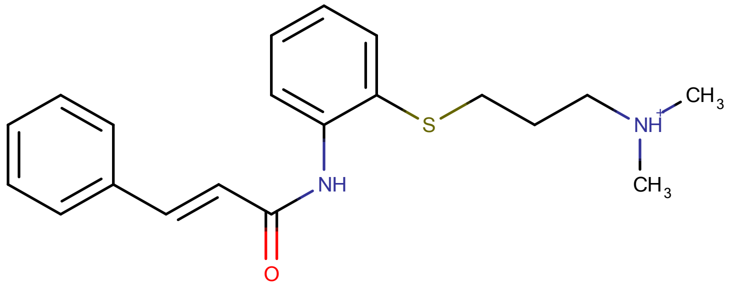	7.6	7.4	7.6
152	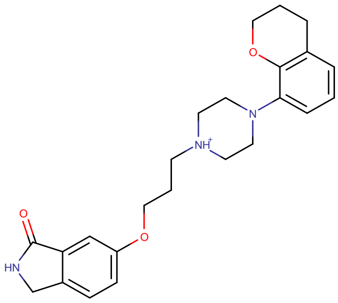	7.6	7.4	7.6
153	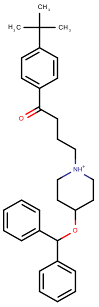	7.6	8.2	7.6
154	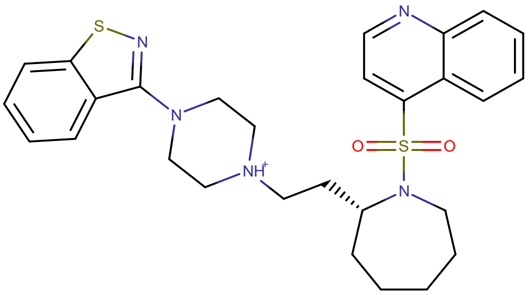	7.6	8	7.6

155	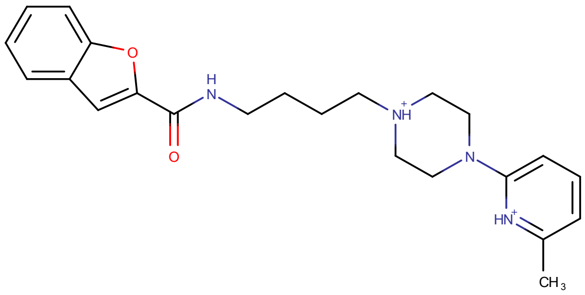	7.6	8	7.6
156	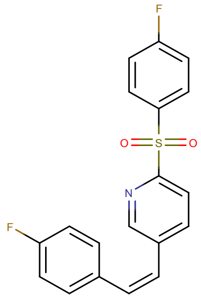	7.6	7.1	7.5
157	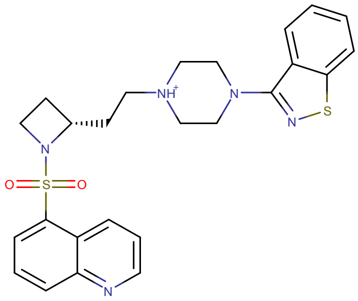	7.5	7.3	7.5
158	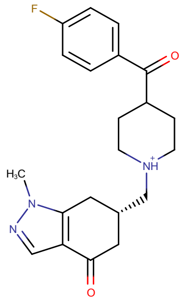	7.5	7.4	7.5
159	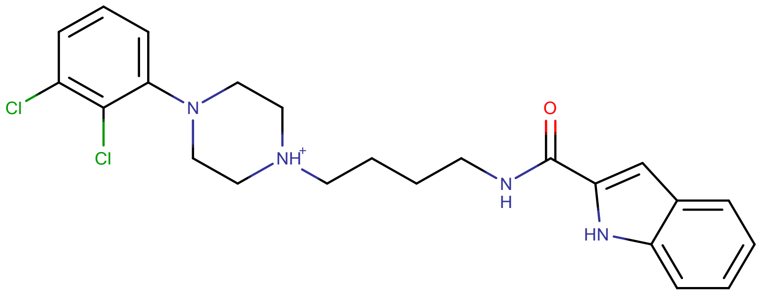	7.5	7.4	7.5

160	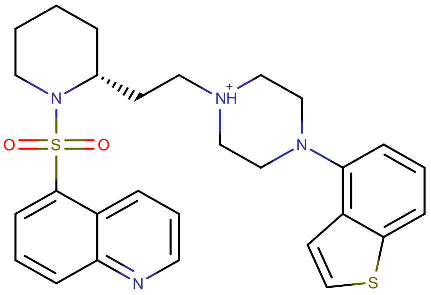	7.5	7.2	7.5
161	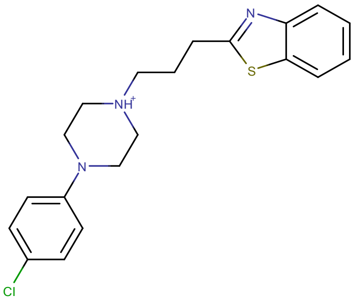	7.4	7.5	7.4
162	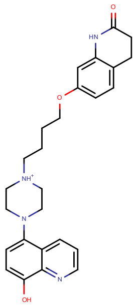	7.4	7.3	7.4
163	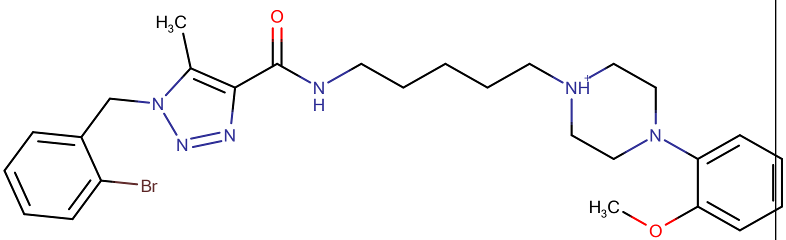	7.4	7.1	7.4
164	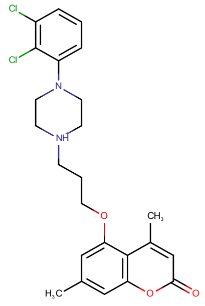	7.4	7.1	7.4

165	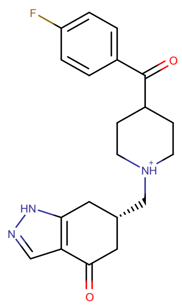	7.4	6.8	7.4
166	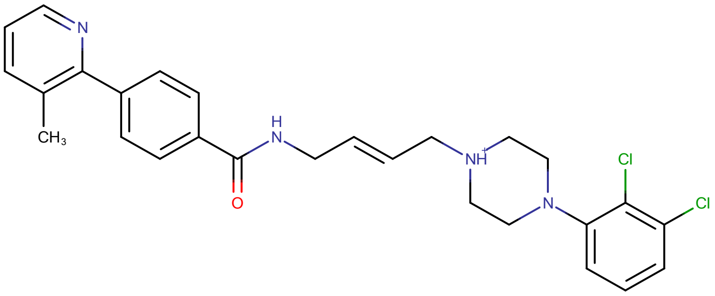	7.3	7.9	7.3
167	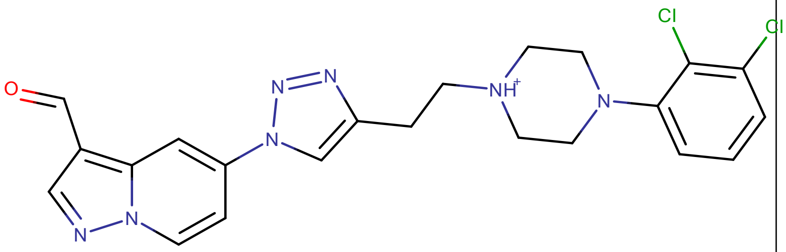	7.3	7.2	7.3
168	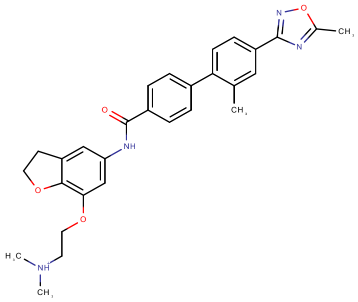	7.3	7.1	7.3
169	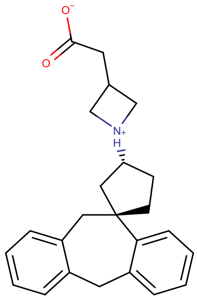	7.3	7.2	7.3

170	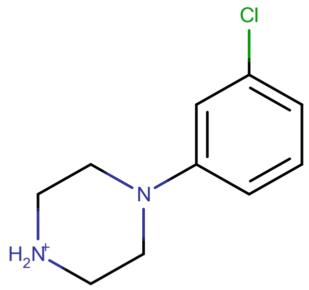	7.3	6.9	7
171	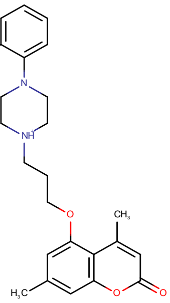	7.3	7	7.3
172	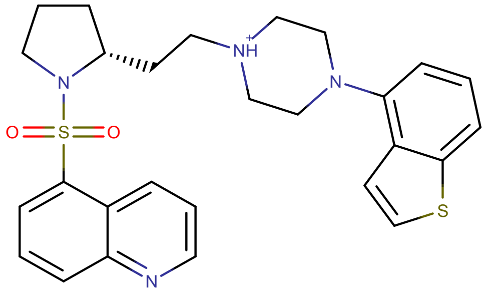	7.3	7.3	7.3
173	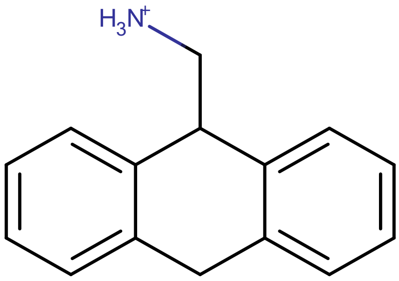	7.2	7	7.2
174	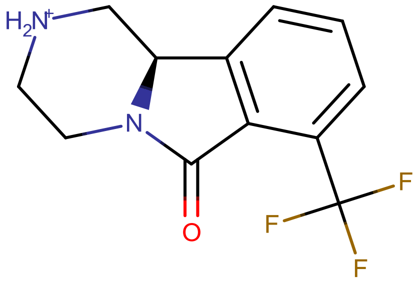	7.2	6.5	6.9

175	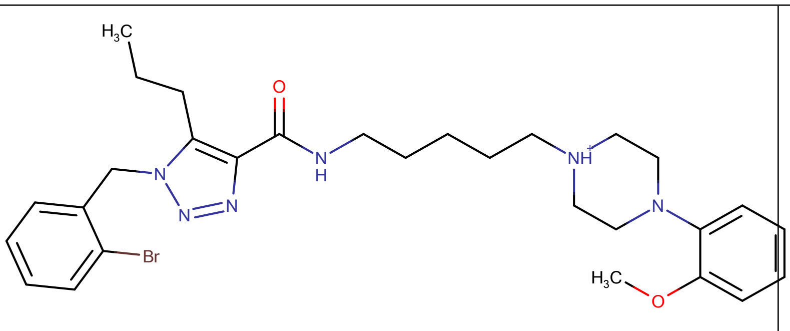	7.2	7.7	7.2
176	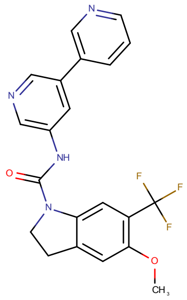	7.2	6.9	7.2
177	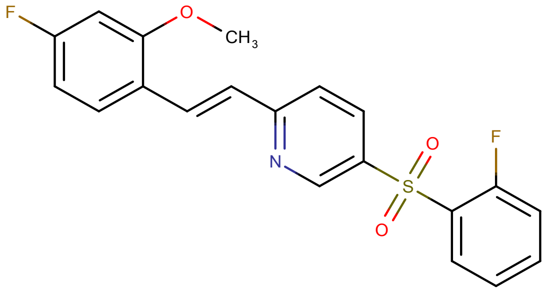	7.2	7.9	7.7
178	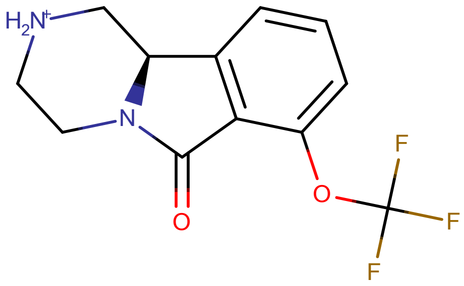	7.2	6.7	6.8
179	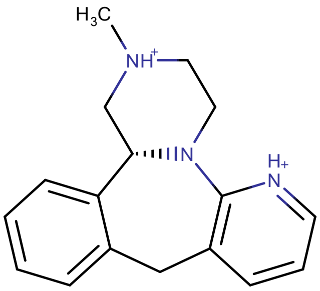	7.2	7.1	7.2

180	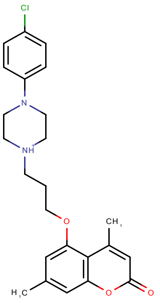	7.2	7.4	7.2
181	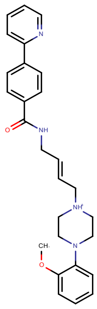	7.1	7.2	7.1
182	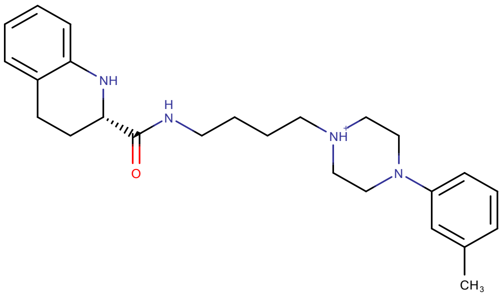	7.1	8	7.1
183	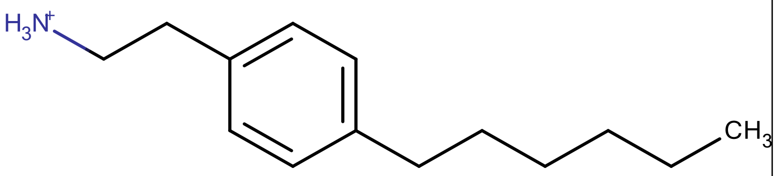	7.1	7.1	7.1
184	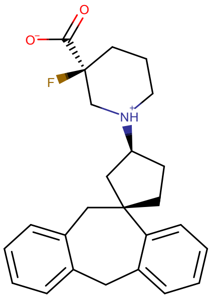	7.1	6.5	7

185	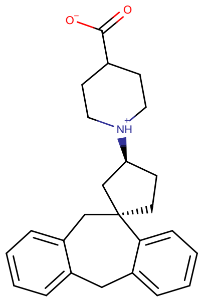	7.1	7.1	7.1
186	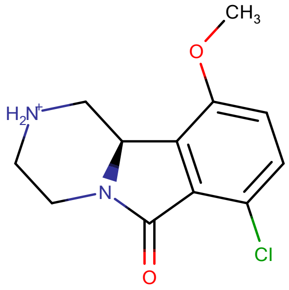	7.1	6.8	7.1
187	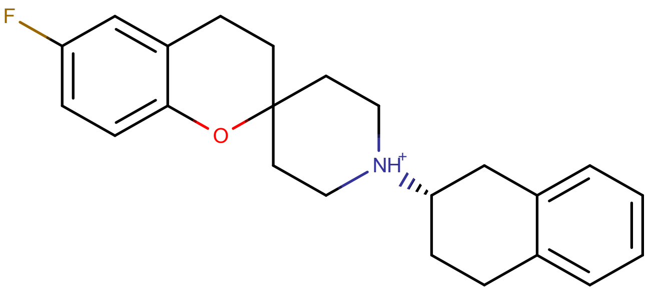	7	7.6	7.3
188	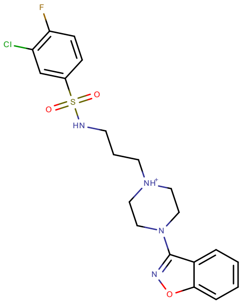	7	6.9	7
189	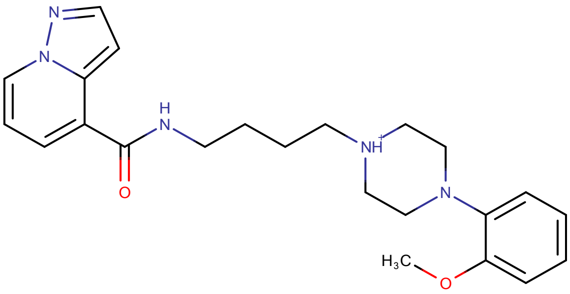	7	7.2	7

190	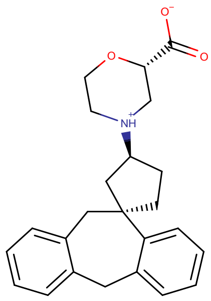	7	6.7	7
191	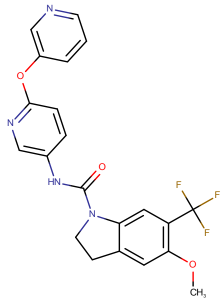	7	6.9	7
192	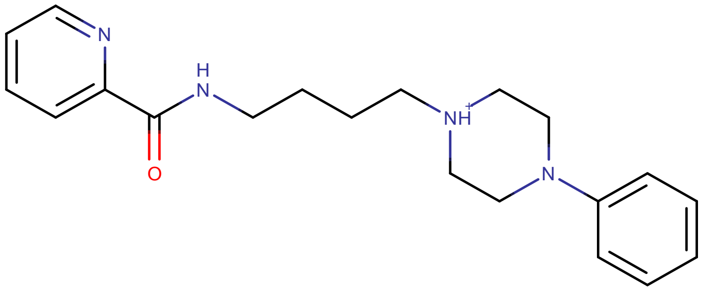	7	7	7
193	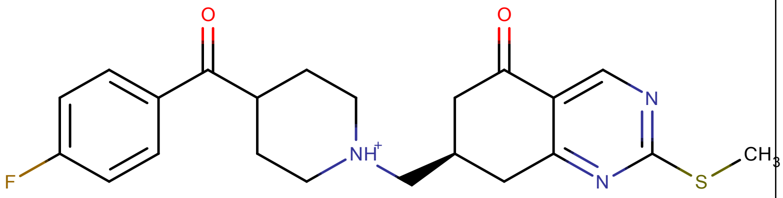	7	7.5	7
194	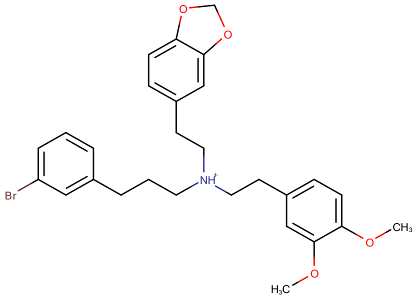	7	6.8	7

195	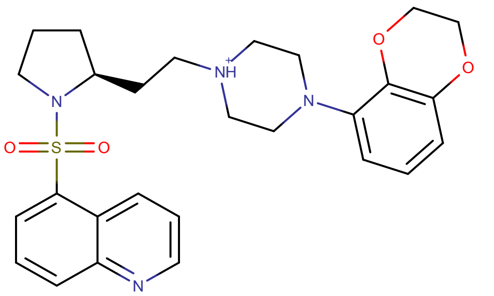	7	7	7
196	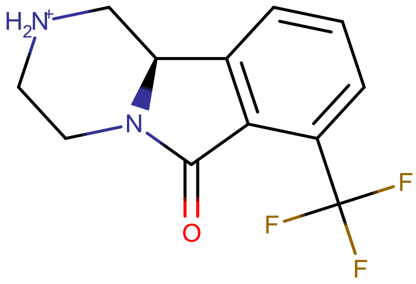	7	6.5	7
197	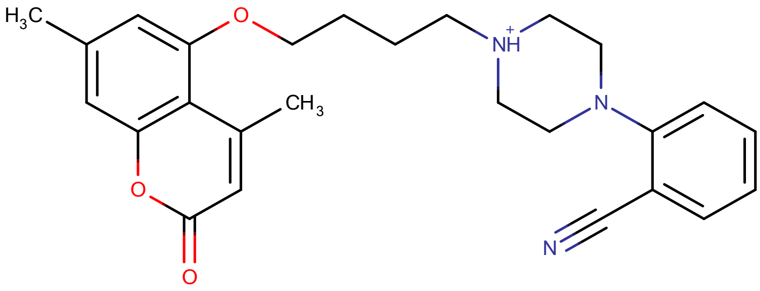	7	7.1	7
198	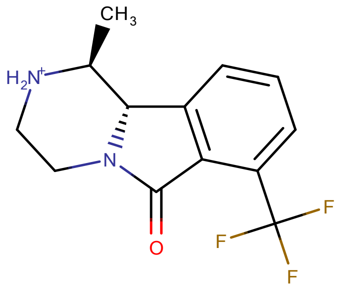	6.9	6.4	6.8
199	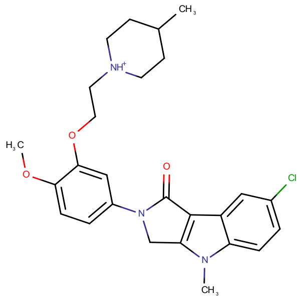	6.9	6.9	6.9

200	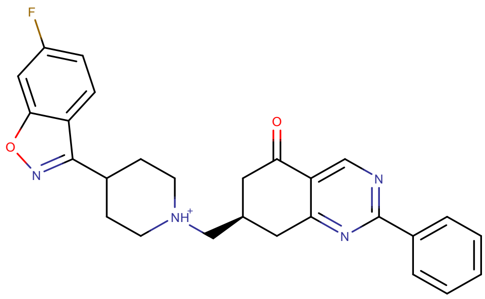	6.9	7.3	6.9
201	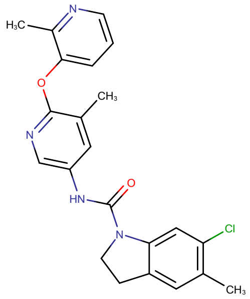	6.9	6.7	6.9
202	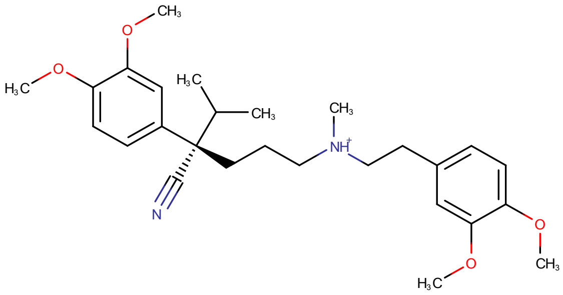	6.9	6.4	6.9
203	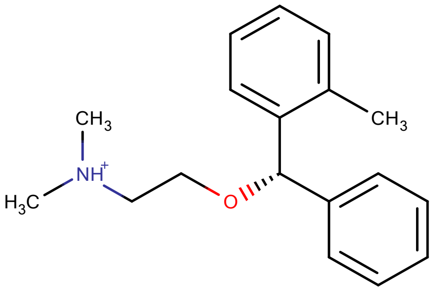	6.9	6.8	6.9
204	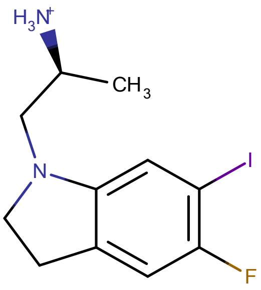	6.9	6.8	6.9

205	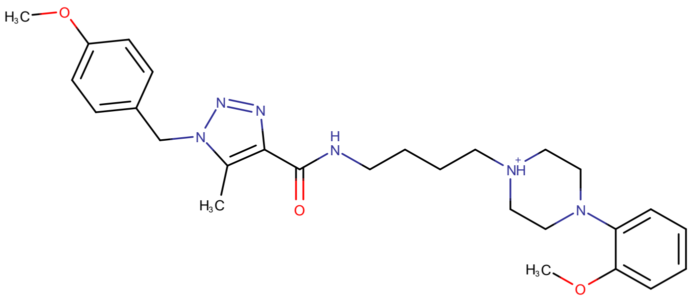	6.9	6.5	6.9
206	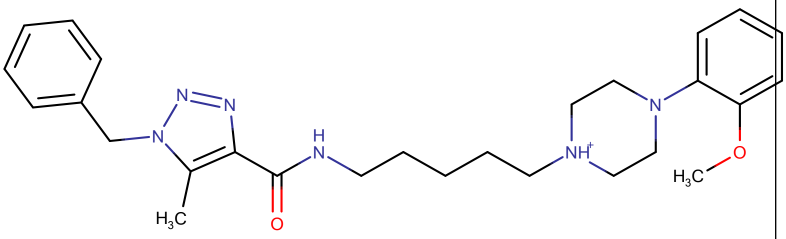	6.9	6.5	6.8
207	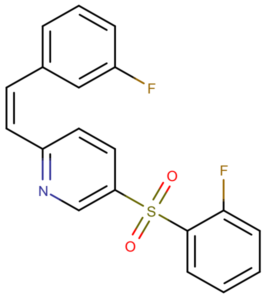	6.8	6.6	6.8
208	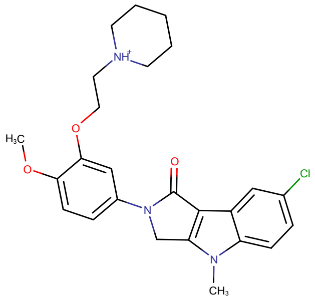	6.8	6.9	6.8
209	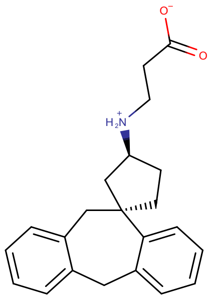	6.8	7.1	6.8

210	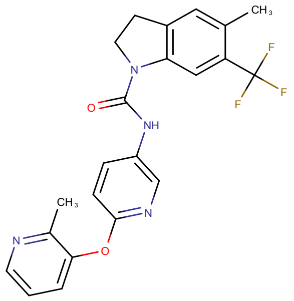	6.8	6.5	6.8
211	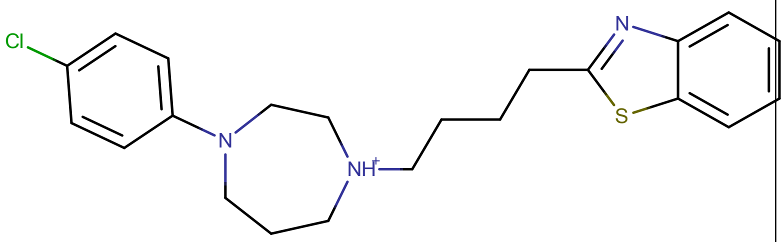	6.8	7.4	7.2
212	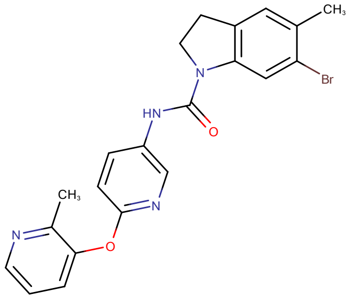	6.8	6.7	6.8
213	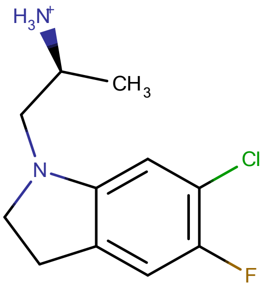	6.8	6.5	6.8
214	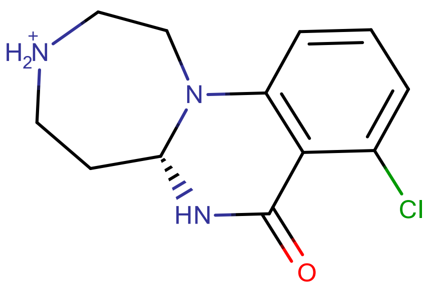	6.8	6.2	6.8

215	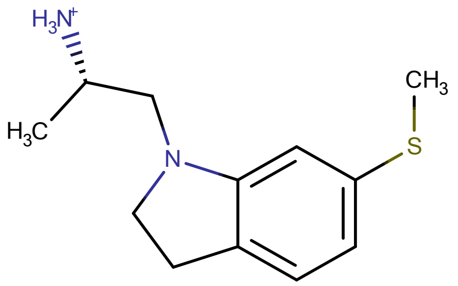	6.8	6.6	6.8
216	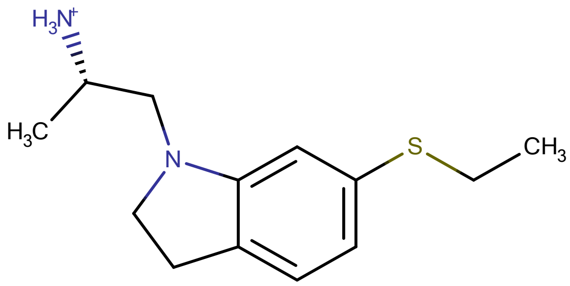	6.7	6.8	6.7
217	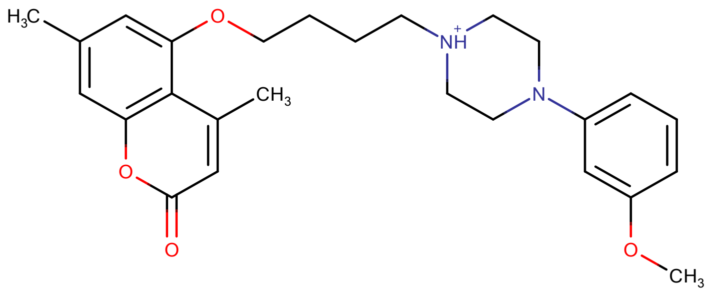	6.7	6.8	6.7
218	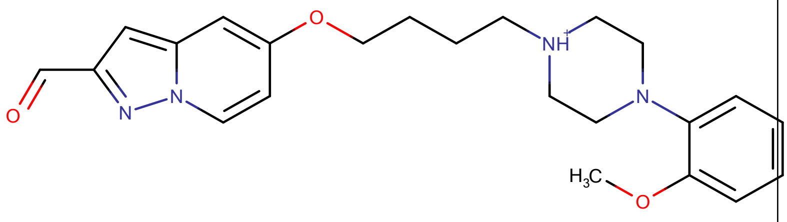	6.7	7.2	6.7
219	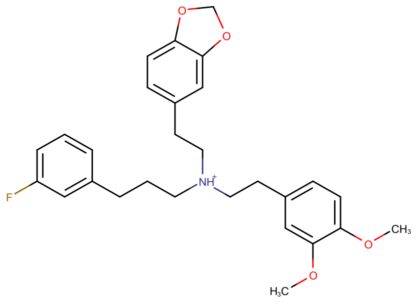	6.7	7.2	6.7

220	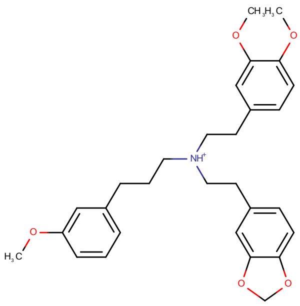	6.7	7.1	6.7
221	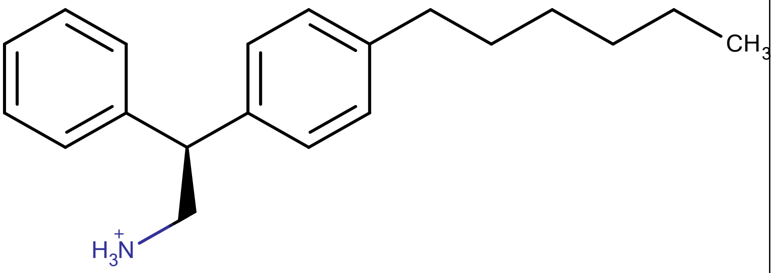	6.7	7.1	6.7
222	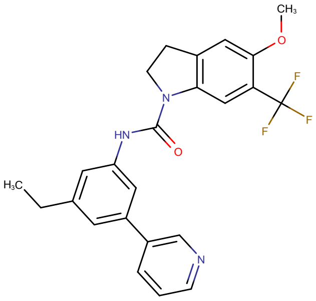	6.7	6.6	6.7
223	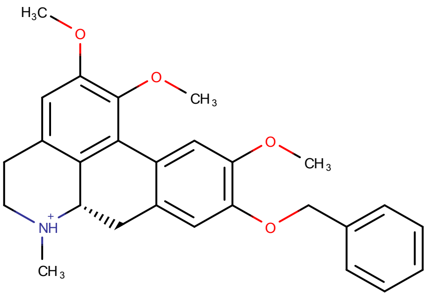	6.7	6.5	6.7
224	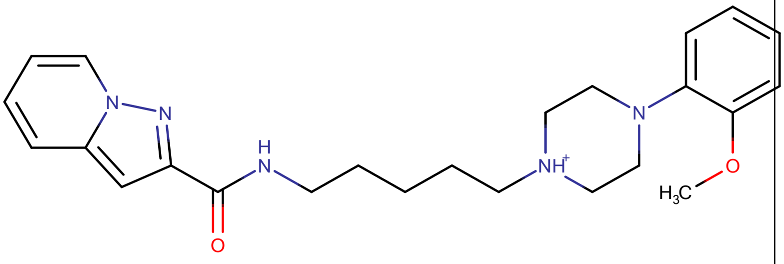	6.7	6.8	6.7

225	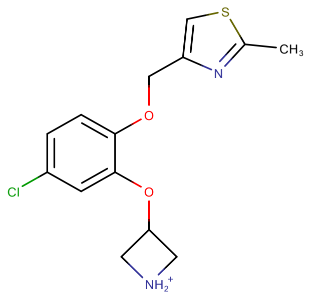	6.7	6.7	6.7
226	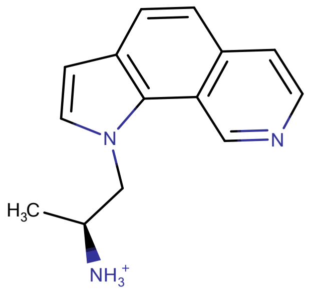	6.6	6.8	6.8
227	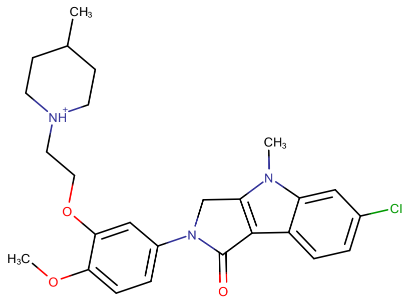	6.6	6.2	6.6
228	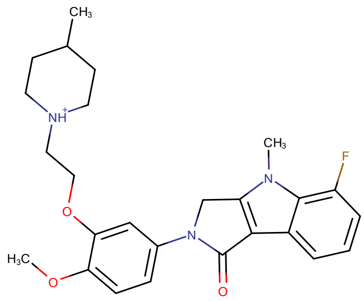	6.6	7.2	6.6
229	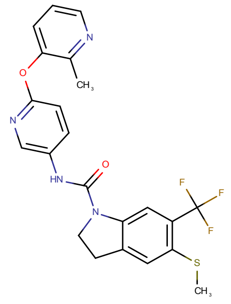	6.6	6.4	6.6

230	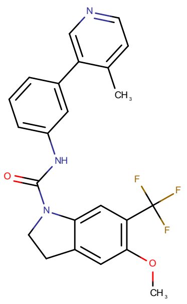	6.6	6.3	6.6
231	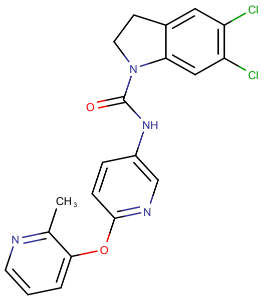	6.6	6.2	6.6
232	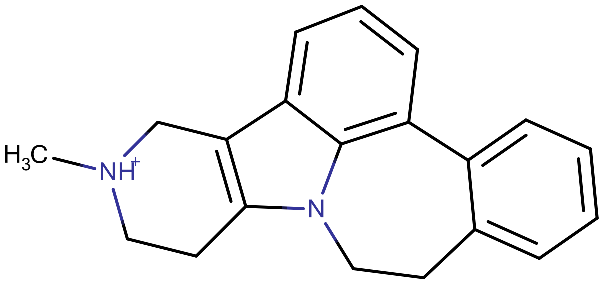	6.6	6.4	6.6
233	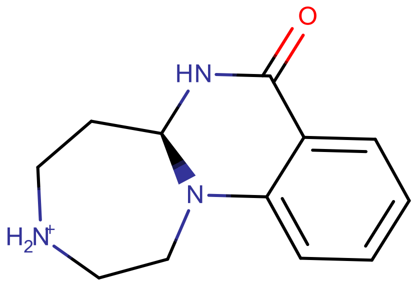	6.6	6.6	6.6
234	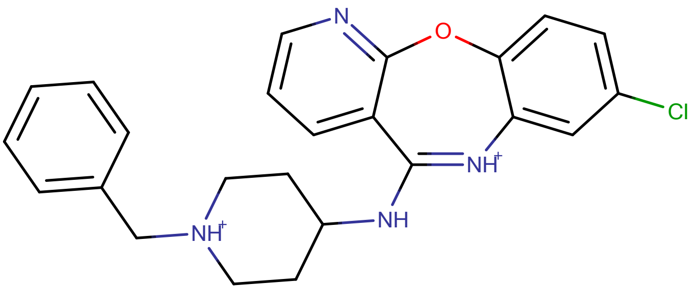	6.6	6.9	6.6

235	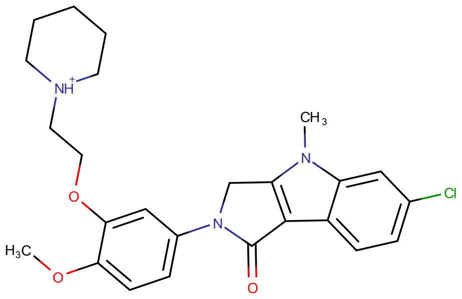	6.5	6.4	6.5
236	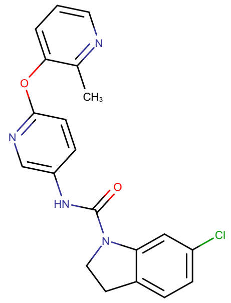	6.5	6.2	6.5
237	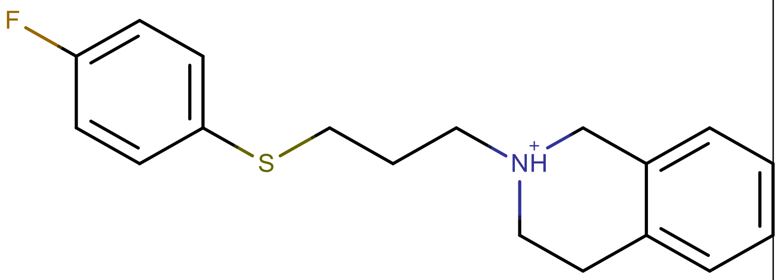	6.5	7.2	7.2
238	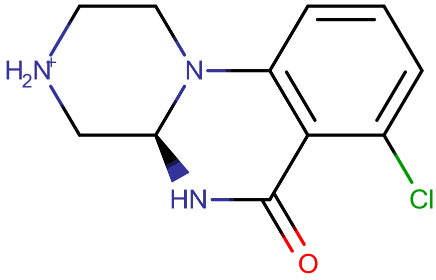	6.5	6.3	6.5
239	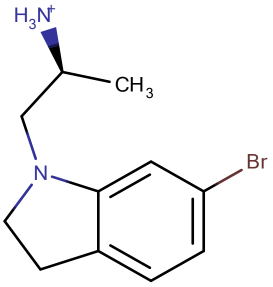	6.5	6.8	6.7

240	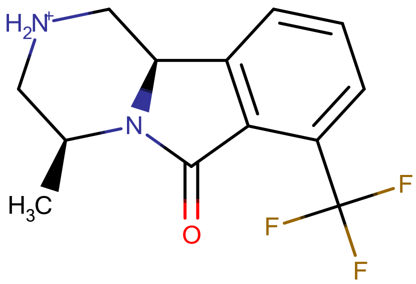	6.4	6.7	6.6
241	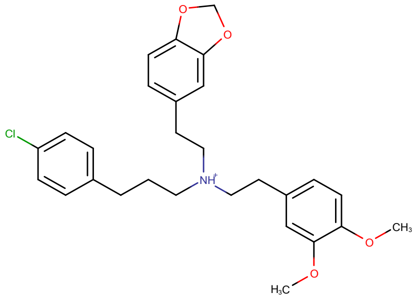	6.4	6.4	6.4
242	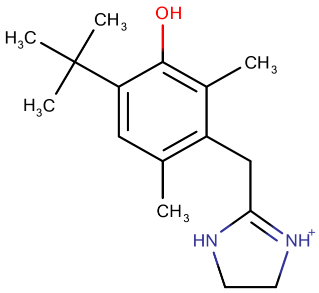	6.4	7	6.9
243	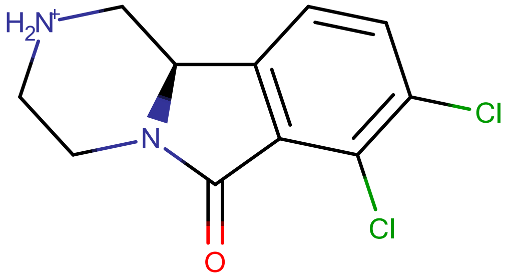	6.4	6.3	6.4
244	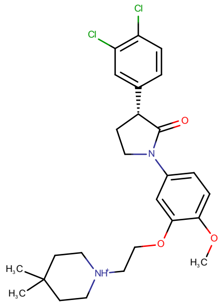	6.4	6.5	6.4

245	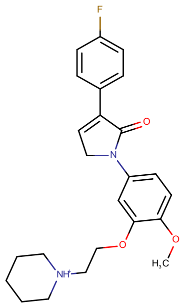	6.4	6.2	6.4
246	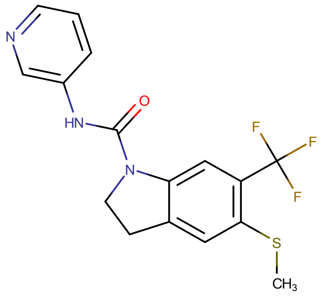	6.4	6.3	6.4
247	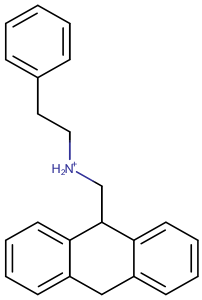	6.4	7.1	7.1
248	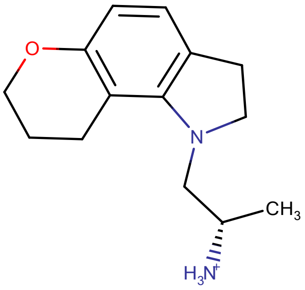	6.4	6.4	6.4
249	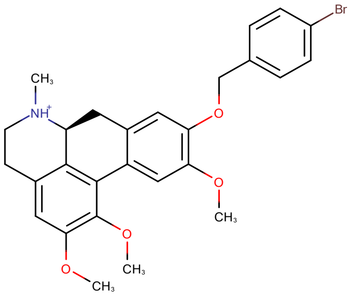	6.4	6.5	6.4

250	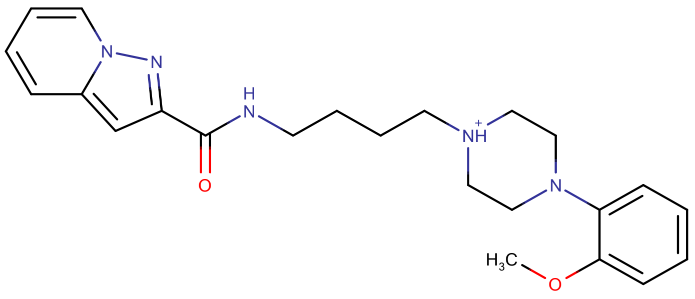	6.4	6.8	6.4
251	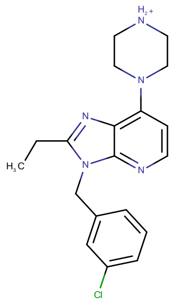	6.4	6.6	6.4
252	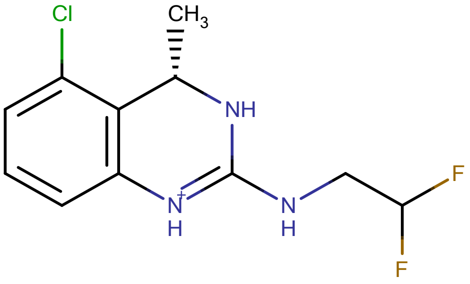	6.3	6.4	6.3
253	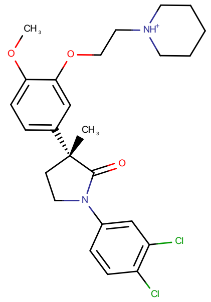	6.3	6.8	6.3
254	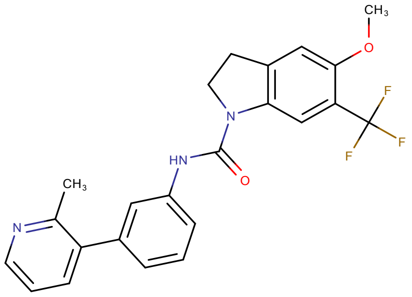	6.3	6.2	6.3

255	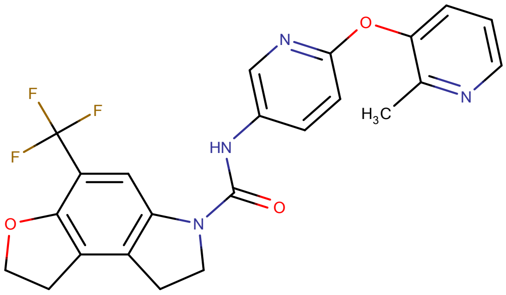	6.3	6.3	6.3
256	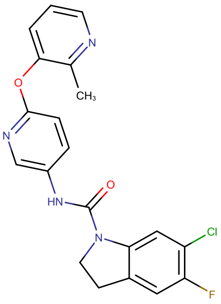	6.3	6	6.3
257	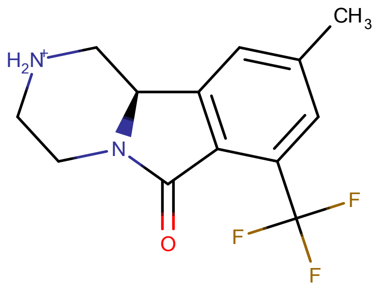	6.3	6.4	6.2
258	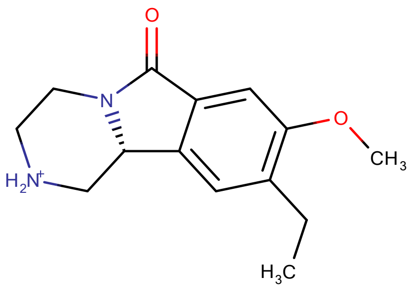	6.2	6.9	6.7
259	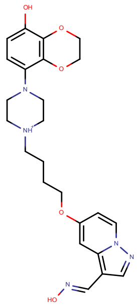	6.2	6.4	6.2

260	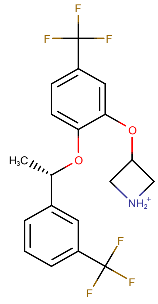	6.2	6.5	6.2
261	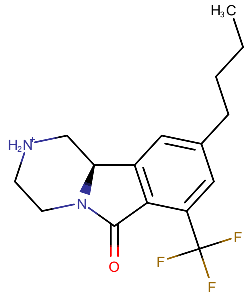	6.2	6.3	6.2
262	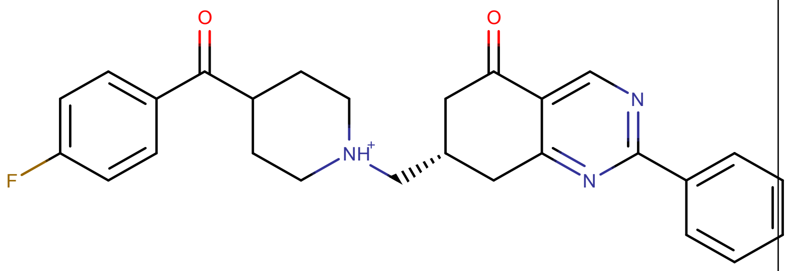	6.2	6.9	6.6
263	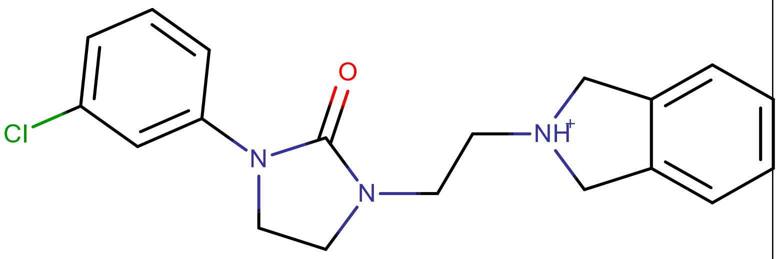	6.2	6.8	6.2
264	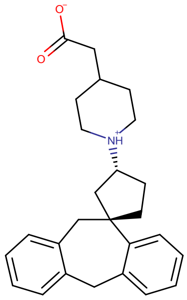	6.2	6.5	6.2

265	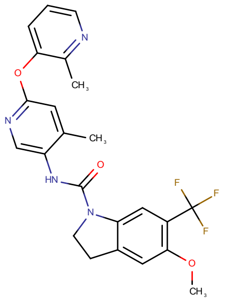	6.2	6.3	6.2
266	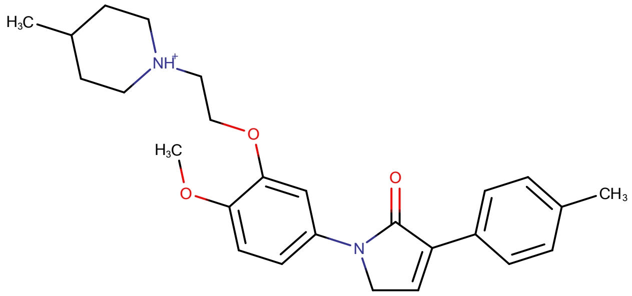	6.2	7	6.2
267	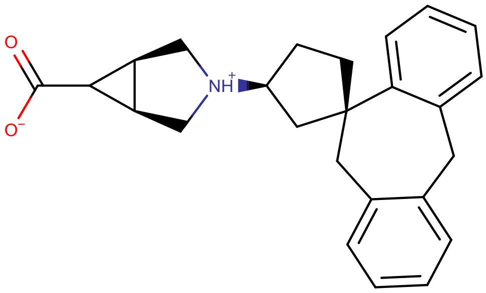	6.2	6.3	6.2
268	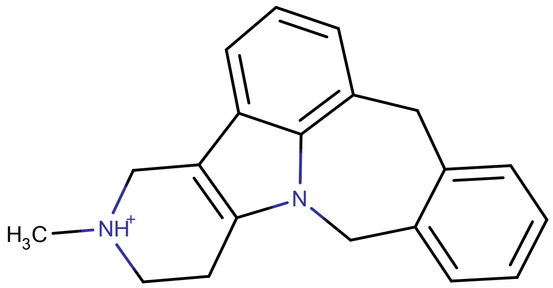	6.1	6.8	6.5
269	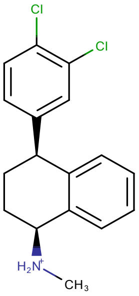	6.1	6.1	6.1

270	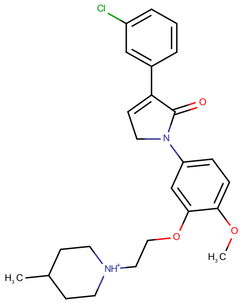	6.1	6.1	6.1
271	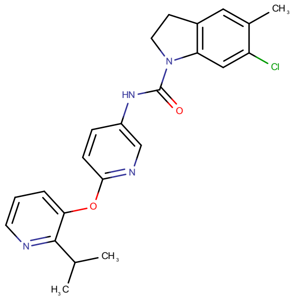	6.1	6.5	6.1
272	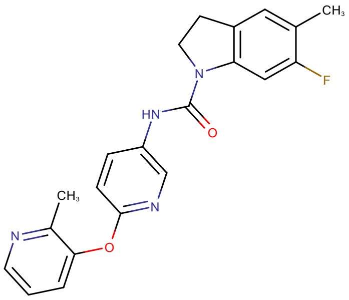	6.1	6.5	6.3
273	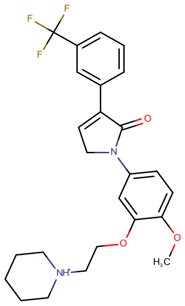	6.1	6.6	6.1
274	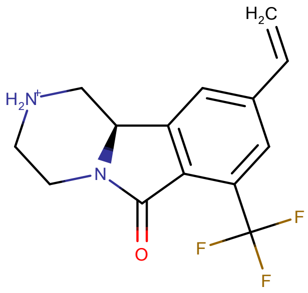	6.1	6.3	6.1

275	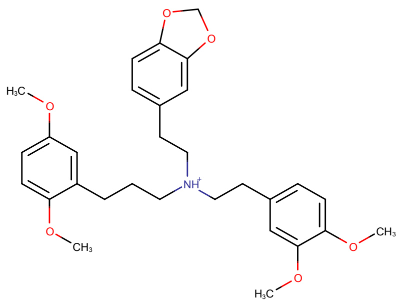	6	6.5	6
276	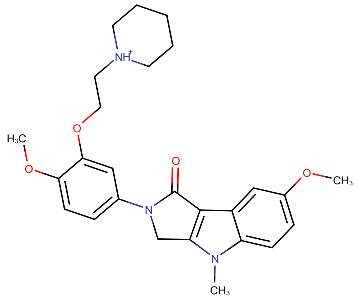	6	6.1	6
277	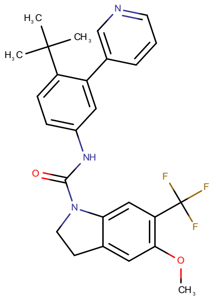	6	5.9	6
278	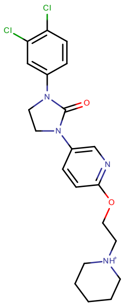	6	6.2	6
279	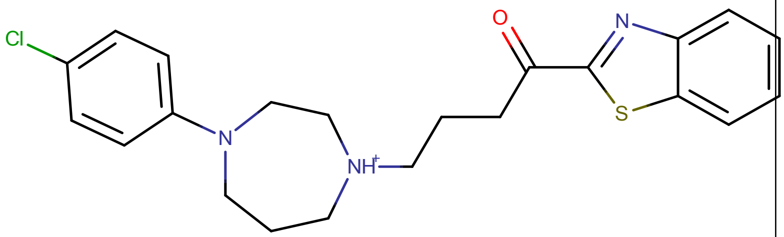	6	6.3	6.4

280	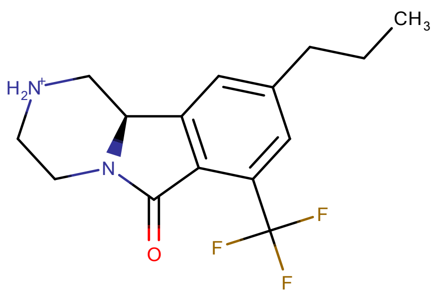	6	6.1	6
281	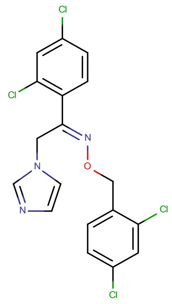	5.9	6.2	5.9
282	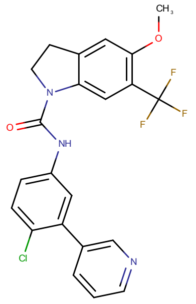	5.9	6.4	6.3
283	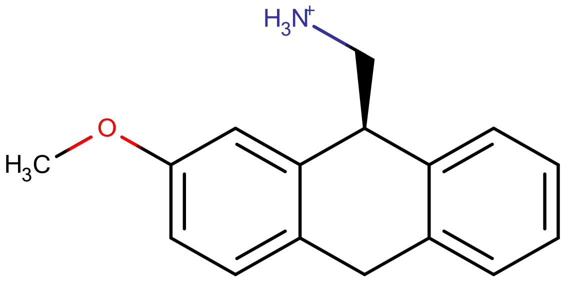	5.9	6.1	5.9
284	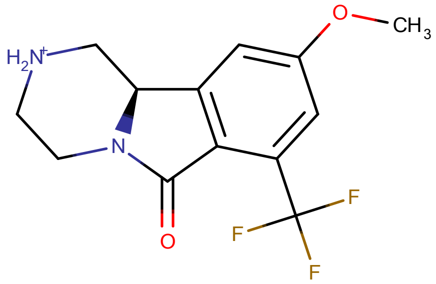	5.9	6.1	5.9

285	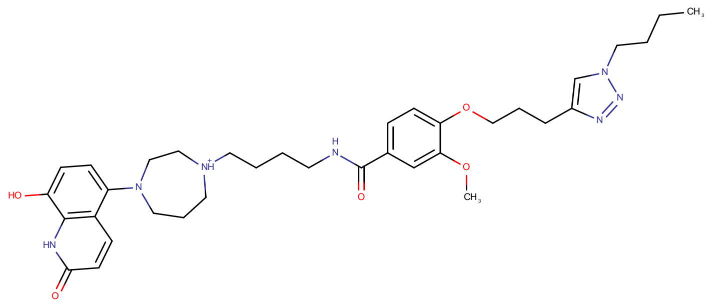	5.8	6	5.8
286	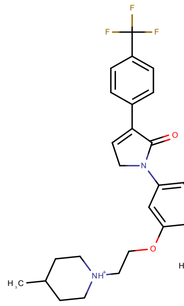	5.8	5.9	5.8
287	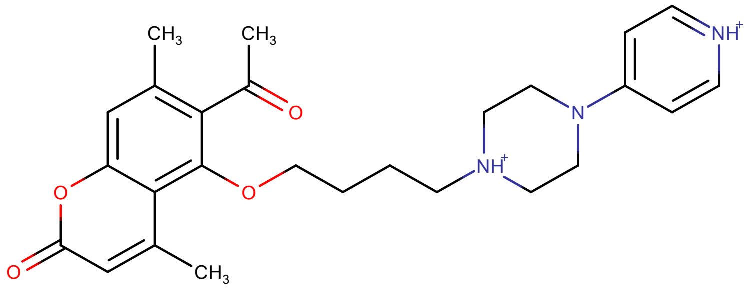	5.8	5.9	5.8
288	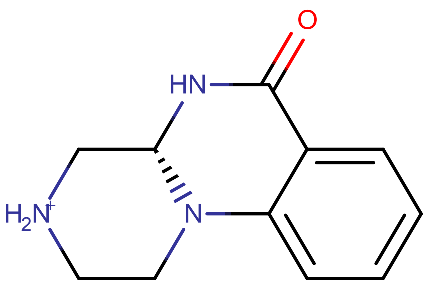	5.7	6.3	6
289	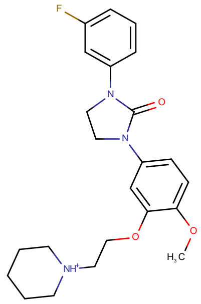	5.7	6.2	5.7

290	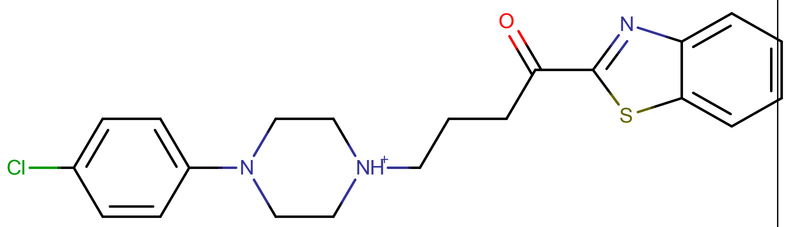	5.7	6.4	5.7
291	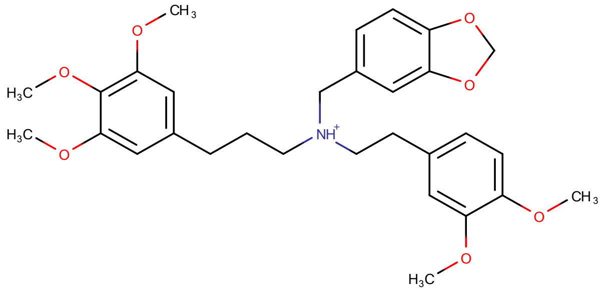	5.7	6.2	5.7
292	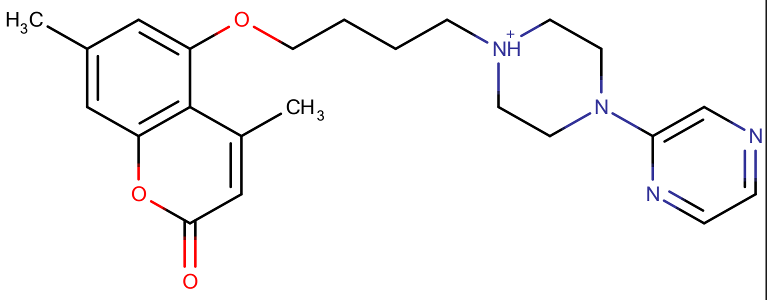	5.6	5.6	5.6
293	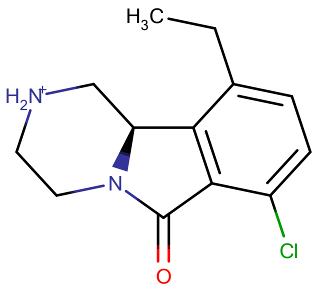	5.6	5.9	5.7
294	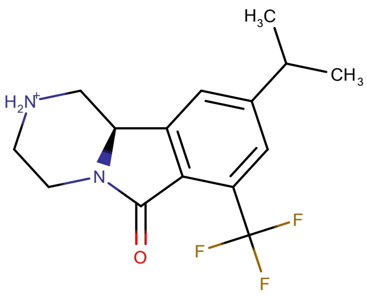	5.6	6.2	5.7

295	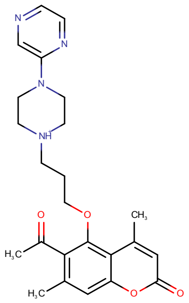	5.5	6	5.5
296	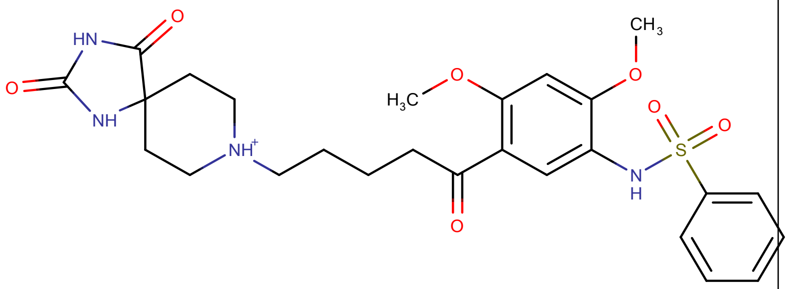	5.5	5.8	5.5
297	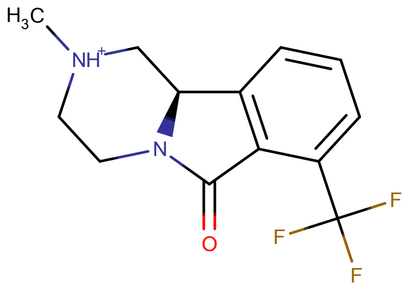	5.4	6.2	6.1
298	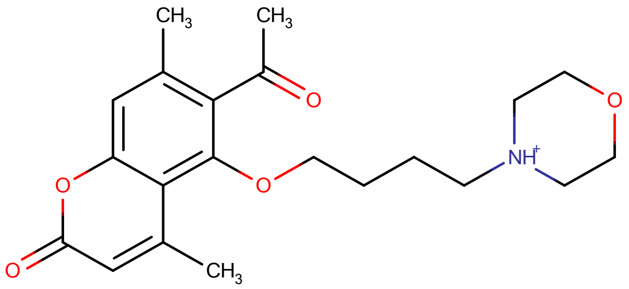	5.4	5	5.3
299	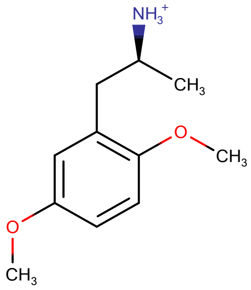	5.3	6	5.4

300	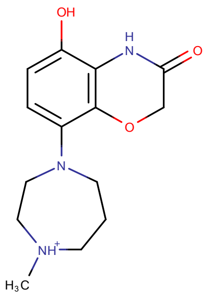	5.2	5.9	5.7

Table S2bis. Structures, experimental and predicted (field and SVM) pKi values of the molecules in the test set

n	Structure	$_{\text{exp}}pK_i$	$_{\text{field}}pK_i$	$_{\text{SVM}}pK_i$
1	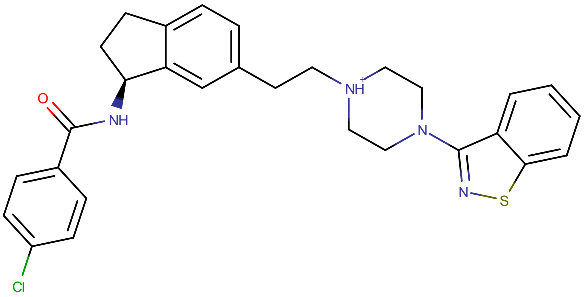	10	9.2	8.8
2	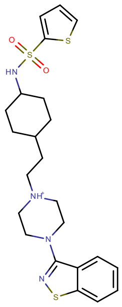	9.5	8.9	8.6

3	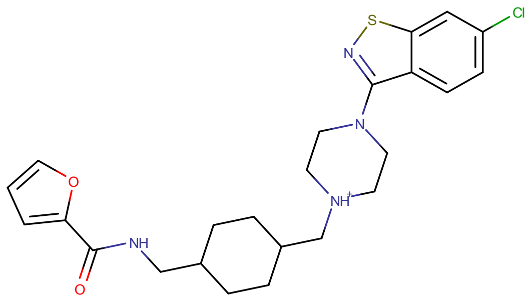	9.5	9.3	9
4	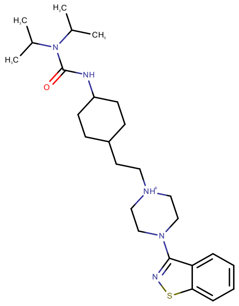	9.4	8.7	8.8
5	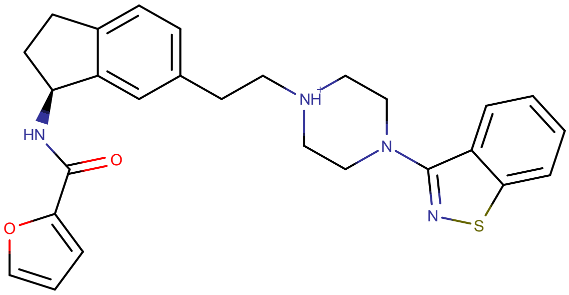	9.3	9.5	9.5
6	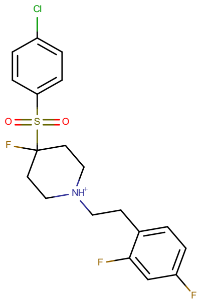	9.2	8.2	8.3
7	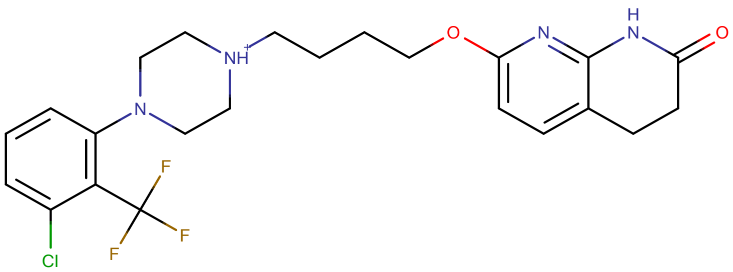	8.9	8.4	8.4

8	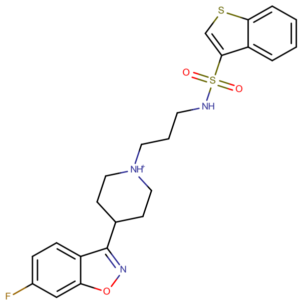	8.8	8.7	8.8
9	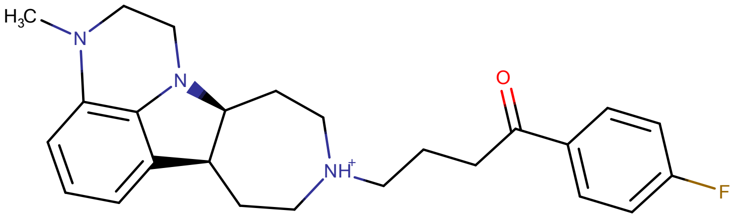	8.8	8.7	8.6
10	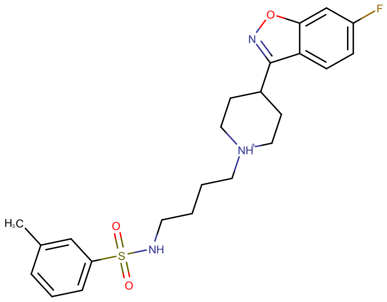	8.7	9.1	9
11	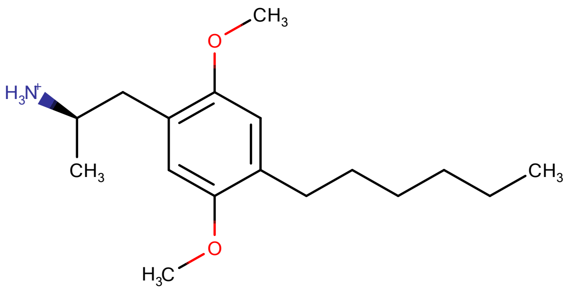	8.6	7.6	7.6

12	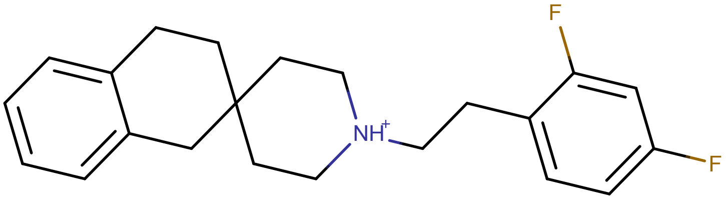	8.5	8.3	8.3
13	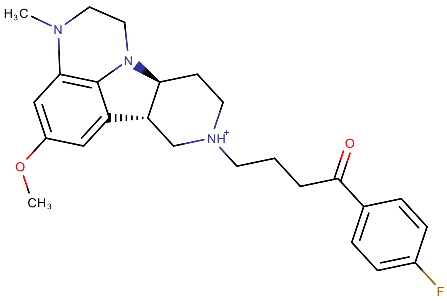	8.5	7.8	8.1
14	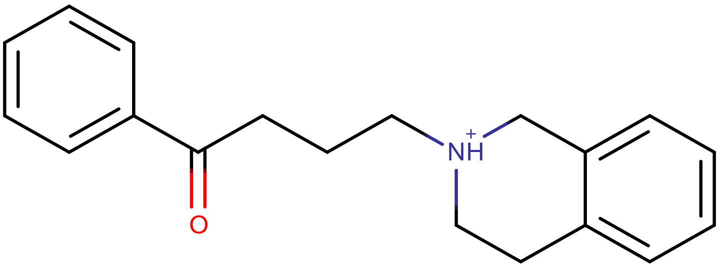	8.4	8	7.9
15	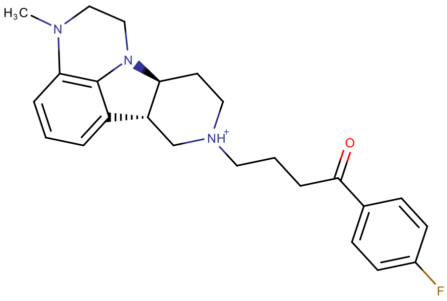	8.3	8.1	8.1
16	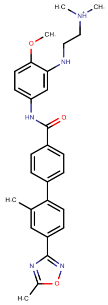	8.3	7.7	8.1

17	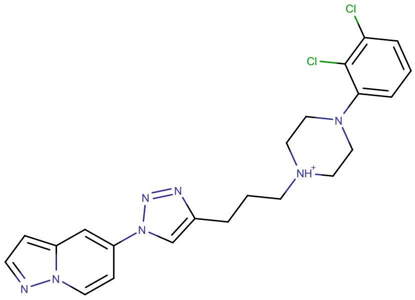	8.3	8.3	8.3
18	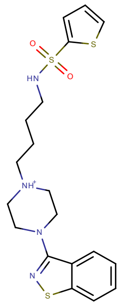	8.3	9	8.2
19	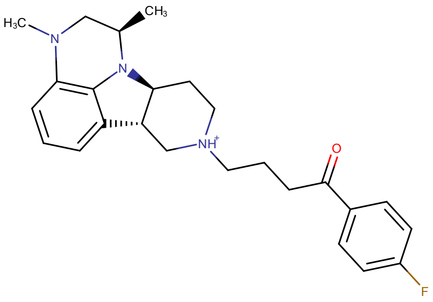	8.2	8.6	8.4
20	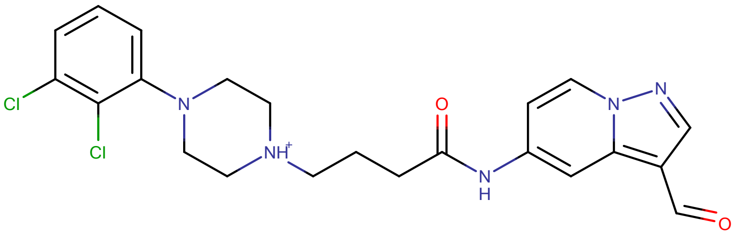	8.2	7.7	7.8

21	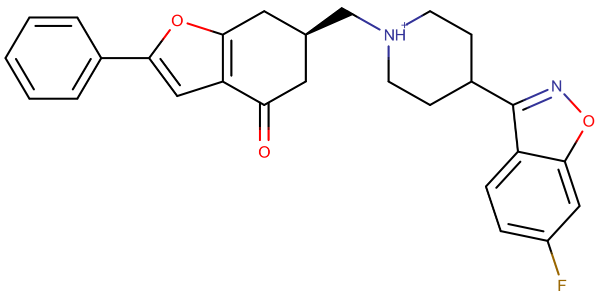	8.2	8.7	8.6
22	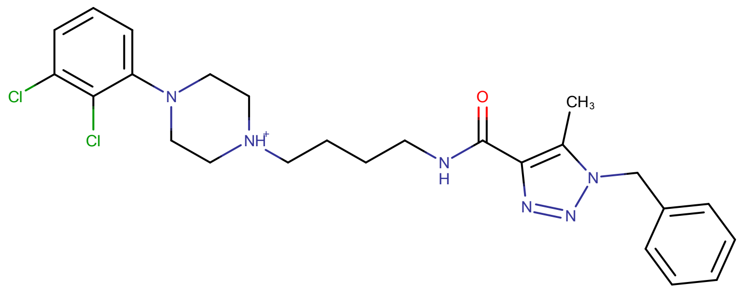	8.1	8.1	8
23	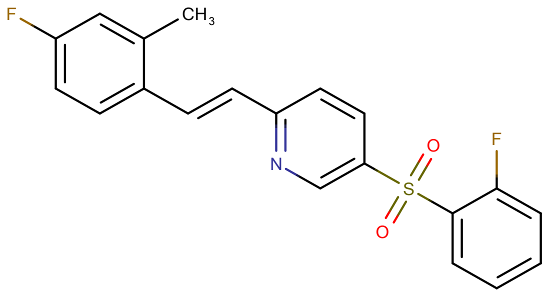	8.1	7.4	7.5
24	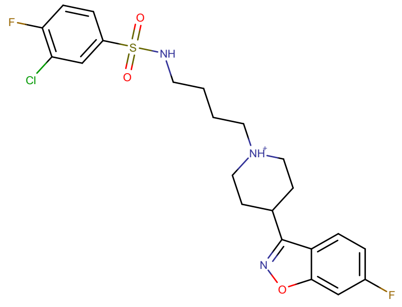	8.1	7	7.1
25	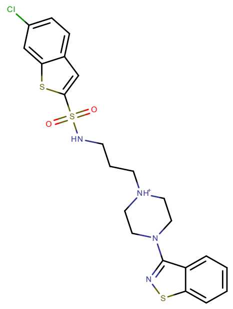	8	8	8.2

26	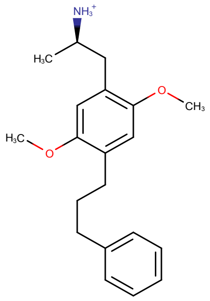	8	7.9	8
27	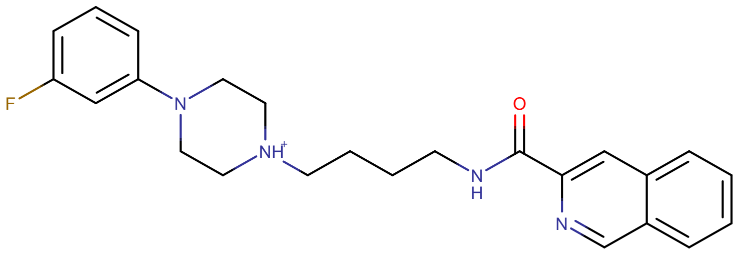	8	7.5	7.8
28	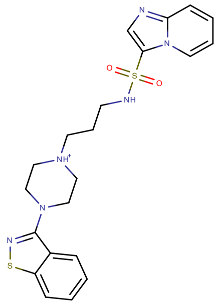	7.9	7.3	7.4
29	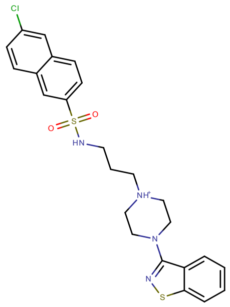	7.9	7.7	7.7

30	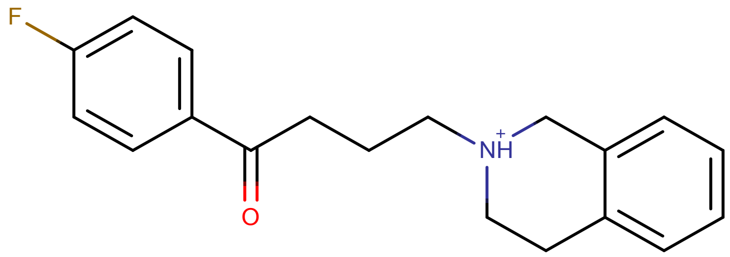	7.9	7.7	7.7
31	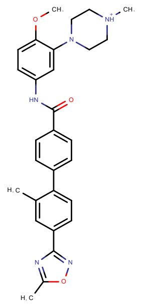	7.8	8	7.7
32	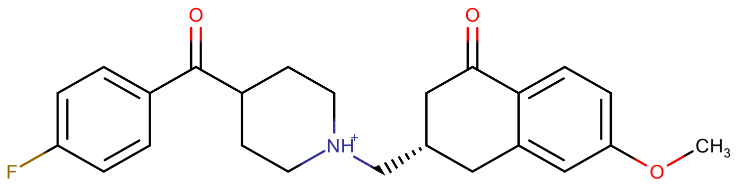	7.8	8.2	8.6
33	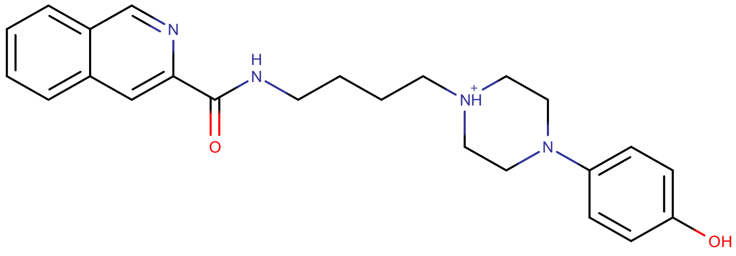	7.8	8.3	8.2
34	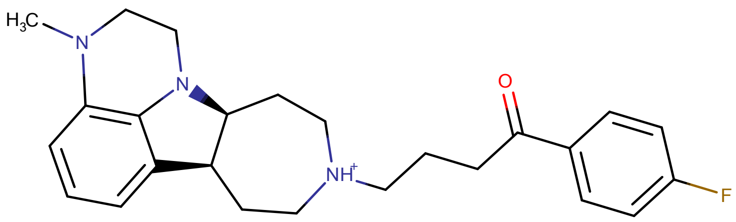	7.8	7.8	7.8

35	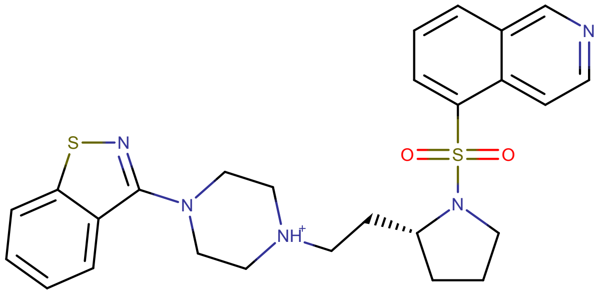	7.7	7.5	7.6
36	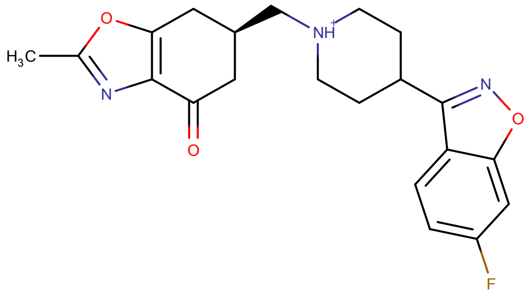	7.7	8.1	7.9
37	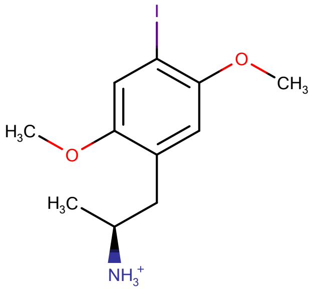	7.6	6.8	6.7
38	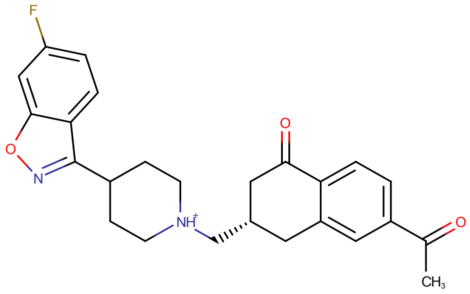	7.5	8	7.6

39	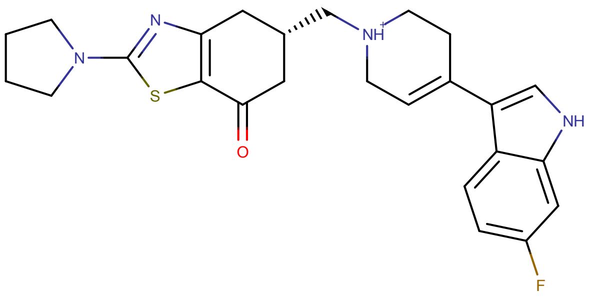	7.4	7.8	7.9
40	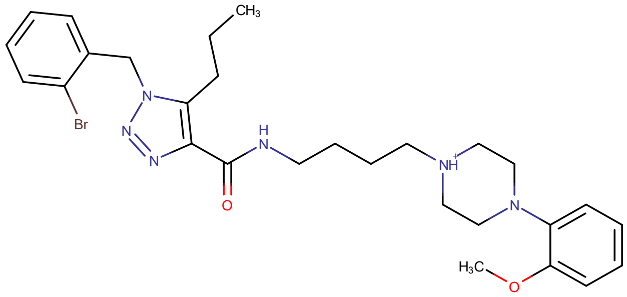	7.4	7.5	7.4
41	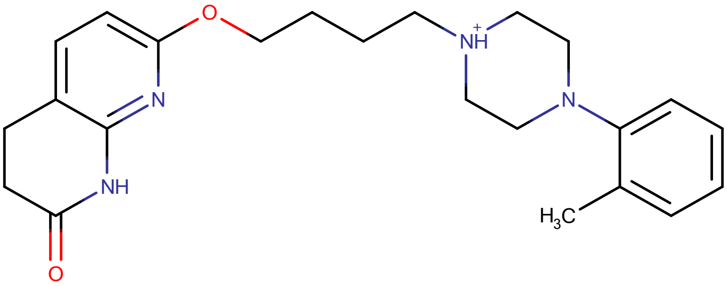	7.3	7.7	7.7
42	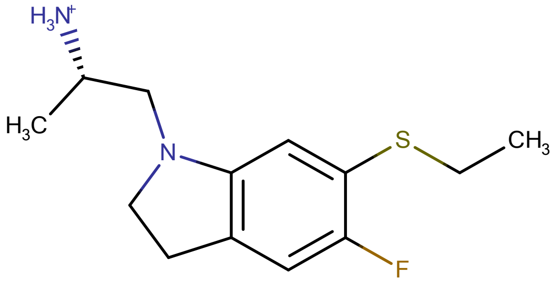	7.2	6.9	7.1
43	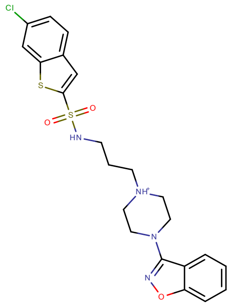	7.2	7.3	7.2

44	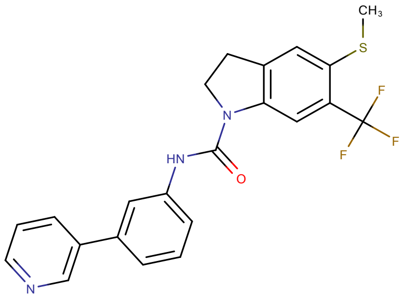	7.1	6.6	7
45	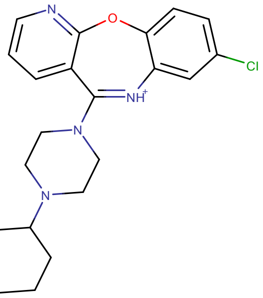	7	6.9	7
46	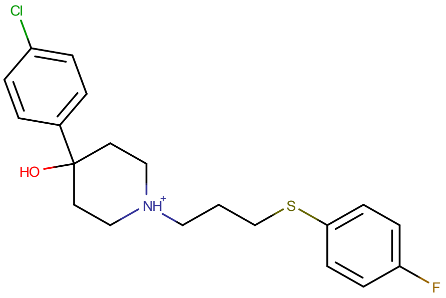	7	8	8
47	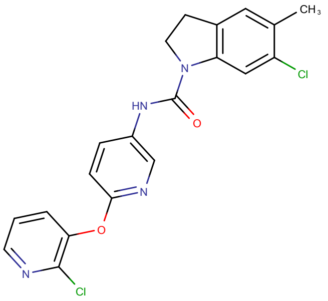	7	6.4	6.5

48	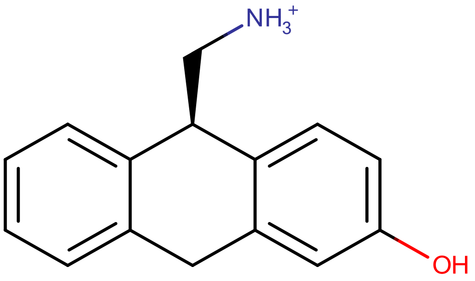	7	7.1	7.1
49	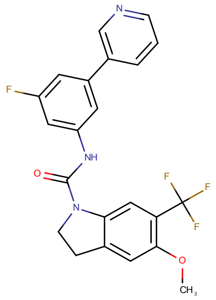	6.9	6.9	7.2
50	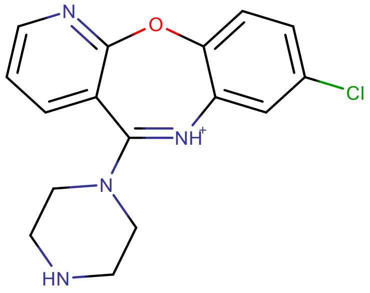	6.9	6.2	6.4
51	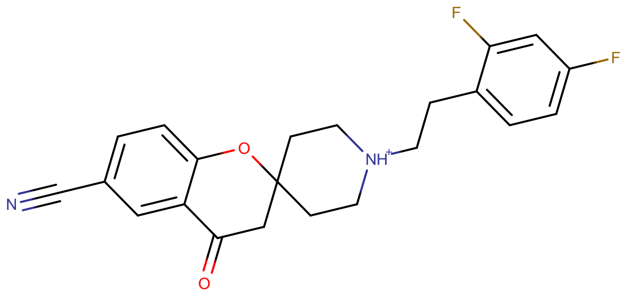	6.8	7.6	7.5
52	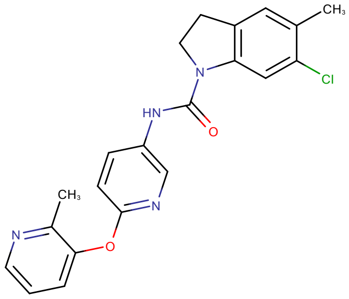	6.8	6.4	6.4

53	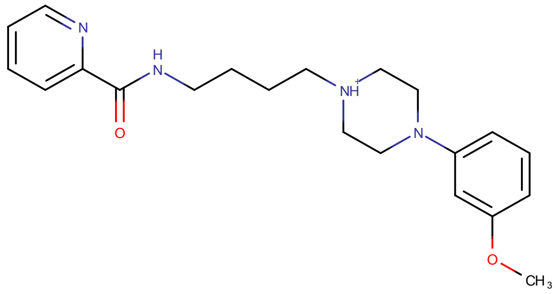	6.8	7.5	7.6
54	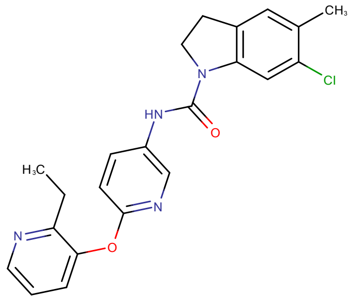	6.7	6.7	6.5
55	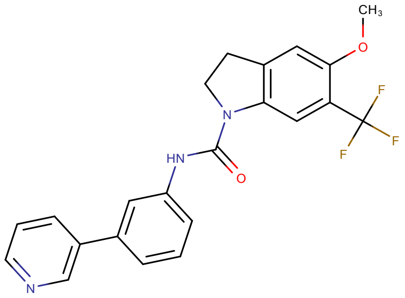	6.7	7	7.2
56	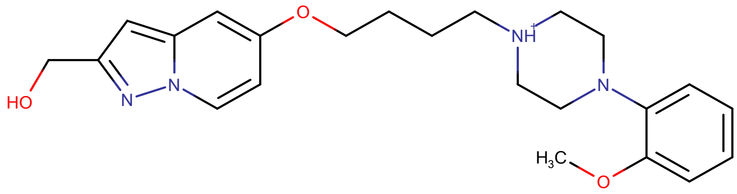	6.6	7.1	7.5

57	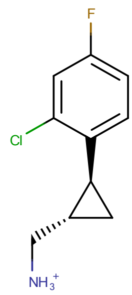	6.6	6.4	6.6
58	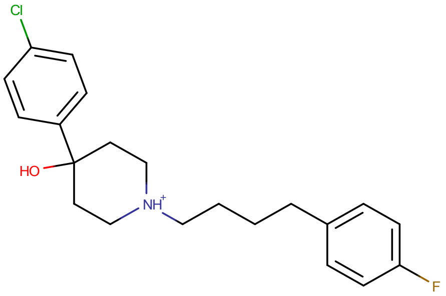	6.5	7.5	7.3
59	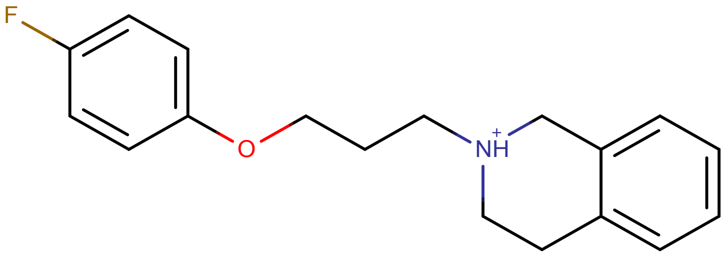	6.5	7.2	7.4
60	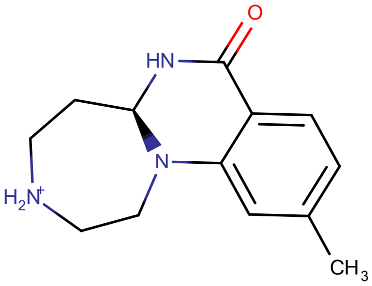	6.4	6.4	6.9
61	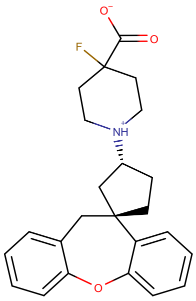	6.4	6.9	7

62	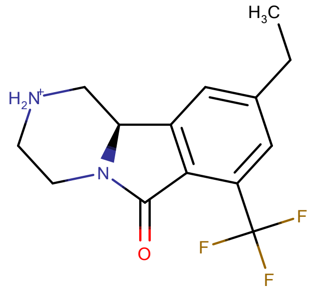	6.4	5.8	5.7
63	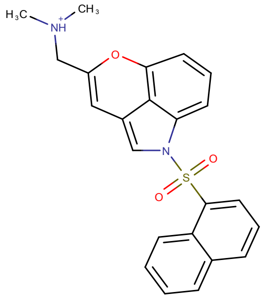	6.3	5.7	5.6
64	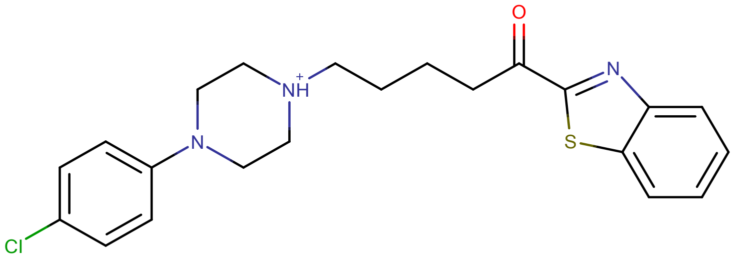	6.3	6.9	6.9
65	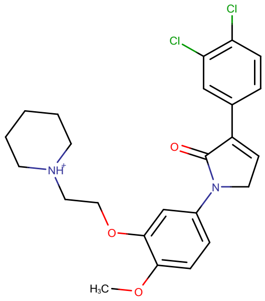	6.2	5.8	5.6

66	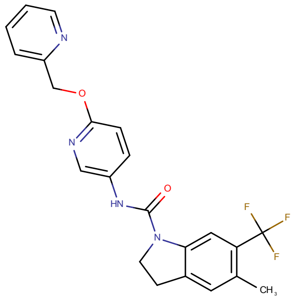	6.2	7.1	7.1
67	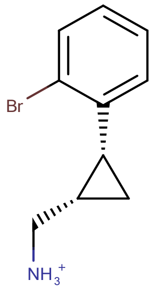	6.2	6.5	6.4
68	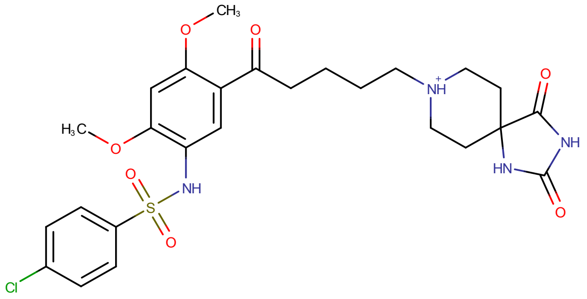	6.1	5.9	6.1
69	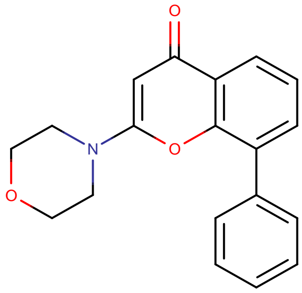	6.1	6.5	7
70	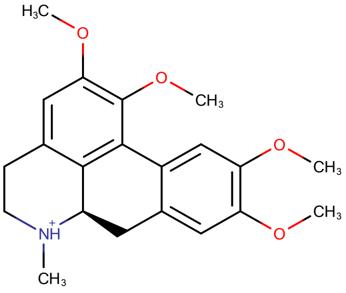	6	7.4	7.1

71	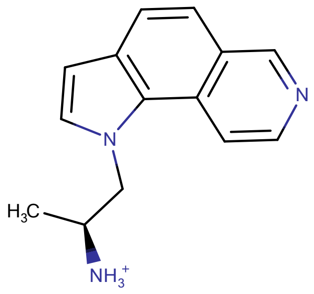	6	6.7	6.7
72	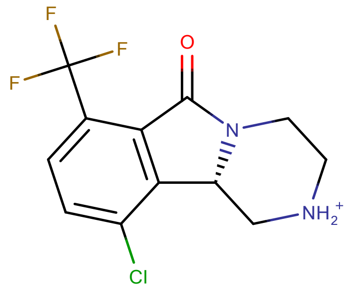	5.8	6.1	6.4
73	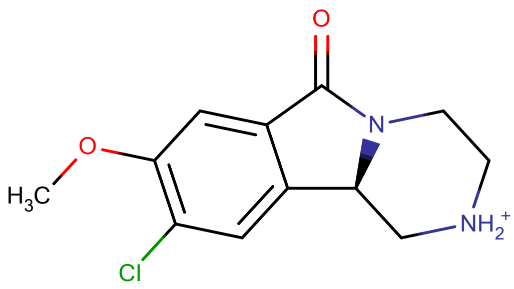	5.8	6.9	7
74	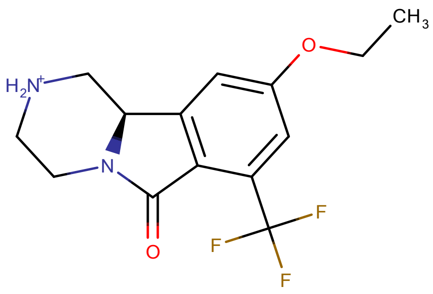	5.4	5.8	5.8

75	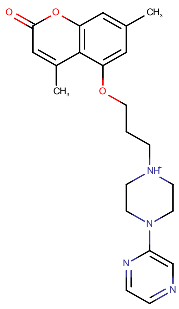	5.3	5.5	5.7

Table S4bis. Calculated ${}_{\text{field}}\text{p}K_{\text{i}}$, ${}_{\text{SVM}}\text{p}K_{\text{i}}$ and ${}_{\text{mean}}\text{p}K_{\text{i}}$ for the natural products screening and structures

n	name	Structure	${}_{\text{field}}\text{p}K_{\text{i}}$	${}_{\text{SVM}}\text{p}K_{\text{i}}$	${}_{\text{mean}}\text{p}K_{\text{i}}$
1	ZINC08856435	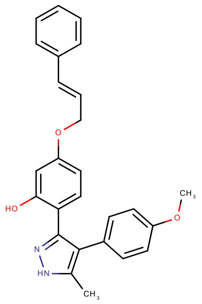	9.1	9.2	9.15
2	ZINC00006368	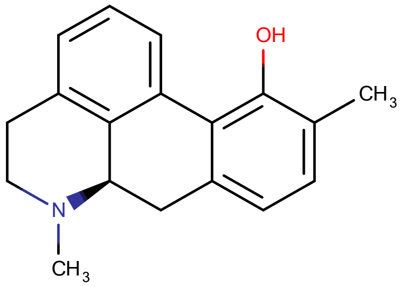	8.9	9	8.95
3	ZINC11867629	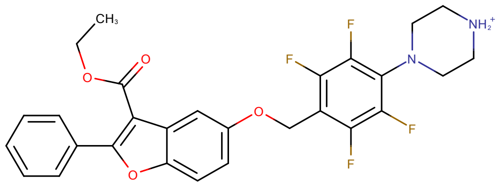	9	8.9	8.95

4	ZINC72116881	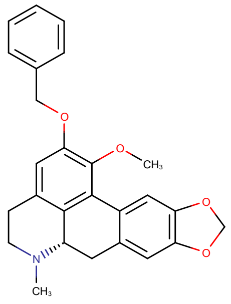	8.7	9	8.9
5	ZINC00607997	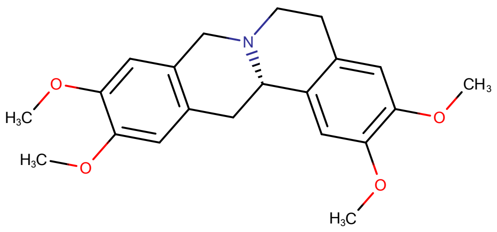	9	8.8	8.9
6	ZINC68576530	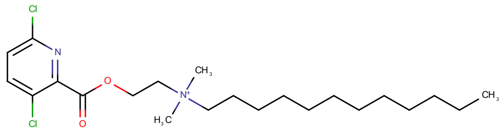	8.7	8.8	8.75
7	ZINC12660071	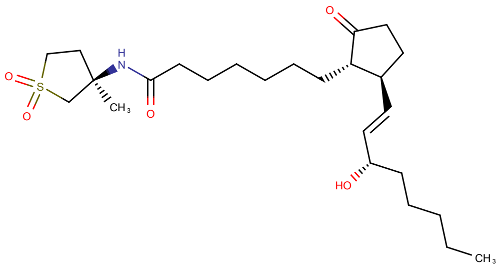	9	8.2	8.6

8	ZINC13690010	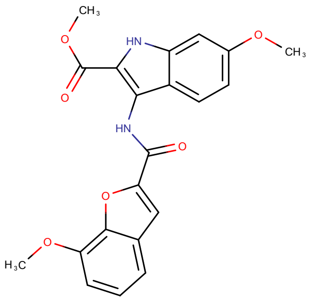	8.9	8.2	8.55
9	ZINC05219478	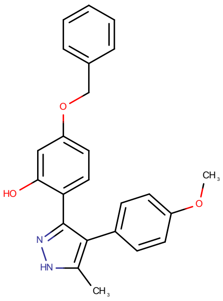	8.7	8.4	8.55
10	ZINC09187792	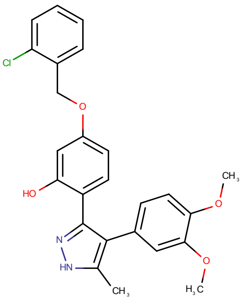	8.6	8.5	8.55
11	12190	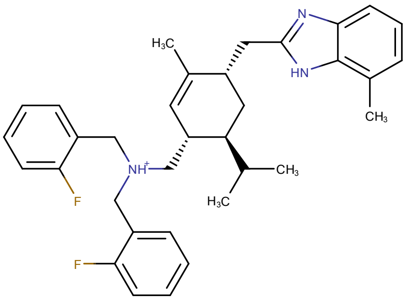	8.9	8.1	8.5
12	ZINC09410825	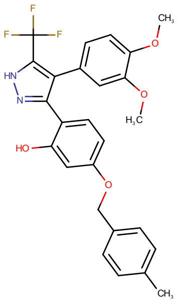	8.5	8.5	8.5

13	MolPort-006-334-884	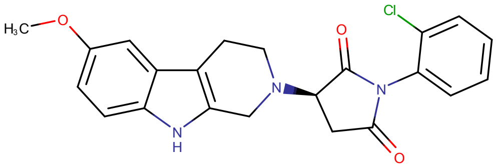	8.5	8.5	8.5
14	ZINC39354033	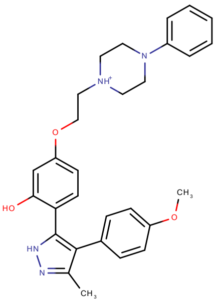	8.5	8.4	8.45
15	ZINC20591088	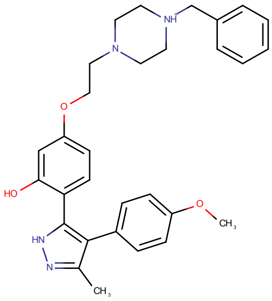	8.5	8.4	8.45
16	ZINC09130703	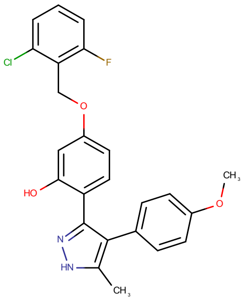	8.4	8.5	8.45
17	ZINC05343552	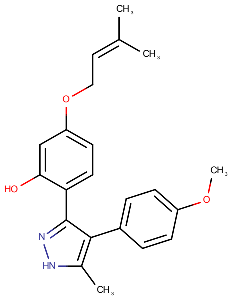	8.4	8.4	8.4

18	ZINC16036365	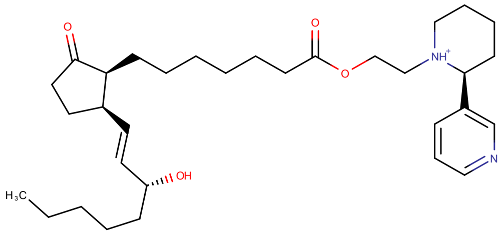	8.3	8.5	8.4
19	ZINC09187797	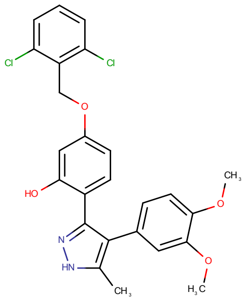	8.4	8.3	8.35
20	157207-88-0	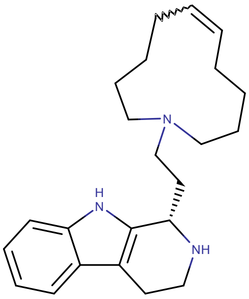	8.4	8.3	8.35
21	ZINC09418544	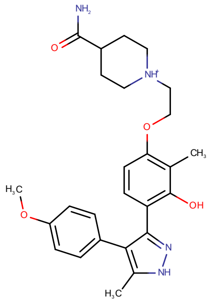	8.3	8.4	8.35
22	ZINC09130113	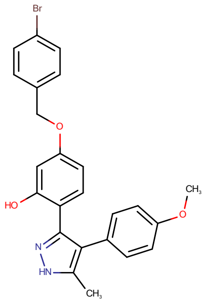	8.3	8.4	8.35

23	MolPort-002-530-410	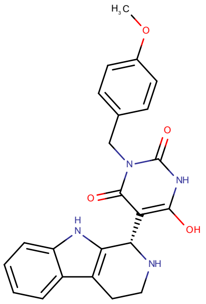	8.5	8.2	8.35
24	SN00009552	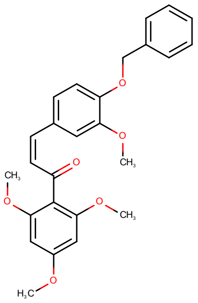	8.5	8.2	8.35
25	ZINC09130734	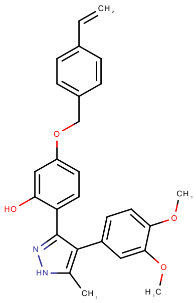	8.4	8.2	8.3
26	ZINC09409677	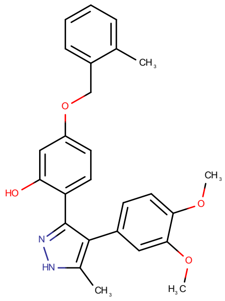	8.4	8.2	8.3
27	ZINC08791003	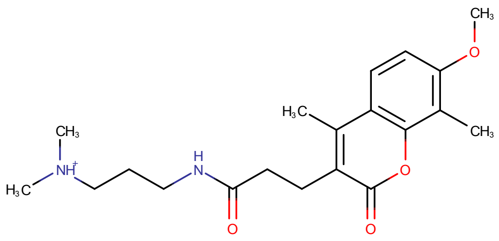	8.4	8.2	8.3

28	ZINC09130659	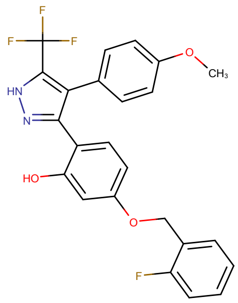	8.4	8.1	8.25
29	6932	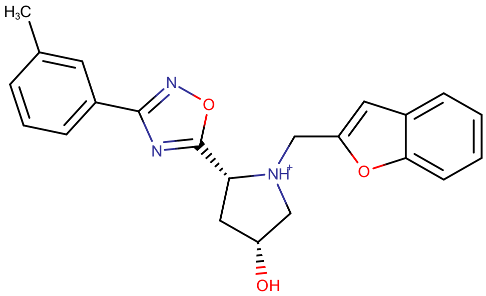	8.3	8.2	8.25
30	ZINC08972988	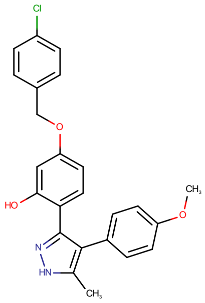	8.3	8.2	8.25
31	SN00149601	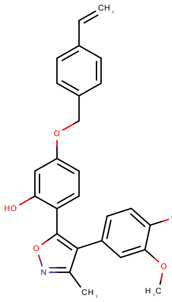	8.3	8.2	8.25
32	SN00061787	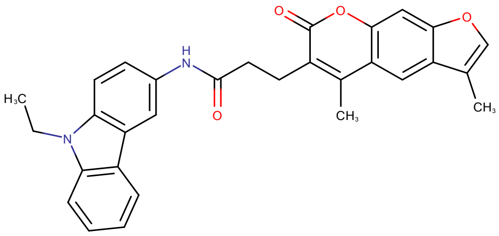	8.3	8.1	8.2

33	ZINC09129618	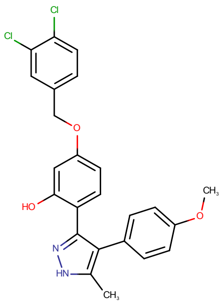	8.3	8.1	8.2
34	ZINC08973292	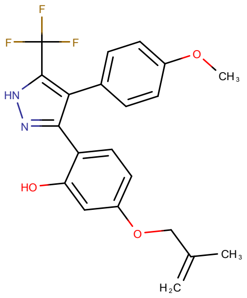	8.2	8.2	8.2
35	ZINC09187780	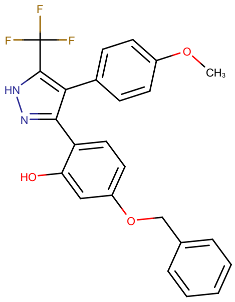	8.2	8.2	8.2
36	ZINC68572101	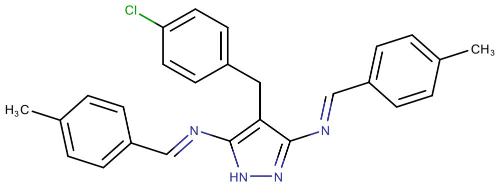	8.2	8.2	8.2
37	MolPort-001-016-686	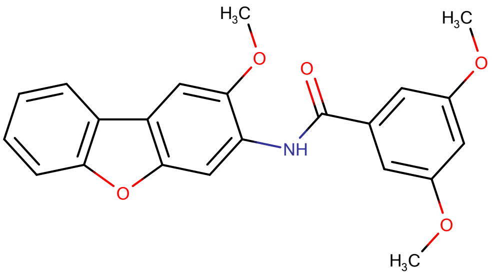	8.1	8.3	8.2

38	ZINC08973452	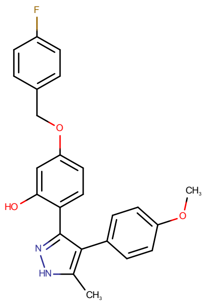	8.1	8.3	8.2
39	MolPort-002-535-647	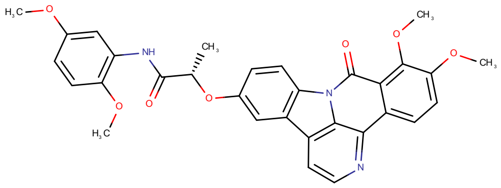	8.3	8	8.15
40	ZINC09410789	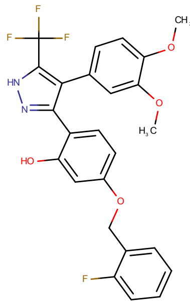	8.1	8.2	8.15
41	MolPort-027-852-775	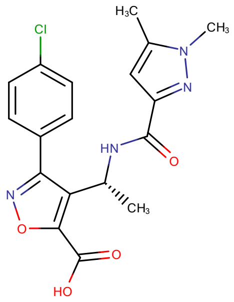	8.1	8.2	8.15
42	ZINC09409653	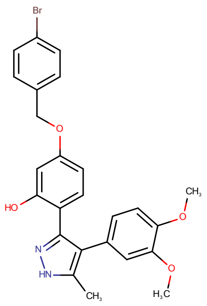	8.1	8.2	8.15

43	MolPort-001-738-042	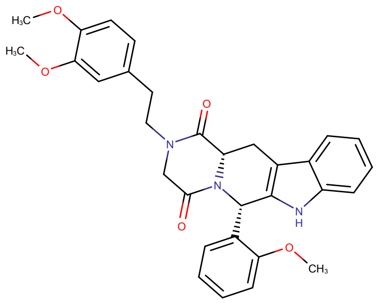	8.1	8.2	8.15
44	SN00148291	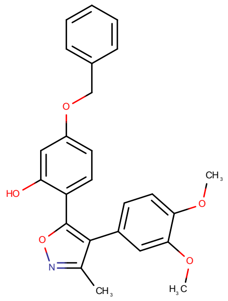	7.9	8.3	8.1
45	ZINC20466733	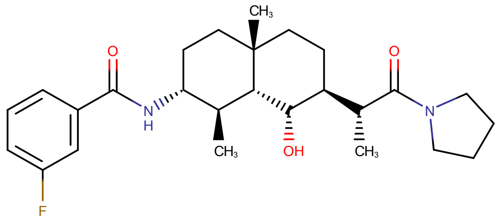	8.1	8.1	8.1
46	SN00147982	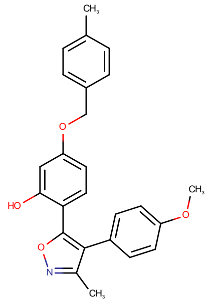	8	8.2	8.1
47	ZINC04235438	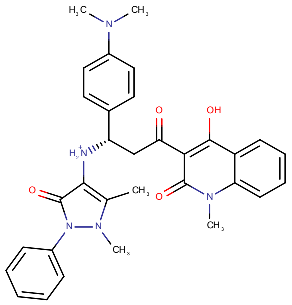	8.2	7.9	8.05

48	ZINC02098147	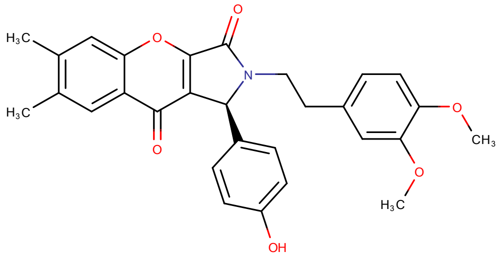	8	8.1	8.05
49	ZINC20411284	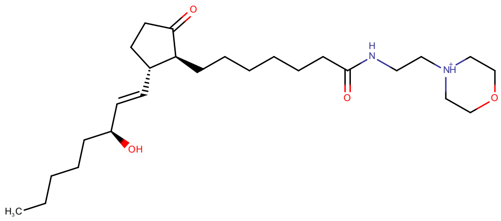	8	8.1	8.05
50	ZINC09410809	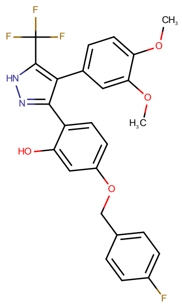	8.1	7.9	8
51	ZINC09421984	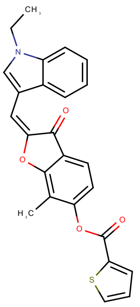	8.1	7.9	8
52	ZINC70672992	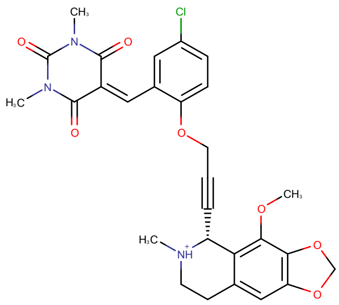	8	8	8

53	ZINC11866356	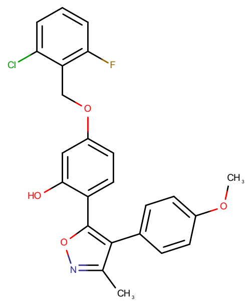	8	8	8
54	ZINC01780045	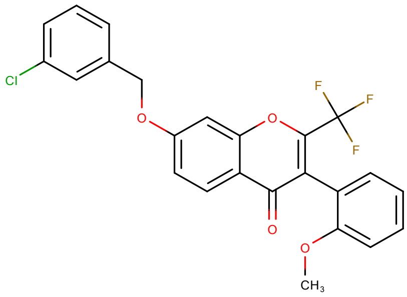	7.9	8.1	8
55	ZINC11866343	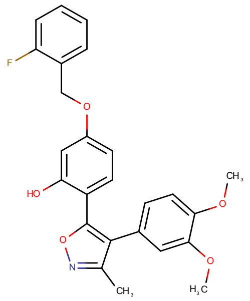	7.8	8.2	8
56	ZINC09186350	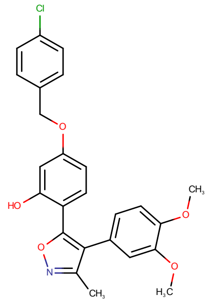	7.8	8.2	8
57	MolPort-002-511-872	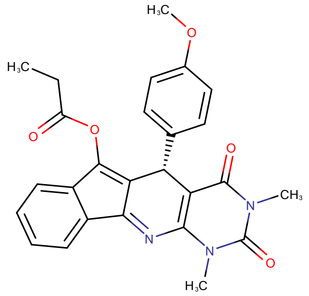	8	7.9	7.95

58	ZINC02122370	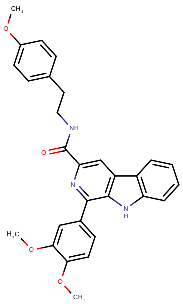	8	7.9	7.95
59	ZINC09409699	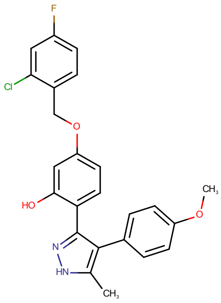	8	7.9	7.95
60	ZINC02429597	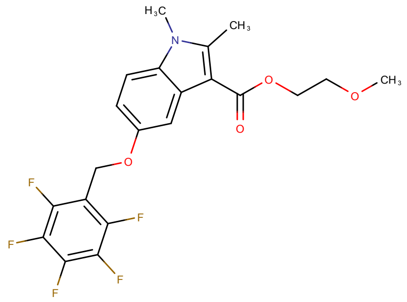	7.9	8	7.95
61	MolPort-000-424-288	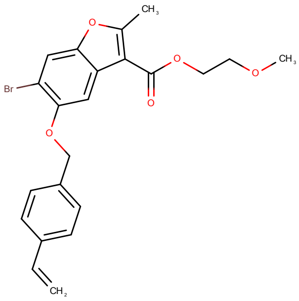	7.9	8	7.95
62	ZINC11866321	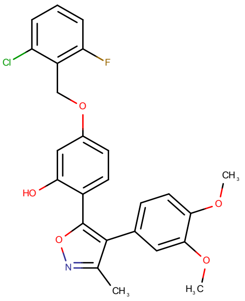	7.9	8	7.95

63	ZINC08972974	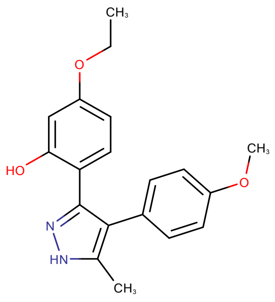	7.9	8	7.95
64	ZINC12898513	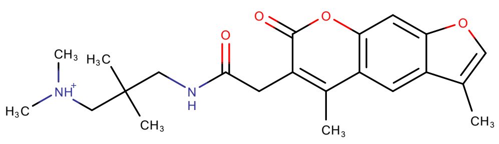	8.1	7.8	7.95
65	147293-04-7	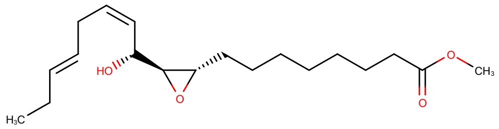	8.1	7.8	7.95
66	SN00031093	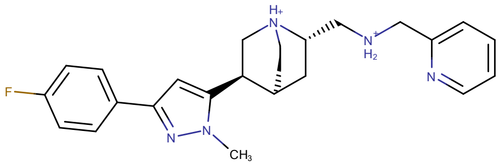	8.1	7.8	7.95
67	ZINC02149559	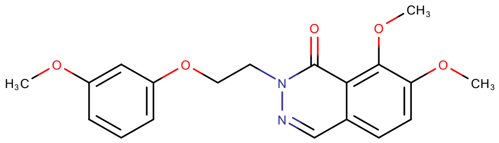	7.8	8.1	7.95

68	ZINC03840589	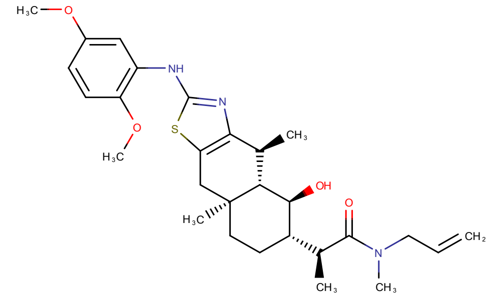	7.8	8.1	7.95
69	MolPort-002-518-881	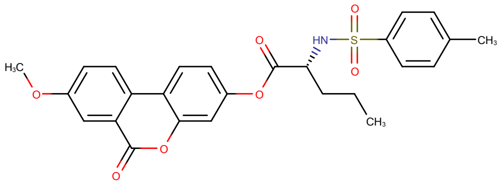	7.7	8.2	7.95
70	ZINC05190540	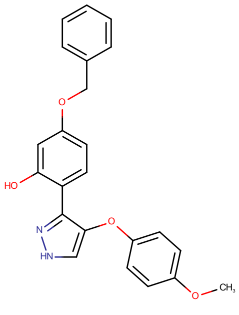	8.1	7.7	7.9
71	MolPort-009-649-103	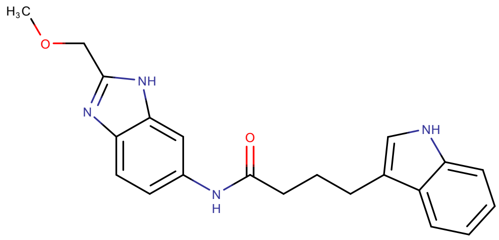	8	7.8	7.9
72	ZINC02319891	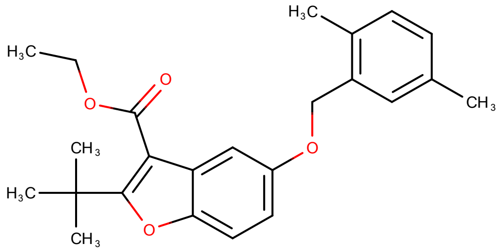	8	7.8	7.9

73	ZINC05572691	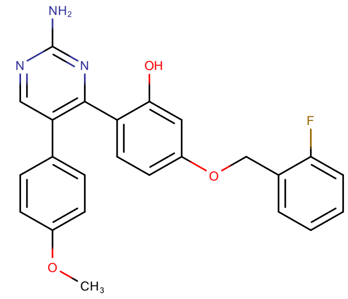	7.9	7.9	7.9
74	ZINC08845645	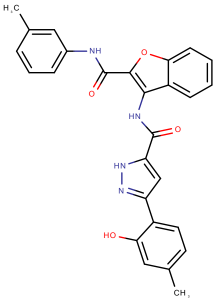	7.6	8.2	7.9
75	ZINC02412441	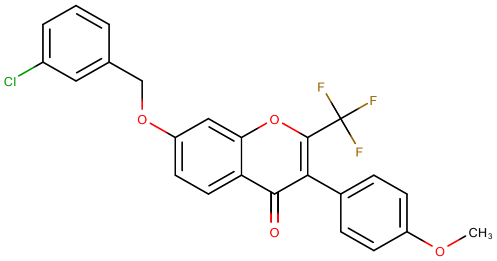	8	7.7	7.85
76	ZINC09129491	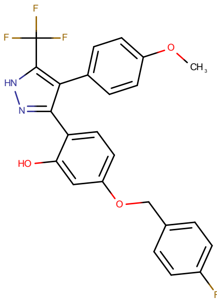	7.9	7.8	7.85
77	ZINC01322966	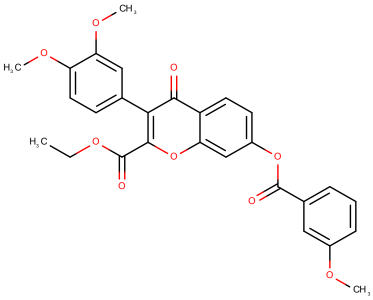	7.9	7.8	7.85

78	ZINC09409930	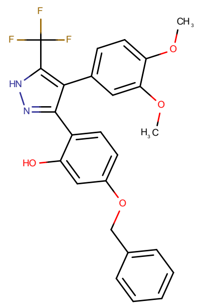	7.9	7.8	7.85
79	ZINC08791787	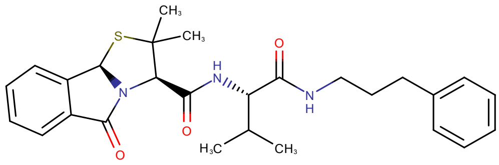	7.9	7.8	7.85
80	ZINC48057077	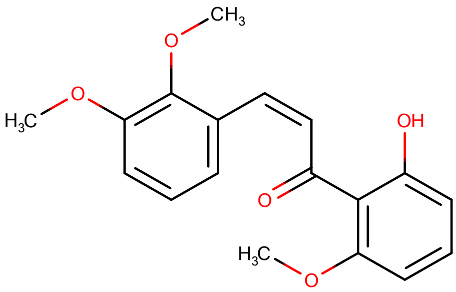	7.8	7.9	7.85
81	ZINC04267171	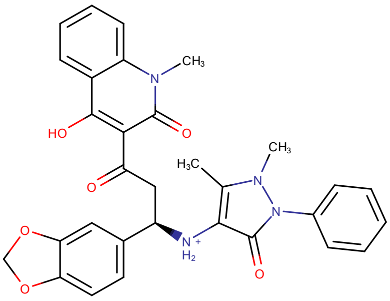	7.8	7.9	7.85
82	ZINC20466528	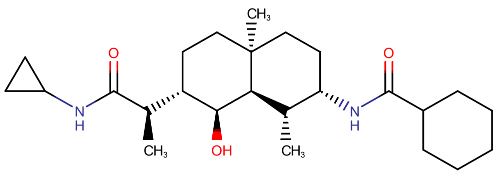	7.8	7.9	7.85

83	ZINC01792699	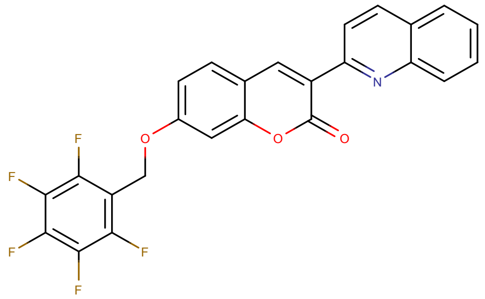	7.8	7.9	7.85
84	ZINC11866287	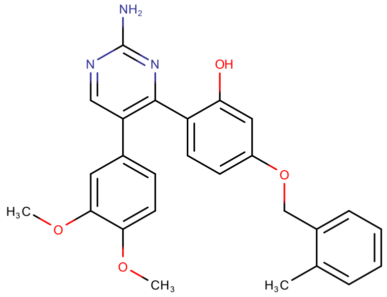	7.8	7.9	7.85
85	ZINC08970919	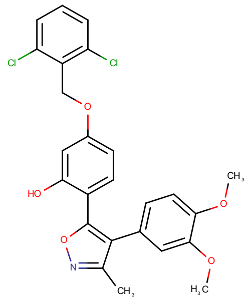	7.7	8	7.85
86	SN00149108	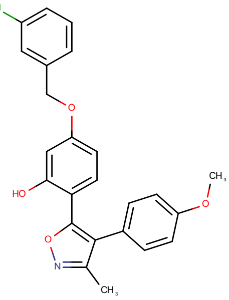	7.7	8	7.85
87	ZINC47284531	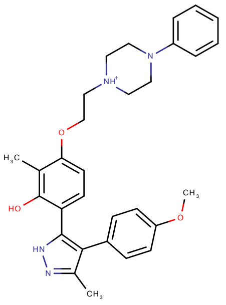	7.6	8.1	7.85

88	ZINC08996172	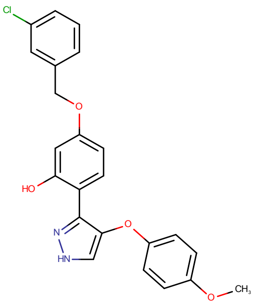	7.7	7.9	7.8
89	ZINC03983996	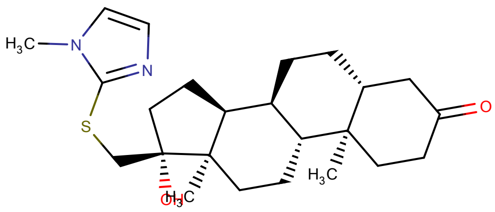	7.7	7.9	7.8
90	ZINC12900696	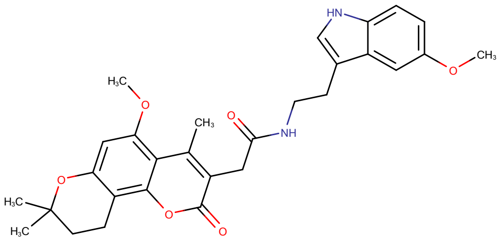	8.1	7.5	7.8
91	ZINC04267149	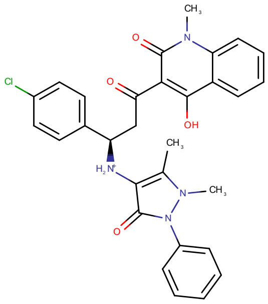	8	7.6	7.8
92	MolPort-035-700-739	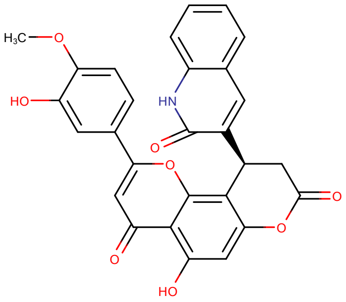	7.8	7.8	7.8

93	ZINC20759596	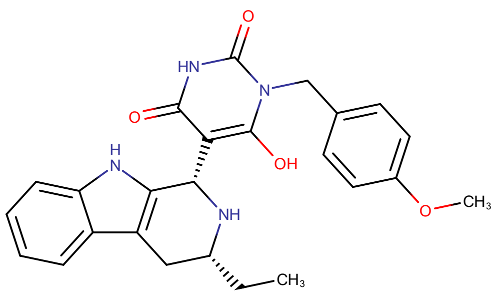	7.8	7.8	7.8
94	ZINC11866347	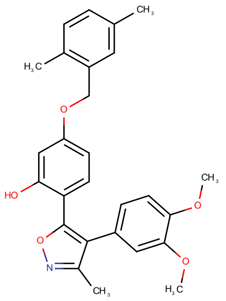	7.8	7.8	7.8
95	ZINC13571467	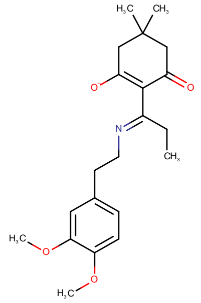	7.8	7.8	7.8
96	ZINC09575415	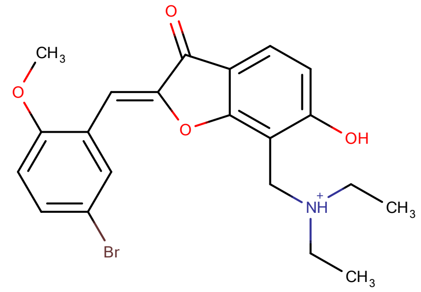	7.8	7.8	7.8
97	ZINC09410298	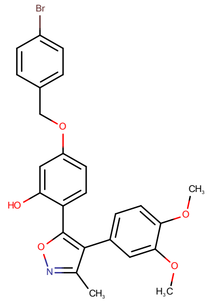	7.6	8	7.8

98	ZINC02158608	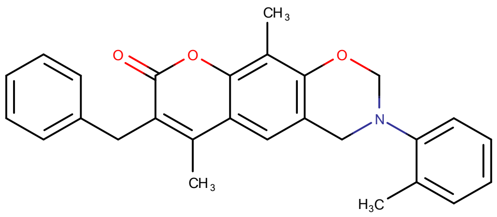	7.6	8	7.8
99	ZINC20760972	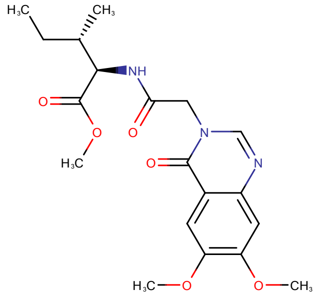	7.5	8.1	7.8
100	SN00149302	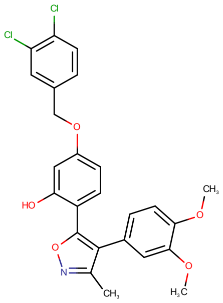	7.4	8.2	7.8
101	ZINC02107196	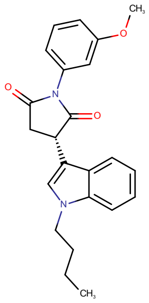	7.9	7.6	7.75
102	MolPort-002-517-334	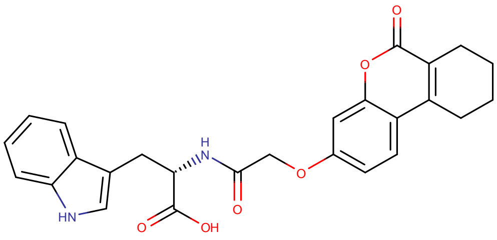	7.9	7.6	7.75

103	ZINC09130740	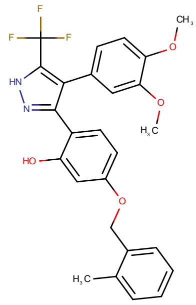	7.8	7.7	7.75
104	MolPort-027-852-794	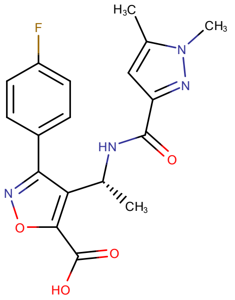	7.8	7.7	7.75
105	ZINC19893649	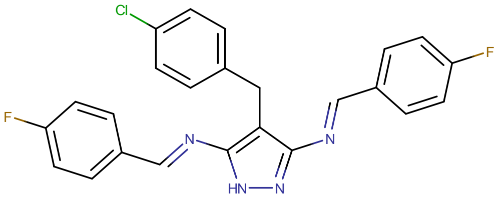	7.8	7.7	7.75
106	ZINC01280062	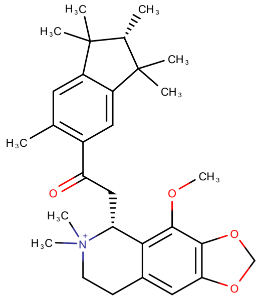	7.7	7.8	7.75
107	ZINC09130747	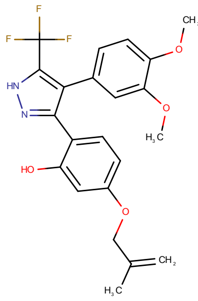	7.7	7.8	7.75

108	MolPort-046-594-356	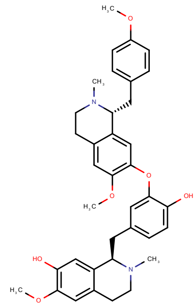	7.7	7.8	7.75
109	ZINC02667596	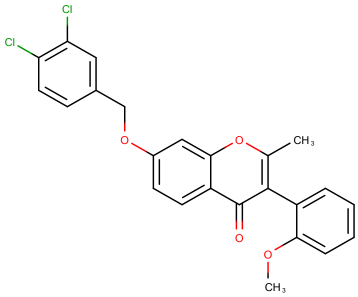	7.7	7.8	7.75
110	MolPort-000-826-974	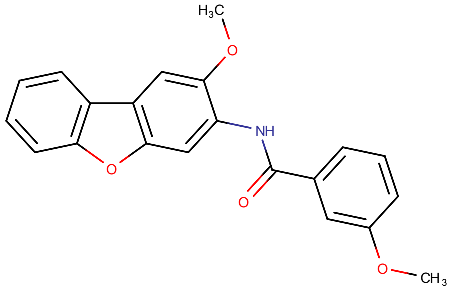	7.6	7.9	7.75
111	ZINC02114178	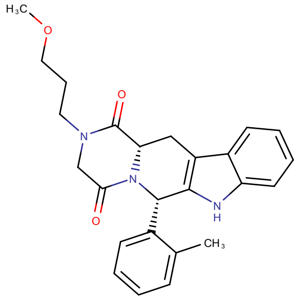	7.6	7.9	7.75
112	SN00382560	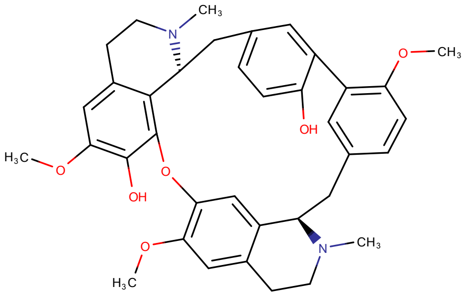	7.6	7.9	7.75

113	SN00151289	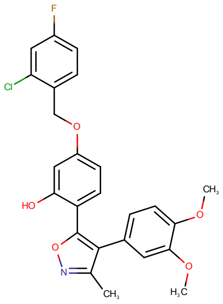	7.6	7.9	7.75
114	ZINC02147505	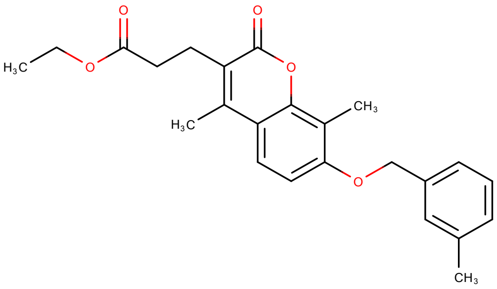	7.5	8	7.75
115	SN00147957	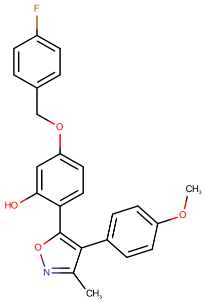	7.5	8	7.75
116	ZINC08792424	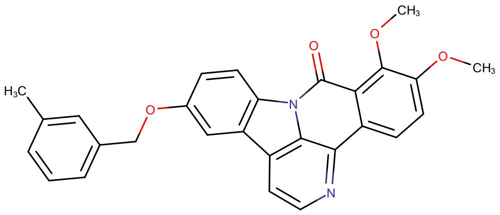	7.7	7.7	7.7
117	ZINC08789180	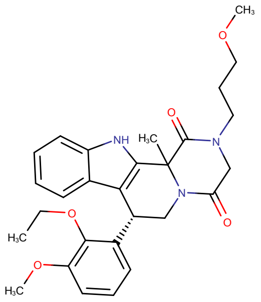	7.7	7.7	7.7

118	ZINC70669913	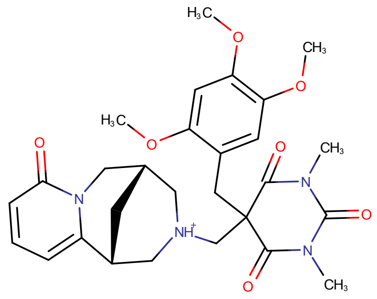	7.7	7.7	7.7
119	ZINC20611836	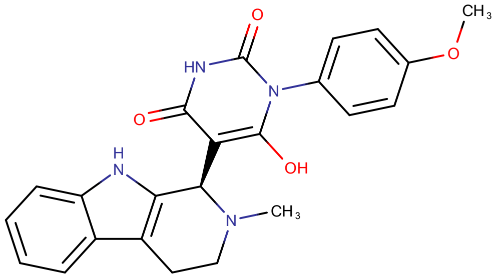	7.5	7.9	7.7
120	ZINC05452205	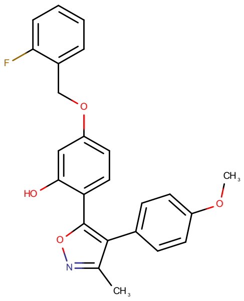	7.5	7.9	7.7
121	ZINC02155319	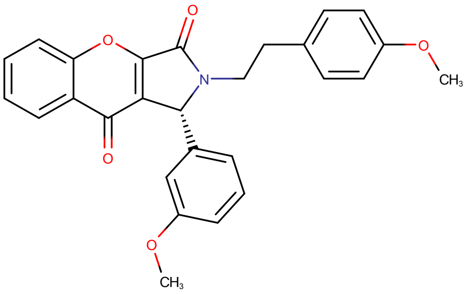	7.8	7.6	7.7
122	MolPort-002-528-757	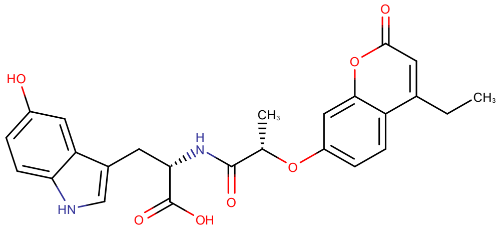	7.8	7.6	7.7

123	MolPort-001-737-511	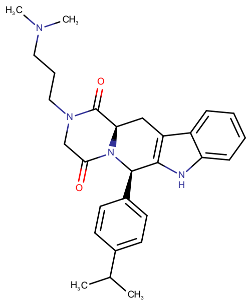	7.8	7.6	7.7
124	20164	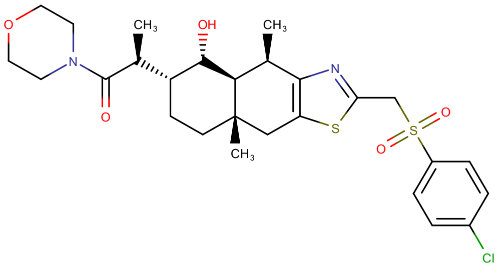	7.6	7.8	7.7
125	MolPort-000-812-834	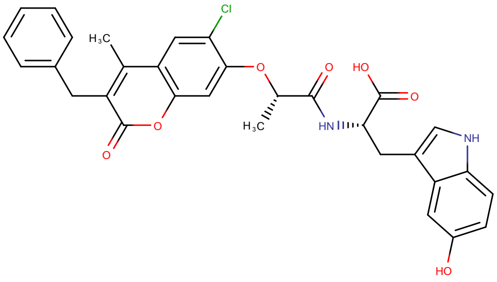	7.6	7.8	7.7
126	ZINC03846610	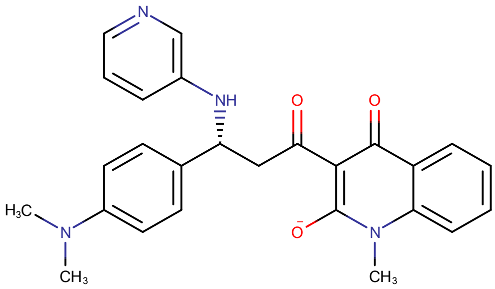	7.6	7.8	7.7
127	8159	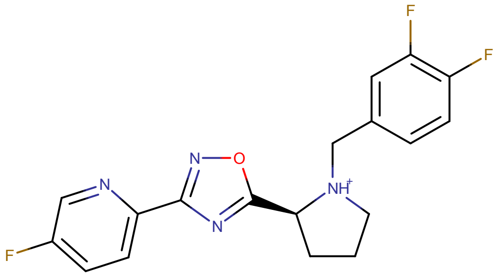	7.6	7.8	7.7

128	ZINC13181151	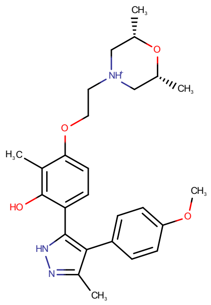	7.9	7.4	7.65
129	ZINC48057072	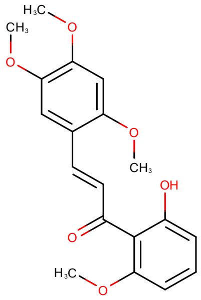	7.8	7.5	7.65
130	MolPort-002-524-694	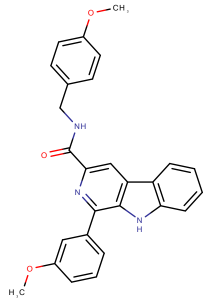	7.8	7.5	7.65
131	MolPort-002-520-663	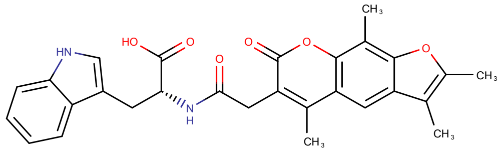	7.8	7.5	7.65
132	ZINC31170105	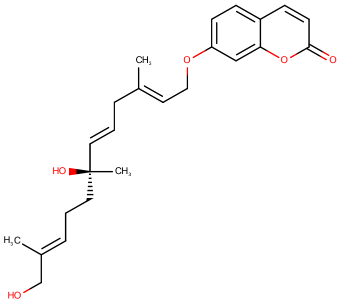	7.7	7.6	7.65

133	11064	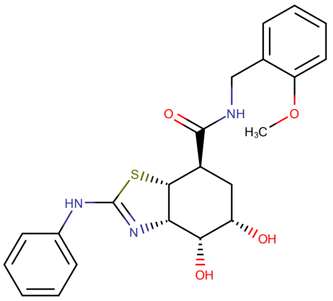	7.6	7.7	7.65
134	ZINC09410850	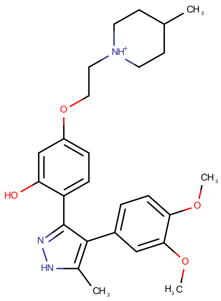	7.6	7.7	7.65
135	ZINC17058595	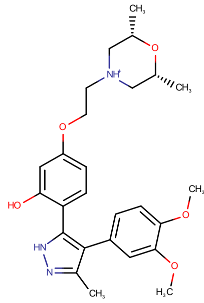	7.6	7.7	7.65
136	ZINC00083643	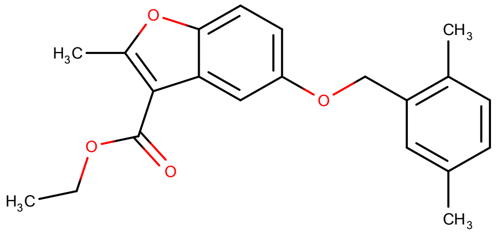	7.6	7.7	7.65
137	ZINC08790710	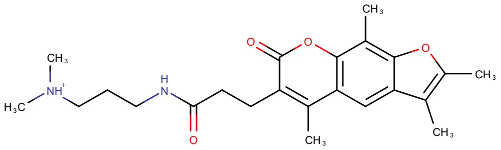	7.6	7.7	7.65

138	ZINC05156892	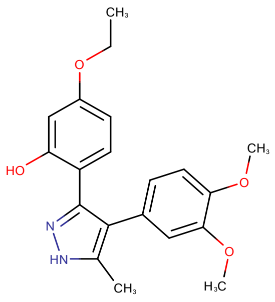	7.5	7.8	7.65
139	MolPort-021-745-971	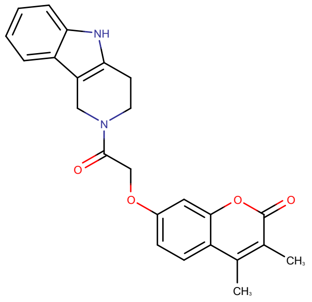	7.5	7.8	7.65
140	ZINC04236981	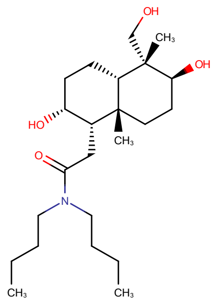	7.4	7.9	7.65
141	ZINC04090087	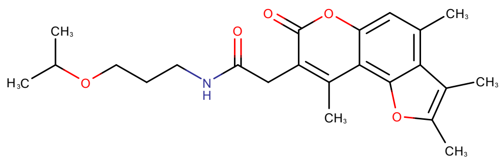	7.9	7.3	7.6
142	MolPort-002-530-479	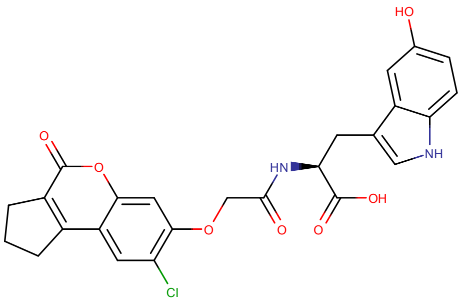	7.7	7.5	7.6

143	ZINC08993461	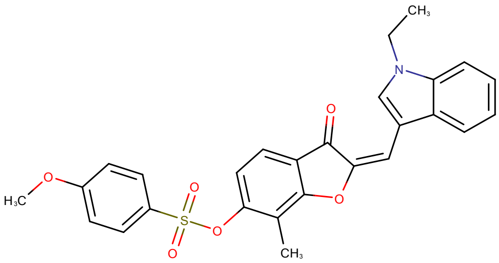	7.6	7.6	7.6
144	ZINC09059912	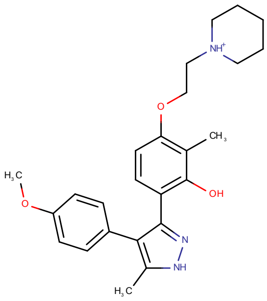	7.6	7.6	7.6
145	ZINC20466291	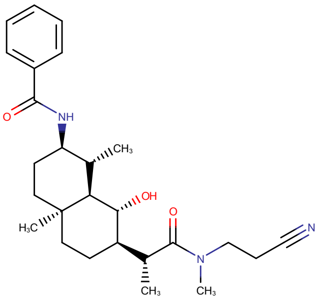	7.6	7.6	7.6
146	ZINC01321188	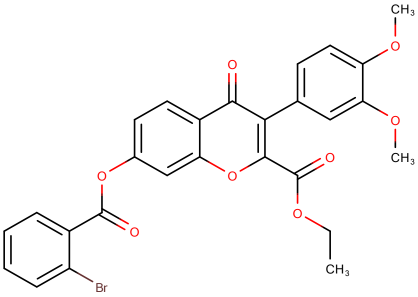	7.5	7.7	7.6
147	ZINC79188720	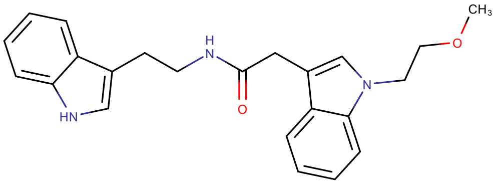	7.5	7.7	7.6

148	ZINC09128884	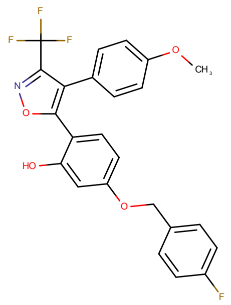	7.5	7.7	7.6
149	ZINC00627450	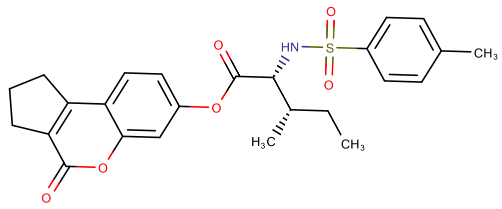	7.5	7.7	7.6
150	ZINC02147037	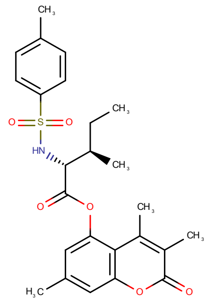	7.5	7.7	7.6
151	ZINC08789652	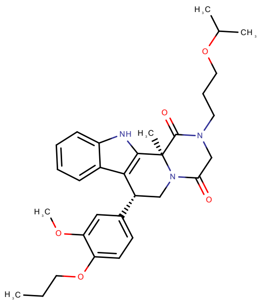	7.5	7.7	7.6
152	19285	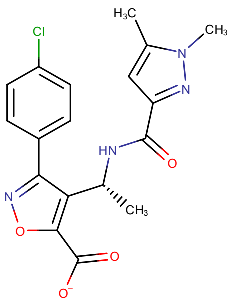	7.4	7.8	7.6

153	MolPort-039-057-114	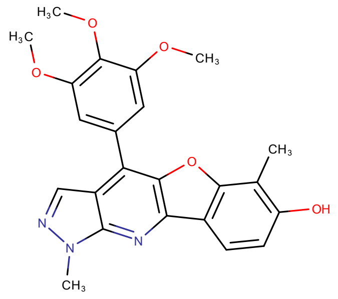	7.3	7.9	7.6
154	MolPort-002-518-975	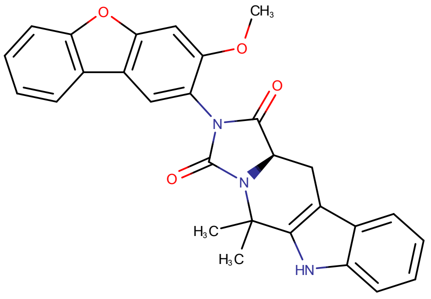	7.7	7.4	7.55
155	ZINC15675098	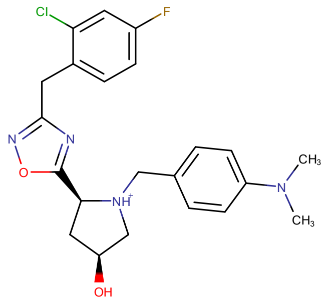	7.7	7.4	7.55
156	20512	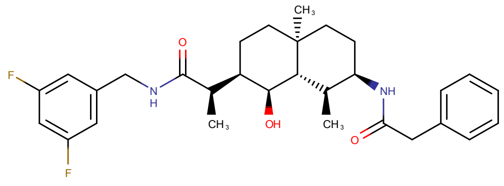	7.4	7.7	7.55
157	ZINC02316741	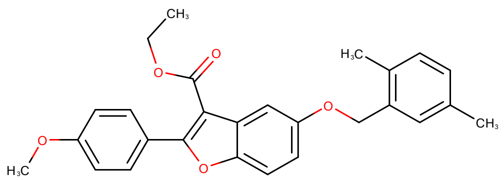	7.4	7.7	7.55

158	SN00147842	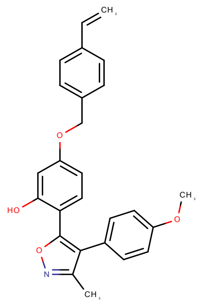	7.4	7.7	7.55
159	SN00149300	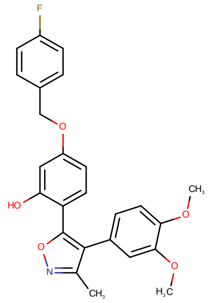	7.2	7.9	7.55
160	ZINC72324873	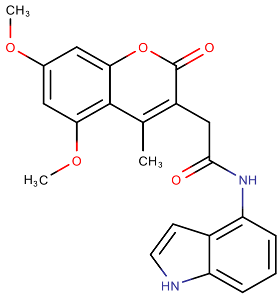	7.6	7.5	7.55
161	SN00151276	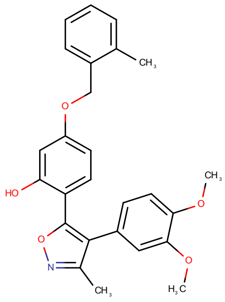	7.6	7.5	7.55
162	144369-45-9	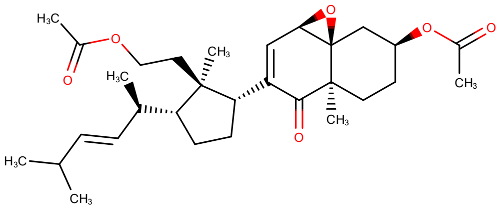	7.5	7.6	7.55

163	ZINC68592313	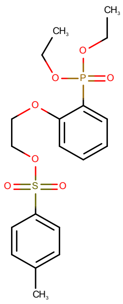	7.5	7.6	7.55
164	6915	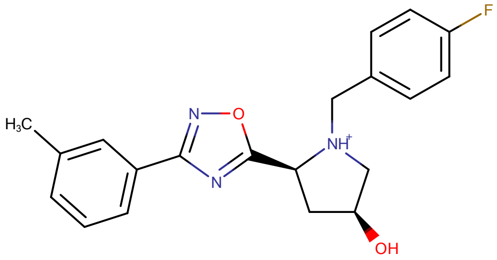	7.5	7.6	7.55
165	ZINC02284233	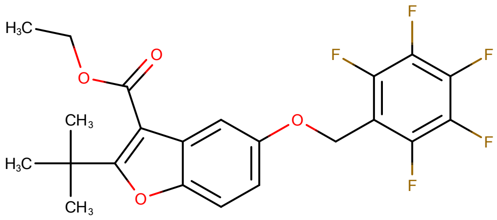	7.5	7.6	7.55
166	3205	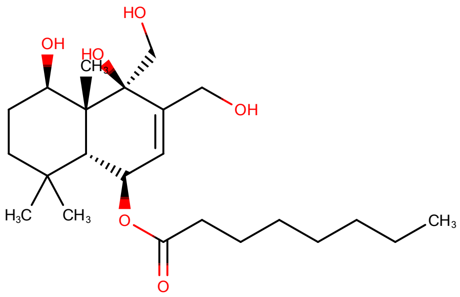	7.5	7.6	7.55
167	ZINC09128858	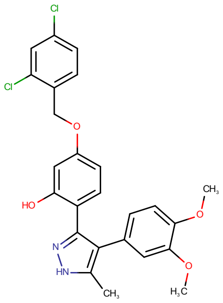	7.3	7.8	7.55

168	SN00149096	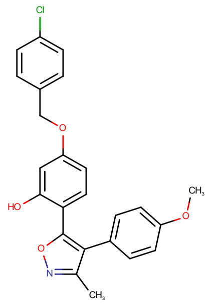	7.3	7.8	7.55
169	ZINC08918384	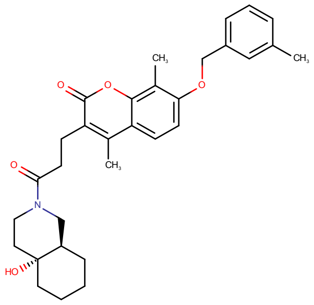	7.7	7.3	7.5
170	ZINC01322258	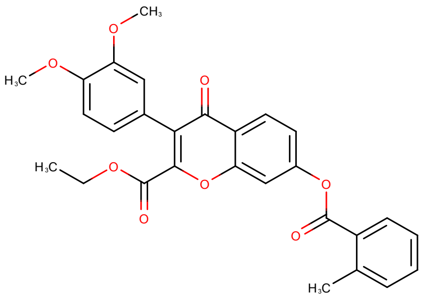	7.6	7.4	7.5
171	ZINC72324469	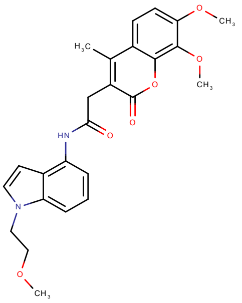	7.6	7.4	7.5
172	ZINC03846553	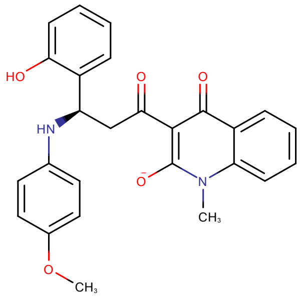	7.6	7.4	7.5

173	SN00278139	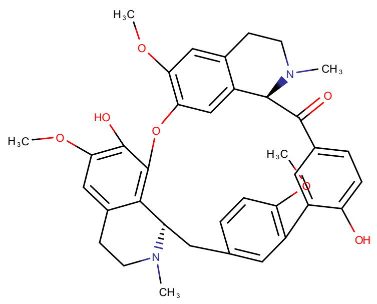	7.5	7.5	7.5
174	ZINC48057026	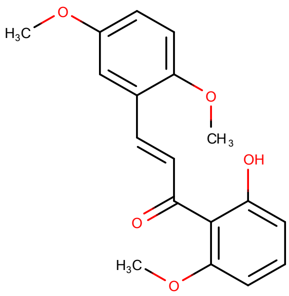	7.5	7.5	7.5
175	467419-73-4	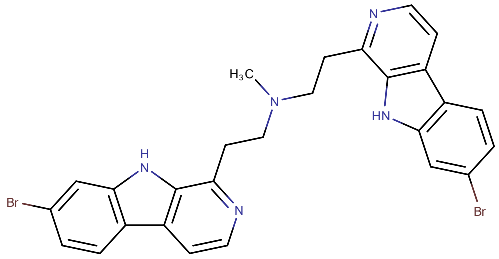	7.5	7.5	7.5
176	SN00149688	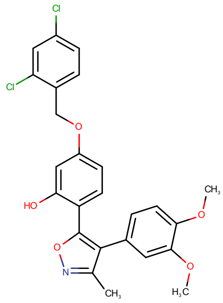	7.5	7.5	7.5
177	ZINC04171591	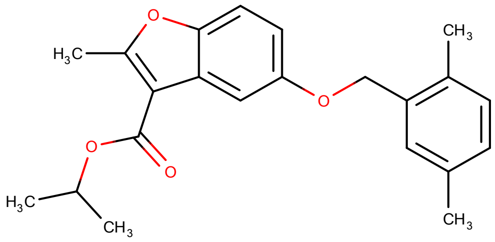	7.5	7.5	7.5

178	ZINC02096175	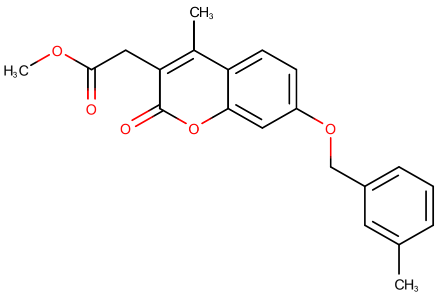	7.4	7.6	7.5
179	ZINC01782009	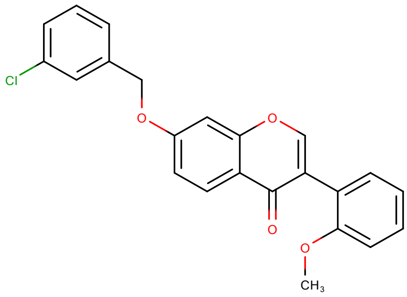	7.3	7.7	7.5
180	ZINC08994386	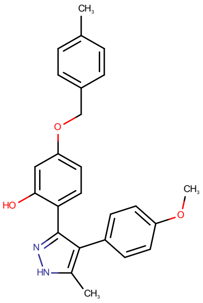	7.3	7.7	7.5
181	ZINC06719437	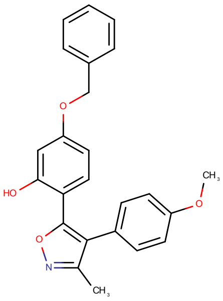	7.3	7.7	7.5
182	ZINC12865161	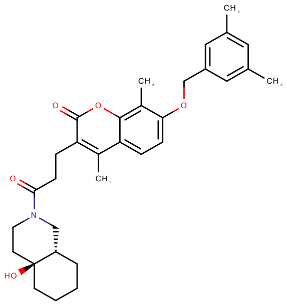	7.5	7.4	7.45

183	ZINC01321752	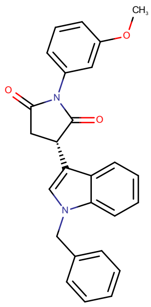	7.4	7.5	7.45
184	ZINC09129980	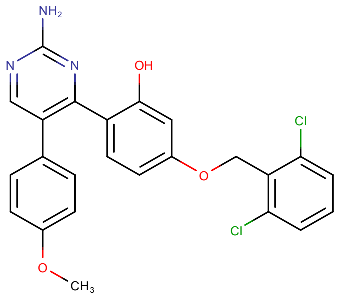	7.4	7.5	7.45
185	ZINC72325204	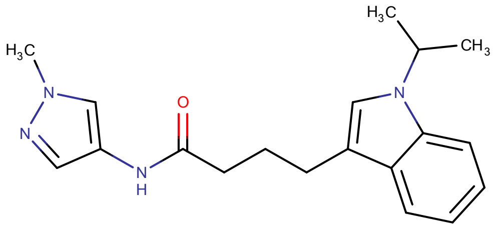	7.4	7.5	7.45
186	SN00009552	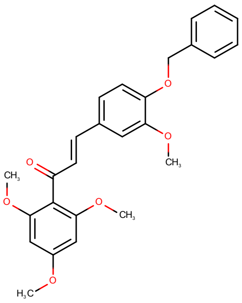	7.4	7.5	7.45
187	ZINC08764447	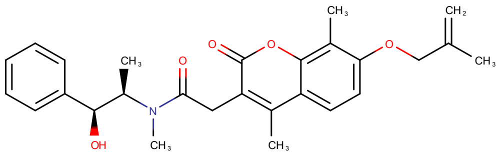	7.4	7.5	7.45

188	ZINC03840748	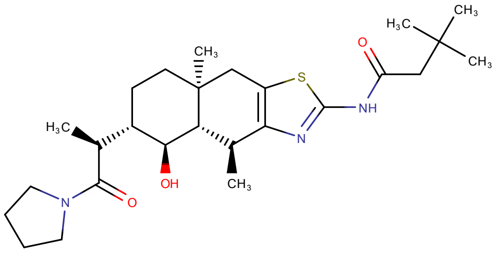	7.4	7.5	7.45
189	MolPort-002-516-108	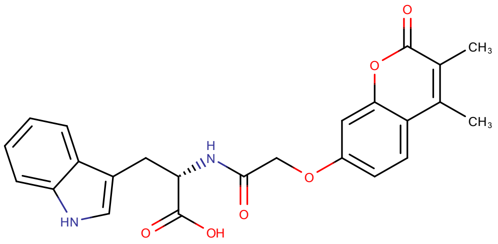	7.2	7.7	7.45
190	SN00149009	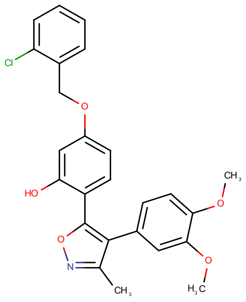	7.2	7.7	7.45
191	ZINC02148733	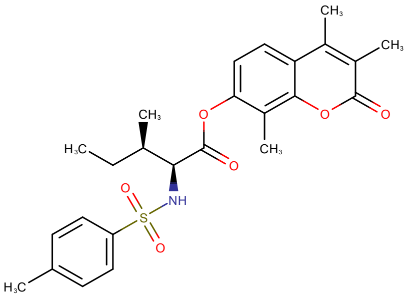	7.2	7.7	7.45
192	ZINC01799152	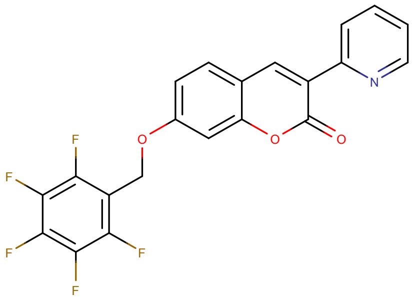	7.2	7.7	7.45

193	ZINC20591923	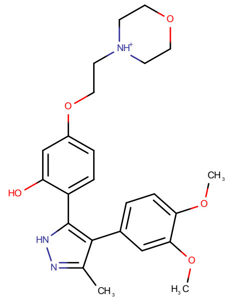	7.2	7.7	7.45
194	ZINC08764397	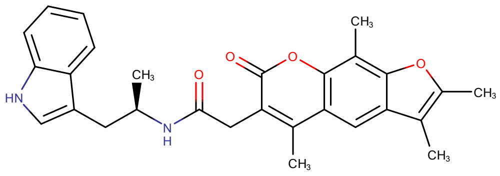	7.6	7.3	7.45
195	SN00149119	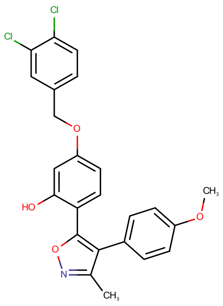	7.6	7.3	7.45
196	MolPort-027-852-800	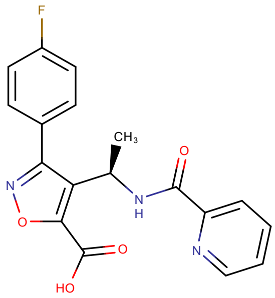	7.3	7.6	7.45
197	MolPort-027-852-111	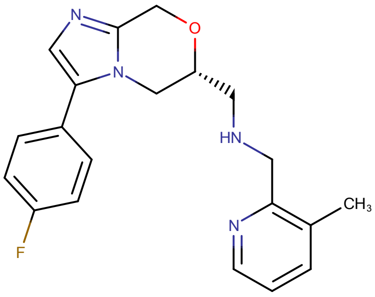	7.3	7.6	7.45

198	ZINC20592055	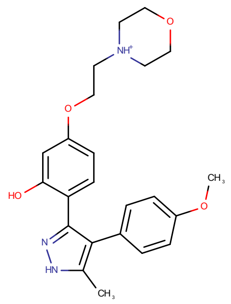	7.3	7.6	7.45
199	ZINC04026460	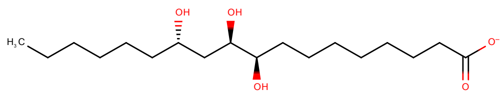	7.1	7.8	7.45
200	SN00245703	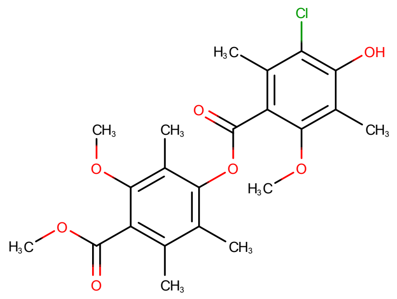	7.1	7.8	7.45
201	ZINC01124363	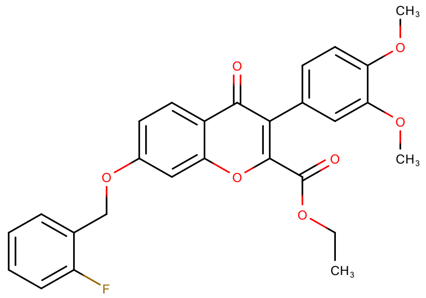	7.5	7.3	7.4
202	ZINC11867170	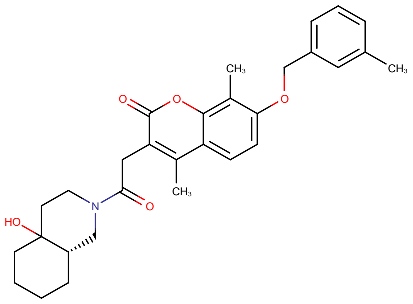	7.5	7.3	7.4

203	ZINC02401695	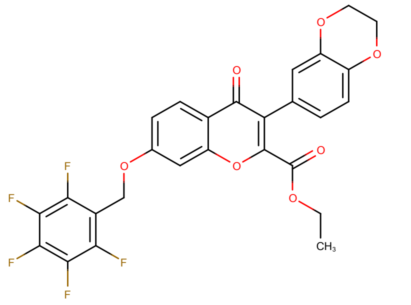	7.5	7.3	7.4
204	ZINC13569111	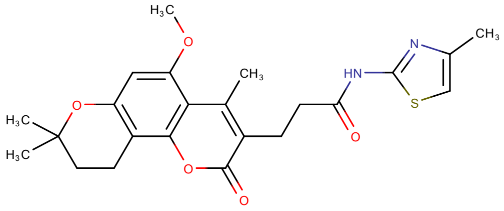	7.5	7.3	7.4
205	MolPort-002-514-023	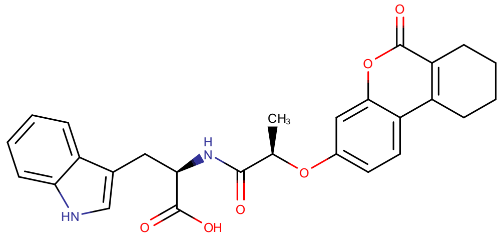	7.4	7.4	7.4
206	141544-63-0	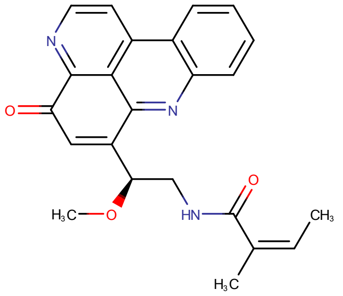	7.4	7.4	7.4
207	ZINC04090560	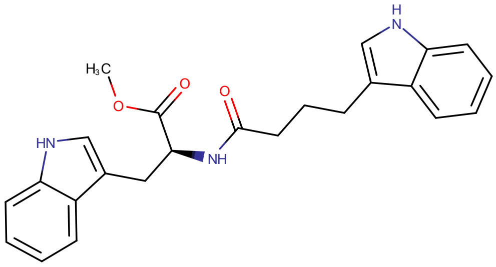	7.4	7.4	7.4

208	ZINC17058595	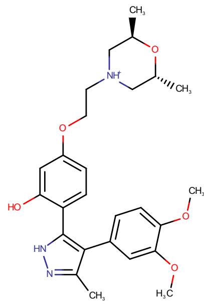	7.4	7.4	7.4
209	MolPort-001-745-794	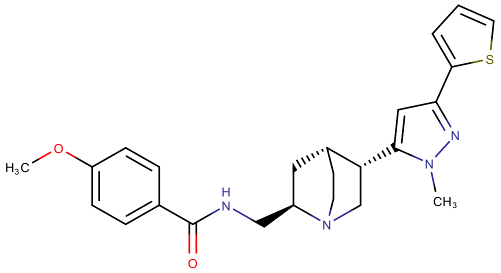	7.3	7.5	7.4
210	ZINC12875010	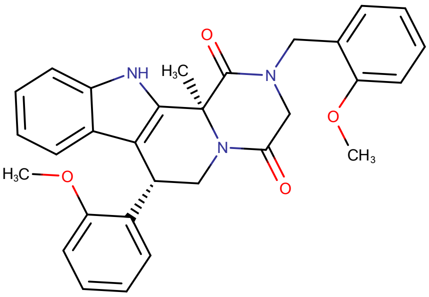	7.3	7.5	7.4
211	ZINC12865161	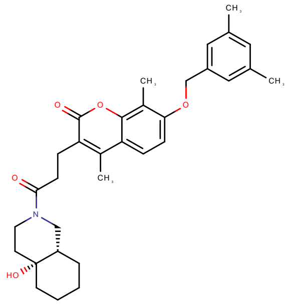	7.3	7.5	7.4
212	MolPort-002-536-594	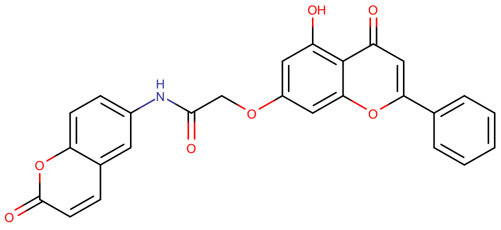	7.3	7.5	7.4

213	20208	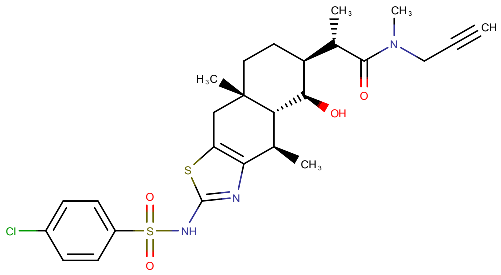	7.3	7.5	7.4
214	ZINC02115645	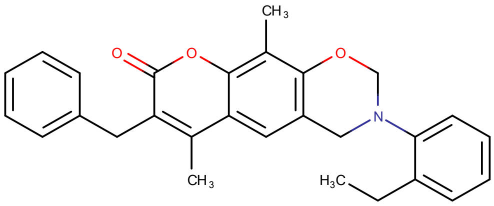	7.3	7.5	7.4
215	ZINC02149058	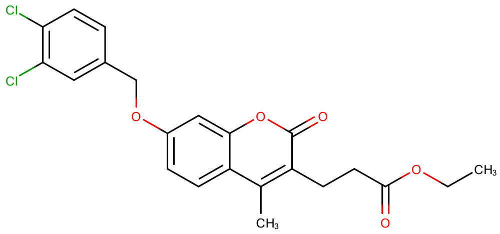	7.3	7.5	7.4
216	ZINC09130539	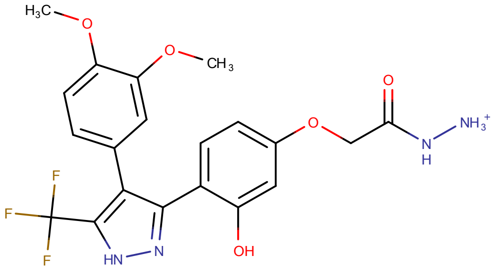	7.2	7.6	7.4
217	ZINC03150514	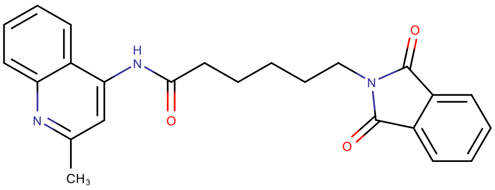	7.2	7.6	7.4

218	ZINC02359452	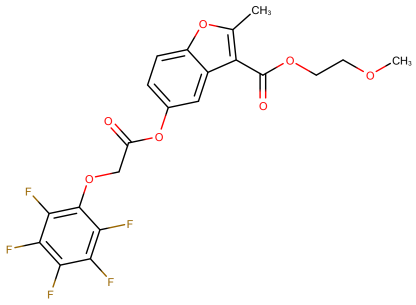	7.2	7.6	7.4
219	ZINC01321303	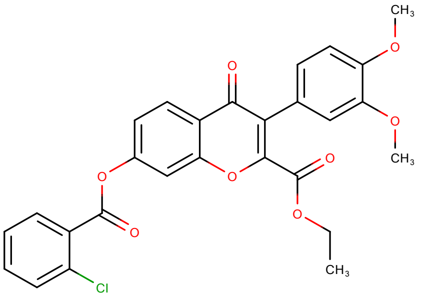	7.5	7.2	7.35
220	ZINC02094744	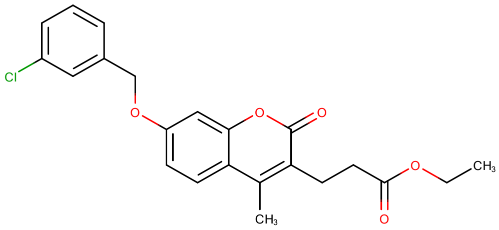	7.5	7.2	7.35
221	ZINC08789240	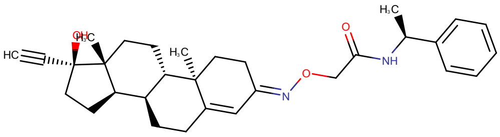	7.4	7.3	7.35
222	MolPort-042-647-983	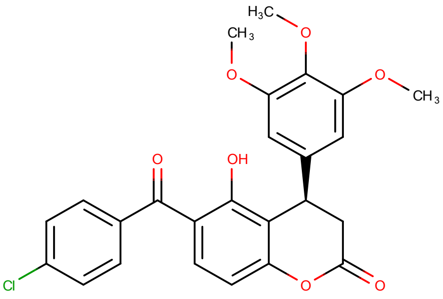	7.4	7.3	7.35

223	SN00359873	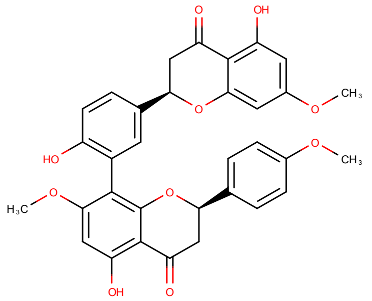	7.4	7.3	7.35
224	ZINC20466656	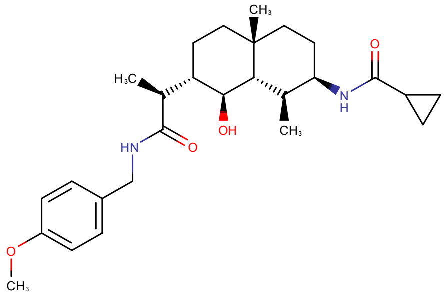	7.4	7.3	7.35
225	ZINC02417488	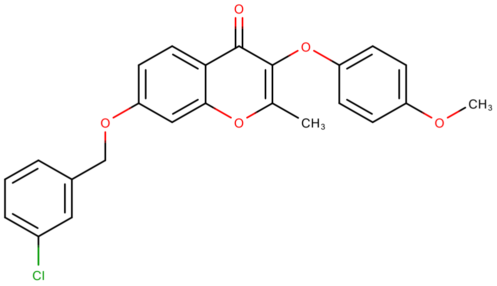	7.4	7.3	7.35
226	ZINC02416730	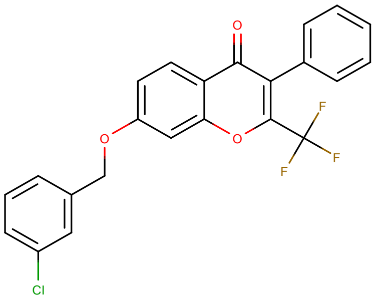	7.4	7.3	7.35
227	ZINC85879450	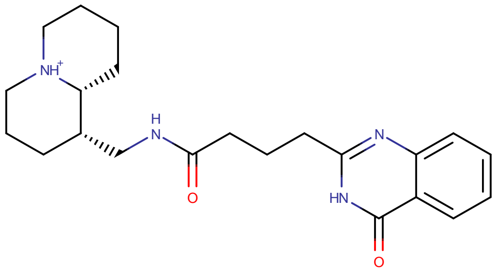	7.3	7.4	7.35

228	ZINC02148395	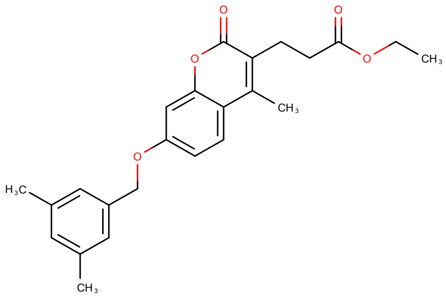	7.3	7.4	7.35
229	ZINC08764768	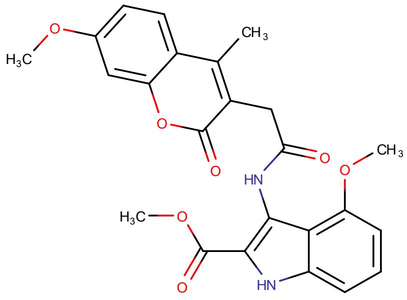	7.3	7.4	7.35
230	121979-39-3	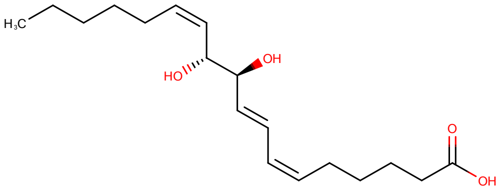	7.3	7.4	7.35
231	ZINC70701314	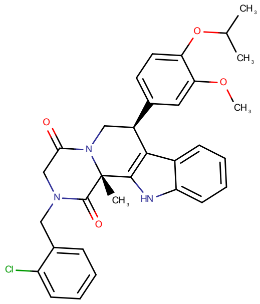	7.3	7.4	7.35
232	ZINC48057026	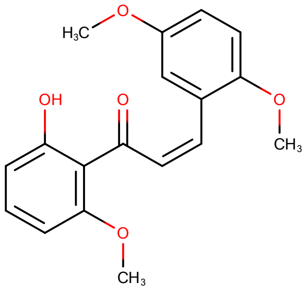	7.3	7.4	7.35

233	ZINC03875039	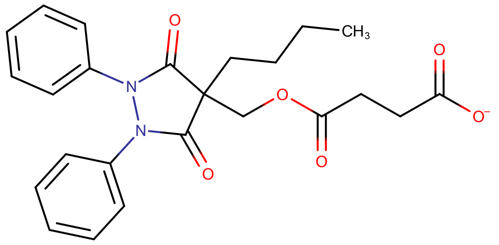	7.2	7.5	7.35
234	ZINC02148180	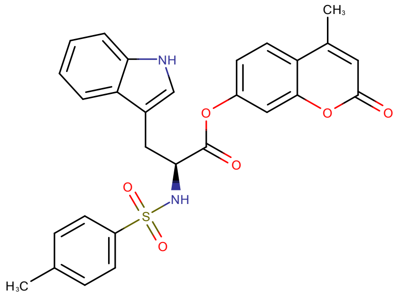	7.2	7.5	7.35
235	ZINC02419763	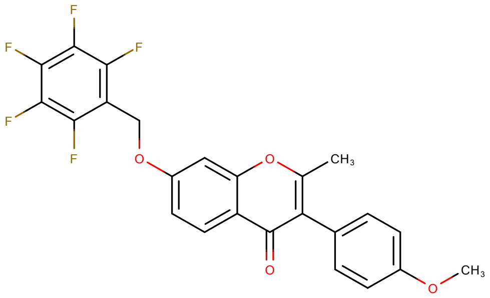	7.2	7.5	7.35
236	ZINC02432591	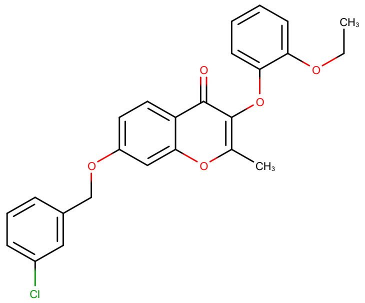	7.1	7.6	7.35
237	ZINC18068961	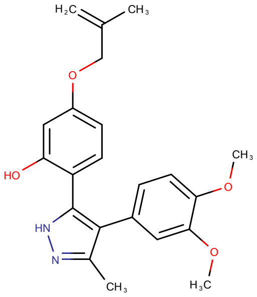	7.1	7.6	7.35

238	ZINC01322844	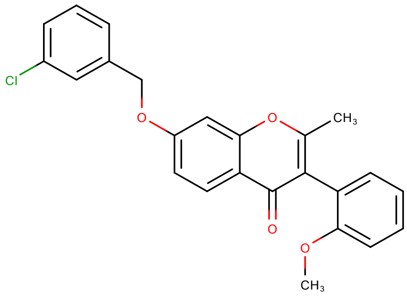	7	7.7	7.35
239	ZINC12603405	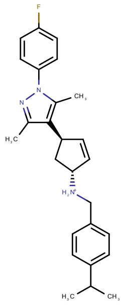	7.4	7.2	7.3
240	MolPort-010-738-591	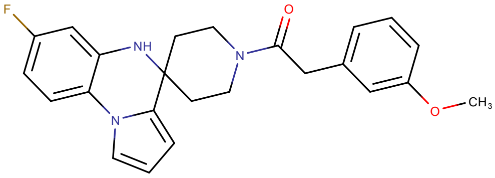	7.4	7.2	7.3
241	114820-25-6	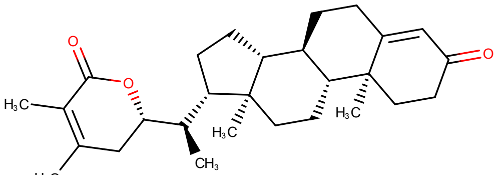	7.4	7.2	7.3
242	ZINC00947089	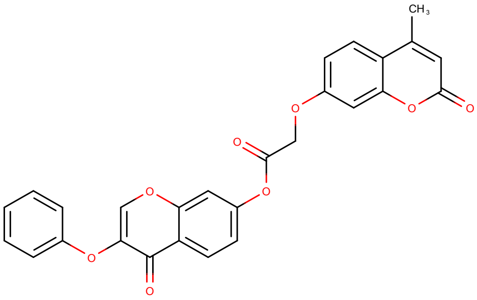	7.4	7.2	7.3

243	SN00009667	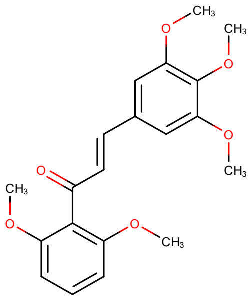	7.4	7.2	7.3
244	ZINC12897517	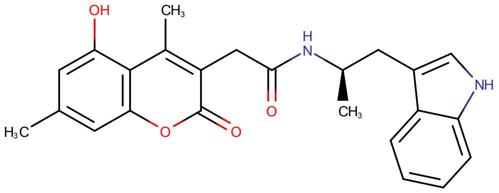	7.4	7.2	7.3
245	MolPort-038-386-307	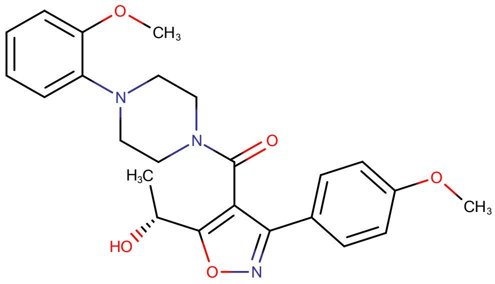	7.2	7.4	7.3
246	105377-88-6	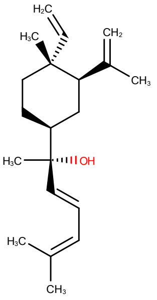	7.2	7.4	7.3
247	ZINC05427801	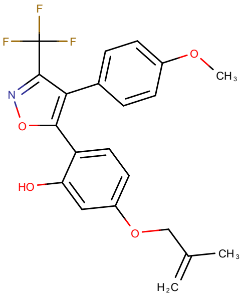	7.2	7.4	7.3

248	ZINC02316868	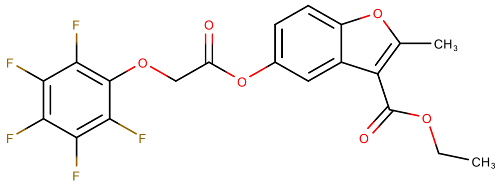	7.2	7.4	7.3
249	MolPort-028-854-802	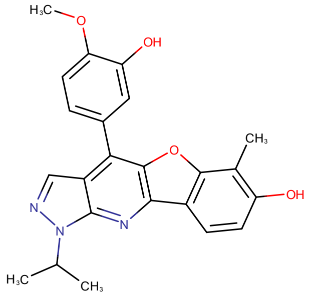	7.2	7.4	7.3
250	MolPort-002-533-316	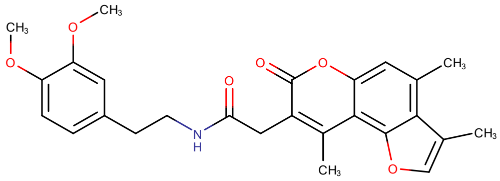	7.2	7.4	7.3
251	SN00345792	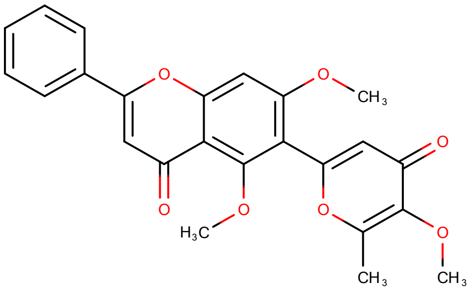	7.2	7.4	7.3
252	MolPort-002-518-869	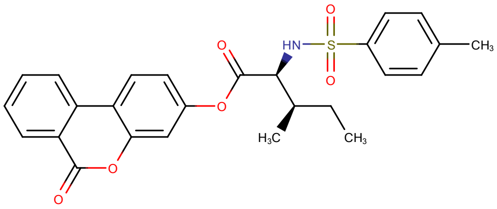	7.2	7.4	7.3

253	ZINC01797720	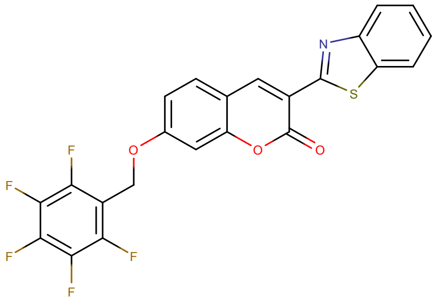	6.9	7.7	7.3
254	MolPort-029-886-452	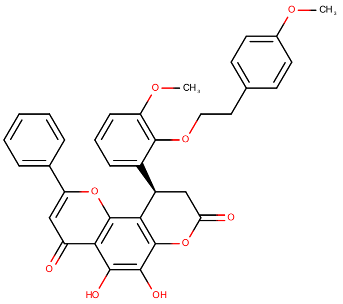	7.3	7.3	7.3
255	ZINC79210340	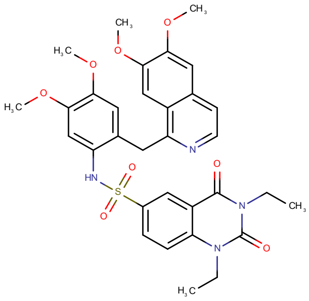	7.3	7.3	7.3
256	9853	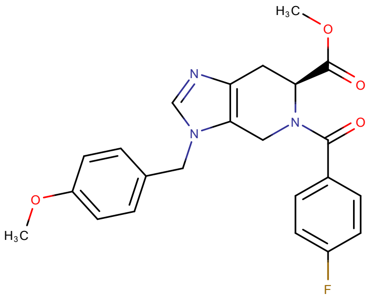	7.3	7.3	7.3
257	ZINC68573697	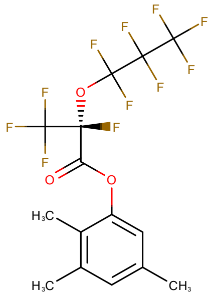	7.3	7.3	7.3

258	MolPort-000-813-035	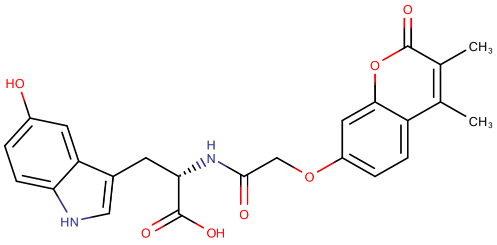	7.3	7.3	7.3
259	MolPort-028-855-502	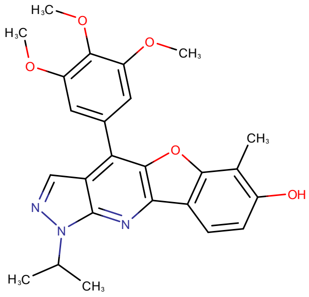	7.3	7.3	7.3
260	ZINC12865161	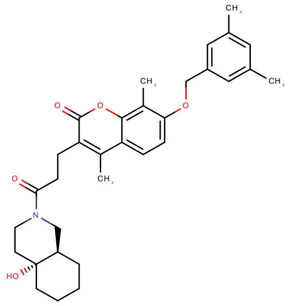	7.3	7.3	7.3
261	ZINC20622826	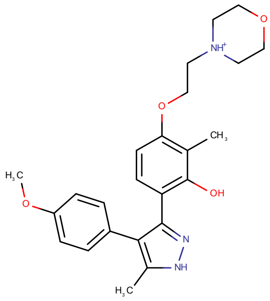	7.3	7.3	7.3
262	ZINC17858262	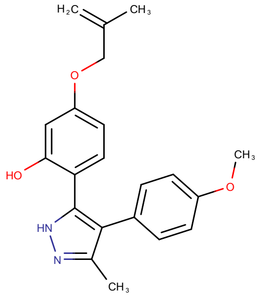	7.3	7.3	7.3

263	ZINC02147827	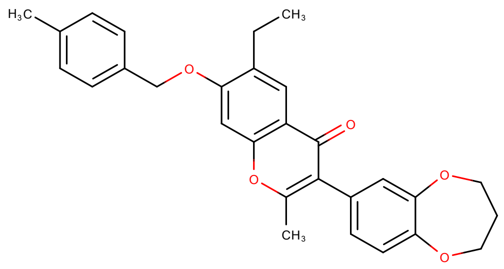	7.3	7.3	7.3
264	SN00149094	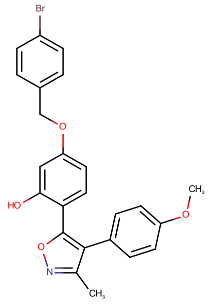	7.3	7.3	7.3
265	ZINC13124861	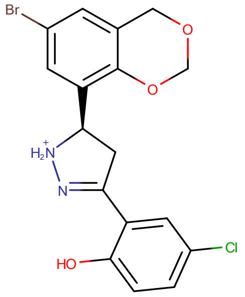	7.3	7.3	7.3
266	MolPort-002-520-217	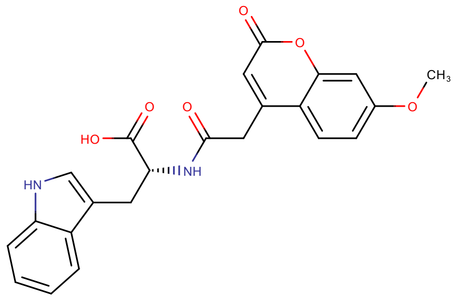	7.1	7.5	7.3
267	MolPort-002-519-718	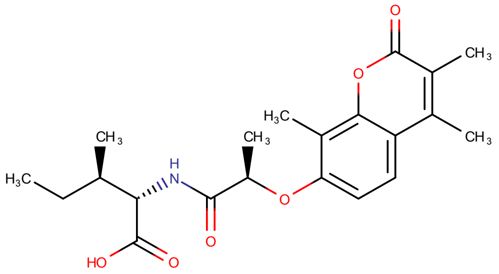	7.1	7.5	7.3

268	ZINC02115916	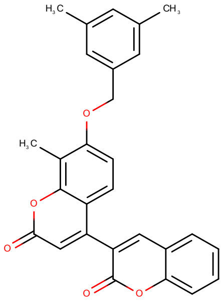	7.1	7.5	7.3
269	ZINC06755178	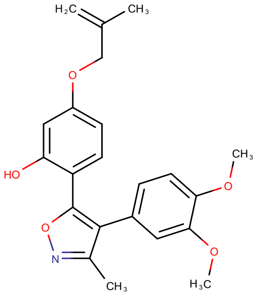	7.1	7.5	7.3
270	ZINC09130710	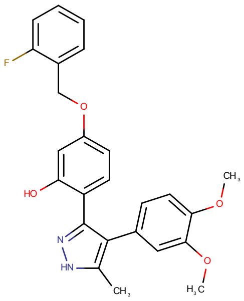	7.1	7.5	7.3
271	SN00098390	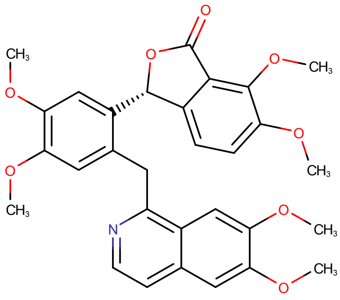	7.1	7.5	7.3
272	ZINC70692199	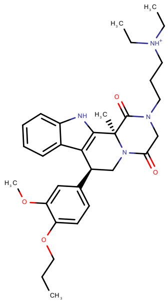	7.1	7.5	7.3

273	MolPort-002-535-528	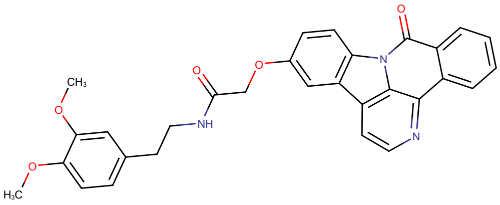	7	7.6	7.3
274	SN00379562	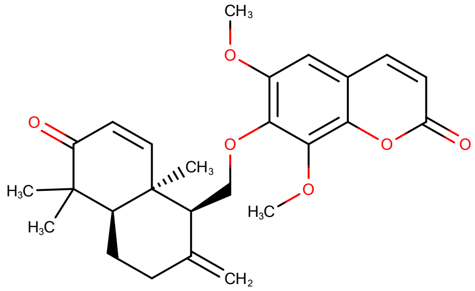	7	7.6	7.3
275	SN00351686	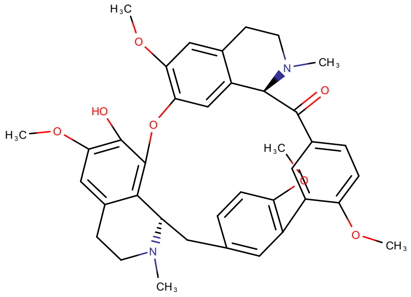	7.4	7.1	7.25
276	SN00168965	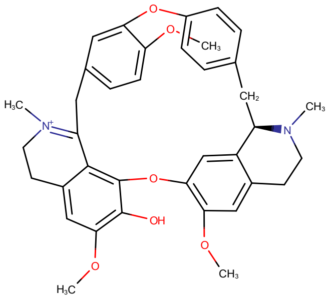	7.4	7.1	7.25
277	ZINC20466317	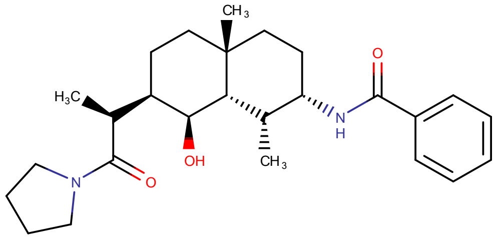	7.4	7.1	7.25

278	ZINC02112486	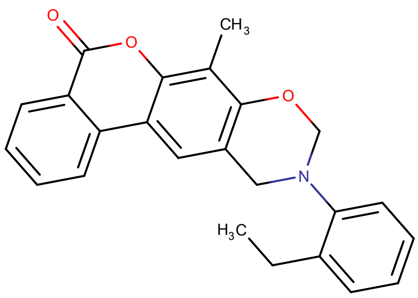	7.3	7.2	7.25
279	6895	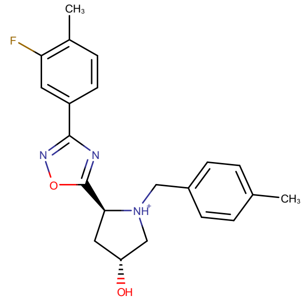	7.3	7.2	7.25
280	MolPort-029-885-299	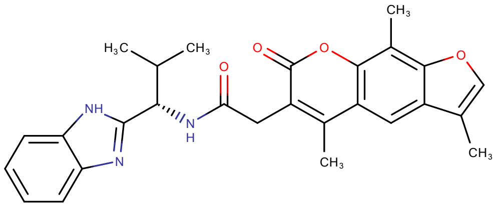	7.3	7.2	7.25
281	MolPort-002-536-261	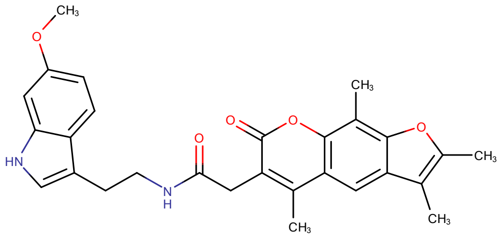	7.3	7.2	7.25
282	ZINC12296352	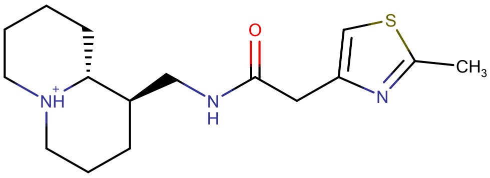	7.3	7.2	7.25

283	MolPort-038-386-306	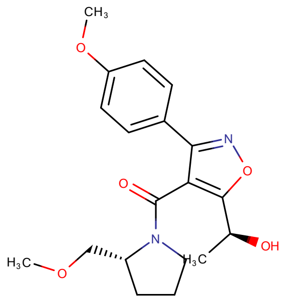	7.3	7.2	7.25
284	ZINC13653587	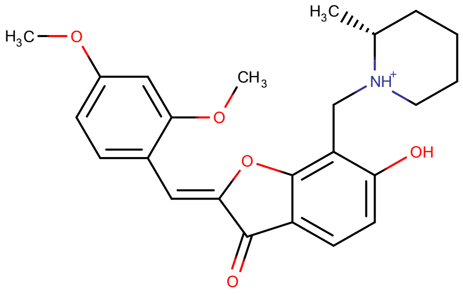	7.2	7.3	7.25
285	ZINC04061925	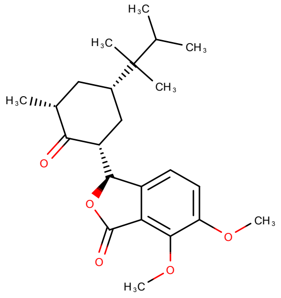	7.2	7.3	7.25
286	MolPort-002-532-941	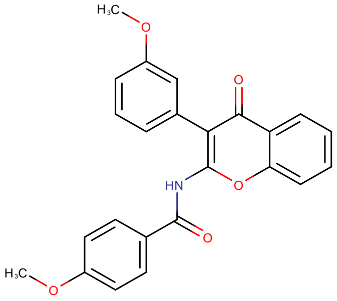	7.1	7.4	7.25
287	ZINC08879630	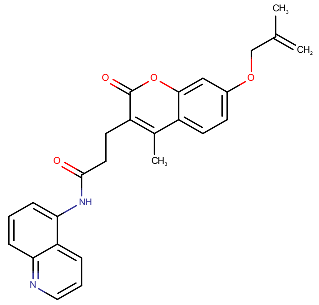	7.1	7.4	7.25

288	ZINC30879908	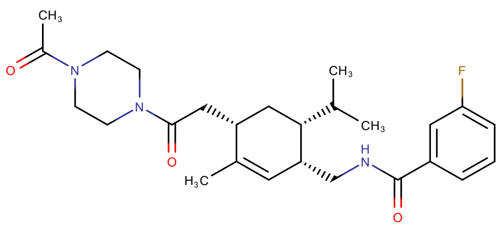	7.1	7.4	7.25
289	ZINC03846576	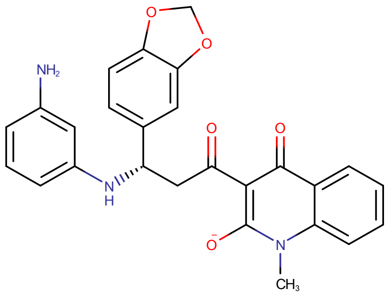	7.1	7.4	7.25
290	ZINC00083661	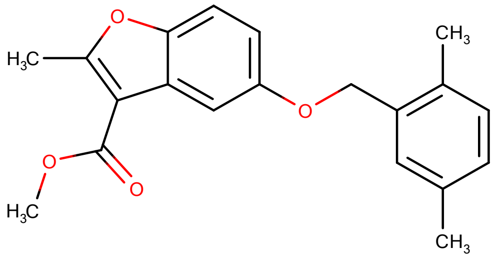	7.1	7.4	7.25
291	ZINC02096181	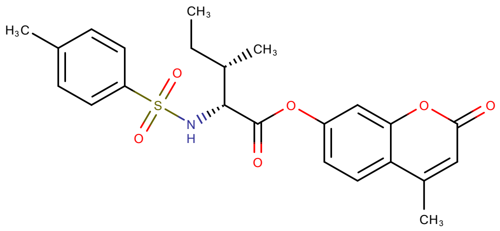	6.8	7.7	7.25
292	ZINC11867175	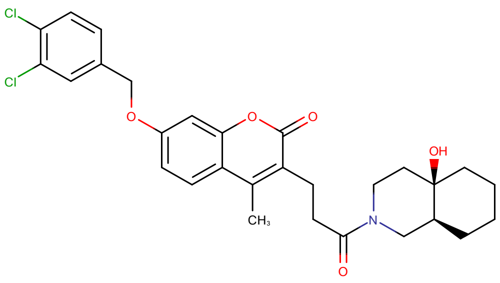	7.4	7	7.2

293	MolPort-028-855-910	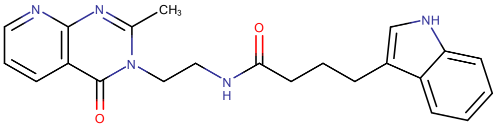	7.2	7.2	7.2
294	ZINC02817963	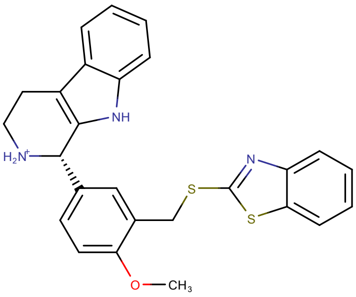	7.2	7.2	7.2
295	ZINC62001394	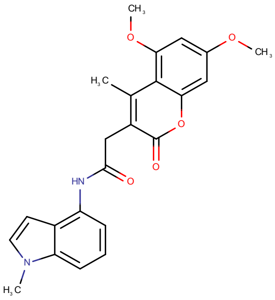	7.2	7.2	7.2
296	ZINC02092024	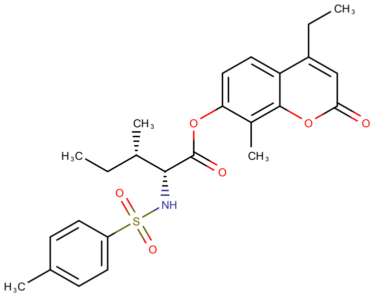	7.2	7.2	7.2
297	ZINC08791608	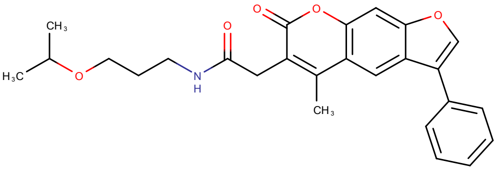	7.2	7.2	7.2

298	8166	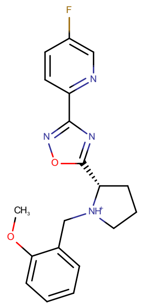	7.2	7.2	7.2
299	ZINC02105206	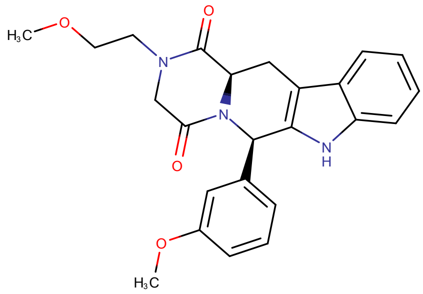	7	7.4	7.2
300	MolPort-005-910-295	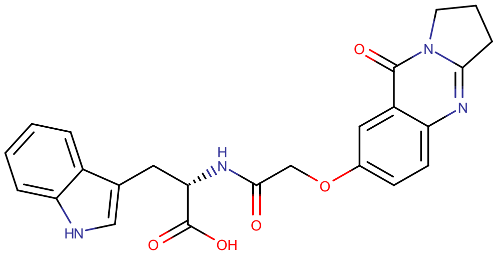	7	7.4	7.2
301	ZINC04025067	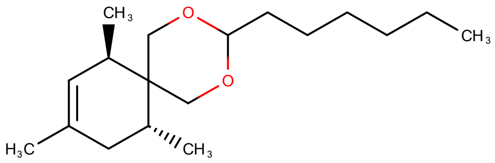	7	7.4	7.2
302	ZINC09130732	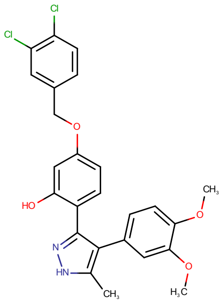	7	7.4	7.2

303	ZINC02092577	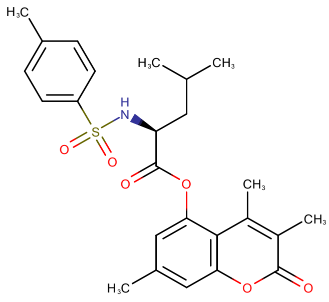	6.9	7.5	7.2
304	SN00268509	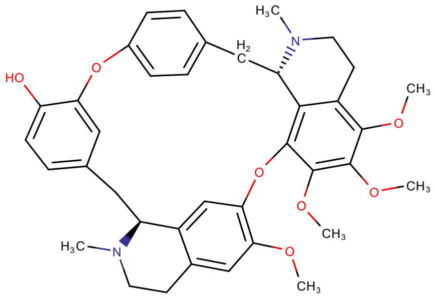	6.9	7.5	7.2
305	ZINC12603350	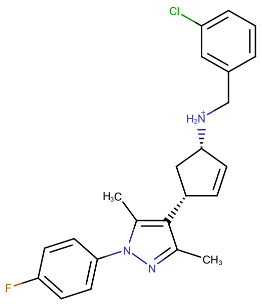	7.3	7.1	7.2
306	ZINC30883993	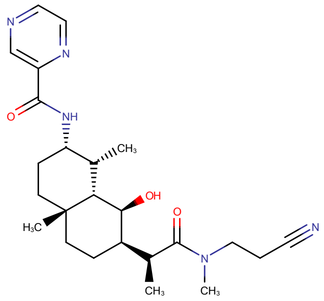	7.3	7.1	7.2
307	MolPort-001-742-517	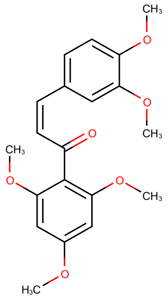	7.3	7.1	7.2

308	ZINC02115467	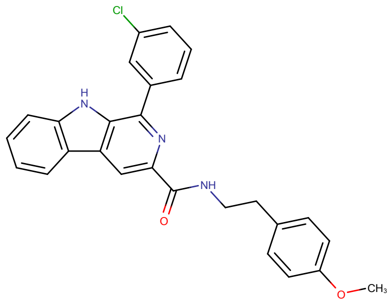	7.3	7.1	7.2
309	MolPort-002-517-246	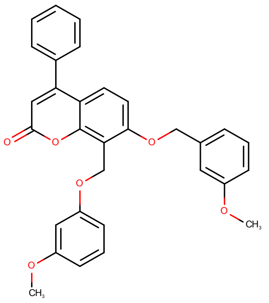	7.1	7.3	7.2
310	ZINC02107236	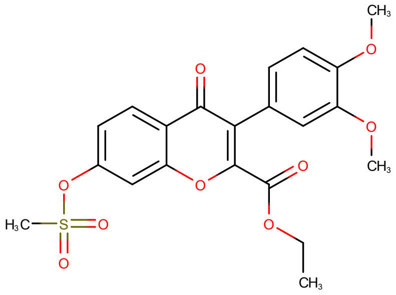	7.1	7.3	7.2
311	ZINC68601377	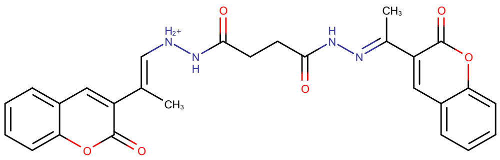	7.1	7.3	7.2
312	ZINC70692423	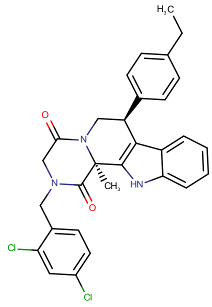	7.1	7.3	7.2

313	MolPort-044-544-310	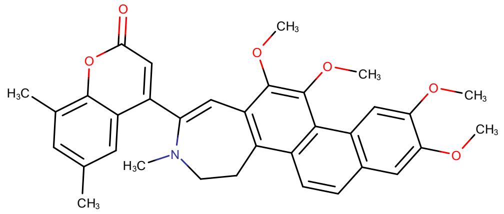	7.1	7.3	7.2
314	ZINC04387787	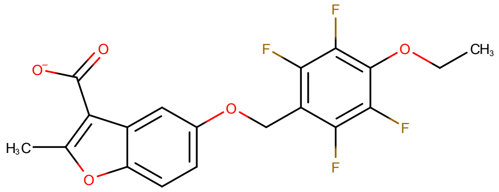	7.1	7.3	7.2
315	1877	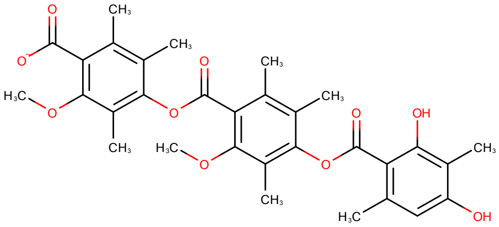	7.1	7.3	7.2
316	ZINC08479128	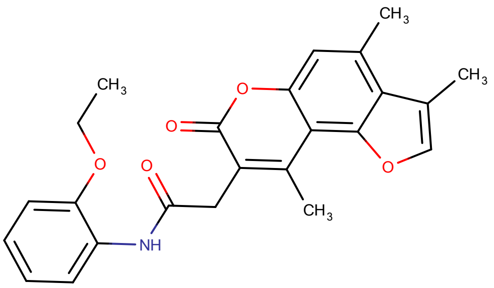	7.1	7.3	7.2
317	ZINC04100775	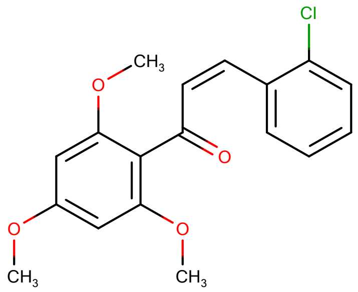	7.1	7.3	7.2

318	ZINC01111774	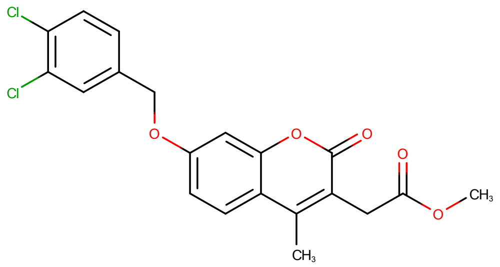	7.1	7.3	7.2
319	ZINC01118985	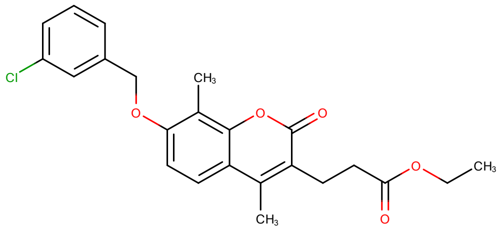	7.1	7.3	7.2
320	SN00063216	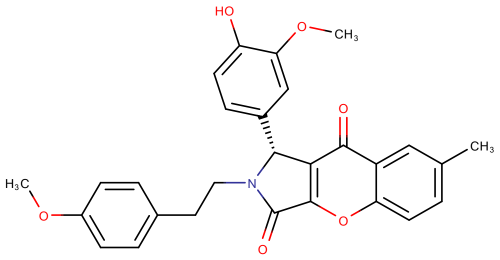	7.4	6.9	7.15
321	ZINC02156156	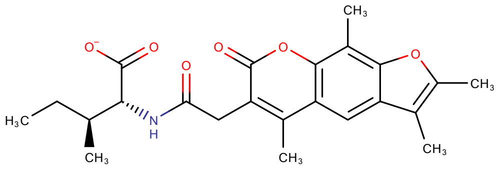	7.4	6.9	7.15
322	ZINC02091864	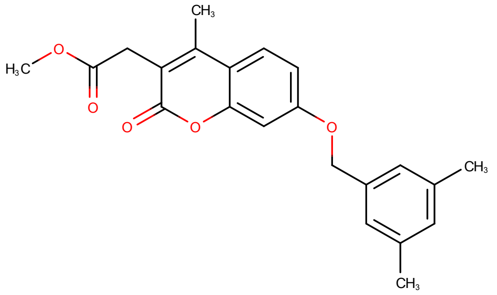	7.4	6.9	7.15

323	MolPort-029-886-141	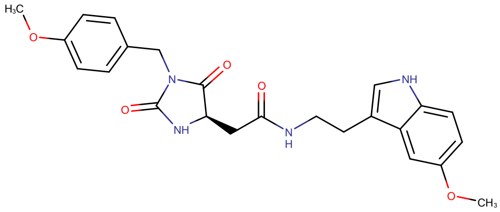	7.4	6.9	7.15
324	ZINC35425888	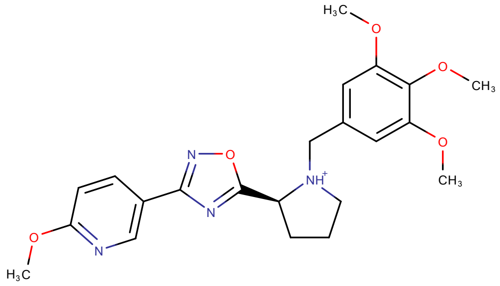	7.3	7	7.15
325	SN00155517	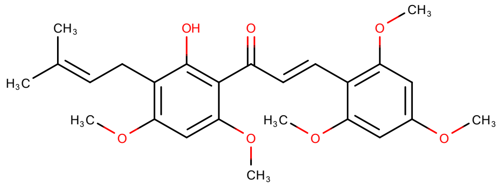	7.2	7.1	7.15
326	ZINC20611253	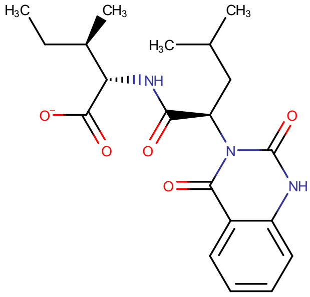	7.2	7.1	7.15
327	ZINC08764272	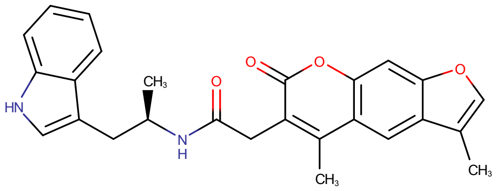	7.2	7.1	7.15

328	ZINC03840780	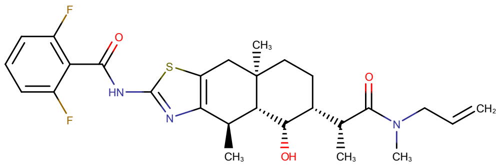	7.2	7.1	7.15
329	ZINC04252739	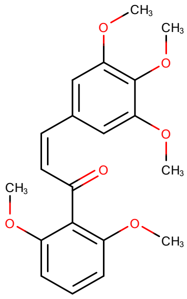	7.2	7.1	7.15
330	ZINC20757263	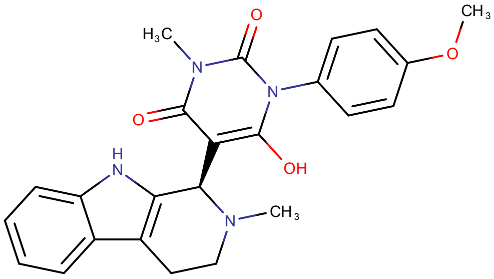	7.1	7.2	7.15
331	SN00077518	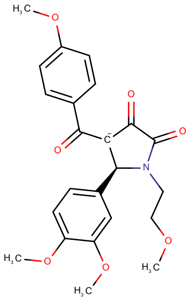	7.1	7.2	7.15
332	ZINC12891809	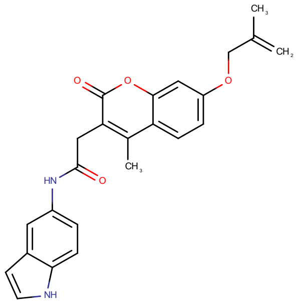	7.1	7.2	7.15

333	ZINC02094302	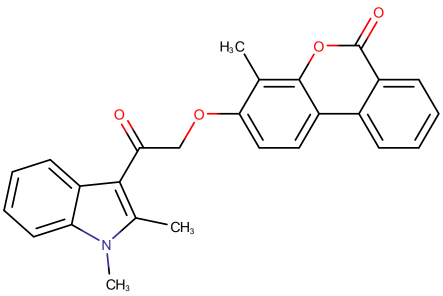	7.1	7.2	7.15
334	ZINC70666401	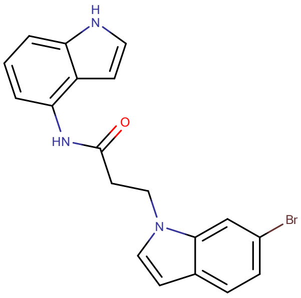	7.1	7.2	7.15
335	ZINC13127298	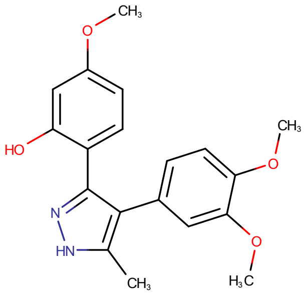	7	7.3	7.15
336	ZINC79189085	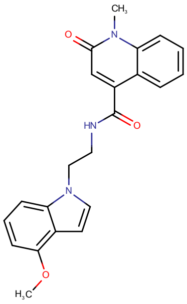	7	7.3	7.15
337	ZINC01111387	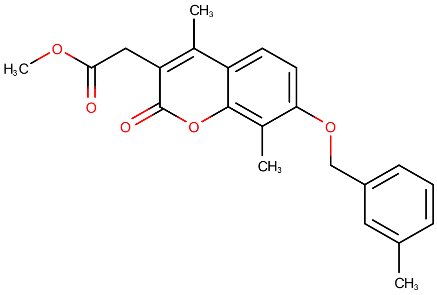	7	7.3	7.15

338	ZINC02159445	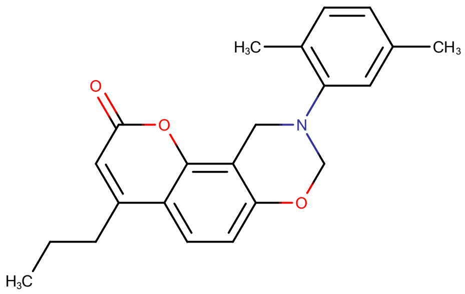	7	7.3	7.15
339	MolPort-044-830-921	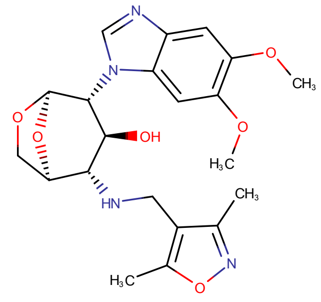	7	7.3	7.15
340	ZINC70687971	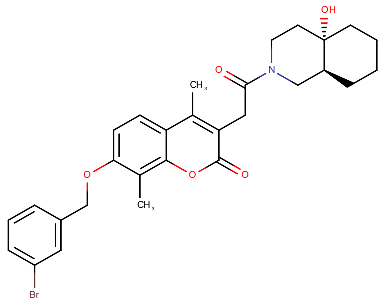	7	7.3	7.15
341	SN00151294	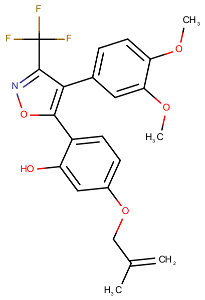	6.9	7.4	7.15
342	MolPort-009-649-059	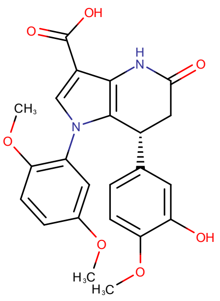	6.9	7.4	7.15

343	ZINC01322799	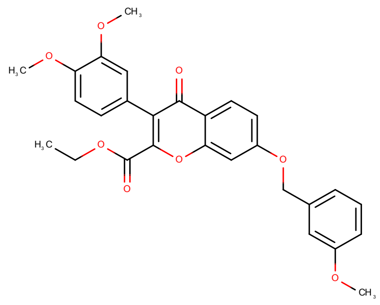	6.9	7.4	7.15
344	ZINC12901891	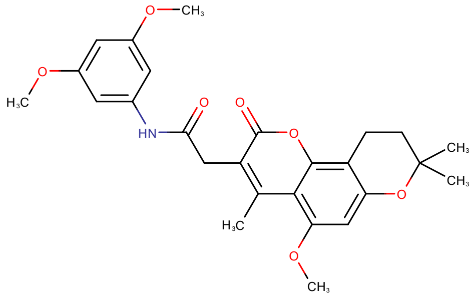	6.9	7.4	7.15
345	12169	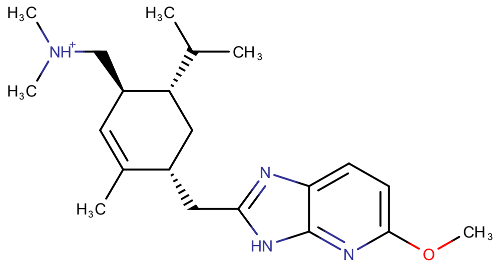	7.2	7	7.1
346	ZINC02093820	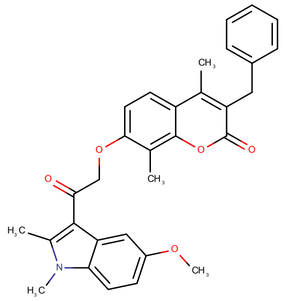	7.2	7	7.1
347	ZINC01447881	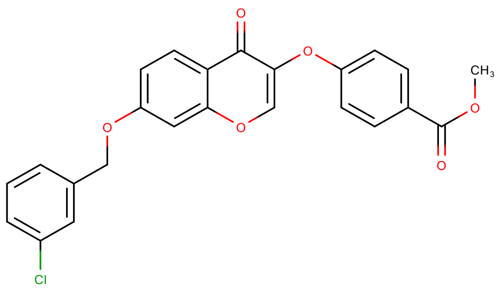	7.2	7	7.1

348	ZINC09266649	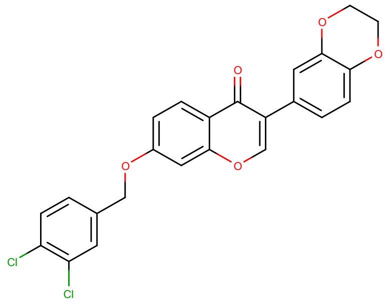	7.2	7	7.1
349	ZINC12881699	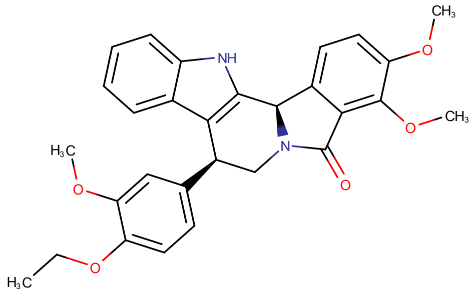	7.1	7.1	7.1
350	78518-73-7	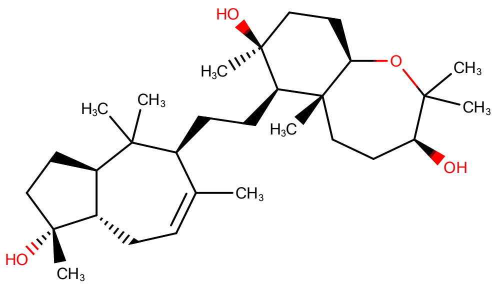	7.1	7.1	7.1
351	ZINC20467354	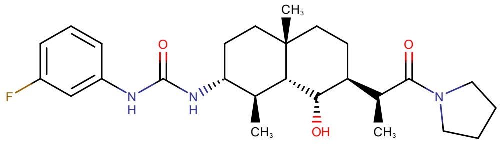	7.1	7.1	7.1
352	MolPort-028-855-487	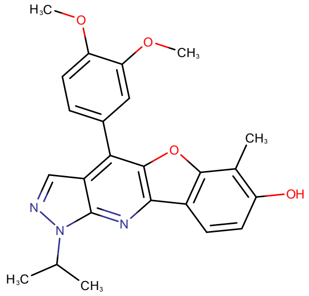	7.1	7.1	7.1

353	ZINC04252699	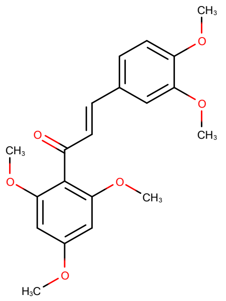	7.1	7.1	7.1
354	ZINC01797677	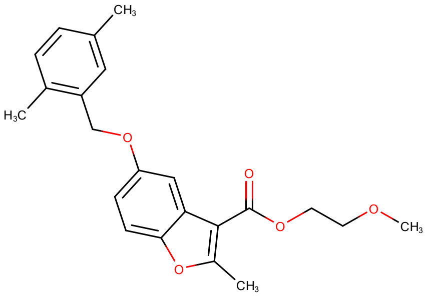	7.1	7.1	7.1
355	ZINC02122276	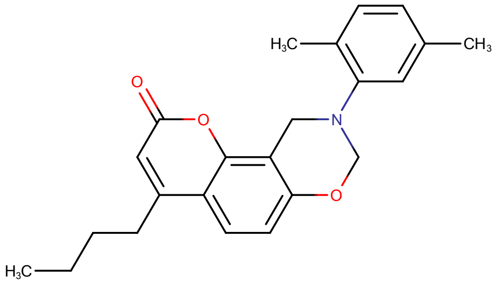	7	7.2	7.1
356	ZINC08993442	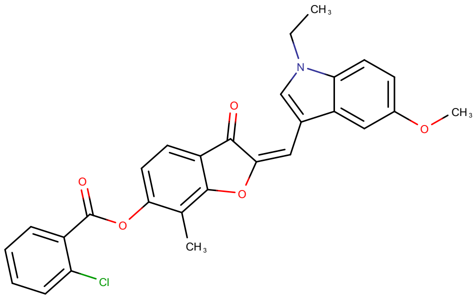	6.9	7.3	7.1
357	SN00151259	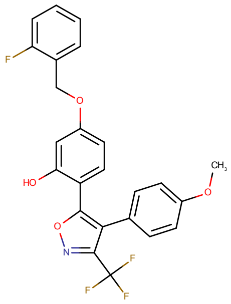	6.9	7.3	7.1

358	ZINC02122006	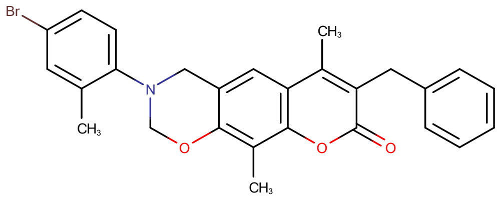	6.9	7.3	7.1
359	ZINC08739938	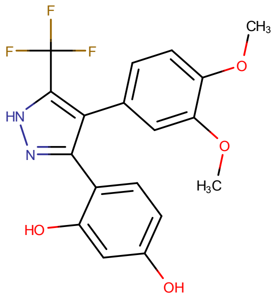	6.9	7.3	7.1
360	ZINC02149345	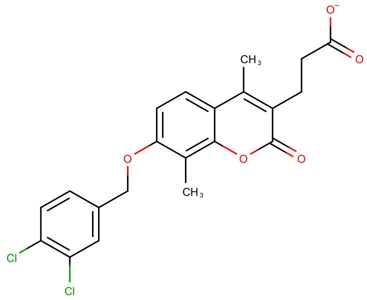	6.8	7.4	7.1
361	ZINC20111812	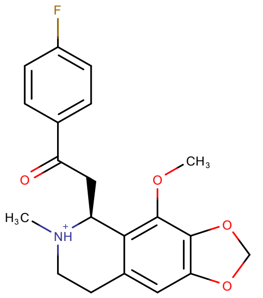	7.2	6.9	7.05
362	20391	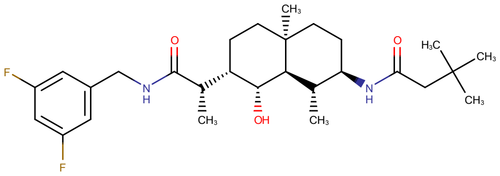	7.2	6.9	7.05

363	ZINC15674342	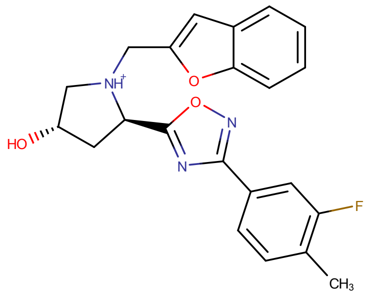	7.2	6.9	7.05
364	MolPort-001-910-598	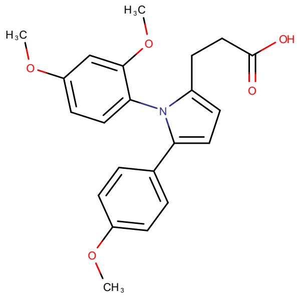	6.9	7.2	7.05
365	MolPort-002-525-441	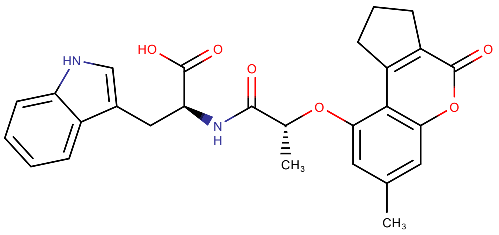	6.9	7.2	7.05
366	MolPort-001-838-316	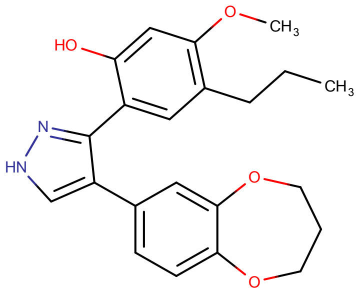	6.9	7.2	7.05
367	ZINC01448111	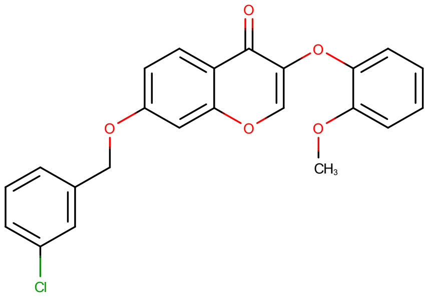	6.9	7.2	7.05

368	19304	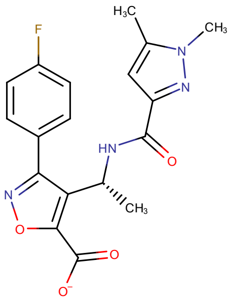	6.7	7.4	7.05
369	169340-01-6	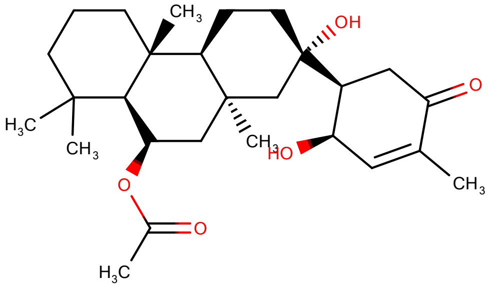	6.7	7.4	7.05
370	ZINC30883259	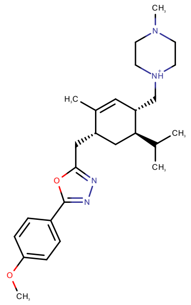	7.3	6.8	7.05
371	ZINC85874833	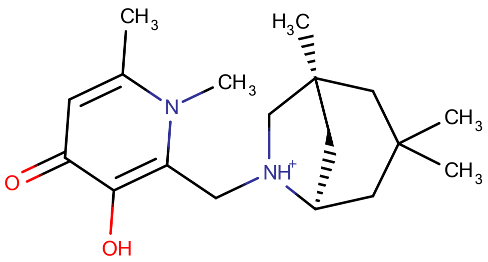	7.1	7	7.05
372	ZINC02341762	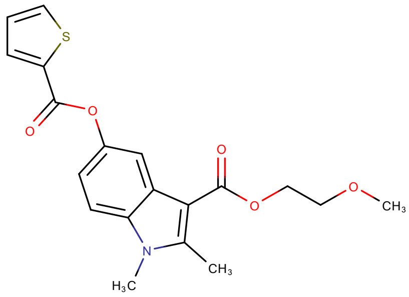	7.1	7	7.05

373	ZINC05646268	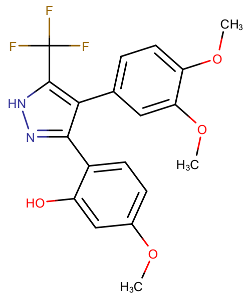	7.1	7	7.05
374	MolPort-002-525-094	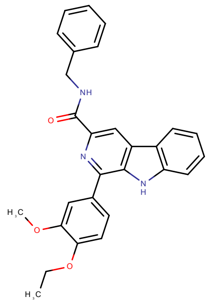	7.1	7	7.05
375	SN00142352	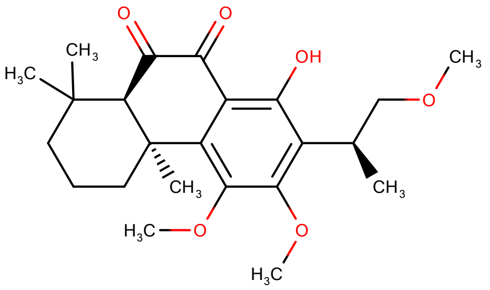	7.1	7	7.05
376	SN00309254	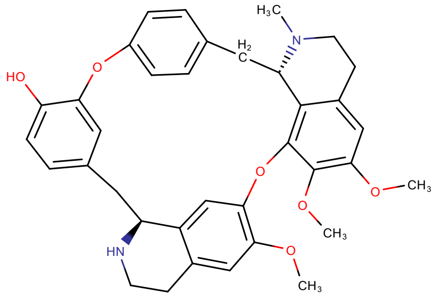	7	7.1	7.05
377	ZINC08991161	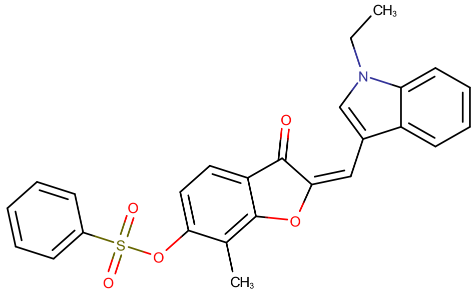	7	7.1	7.05

378	ZINC09129150	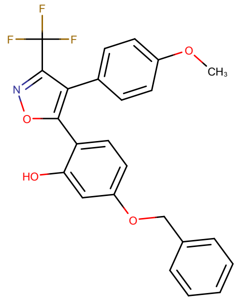	7	7.1	7.05
379	SN00265756	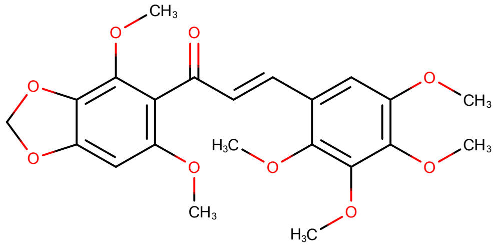	7	7.1	7.05
380	SN00379562	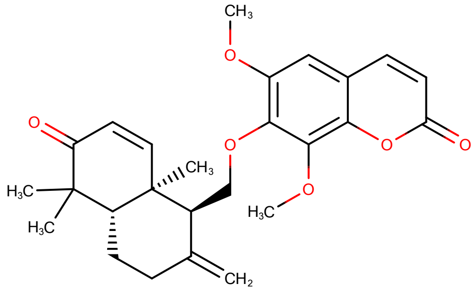	7	7.1	7.05
381	ZINC01463420	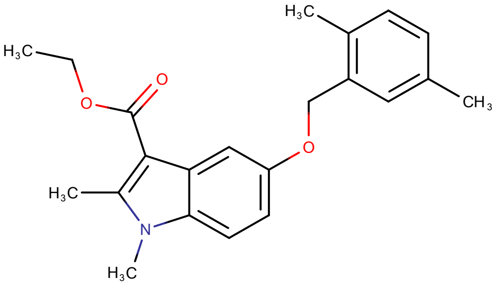	7	7.1	7.05
382	ZINC03846558	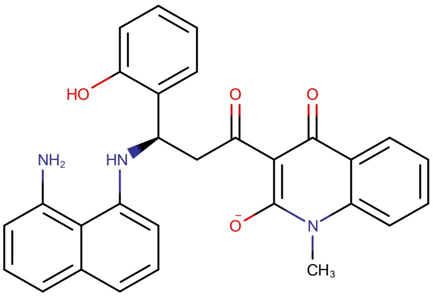	6.8	7.3	7.05

383	ZINC20611343	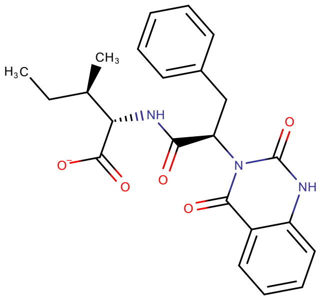	6.8	7.3	7.05
384	6277-14-1	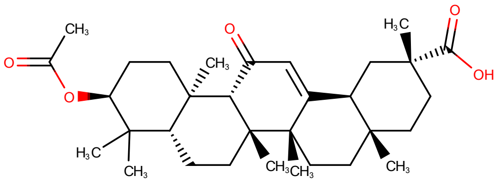	6.8	7.3	7.05
385	MolPort-000-852-534	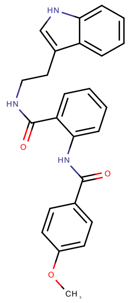	7.2	6.8	7
386	ZINC08790706	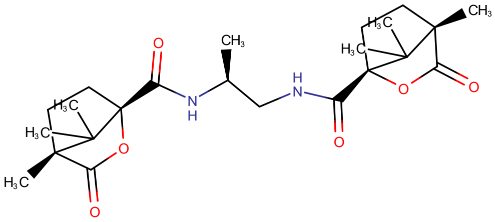	7.1	6.9	7
387	ZINC03999931	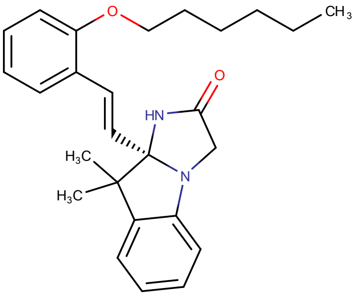	7.1	6.9	7

388	163597-60-2	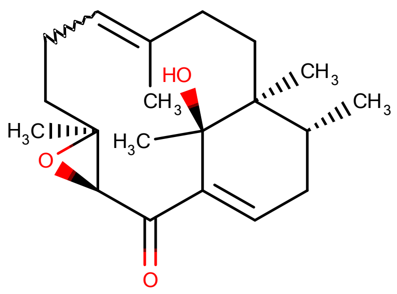	7	7	7
389	MolPort-002-536-237	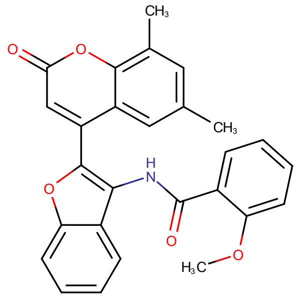	7	7	7
390	SN00083390	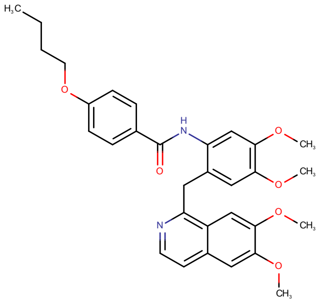	7	7	7
391	23212	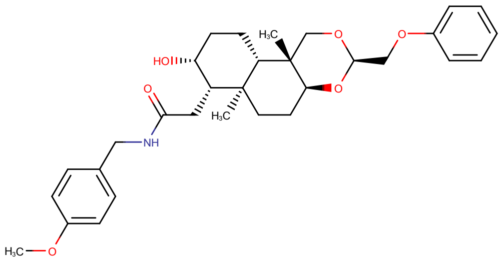	7	7	7
392	ZINC02401244	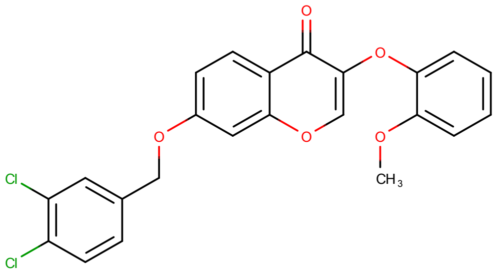	7	7	7

393	ZINC12660555	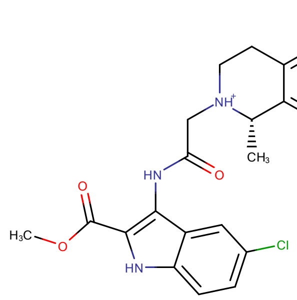	7	7	7
394	MolPort-002-509-346	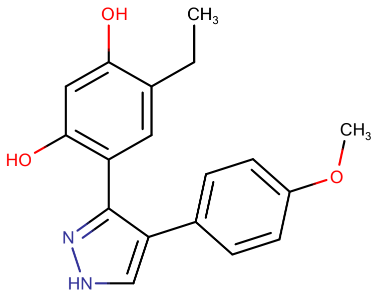	6.9	7.1	7
395	ZINC05646116	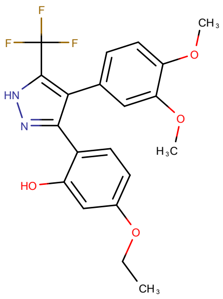	6.9	7.1	7
396	ZINC02094353	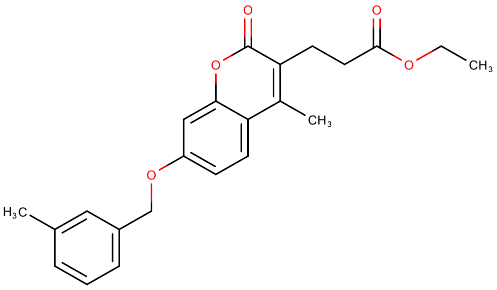	6.9	7.1	7
397	ZINC03846618	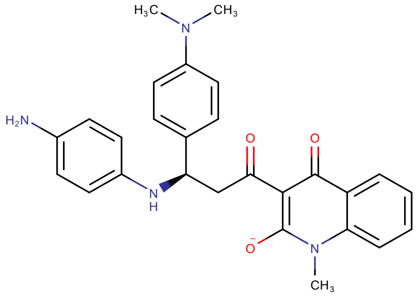	6.9	7.1	7

398	ZINC09129706	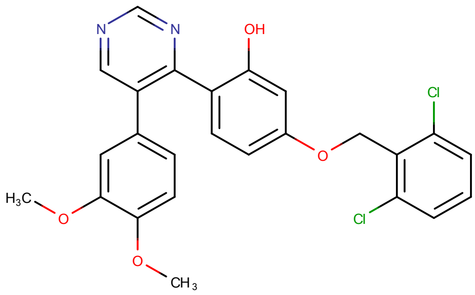	6.9	7.1	7
399	ZINC20113162	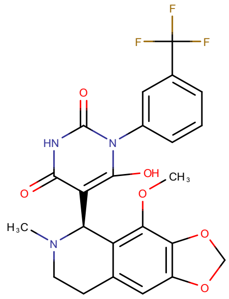	6.9	7.1	7
400	ZINC02119961	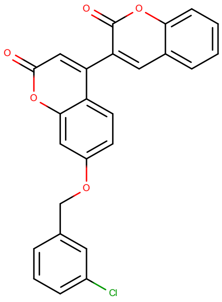	6.9	7.1	7
401	ZINC06662659	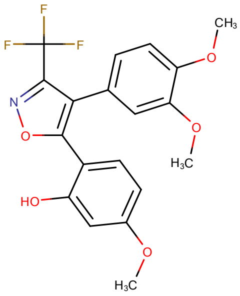	6.8	7.2	7
402	ZINC00129480	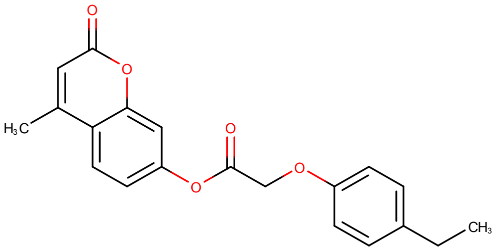	6.8	7.2	7

403	ZINC02283570	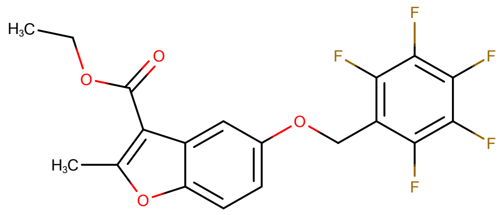	6.8	7.2	7
404	MolPort-039-195-602	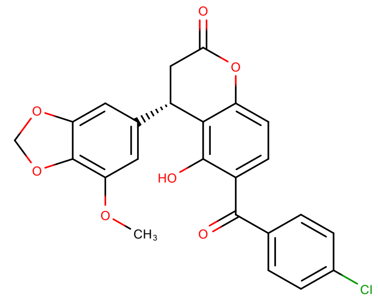	6.8	7.2	7
405	ZINC05484338	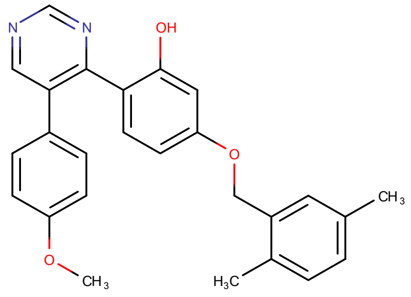	6.8	7.2	7
406	ZINC06669439	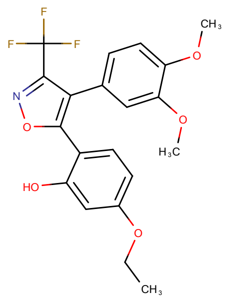	6.7	7.3	7
407	19317	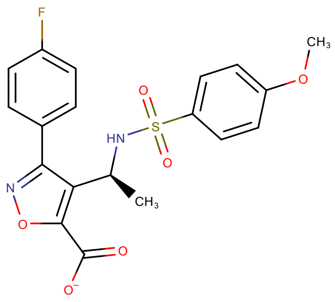	6.7	7.3	7

408	ZINC68591815	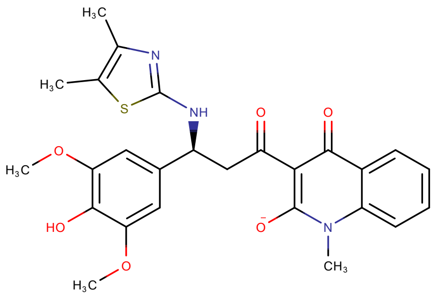	6.7	7.3	7
409	6883	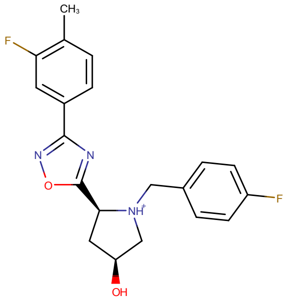	7.2	6.7	6.95
410	ZINC70687765	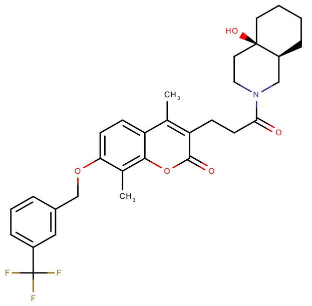	7	6.9	6.95
411	ZINC02112065	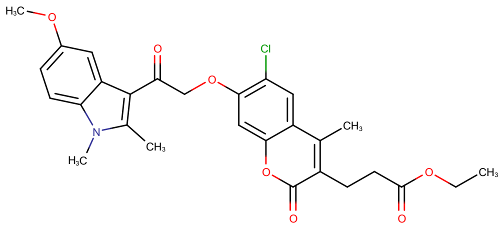	7	6.9	6.95
412	ZINC02111331	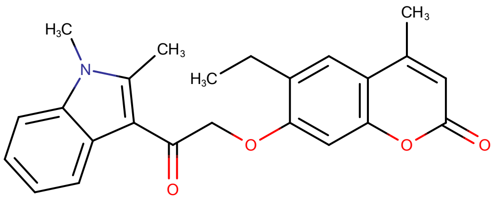	7	6.9	6.95

413	12308	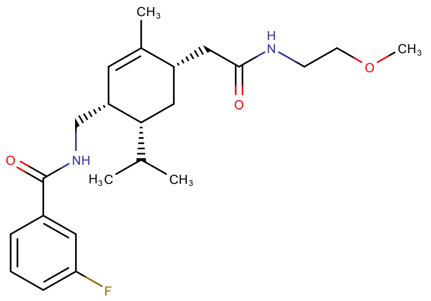	7	6.9	6.95
414	149355-76-0	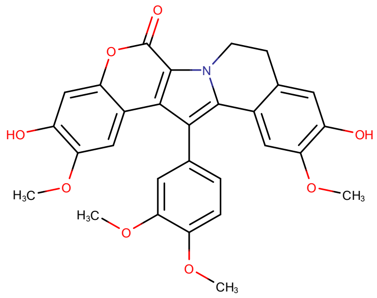	7	6.9	6.95
415	SN00309240	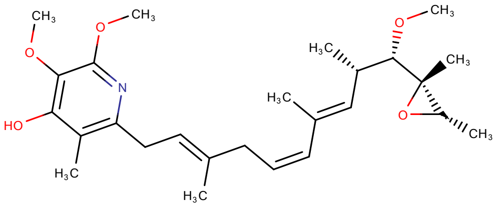	6.9	7	6.95
416	MolPort-001-626-086	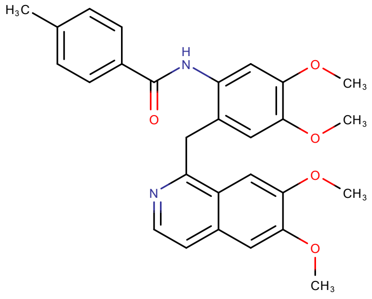	6.9	7	6.95
417	ZINC01321302	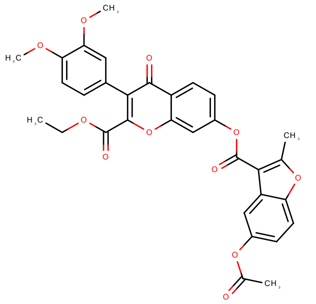	6.9	7	6.95

418	ZINC16364038	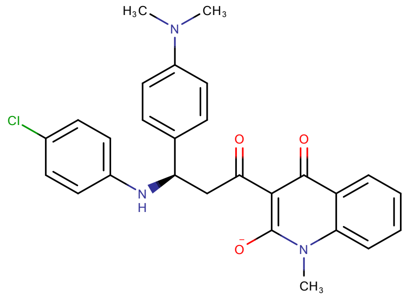	6.9	7	6.95
419	ZINC04278011	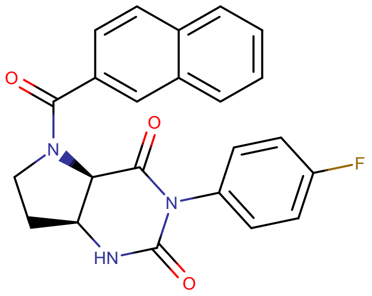	6.9	7	6.95
420	MolPort-044-180-963	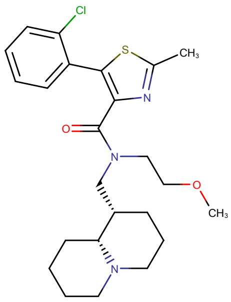	6.9	7	6.95
421	ZINC01280062	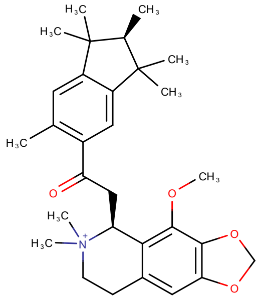	6.9	7	6.95
422	ZINC18166941	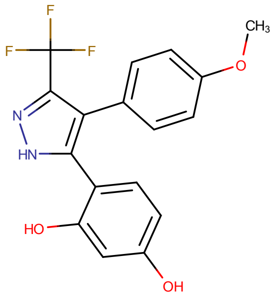	6.7	7.2	6.95

423	SN00353994	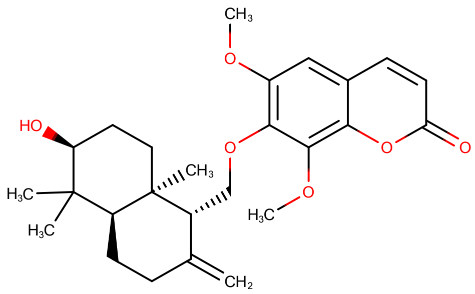	6.7	7.2	6.95
424	15714	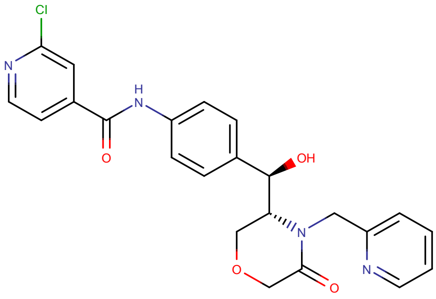	6.7	7.2	6.95
425	MolPort-035-701-757	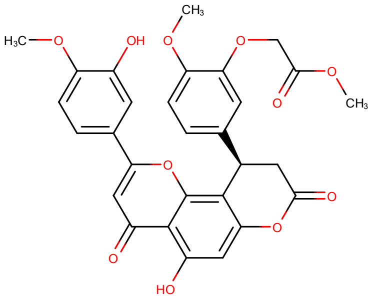	6.7	7.2	6.95
426	ZINC04045543	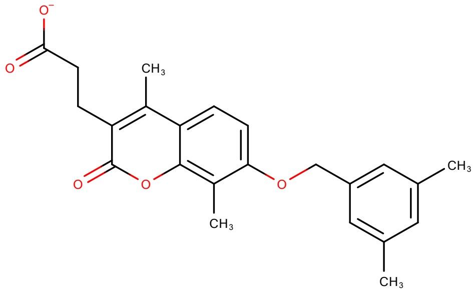	6.5	7.4	6.95
427	MolPort-035-701-683	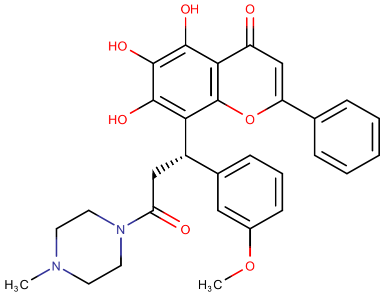	6.8	7.1	6.95

428	ZINC09423424	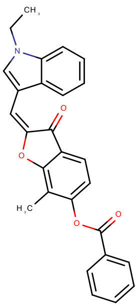	6.8	7.1	6.95
429	SN00073713	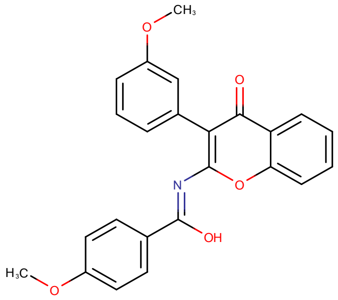	6.8	7.1	6.95
430	ZINC02281027	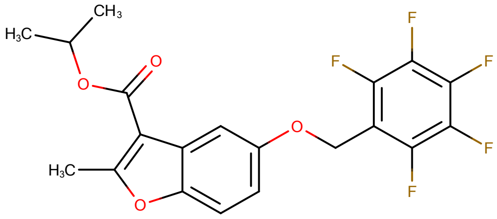	6.8	7.1	6.95
431	ZINC01663395	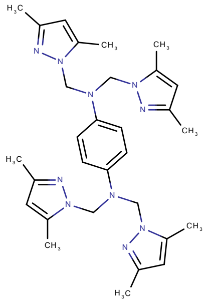	6.8	7.1	6.95
432	ZINC01463853	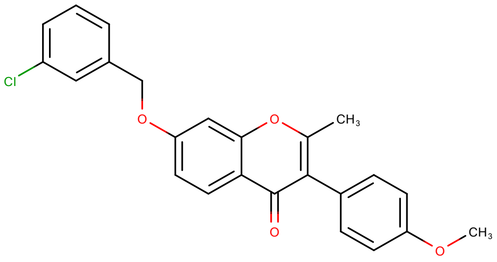	6.8	7.1	6.95

433	SN00173499	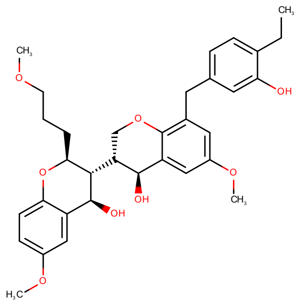	6.8	7.1	6.95
434	MolPort-000-823-233	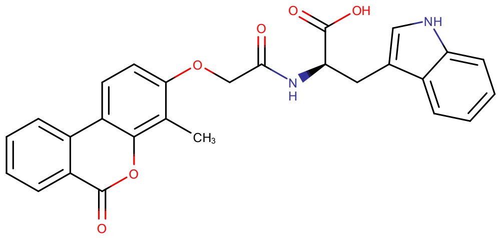	6.6	7.3	6.95
435	ZINC01940964	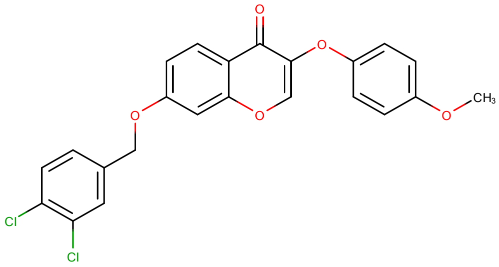	6.6	7.3	6.95
436	MolPort-002-532-272	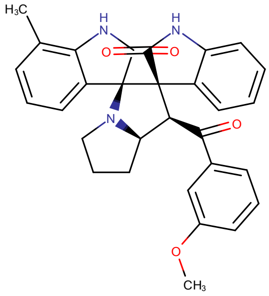	7.1	6.7	6.9
437	MolPort-001-737-843	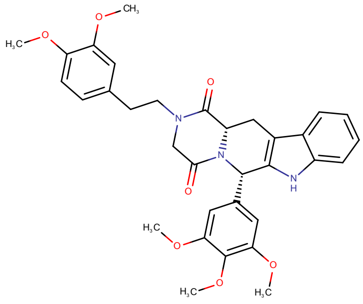	7.1	6.7	6.9

438	15331	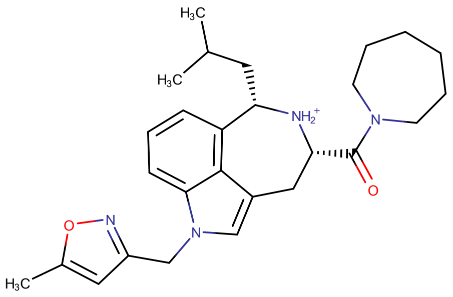	7.1	6.7	6.9
439	MolPort-008-348-482	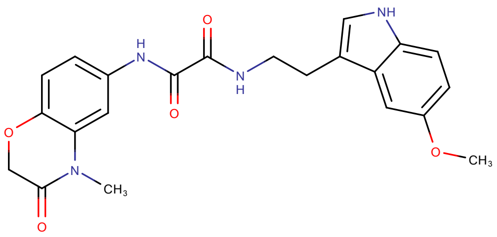	7.1	6.7	6.9
440	13183	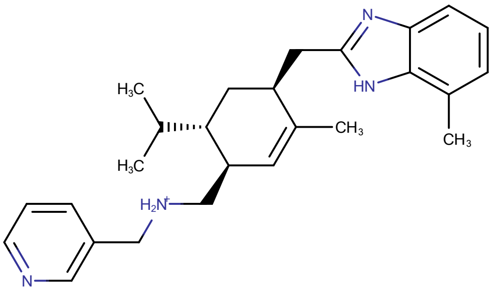	7	6.8	6.9
441	ZINC68572030	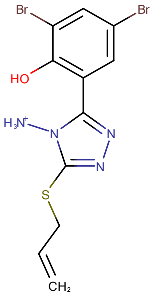	7	6.8	6.9
442	ZINC00945051	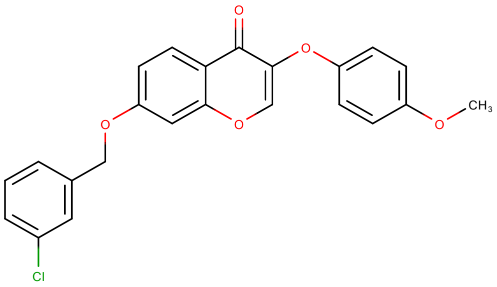	7	6.8	6.9

443	189083-79-2	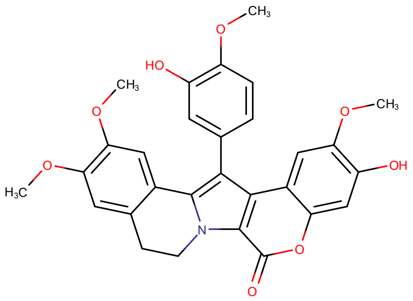	7	6.8	6.9
444	ZINC49543314	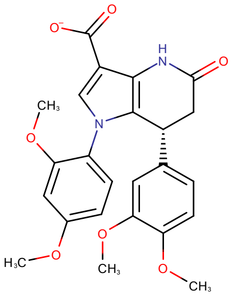	6.9	6.9	6.9
445	ZINC62001460	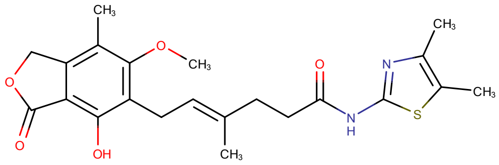	6.9	6.9	6.9
446	MolPort-002-675-581	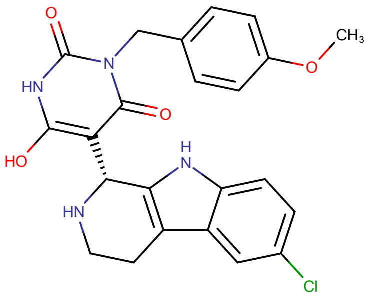	6.9	6.9	6.9
447	ZINC70701314	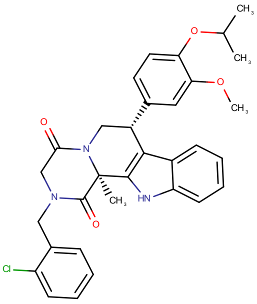	6.9	6.9	6.9

448	ZINC02289379	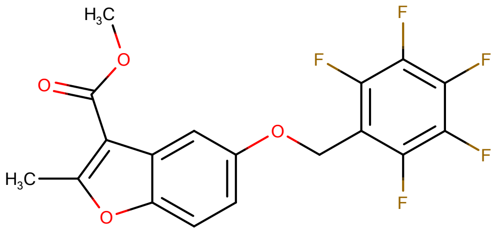	6.9	6.9	6.9
449	ZINC00323509	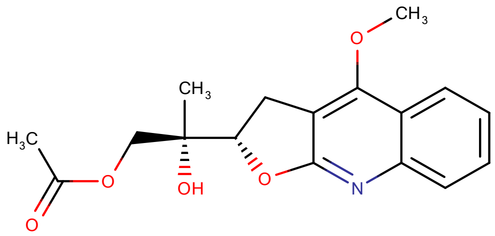	6.9	6.9	6.9
450	ZINC02433600	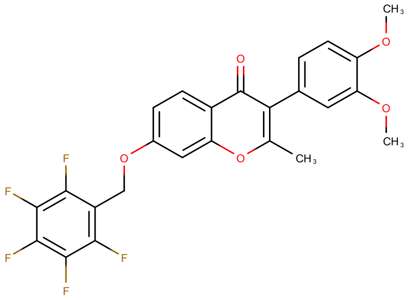	6.9	6.9	6.9
451	115982-19-9	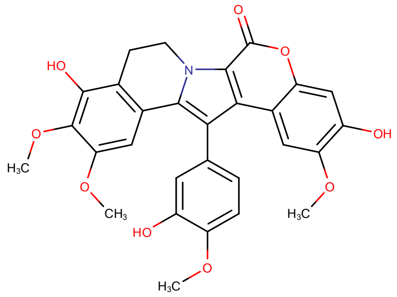	6.9	6.9	6.9
452	ZINC02118774	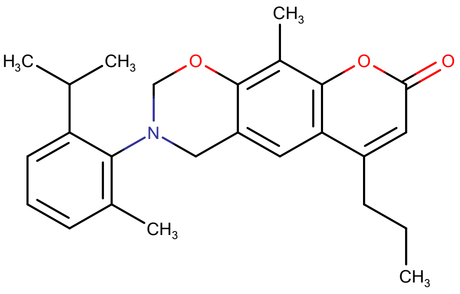	6.9	6.9	6.9

453	131917-09-4	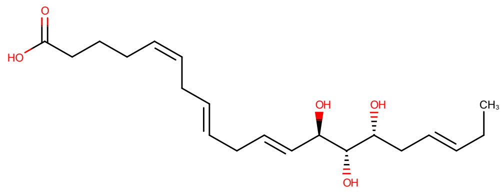	6.8	7	6.9
454	ZINC09409633	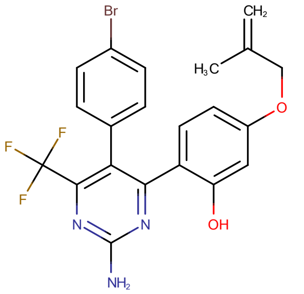	6.8	7	6.9
455	MolPort-002-516-123	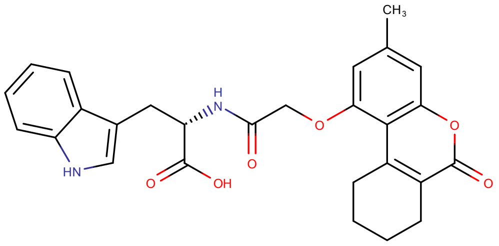	6.8	7	6.9
456	ZINC40312612	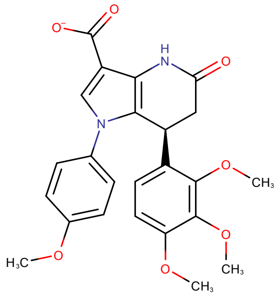	6.7	7.1	6.9
457	ZINC11867078	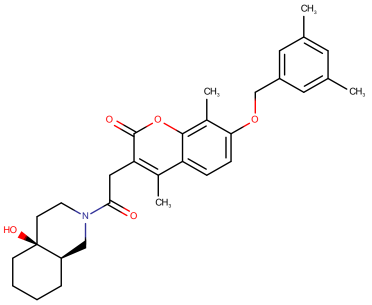	6.7	7.1	6.9

458	ZINC02125245	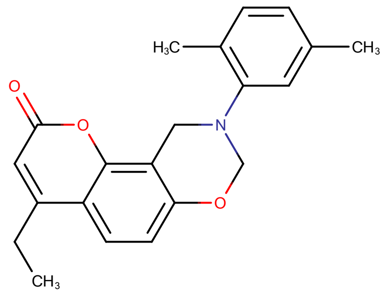	6.7	7.1	6.9
459	MolPort-023-275-183	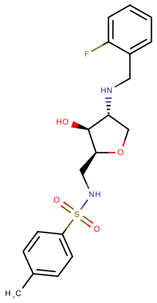	6.6	7.2	6.9
460	ZINC02091635	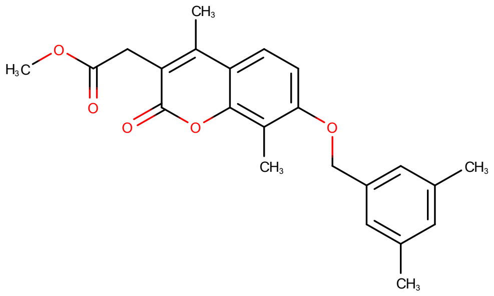	7.1	6.6	6.85
461	ZINC01799114	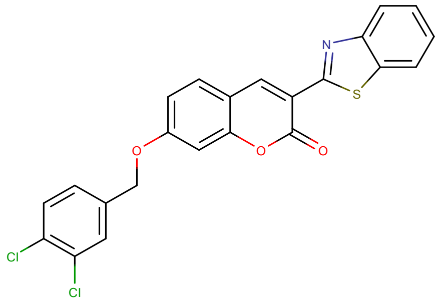	7.1	6.6	6.85
462	MolPort-009-759-056	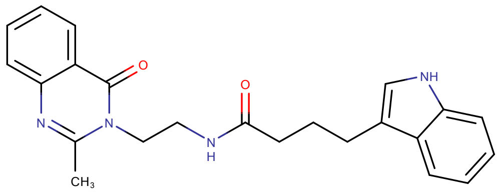	6.9	6.8	6.85

463	ZINC04222564	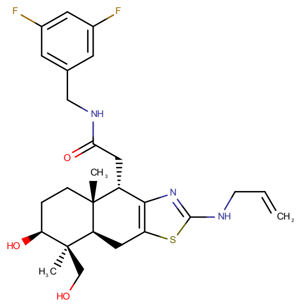	6.9	6.8	6.85
464	MolPort-002-522-484	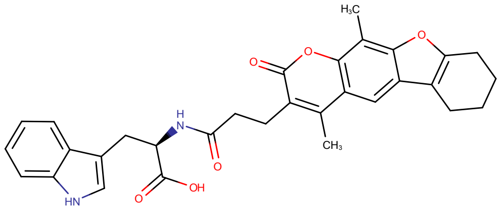	6.9	6.8	6.85
465	SN00267894	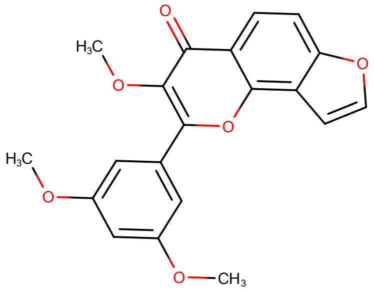	6.9	6.8	6.85
466	ZINC08791835	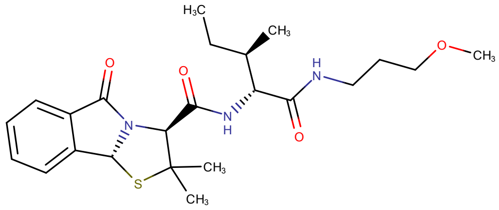	6.9	6.8	6.85
467	ZINC20466689	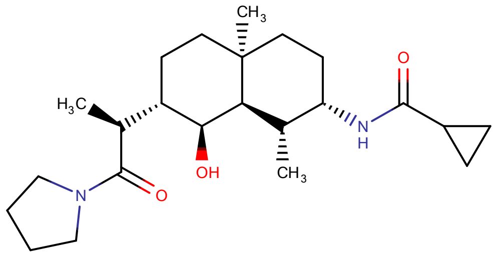	6.9	6.8	6.85

468	SN00073847	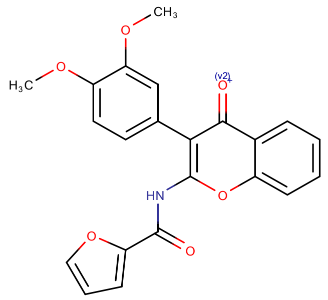	6.9	6.8	6.85
469	ZINC38564389	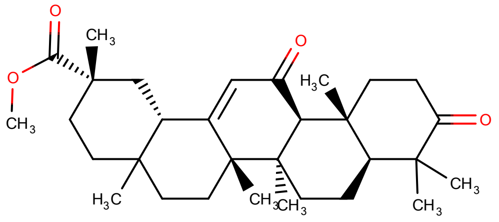	6.8	6.9	6.85
470	7338	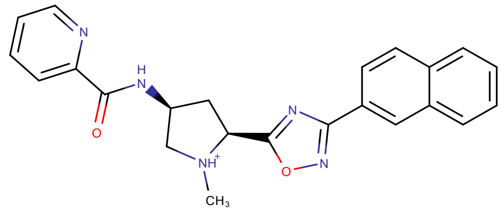	6.8	6.9	6.85
471	ZINC02150013	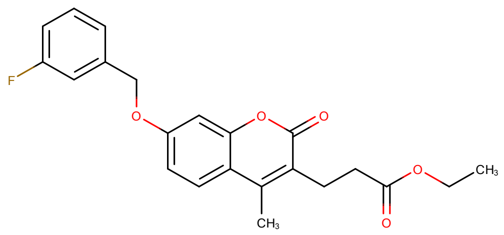	6.8	6.9	6.85
472	ZINC20411593	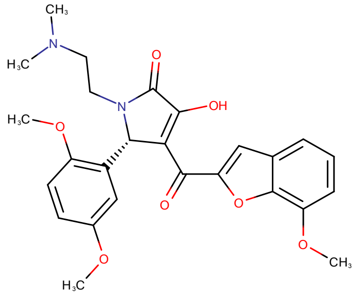	6.8	6.9	6.85

473	MolPort-007-673-275	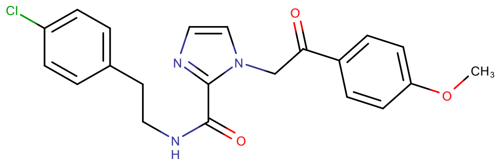	6.8	6.9	6.85
474	ZINC01648045	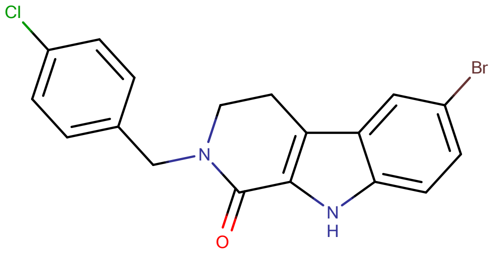	6.8	6.9	6.85
475	ZINC02442084	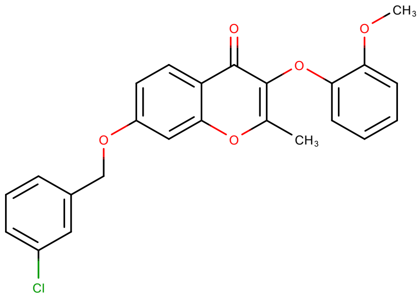	6.8	6.9	6.85
476	6277	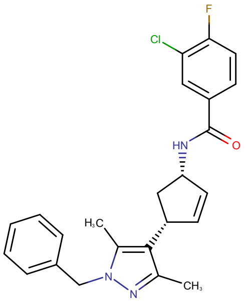	6.7	7	6.85
477	ZINC13729784	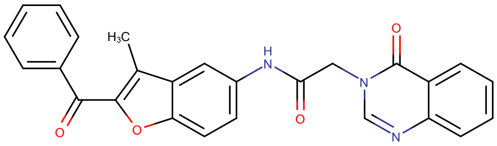	6.7	7	6.85

478	MolPort-028-856-572	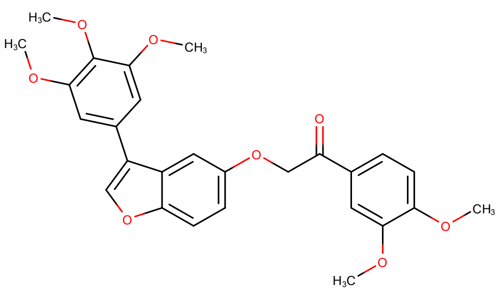	6.7	7	6.85
479	ZINC18046579	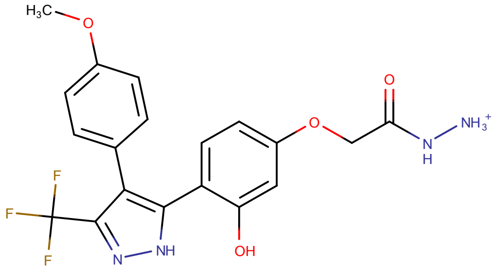	6.7	7	6.85
480	SN00147976	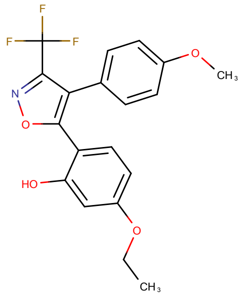	6.7	7	6.85
481	SN00283805	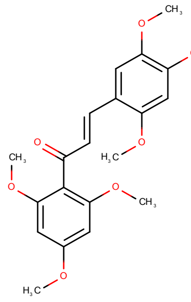	6.7	7	6.85
482	163768-83-0	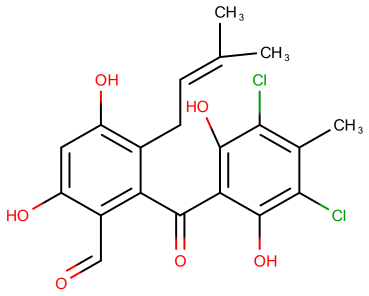	6.7	7	6.85

483	ZINC40309002	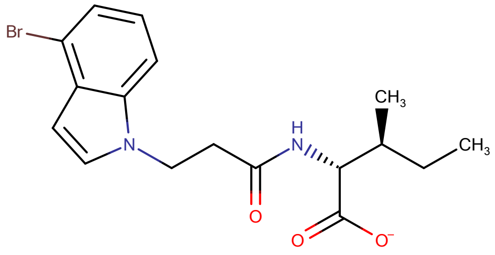	6.7	7	6.85
484	SN00084157	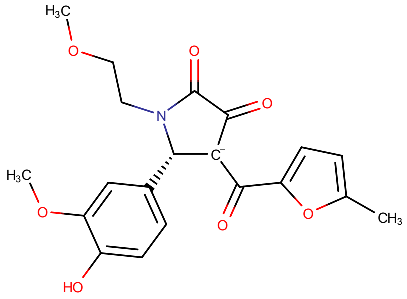	6.7	7	6.85
485	ZINC02092165	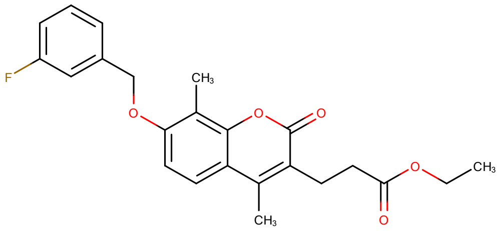	6.6	7.1	6.85
486	ZINC02151660	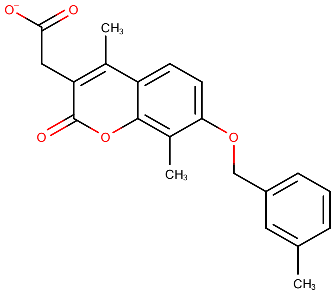	6.5	7.2	6.85
487	ZINC06407719	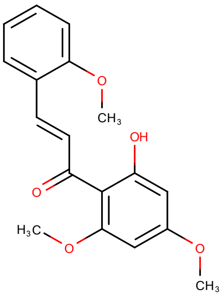	6.7	6.9	6.8

488	ZINC31157673	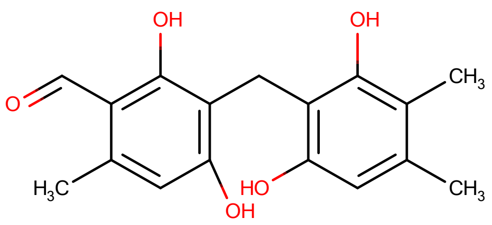	6.7	6.9	6.8
489	MolPort-005-950-245	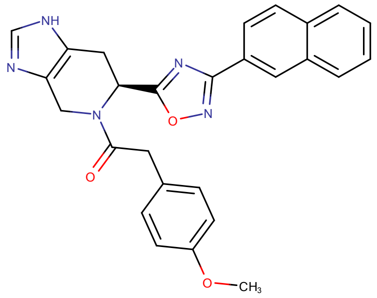	6.7	6.9	6.8
490	SN00145601	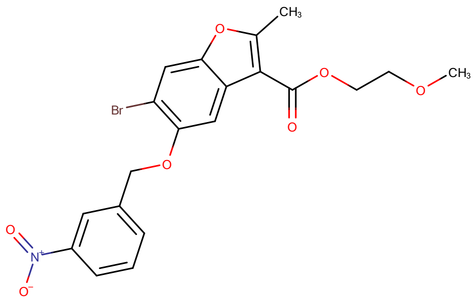	6.7	6.9	6.8
491	ZINC40313355	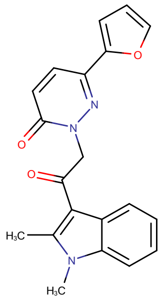	6.7	6.9	6.8
492	ZINC11866426	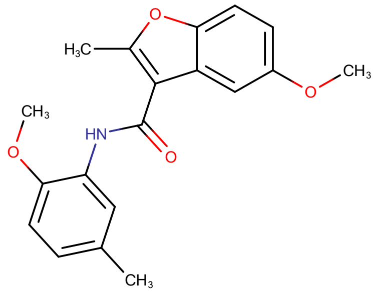	6.7	6.9	6.8

493	MolPort-002-535-447	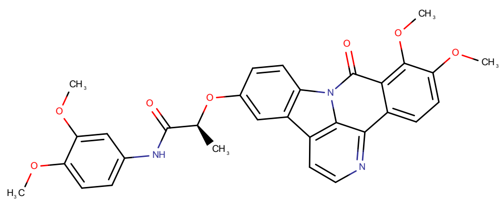	6.7	6.9	6.8
494	ZINC02319078	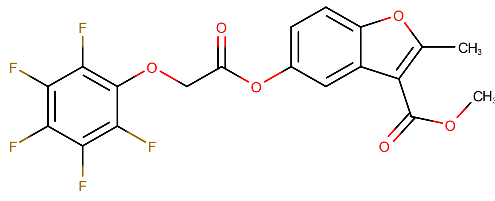	6.7	6.9	6.8
495	ZINC09410228	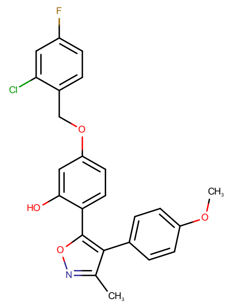	6.7	6.9	6.8
496	ZINC15674316	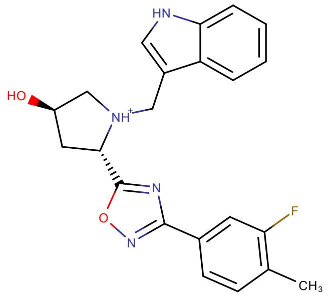	6.8	6.8	6.8
497	ZINC02122012	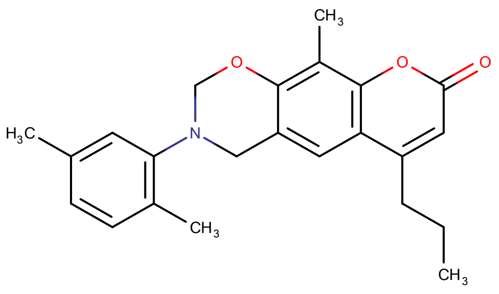	6.8	6.8	6.8

498	ZINC08740407	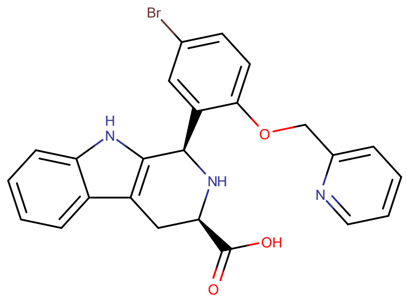	6.8	6.8	6.8
499	SN00147906	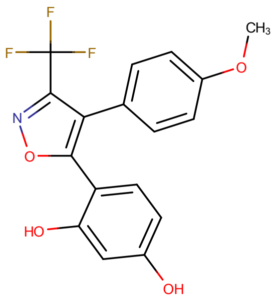	6.6	7	6.8
500	ZINC03846578	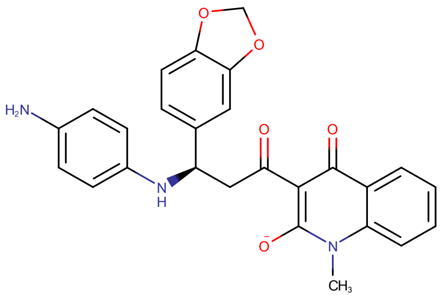	6.6	7	6.8
501	SN00290874	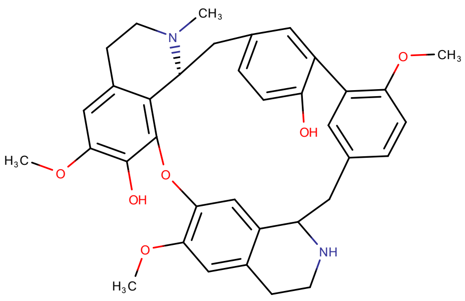	6.6	7	6.8
502	ZINC02126974	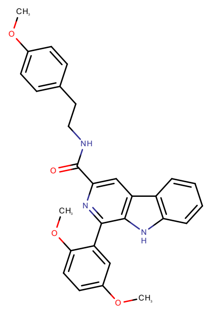	6.6	7	6.8

503	ZINC05389290	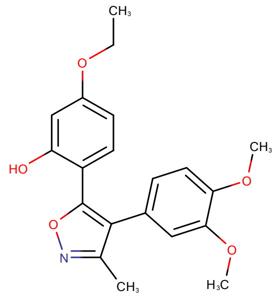	6.5	7.1	6.8
504	MolPort-002-535-319	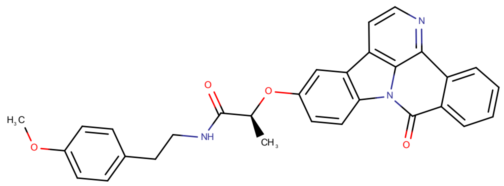	6.5	7.1	6.8
505	ZINC35466154	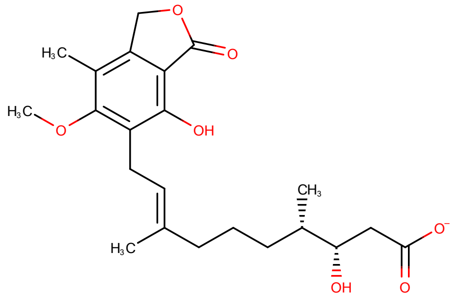	6.5	7.1	6.8
506	ZINC31164529	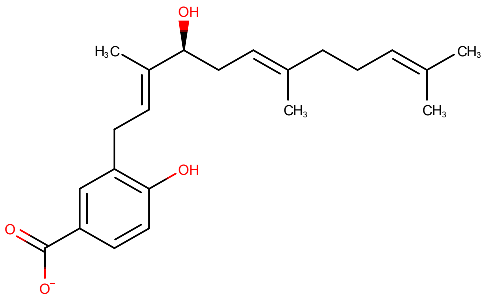	6.5	7.1	6.8
507	MolPort-005-948-910	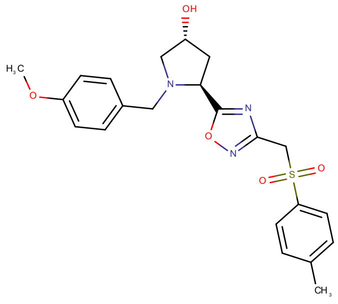	6.5	7.1	6.8

508	ZINC13623573	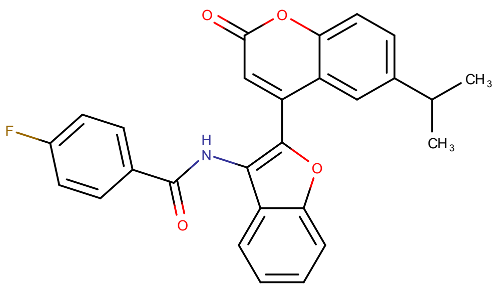	6.9	6.6	6.75
509	MolPort-002-526-623	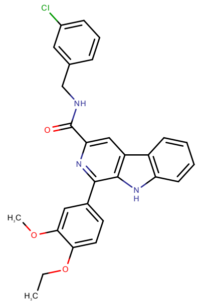	6.9	6.6	6.75
510	ZINC02200212	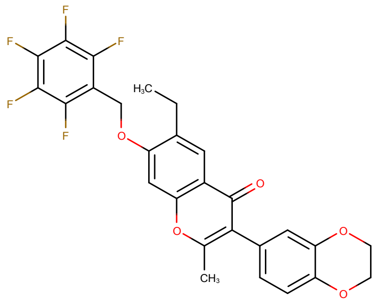	6.9	6.6	6.75
511	ZINC01799112	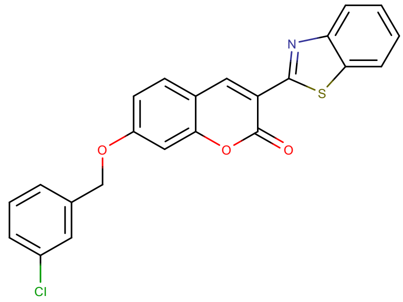	6.9	6.6	6.75
512	ZINC02153244	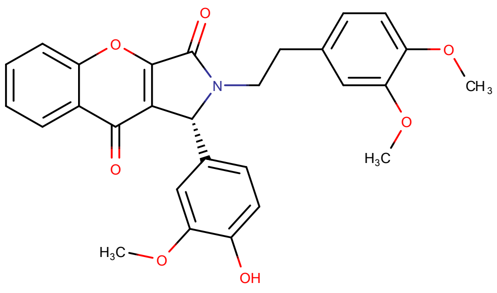	6.8	6.7	6.75

513	SN00017085	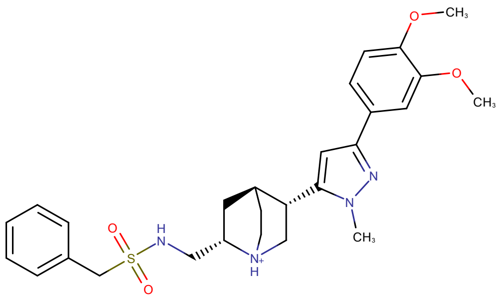	6.8	6.7	6.75
514	MolPort-009-649-082	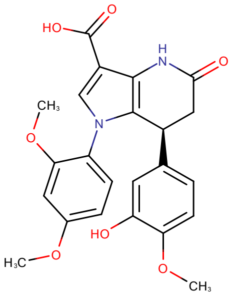	6.8	6.7	6.75
515	MolPort-002-516-371	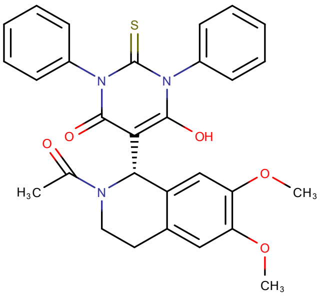	6.7	6.8	6.75
516	ZINC04237030	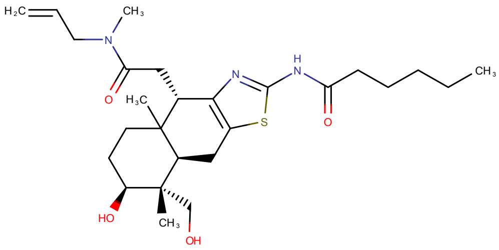	6.7	6.8	6.75
517	ZINC15676415	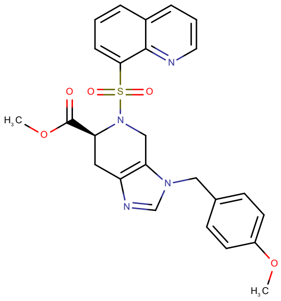	6.7	6.8	6.75

518	ZINC02118808	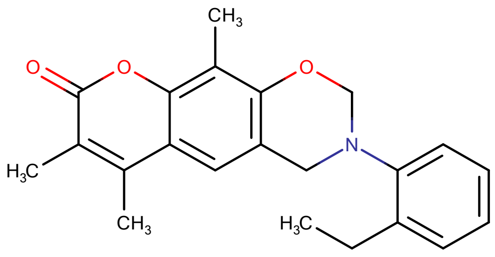	6.7	6.8	6.75
519	ZINC01799111	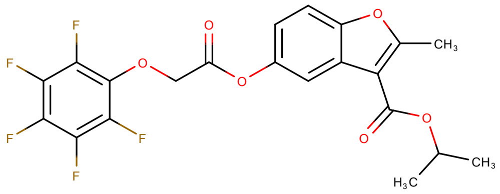	6.7	6.8	6.75
520	SN00332856	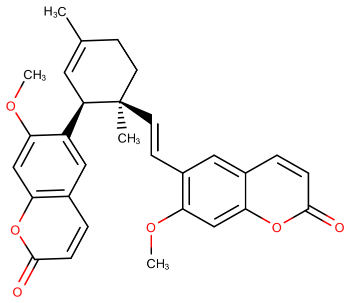	6.7	6.8	6.75
521	ZINC02667598	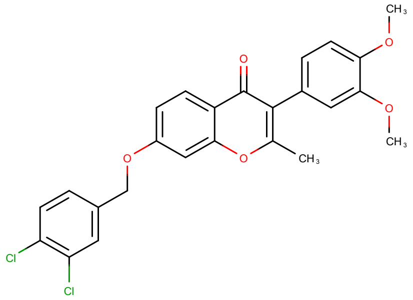	6.7	6.8	6.75
522	ZINC12860017	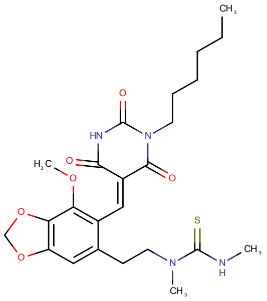	6.7	6.8	6.75

523	20464	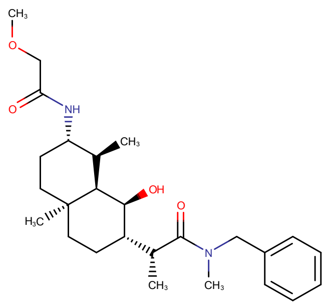	6.6	6.9	6.75
524	ZINC68575016	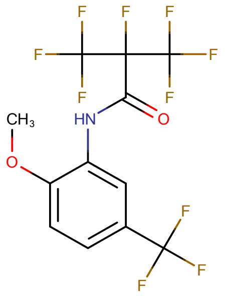	6.6	6.9	6.75
525	ZINC68574667	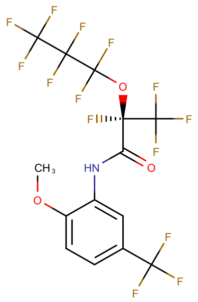	6.6	6.9	6.75
526	SN00148474	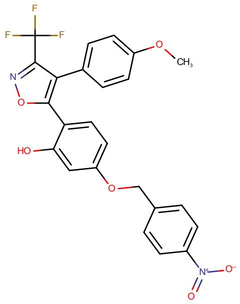	6.6	6.9	6.75
527	ZINC04270713	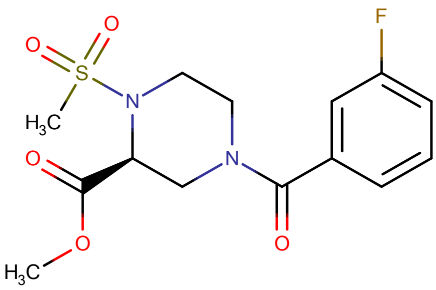	6.6	6.9	6.75

528	ZINC70707164	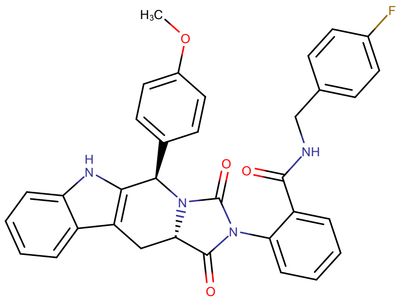	6.6	6.9	6.75
529	ZINC68589892	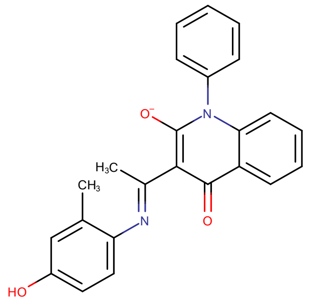	6.6	6.9	6.75
530	MolPort-044-810-768	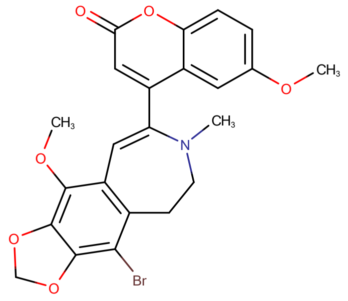	6.6	6.9	6.75
531	19205	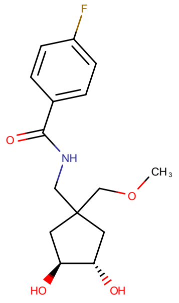	6.5	7	6.75
532	ZINC04222564	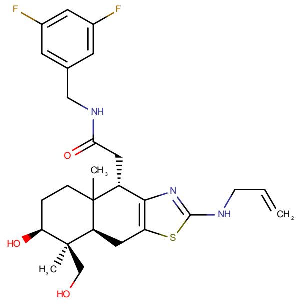	6.5	7	6.75

533	MolPort-035-700-267	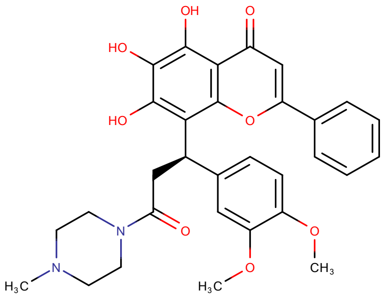	6.5	7	6.75
534	MolPort-002-511-055	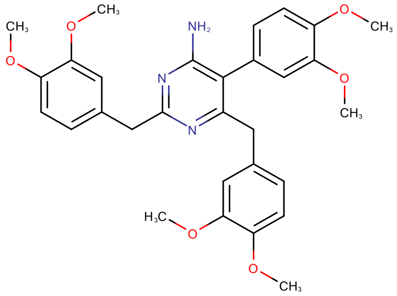	6.5	7	6.75
535	ZINC02135653	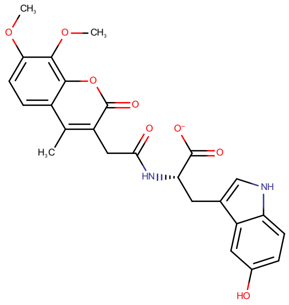	6.5	7	6.75
536	MolPort-000-487-182	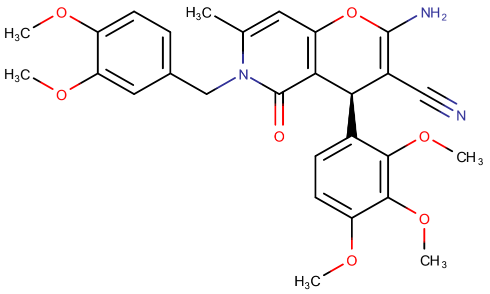	6.4	7.1	6.75
537	MolPort-028-856-091	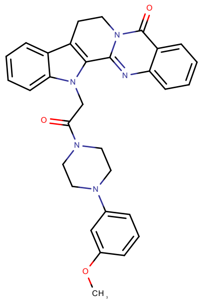	6.9	6.5	6.7

538	ZINC02666313	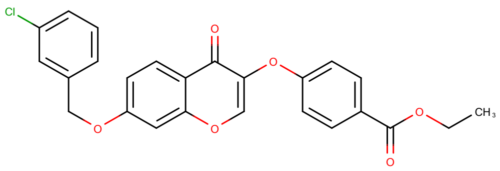	6.9	6.5	6.7
539	MolPort-035-700-717	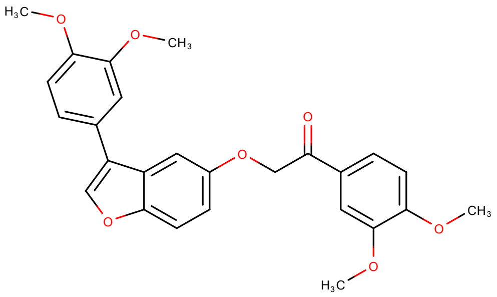	6.7	6.7	6.7
540	MolPort-002-528-533	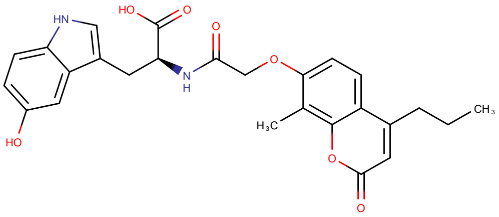	6.7	6.7	6.7
541	MolPort-035-699-980	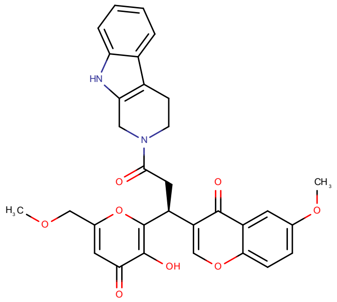	6.7	6.7	6.7
542	MolPort-021-746-036	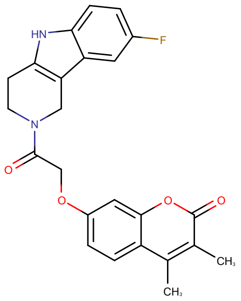	6.7	6.7	6.7

543	SN00075071	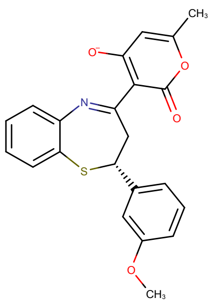	6.5	6.9	6.7
544	ZINC05163437	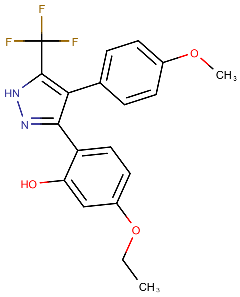	6.5	6.9	6.7
545	MolPort-028-856-192	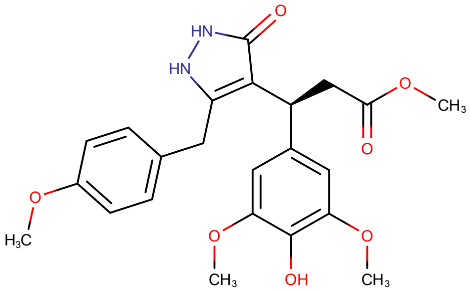	6.5	6.9	6.7
546	18636	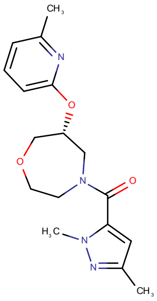	6.5	6.9	6.7
547	MolPort-044-811-170	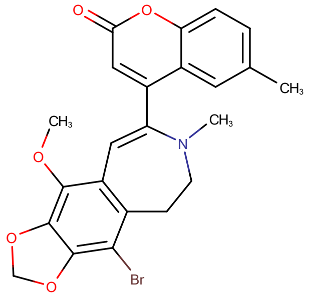	6.5	6.9	6.7

548	200936-84-1	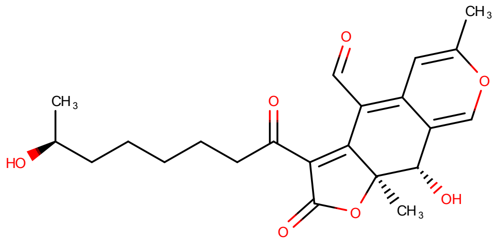	6.4	7	6.7
549	SN00306304	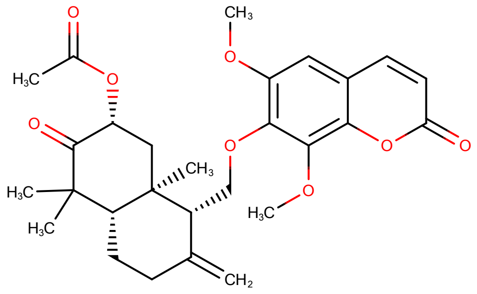	6.4	7	6.7
550	ZINC02122747	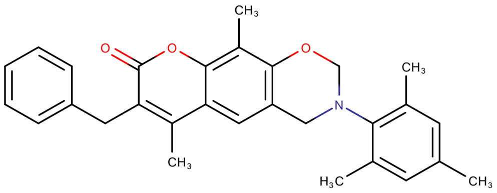	6.4	7	6.7
551	ZINC04267149	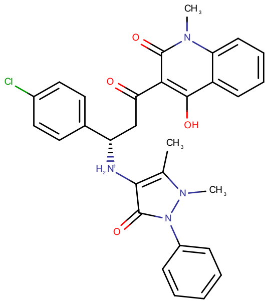	6.4	7	6.7
552	ZINC08918342	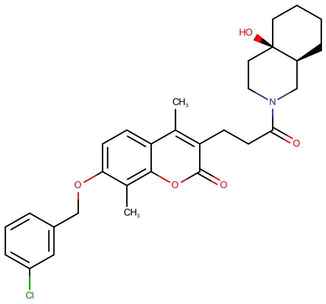	6.8	6.6	6.7

553	ZINC02146856	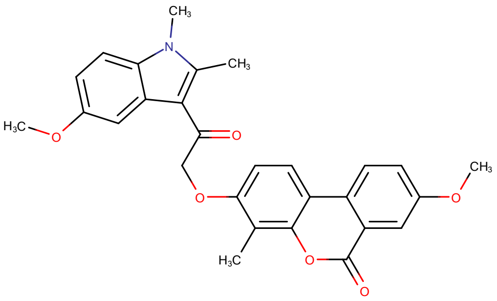	6.8	6.6	6.7
554	MolPort-000-846-216	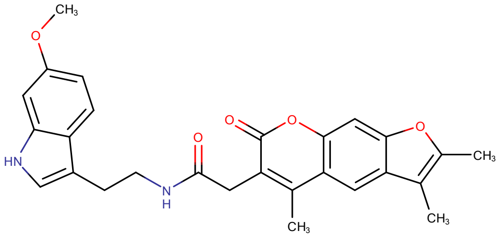	6.8	6.6	6.7
555	ZINC02094262	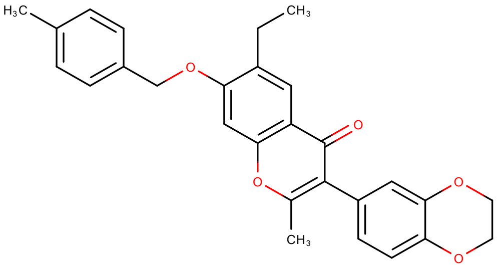	6.8	6.6	6.7
556	MolPort-002-518-378	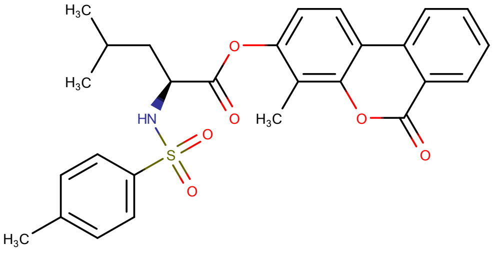	6.6	6.8	6.7
557	ZINC09033642	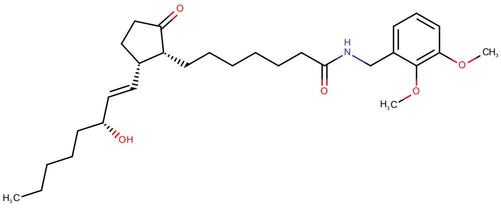	6.6	6.8	6.7

558	MolPort-002-524-112	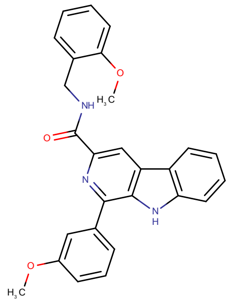	6.6	6.8	6.7
559	ZINC06755168	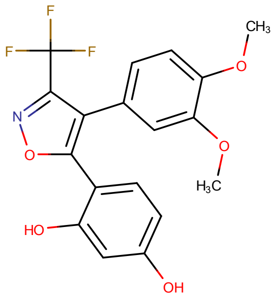	6.6	6.8	6.7
560	ZINC20467205	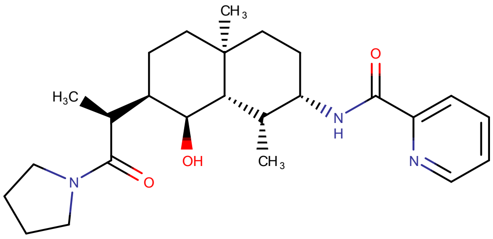	6.6	6.8	6.7
561	ZINC00487450	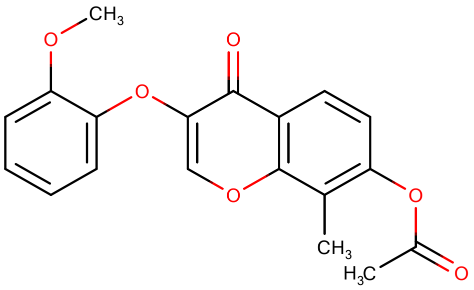	6.6	6.8	6.7
562	MolPort-035-701-927	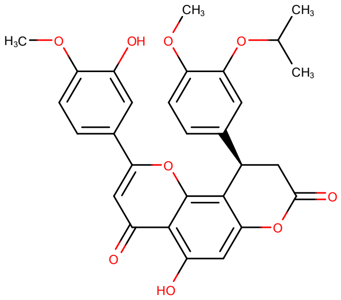	6.6	6.8	6.7

563	ZINC11865237	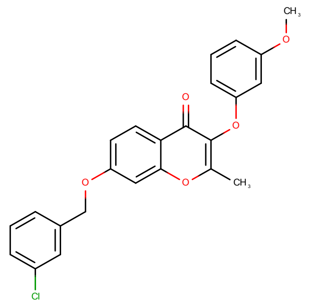	6.6	6.8	6.7
564	ZINC03851657	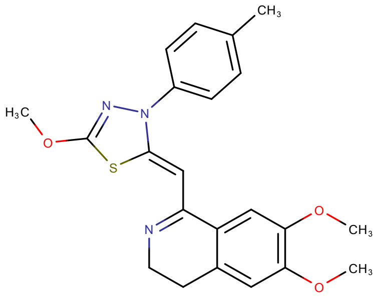	6.6	6.8	6.7
565	149355-75-9	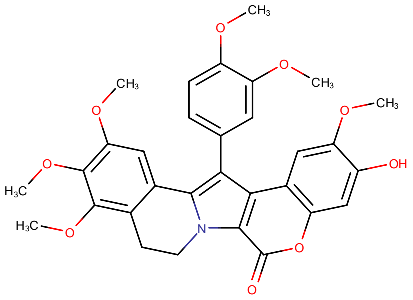	6.6	6.8	6.7
566	ZINC12892682	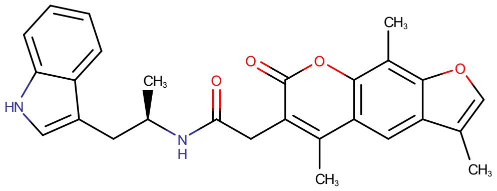	6.9	6.4	6.65
567	ZINC02149868	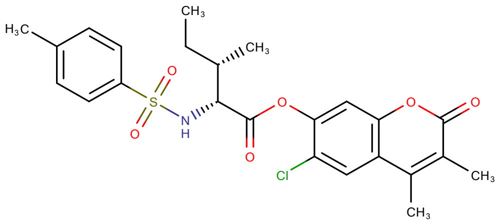	6.8	6.5	6.65

568	ZINC13609475	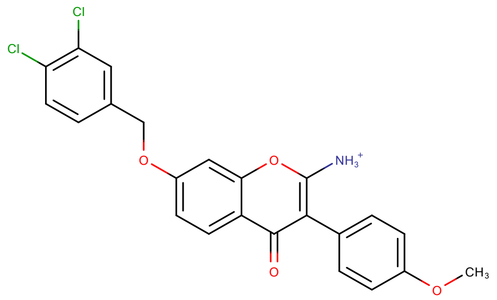	6.8	6.5	6.65
569	SN00241623	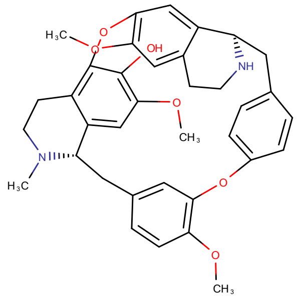	6.7	6.6	6.65
570	SN00101930	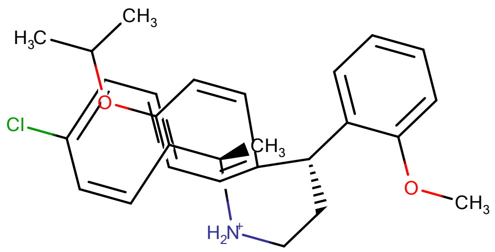	6.7	6.6	6.65
571	MolPort-028-856-171	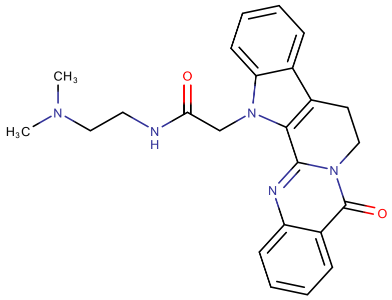	6.7	6.6	6.65
572	ZINC02667544	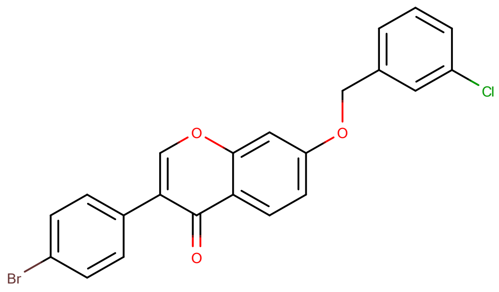	6.7	6.6	6.65

573	ZINC02217015	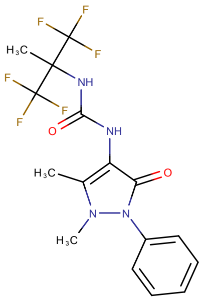	6.7	6.6	6.65
574	ZINC12603403	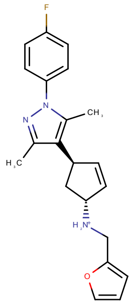	6.6	6.7	6.65
575	ZINC02152082	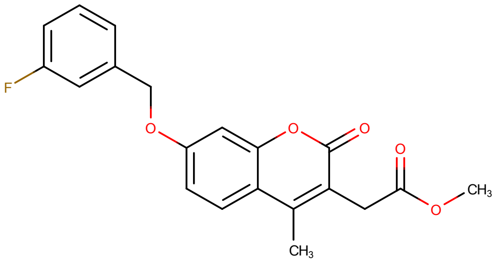	6.6	6.7	6.65
576	ZINC03846418	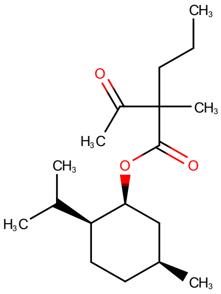	6.6	6.7	6.65
577	MolPort-002-518-659	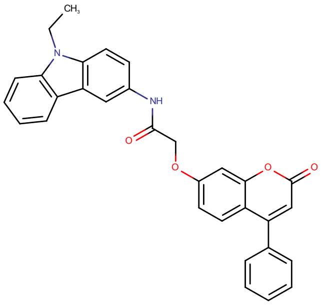	6.6	6.7	6.65

578	143682-17-1	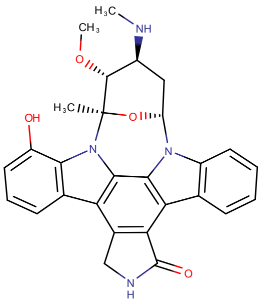	6.5	6.8	6.65
579	ZINC20728784	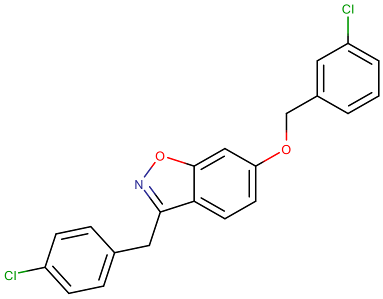	6.5	6.8	6.65
580	ZINC04023868	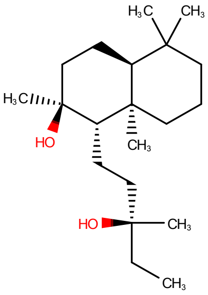	6.5	6.8	6.65
581	SN00052039	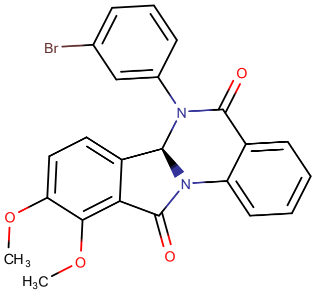	6.5	6.8	6.65
582	MolPort-002-518-297	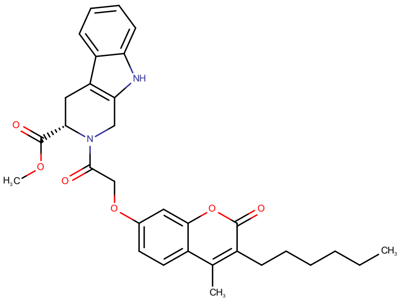	6.5	6.8	6.65

583	121819-68-9	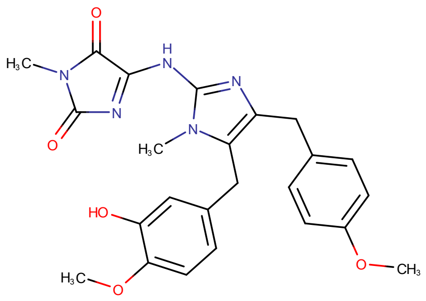	6.4	6.9	6.65
584	SN00074949	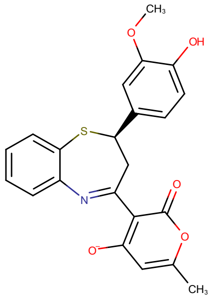	6.4	6.9	6.65
585	SN00049419	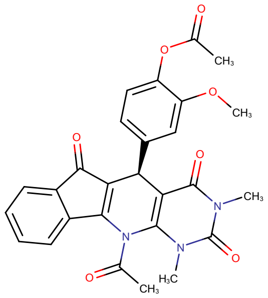	6.4	6.9	6.65
586	MolPort-002-533-130	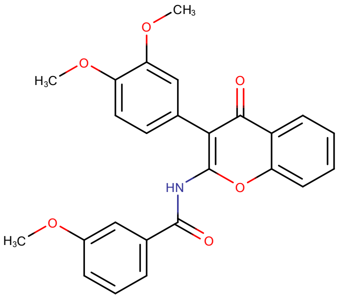	6.3	7	6.65
587	ZINC11867349	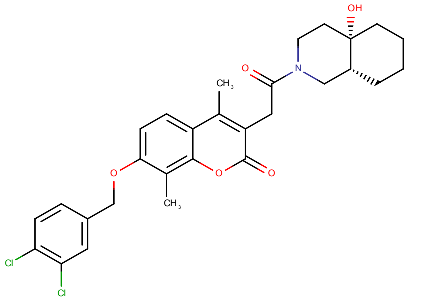	6.7	6.5	6.6

588	ZINC11867170	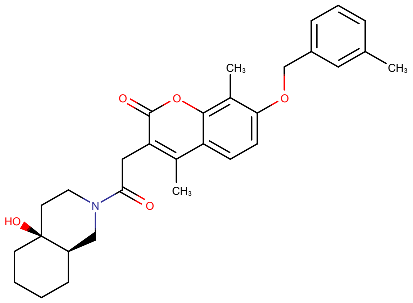	6.6	6.6	6.6
589	MolPort-028-855-419	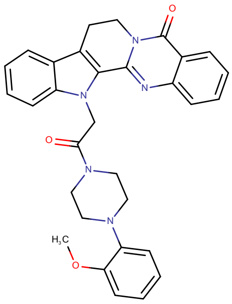	6.6	6.6	6.6
590	ZINC02124948	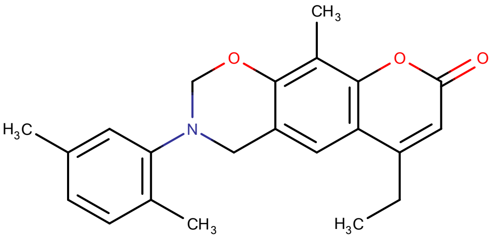	6.6	6.6	6.6
591	ZINC04252491	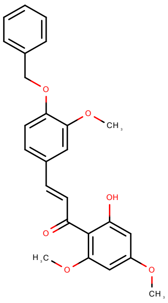	6.6	6.6	6.6
592	MolPort-002-151-076	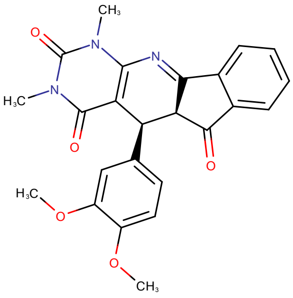	6.5	6.7	6.6

593	ZINC05389044	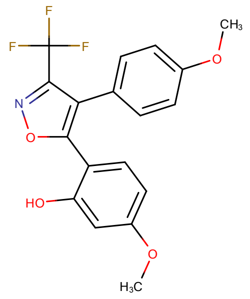	6.5	6.7	6.6
594	ZINC02159827	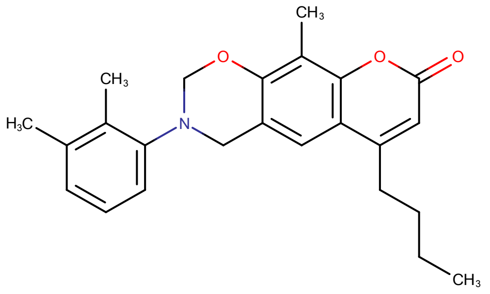	6.5	6.7	6.6
595	ZINC02093059	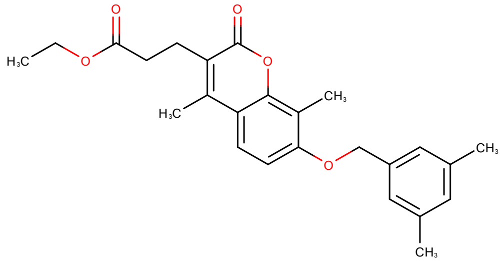	6.5	6.7	6.6
596	ZINC04046749	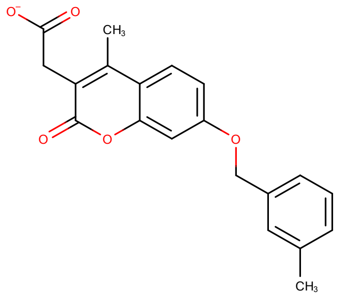	6.5	6.7	6.6
597	SN00050294	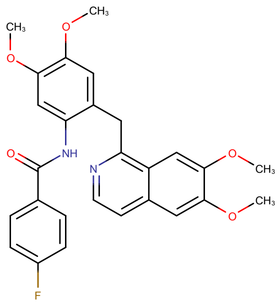	6.5	6.7	6.6

598	SN00348754	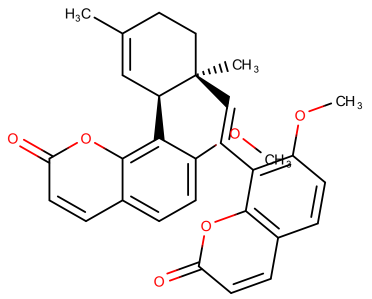	6.5	6.7	6.6
599	ZINC02122508	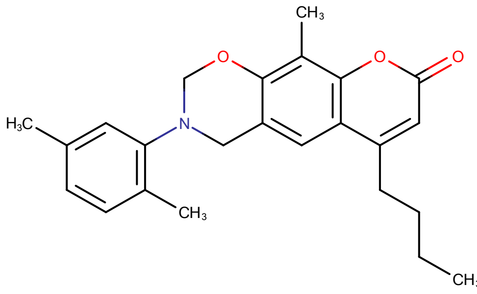	6.5	6.7	6.6
600	SN00151021	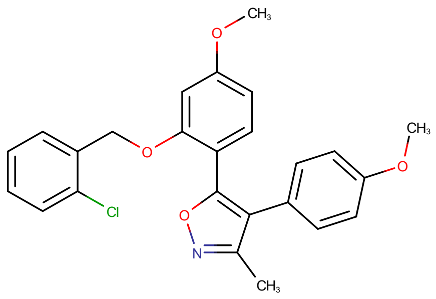	6.5	6.7	6.6
601	ZINC20762168	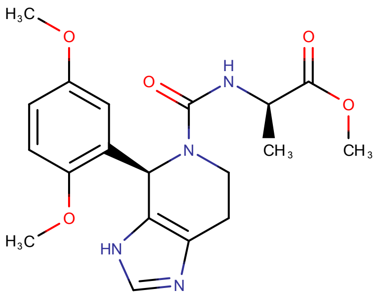	6.4	6.8	6.6
602	ZINC79189259	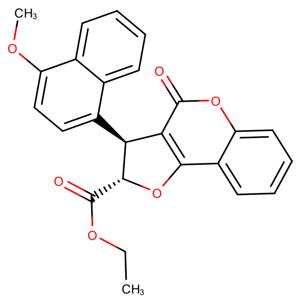	6.4	6.8	6.6

603	ZINC02094763	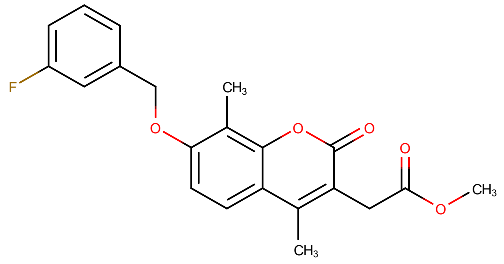	6.4	6.8	6.6
604	ZINC02148915	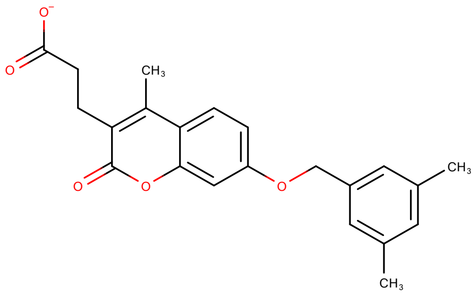	6.4	6.8	6.6
605	ZINC02133746	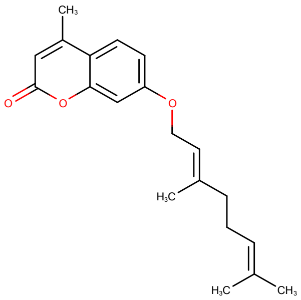	6.4	6.8	6.6
606	ZINC03846610	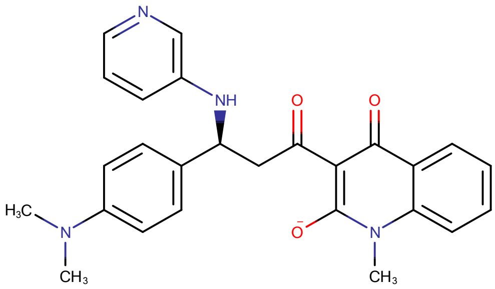	6.4	6.8	6.6
607	MolPort-027-852-780	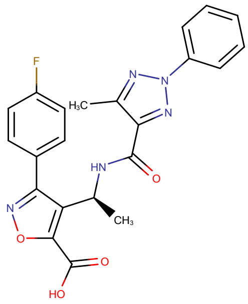	6.4	6.8	6.6

608	ZINC01444304	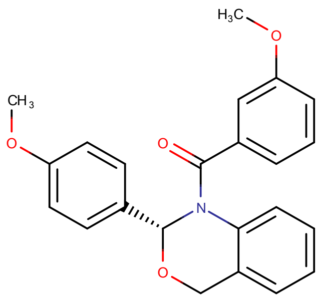	6.4	6.8	6.6
609	ZINC02333037	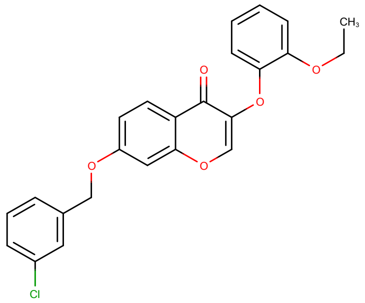	6.4	6.8	6.6
610	ZINC14492226	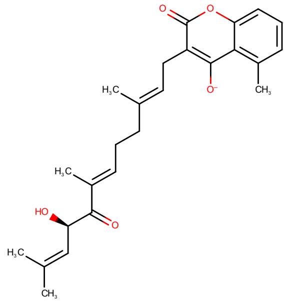	6.3	6.9	6.6
611	ZINC02118316	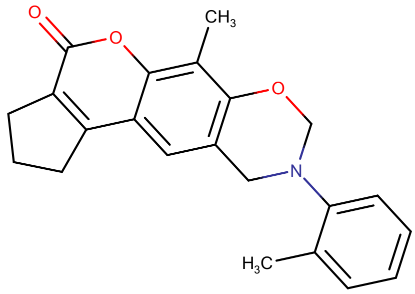	6.3	6.9	6.6
612	MolPort-028-856-432	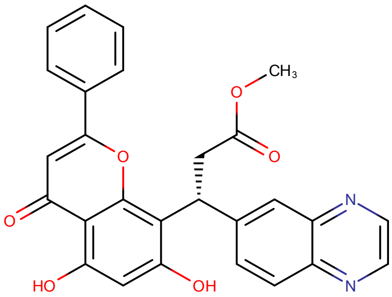	6.3	6.9	6.6

613	ZINC02092152	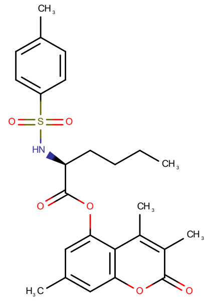	6.3	6.9	6.6
614	SN00084180	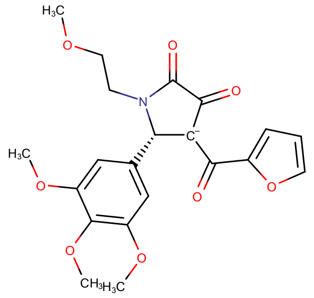	6.3	6.9	6.6
615	SN00077068	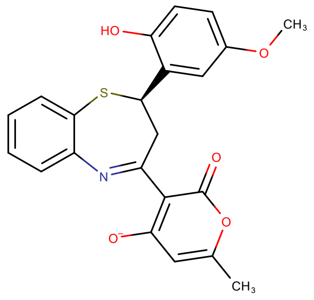	6.2	7	6.6
616	ZINC02277837	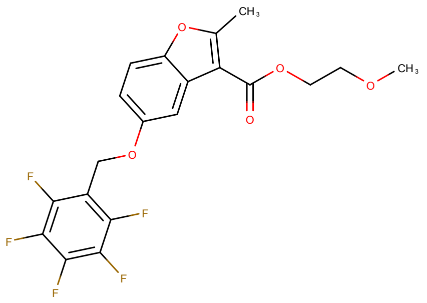	6.2	7	6.6
617	ZINC68591828	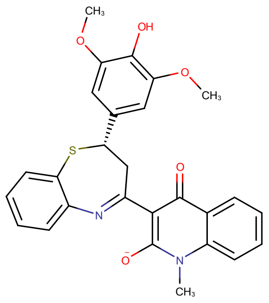	6.2	7	6.6

618	ZINC38143767	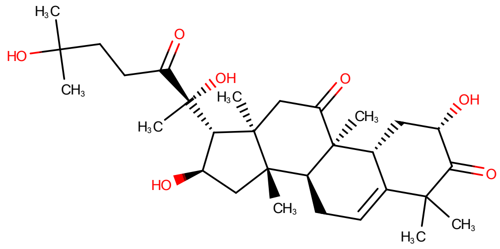	6.7	6.4	6.55
619	SN00048103	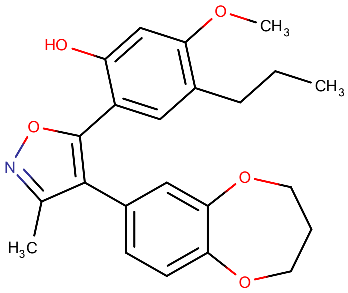	6.4	6.7	6.55
620	20512	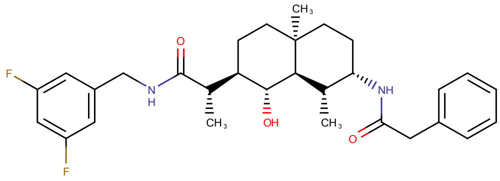	6.4	6.7	6.55
621	ZINC02094396	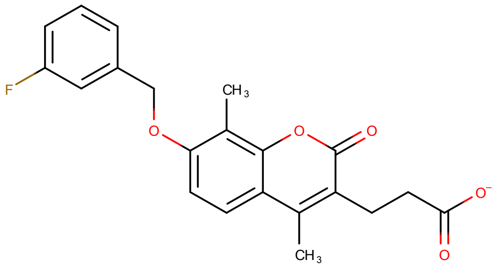	6.4	6.7	6.55
622	ZINC02148668	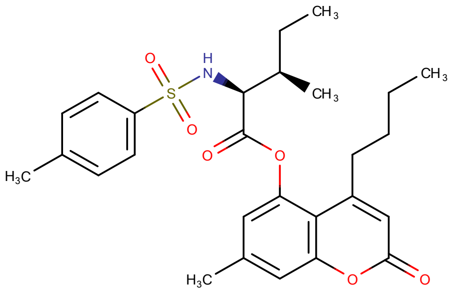	6.4	6.7	6.55

623	ZINC02147761	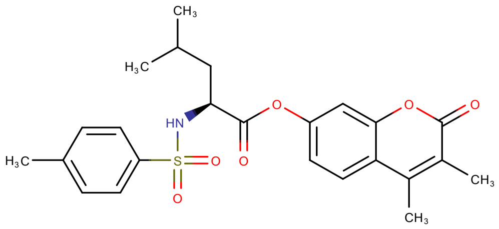	6.4	6.7	6.55
624	ZINC02109980	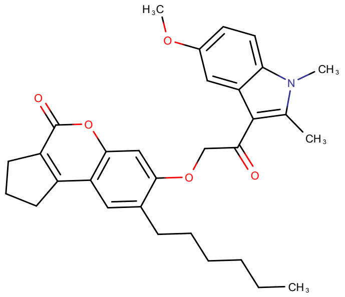	6.4	6.7	6.55
625	ZINC02666276	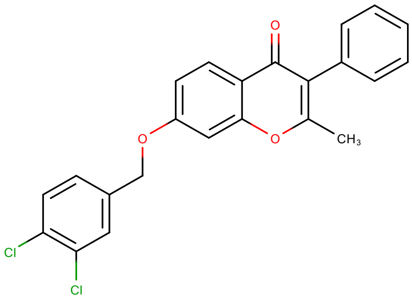	6.4	6.7	6.55
626	10245-81-5	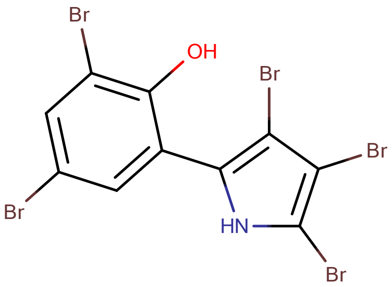	6.4	6.7	6.55
627	SN00267570	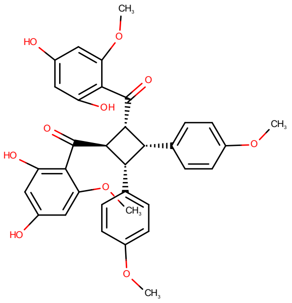	6.4	6.7	6.55

628	ZINC12530252	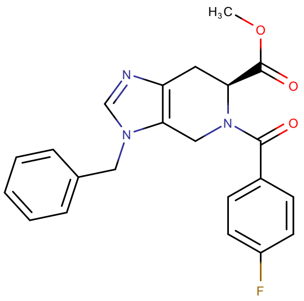	6.6	6.5	6.55
629	MolPort-002-527-363	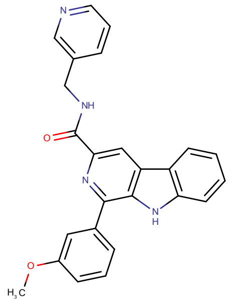	6.6	6.5	6.55
630	115982-21-3	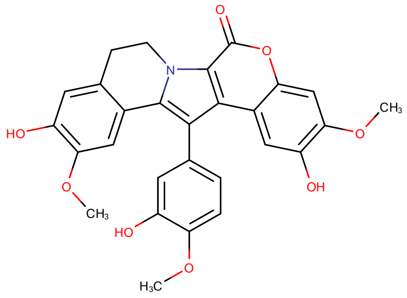	6.6	6.5	6.55
631	115982-20-2	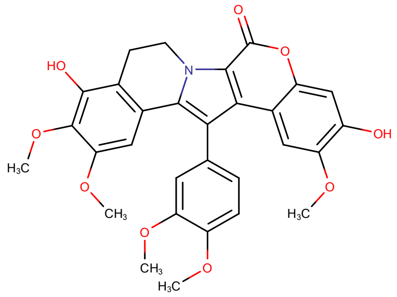	6.6	6.5	6.55
632	MolPort-008-347-150	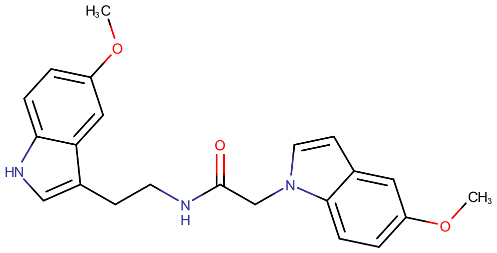	6.5	6.6	6.55

633	ZINC48057066	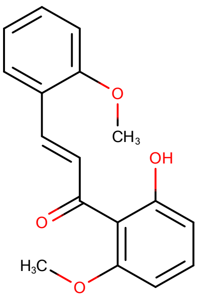	6.5	6.6	6.55
634	7138	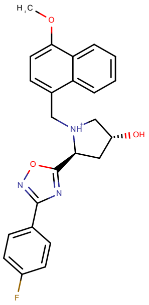	6.5	6.6	6.55
635	12504	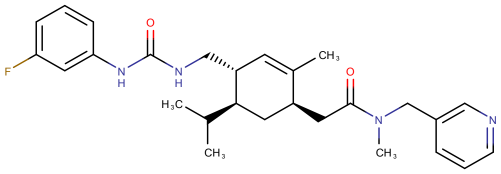	6.5	6.6	6.55
636	ZINC20466733	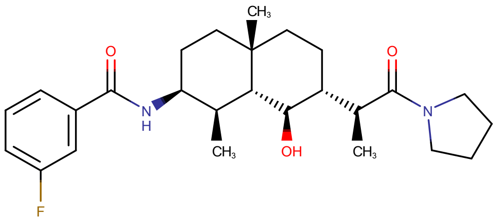	6.5	6.6	6.55
637	MolPort-002-525-564	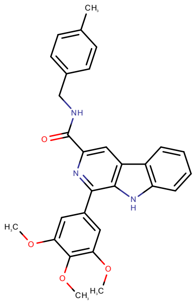	6.5	6.6	6.55

638	ZINC02148701	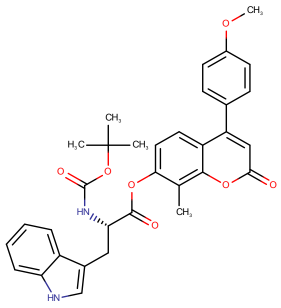	6.5	6.6	6.55
639	MolPort-001-683-717	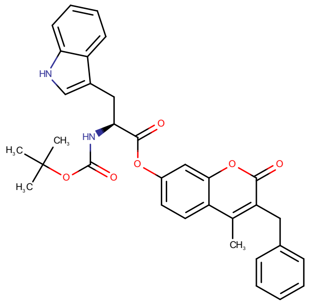	6.3	6.8	6.55
640	MolPort-009-649-155	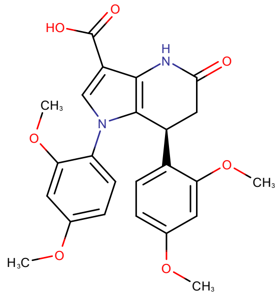	6.3	6.8	6.55
641	ZINC02124986	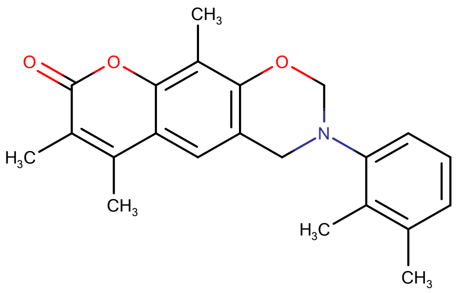	6.3	6.8	6.55
642	MolPort-002-520-410	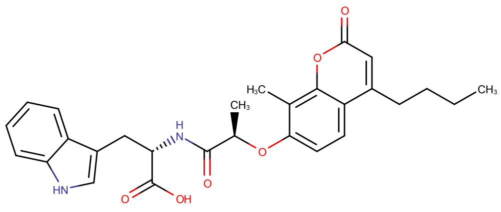	6.1	7	6.55

643	ZINC02423589	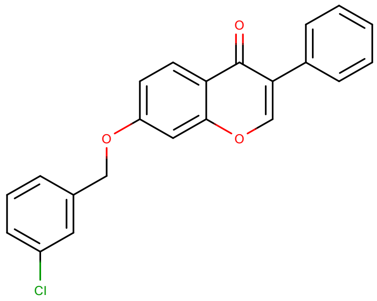	6.6	6.4	6.5
644	ZINC20467109	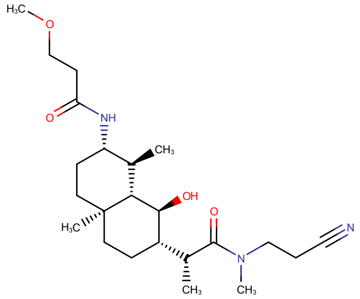	6.5	6.5	6.5
645	MolPort-042-647-992	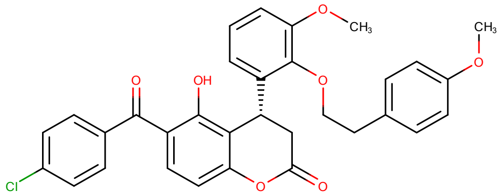	6.5	6.5	6.5
646	ZINC02424621	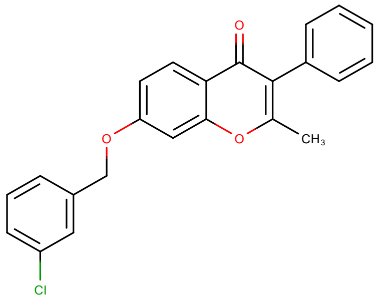	6.5	6.5	6.5
647	ZINC11865561	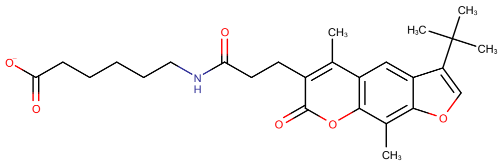	6.4	6.6	6.5

648	ZINC09233757	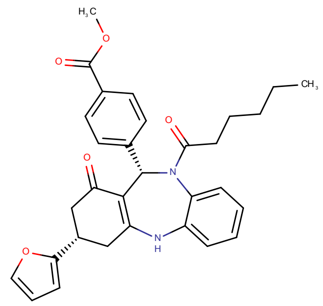	6.4	6.6	6.5
649	ZINC02091929	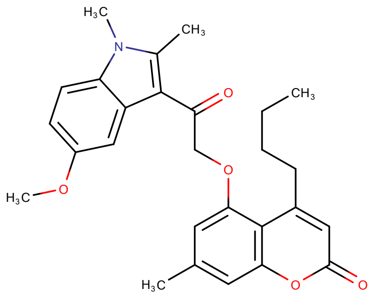	6.4	6.6	6.5
650	ZINC13727702	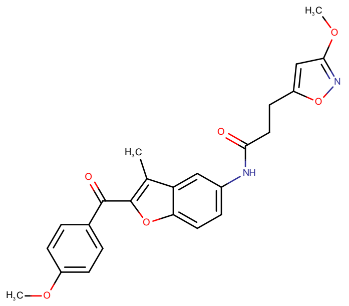	6.3	6.7	6.5
651	MolPort-002-518-545	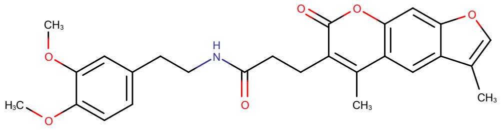	6.3	6.7	6.5
652	SN00396633	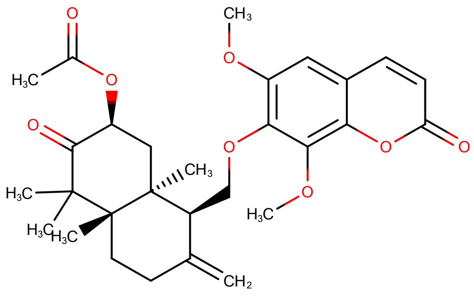	6.3	6.7	6.5

653	ZINC02148113	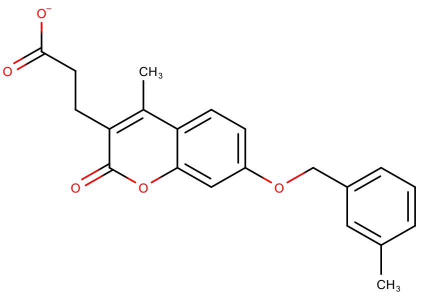	6.3	6.7	6.5
654	ZINC02116736	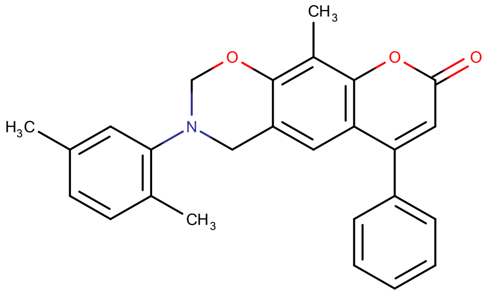	6.3	6.7	6.5
655	ZINC68569482	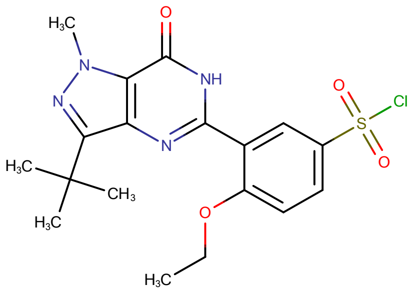	6.3	6.7	6.5
656	SN00077982	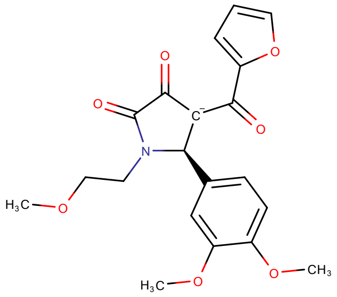	6.2	6.8	6.5
657	MolPort-028-854-753	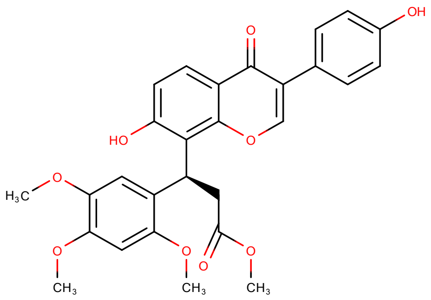	6.2	6.8	6.5

658	ZINC00480646	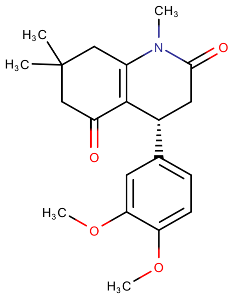	6.2	6.8	6.5
659	ZINC11866575	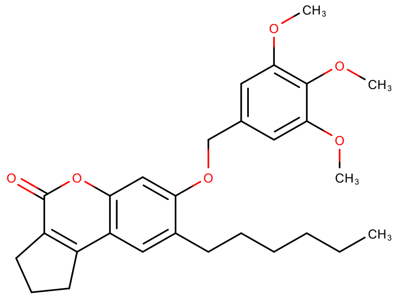	6.1	6.9	6.5
660	3037	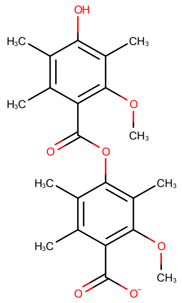	6.1	6.9	6.5
661	ZINC04046044	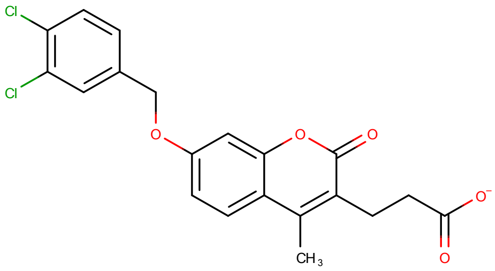	6.1	6.9	6.5
662	ZINC02148012	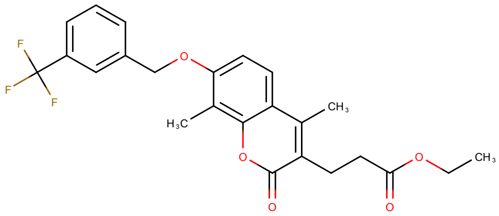	6.5	6.4	6.45

663	ZINC09357294	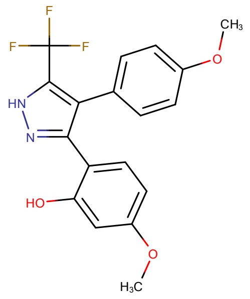	6.4	6.5	6.45
664	SN00092731	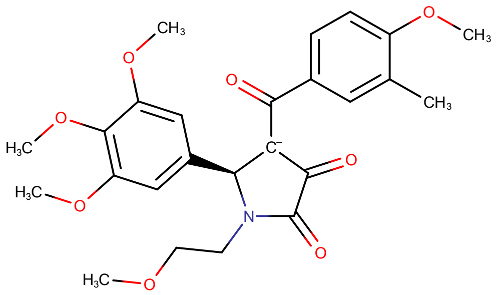	6.4	6.5	6.45
665	ZINC04222564	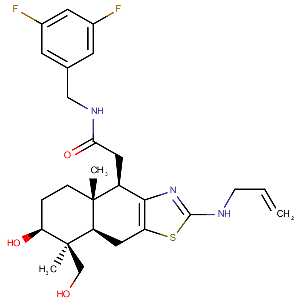	6.4	6.5	6.45
666	SN00107363	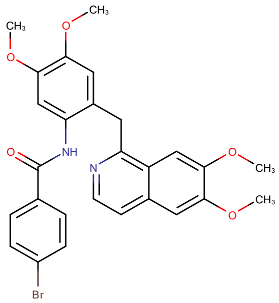	6.4	6.5	6.45
667	ZINC02151928	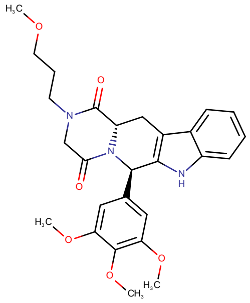	6.4	6.5	6.45

668	MolPort-044-180-902	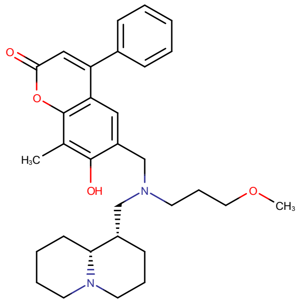	6.2	6.7	6.45
669	SN00080681	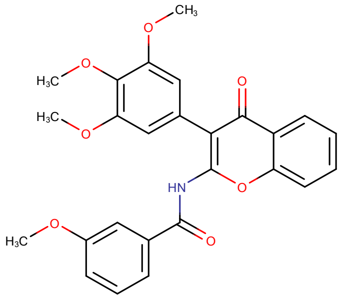	6.2	6.7	6.45
670	ZINC02120791	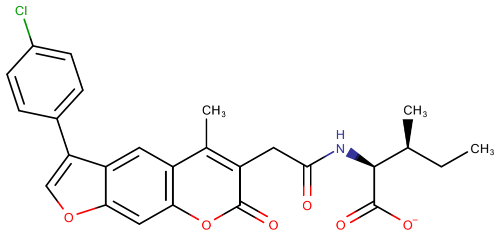	6.2	6.7	6.45
671	SN00376520	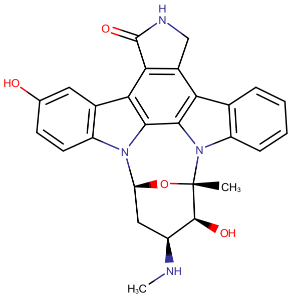	6.2	6.7	6.45
672	SN00333183	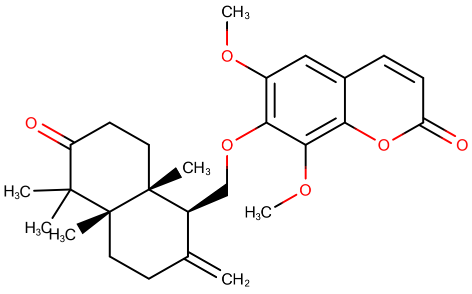	6.2	6.7	6.45

673	189083-78-1	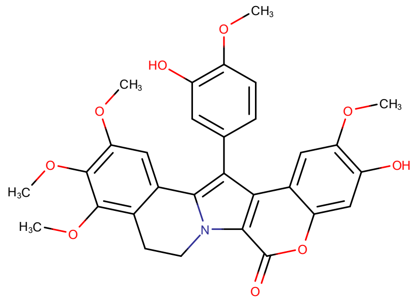	6.2	6.7	6.45
674	ZINC02152224	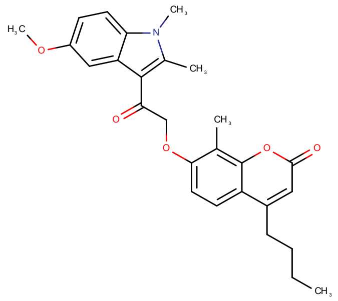	6.2	6.7	6.45
675	SN00294537	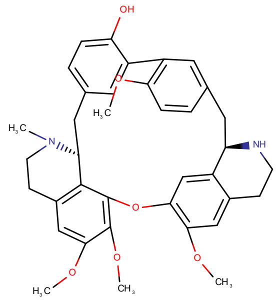	6.2	6.7	6.45
676	ZINC02092882	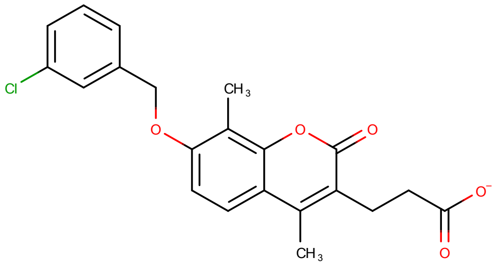	6.2	6.7	6.45
677	MolPort-044-543-486	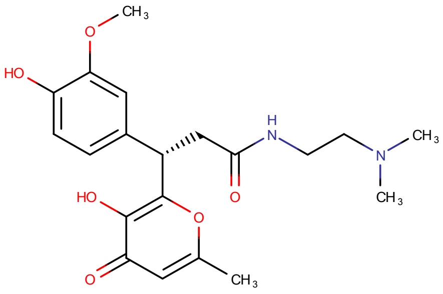	6	6.9	6.45

678	ZINC02148765	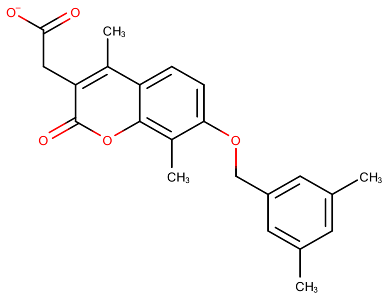	5.9	7	6.45
679	SN00333183	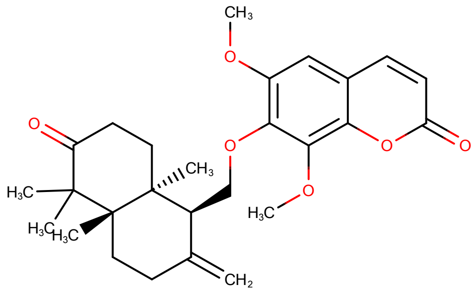	6.3	6.6	6.45
680	ZINC06058691	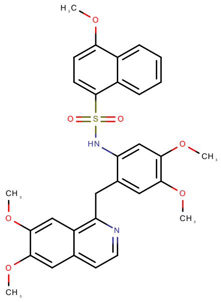	6.3	6.6	6.45
681	ZINC68575391	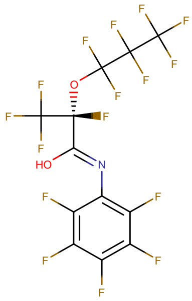	6.3	6.6	6.45
682	ZINC06075836	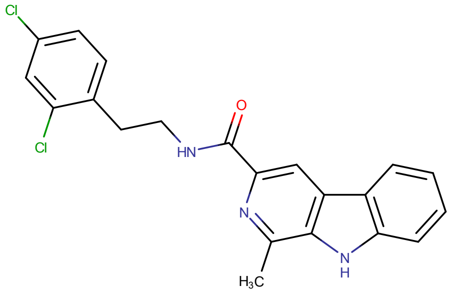	6.3	6.6	6.45

683	SN00366689	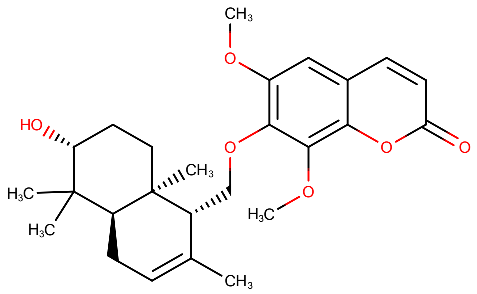	6.3	6.6	6.45
684	ZINC02095090	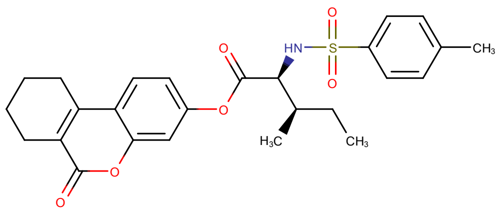	6.3	6.6	6.45
685	MolPort-027-718-092	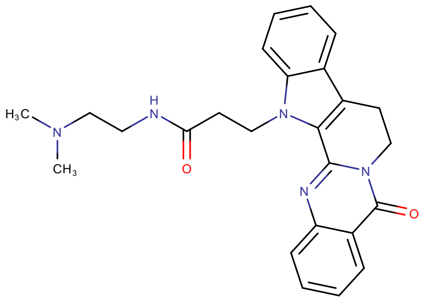	6.3	6.6	6.45
686	ZINC30882130	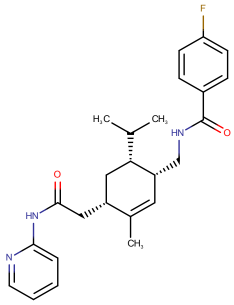	6.1	6.8	6.45
687	ZINC20466245	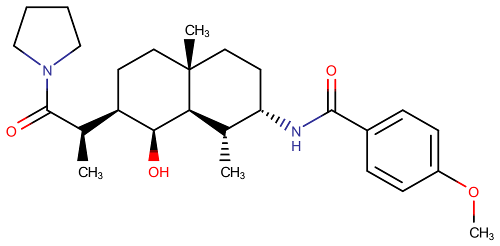	6.5	6.3	6.4

688	ZINC02125435	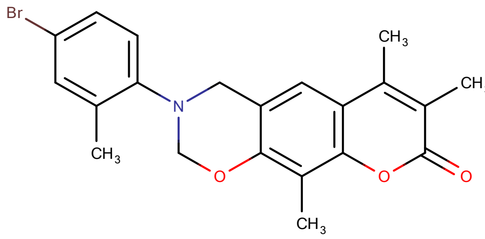	6.4	6.4	6.4
689	ZINC02227306	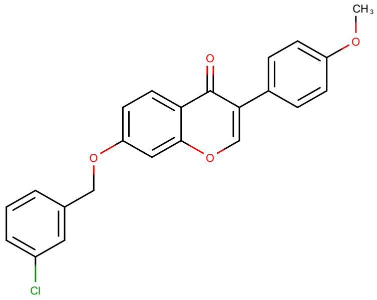	6.4	6.4	6.4
690	MolPort-038-386-398	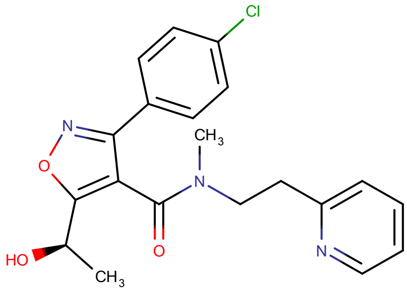	6.4	6.4	6.4
691	MolPort-028-855-303	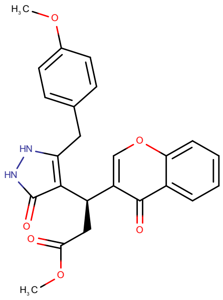	6.4	6.4	6.4
692	MolPort-002-513-842	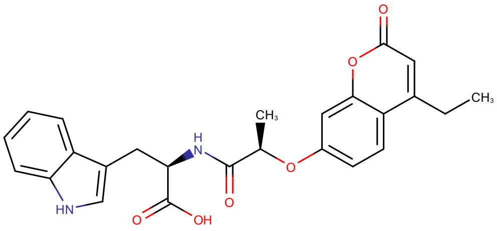	6.3	6.5	6.4

693	MolPort-000-847-367	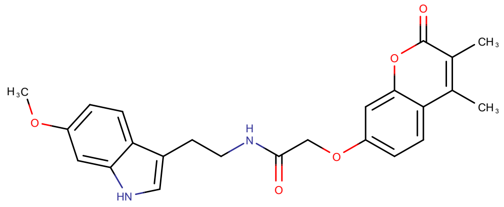	6.3	6.5	6.4
694	MolPort-001-573-006	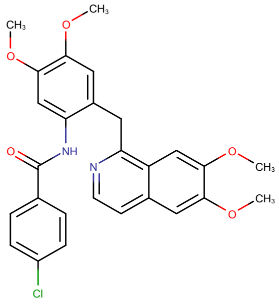	6.3	6.5	6.4
695	MolPort-028-855-404	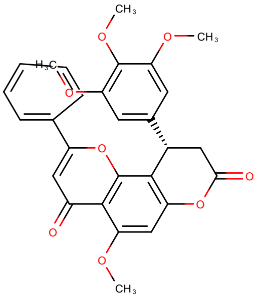	6.2	6.6	6.4
696	ZINC04236557	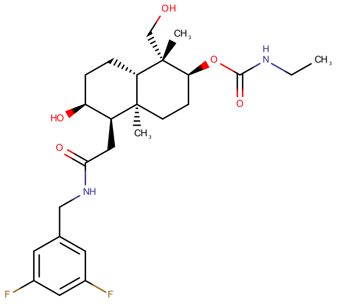	6.2	6.6	6.4
697	ZINC20761201	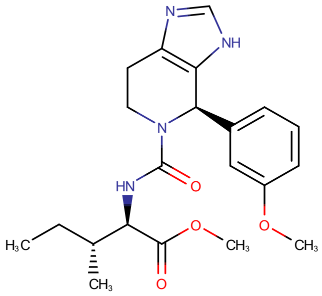	6.2	6.6	6.4

698	62008-00-8	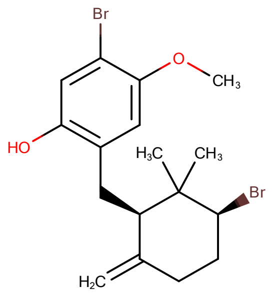	6.2	6.6	6.4
699	ZINC01902806	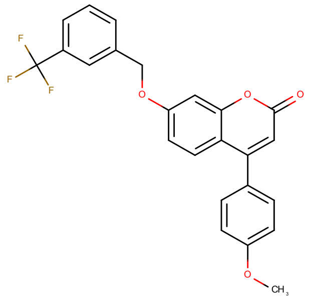	6.2	6.6	6.4
700	ZINC35466060	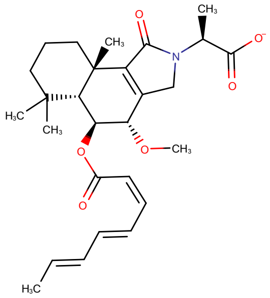	6.1	6.7	6.4
701	SN00074324	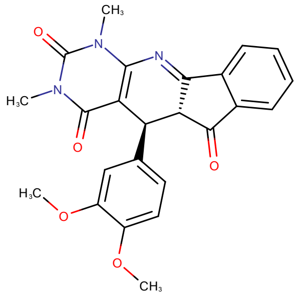	6.1	6.7	6.4
702	ZINC30880470	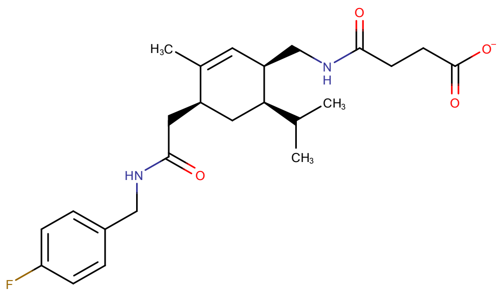	6.1	6.7	6.4

703	SN00092731	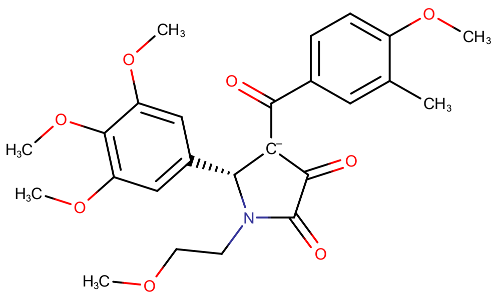	6	6.8	6.4
704	ZINC02093739	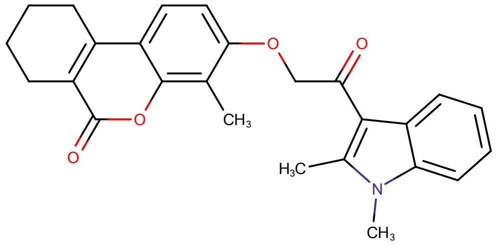	6	6.8	6.4
705	ZINC03999825	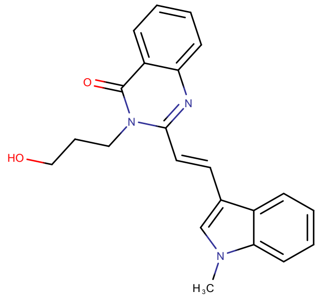	5.9	6.9	6.4
706	MolPort-000-797-746	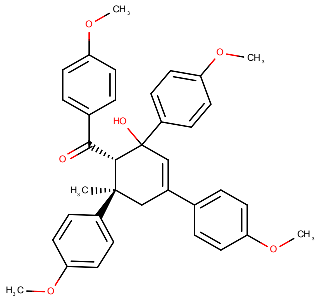	5.9	6.9	6.4
707	ZINC72325705	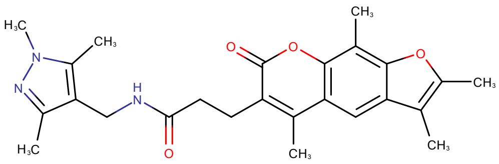	6.5	6.2	6.35

708	ZINC02345008	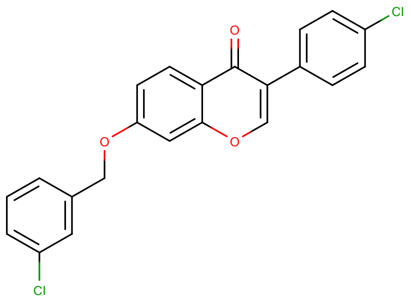	6.5	6.2	6.35
709	SN00372645	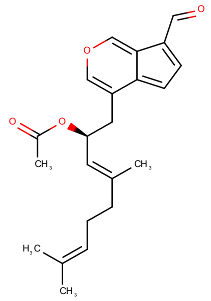	6.3	6.4	6.35
710	ZINC06624183	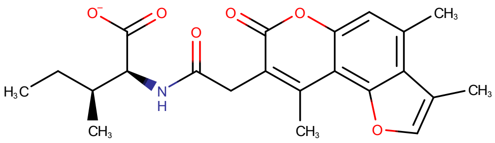	6.3	6.4	6.35
711	SN00102958	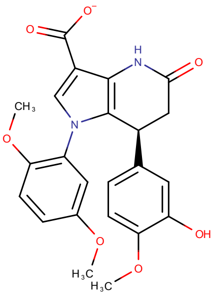	6.3	6.4	6.35
712	MolPort-001-737-182	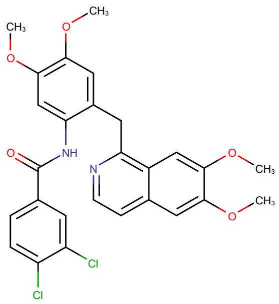	6.2	6.5	6.35

713	169181-25-3	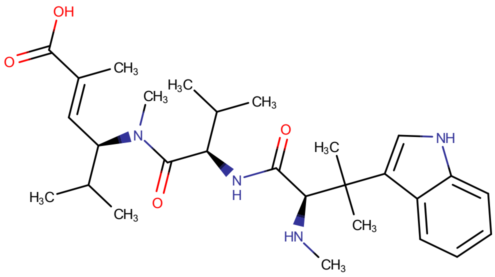	6.2	6.5	6.35
714	SN00295762	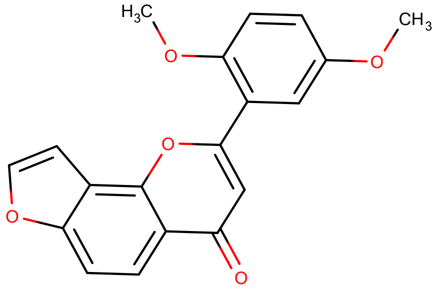	6.2	6.5	6.35
715	SN00379562	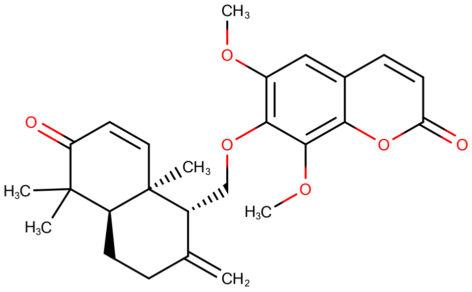	6.2	6.5	6.35
716	ZINC20611253	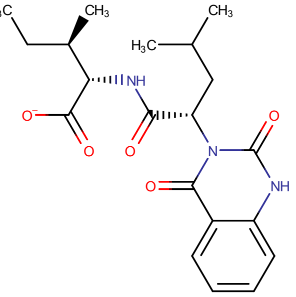	6	6.7	6.35
717	SN00282943	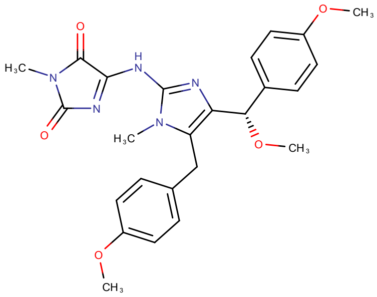	6.4	6.2	6.3

718	ZINC12896984	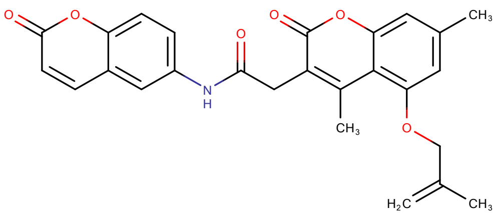	6.2	6.4	6.3
719	ZINC38139997	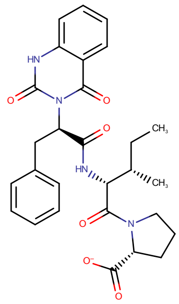	6.2	6.4	6.3
720	SN00083504	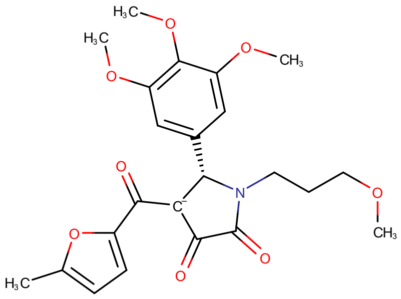	6.2	6.4	6.3
721	ZINC02145888	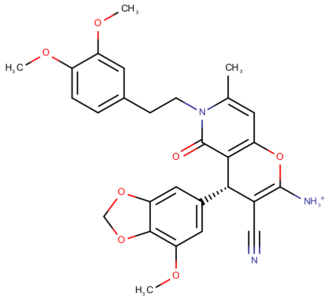	6.2	6.4	6.3
722	MolPort-009-649-148	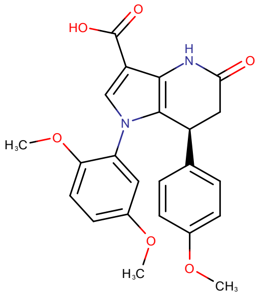	6.2	6.4	6.3

723	SN00138838	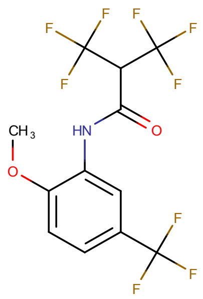	6.2	6.4	6.3
724	ZINC68575413	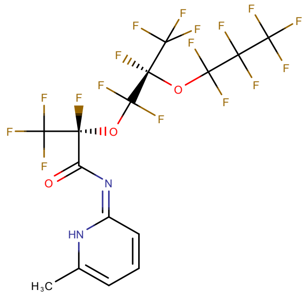	6.2	6.4	6.3
725	ZINC04273464	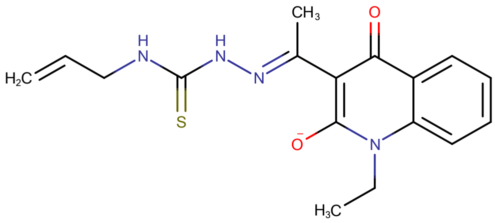	6.2	6.4	6.3
726	ZINC02151831	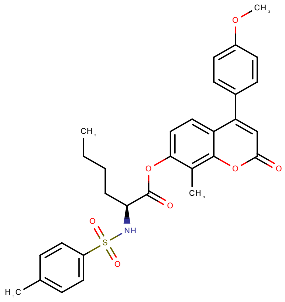	5.9	6.7	6.3
727	ZINC11865226	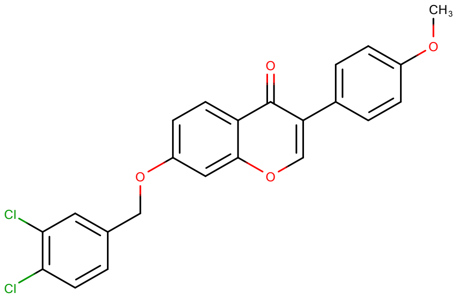	6.5	6.1	6.3

728	SN00061024	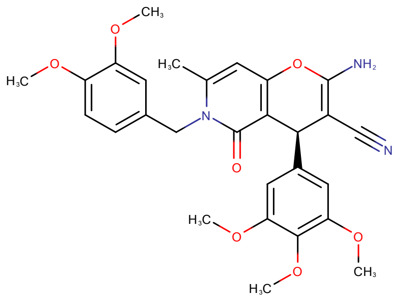	6.3	6.3	6.3
729	MolPort-028-855-479	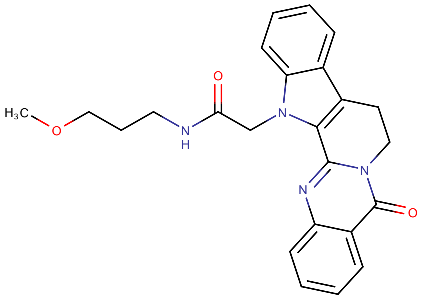	6.3	6.3	6.3
730	ZINC06075844	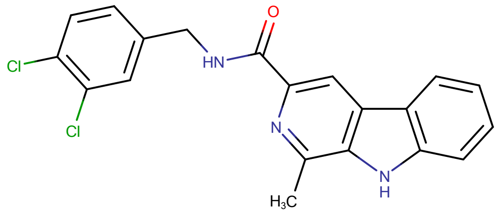	6.3	6.3	6.3
731	ZINC20466733	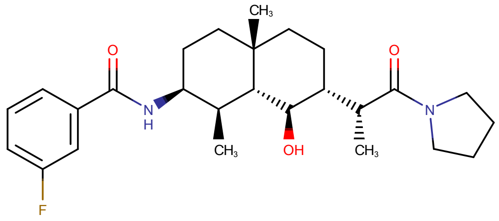	6.3	6.3	6.3
732	23230	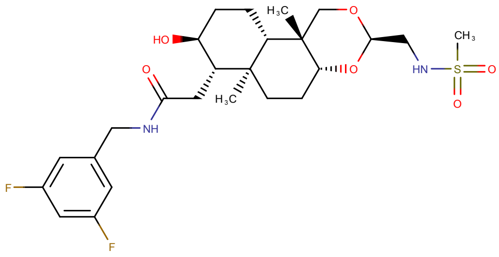	6.1	6.5	6.3

733	ZINC71386405	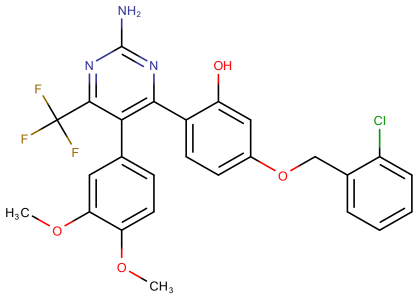	6.1	6.5	6.3
734	ZINC48057069	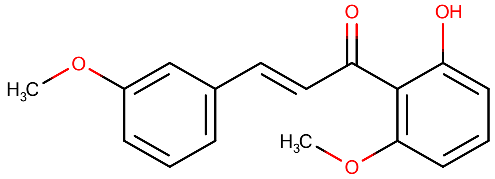	6.1	6.5	6.3
735	SN00258775	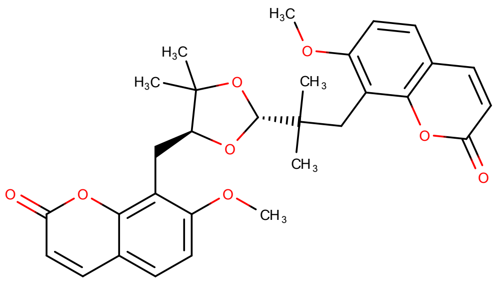	6.1	6.5	6.3
736	ZINC02092390	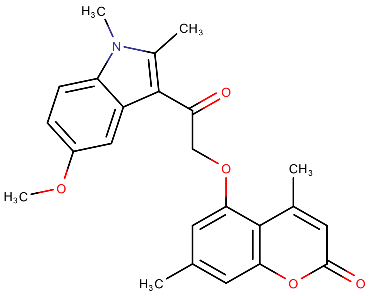	6.1	6.5	6.3
737	MolPort-009-649-144	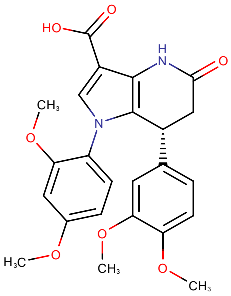	6	6.6	6.3

738	SN00073804	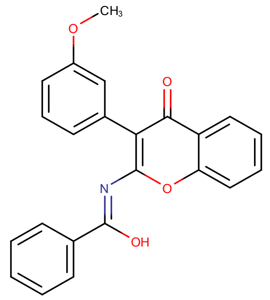	6	6.6	6.3
739	SN00083510	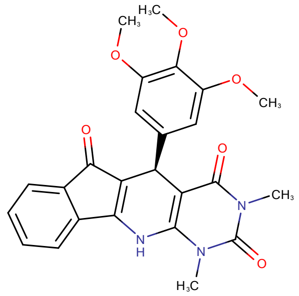	6	6.6	6.3
740	ZINC02114015	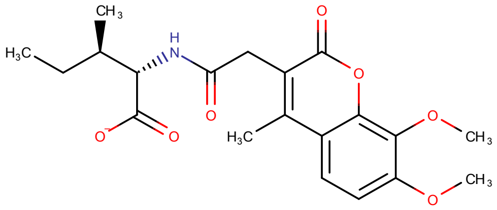	5.8	6.8	6.3
741	ZINC11865162	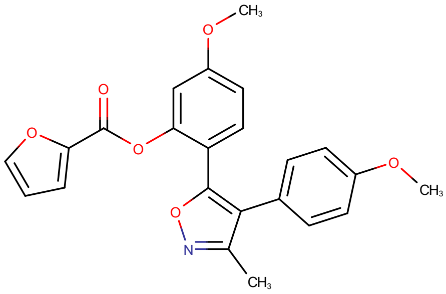	6.4	6.1	6.25
742	MolPort-002-525-974	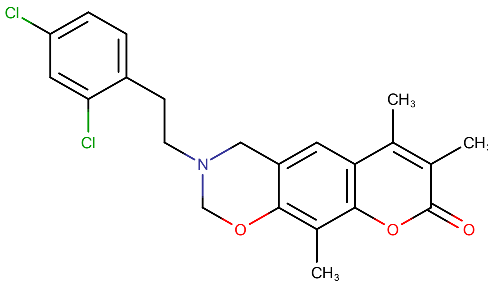	6.2	6.3	6.25

743	ZINC02096348	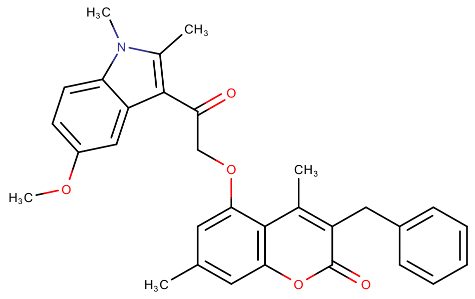	6.2	6.3	6.25
744	MolPort-005-950-564	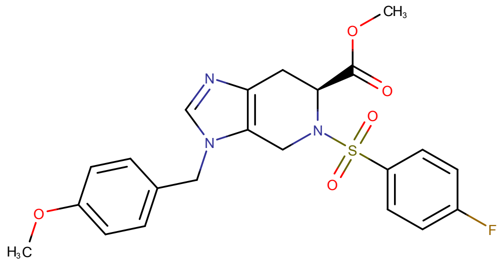	6.2	6.3	6.25
745	ZINC79189935	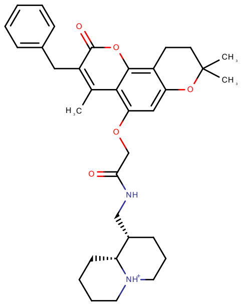	6.2	6.3	6.25
746	MolPort-001-745-897	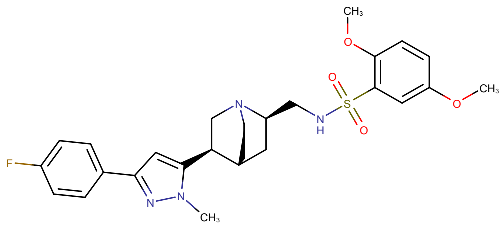	6.1	6.4	6.25
747	ZINC17198153	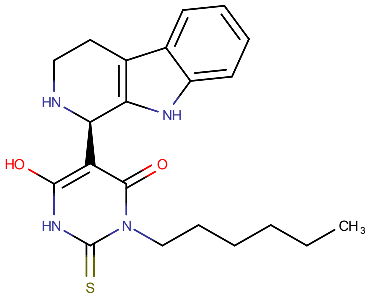	6.1	6.4	6.25

748	MolPort-039-195-378	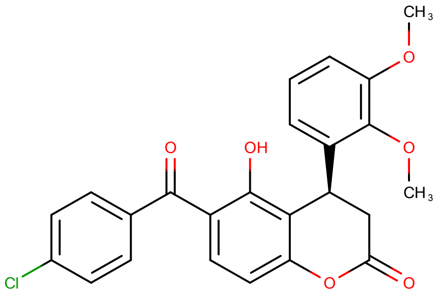	6.1	6.4	6.25
749	ZINC20467354	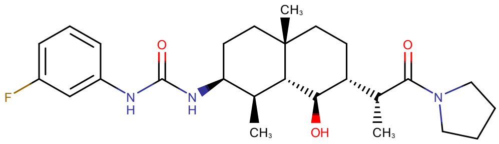	6.1	6.4	6.25
750	SN00053349	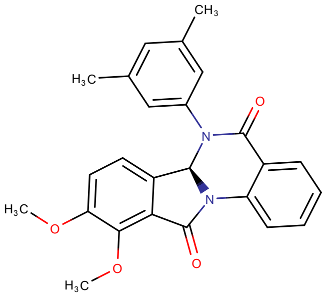	6.1	6.4	6.25
751	ZINC49543301	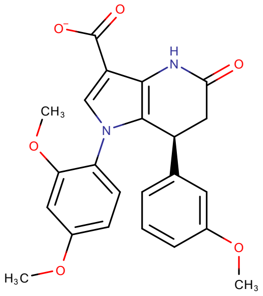	6	6.5	6.25
752	ZINC02095669	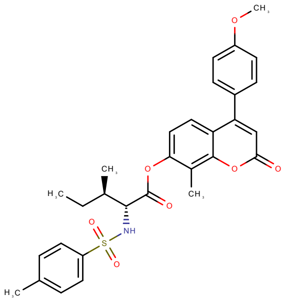	6	6.5	6.25

753	143682-18-2	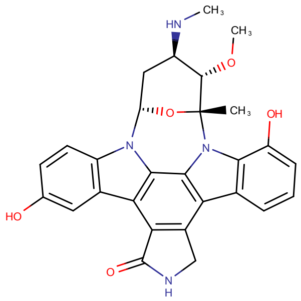	6	6.5	6.25
754	129623-30-9	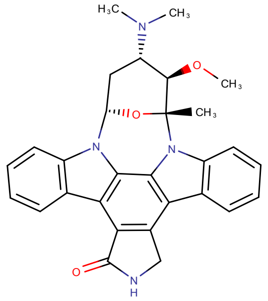	6	6.5	6.25
755	MolPort-007-564-138	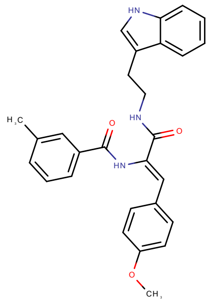	6	6.5	6.25
756	ZINC04046667	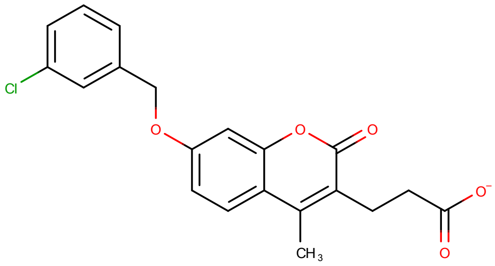	6	6.5	6.25
757	ZINC72324754	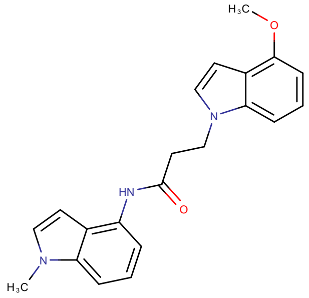	6	6.5	6.25

758	MolPort-005-910-869	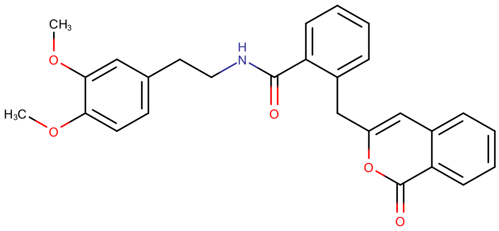	5.9	6.6	6.25
759	MolPort-008-348-717	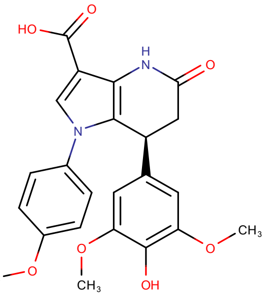	5.9	6.6	6.25
760	SN00077983	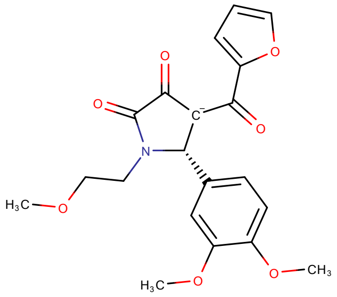	5.9	6.6	6.25
761	ZINC04062053	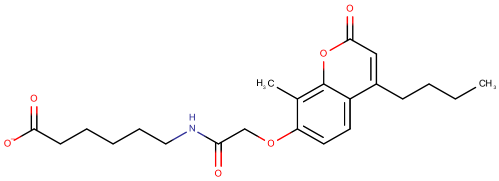	5.9	6.6	6.25
762	MolPort-000-813-035	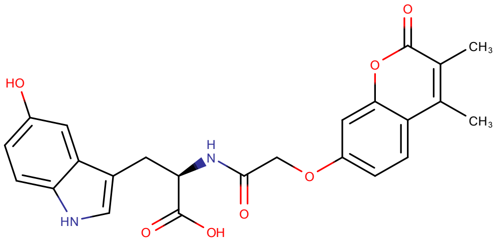	5.8	6.7	6.25

763	ZINC11865225	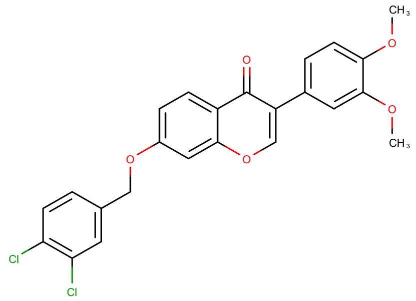	6.4	6	6.2
764	MolPort-039-194-841	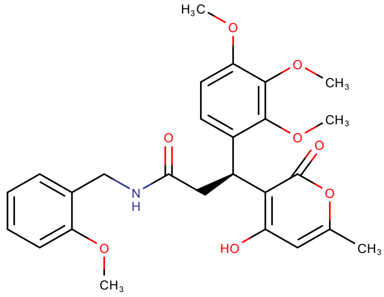	6.2	6.2	6.2
765	MolPort-044-810-910	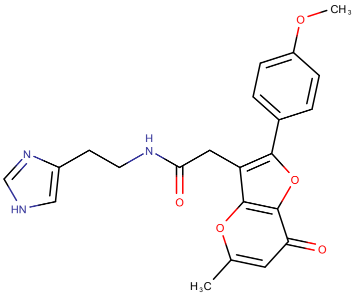	6.2	6.2	6.2
766	SN00161478	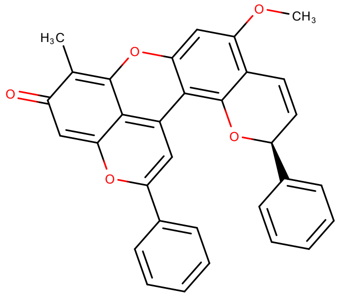	6.2	6.2	6.2
767	ZINC08918194	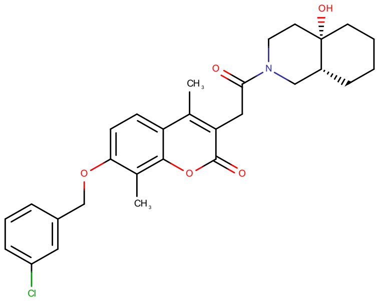	6.2	6.2	6.2

768	ZINC05817910	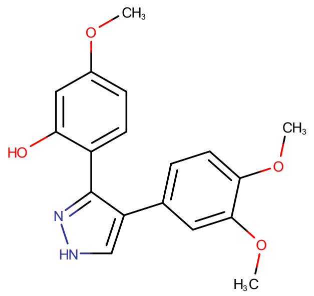	6	6.4	6.2
769	ZINC12899640	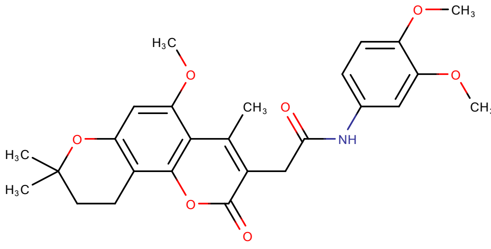	6	6.4	6.2
770	ZINC38549848	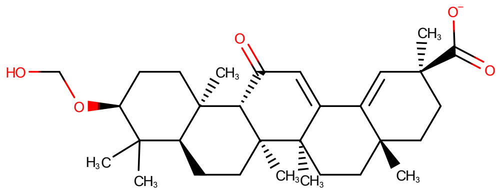	6	6.4	6.2
771	ZINC02119925	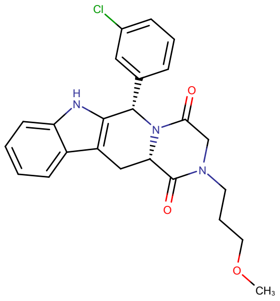	6	6.4	6.2
772	MolPort-009-649-155	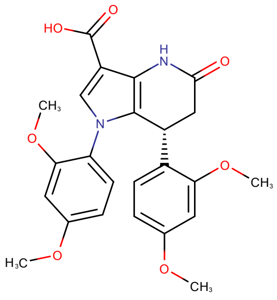	6	6.4	6.2

773	MolPort-002-524-289	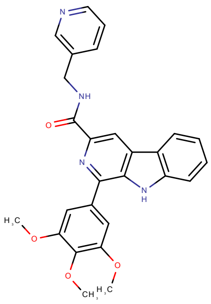	6	6.4	6.2
774	ZINC49543327	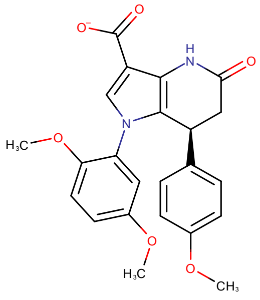	5.7	6.7	6.2
775	155233-31-1	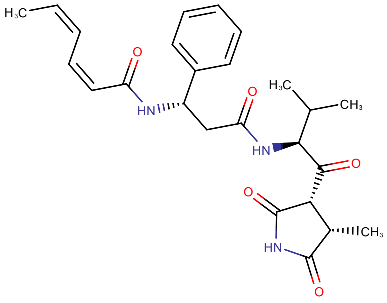	6.3	6.1	6.2
776	181423-71-2	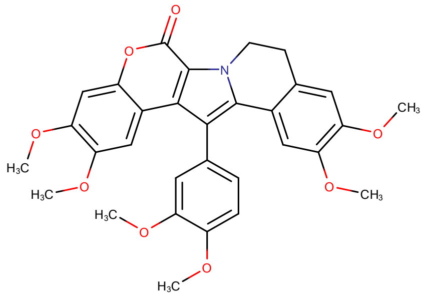	6.3	6.1	6.2
777	ZINC04046180	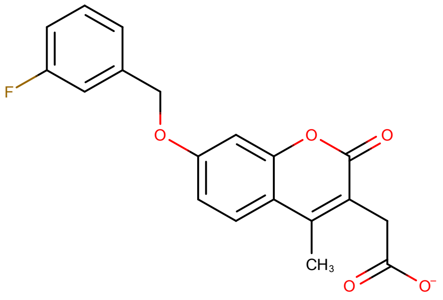	6.1	6.3	6.2

778	MolPort-028-856-366	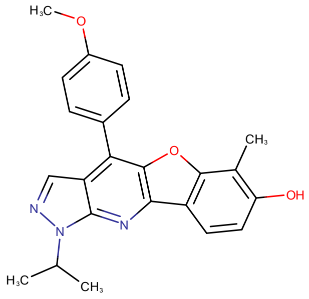	6.1	6.3	6.2
779	MolPort-007-902-409	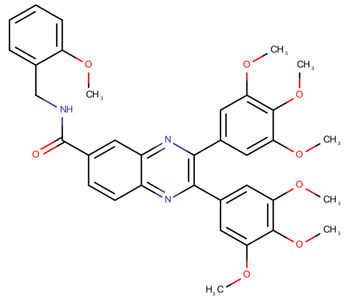	6.1	6.3	6.2
780	ZINC02120633	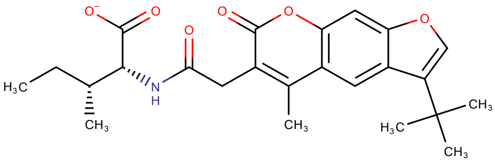	5.8	6.6	6.2
781	12190	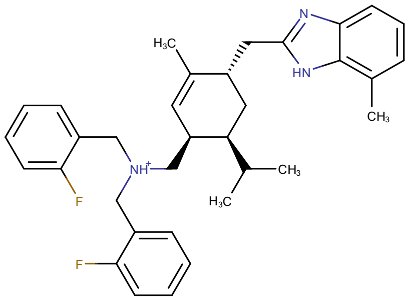	6.3	6	6.15
782	99457-91-7	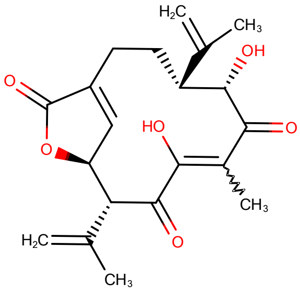	6.2	6.1	6.15

783	ZINC04089321	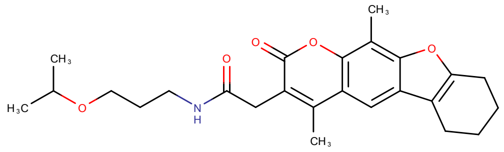	6.1	6.2	6.15
784	ZINC02149278	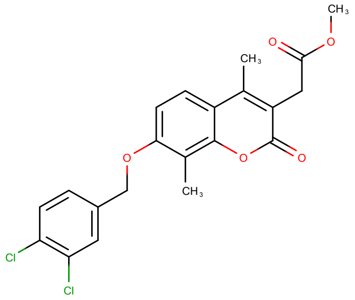	6.1	6.2	6.15
785	ZINC30881458	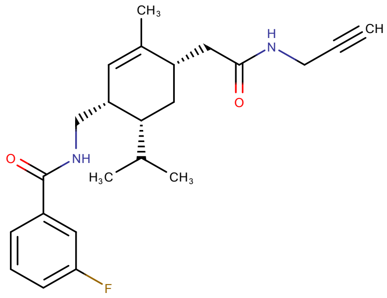	6	6.3	6.15
786	MolPort-019-936-025	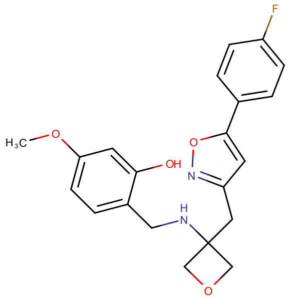	5.9	6.4	6.15
787	ZINC02104075	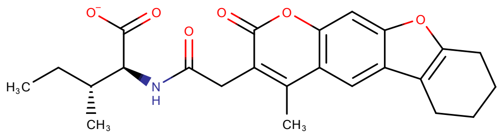	5.8	6.5	6.15

788	MolPort-002-533-189	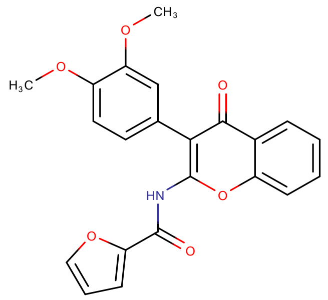	5.7	6.6	6.15
789	ZINC02092427	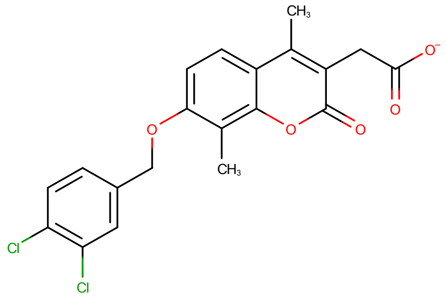	5.7	6.6	6.15
790	MolPort-028-854-697	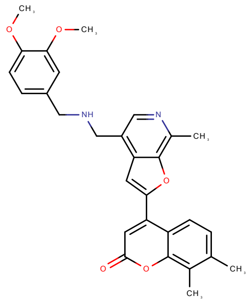	6.2	6	6.1
791	ZINC19927379	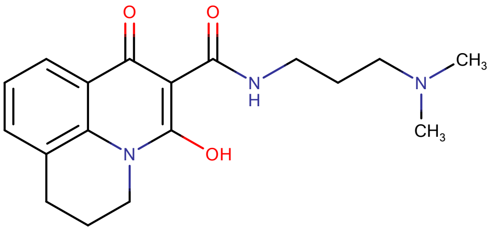	6.1	6.1	6.1
792	SN00060965	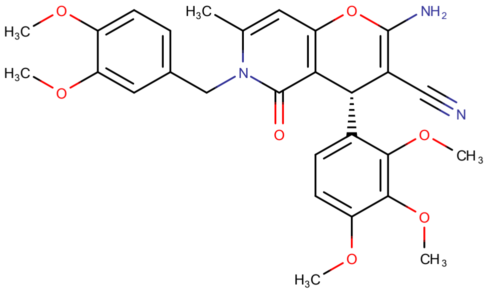	6.1	6.1	6.1

793	SN00062839	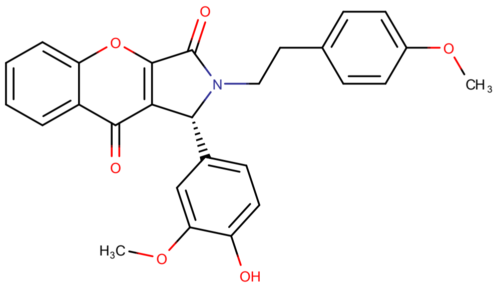	6	6.2	6.1
794	ZINC12875599	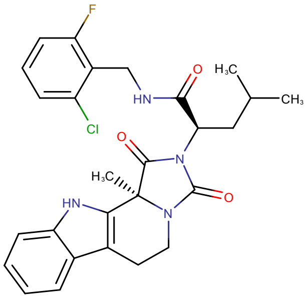	6	6.2	6.1
795	MolPort-028-856-471	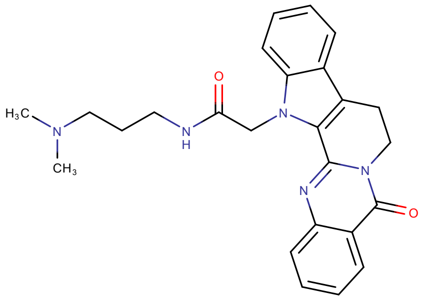	6	6.2	6.1
796	ZINC08791756	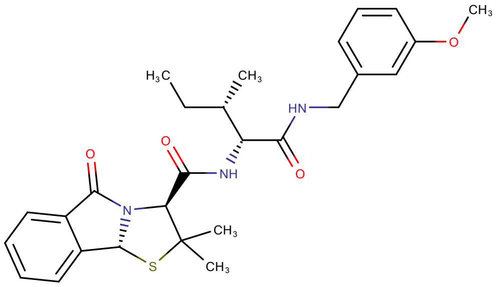	6	6.2	6.1
797	MolPort-009-649-145	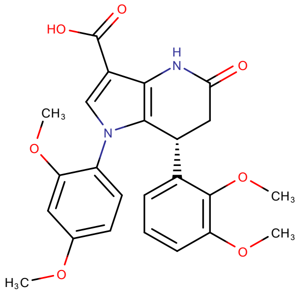	5.9	6.3	6.1

798	SN00073864	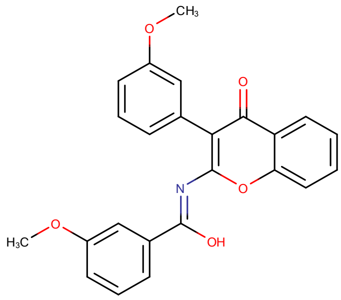	5.9	6.3	6.1
799	MolPort-002-087-773	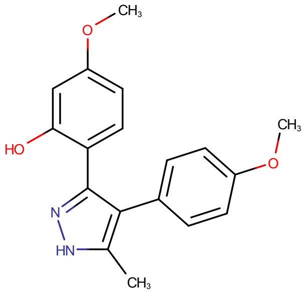	5.9	6.3	6.1
800	ZINC03846616	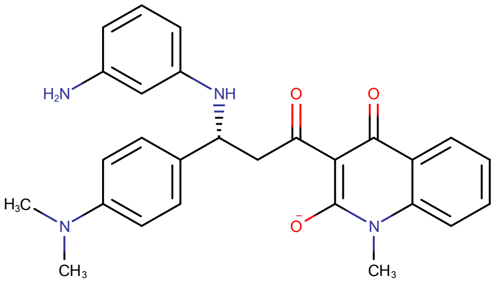	5.8	6.4	6.1
801	MolPort-035-873-330	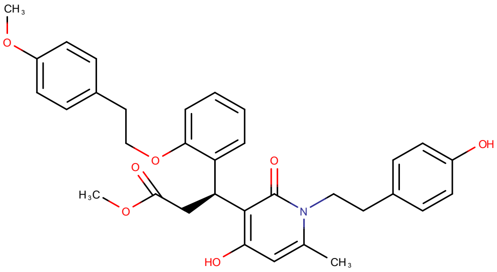	5.8	6.4	6.1
802	MolPort-009-649-145	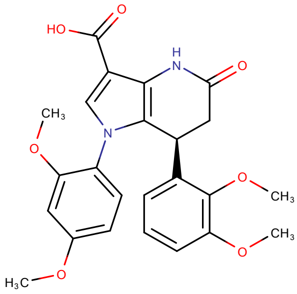	5.8	6.4	6.1

803	MolPort-035-702-025	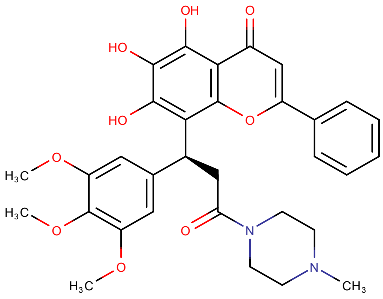	5.7	6.5	6.1
804	MolPort-005-950-438	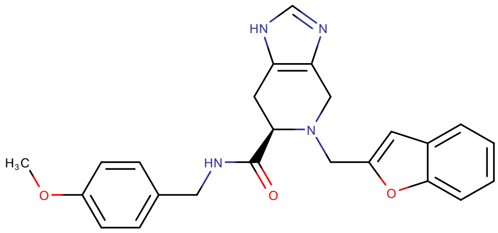	5.7	6.5	6.1
805	MolPort-002-533-108	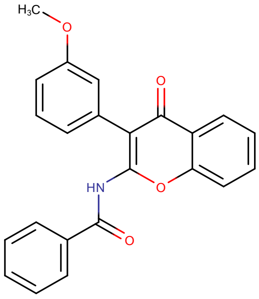	5.6	6.6	6.1
806	MolPort-002-529-261	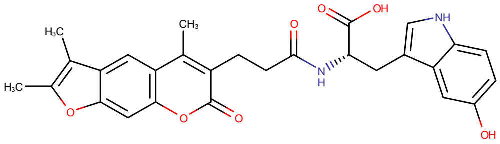	5.6	6.6	6.1
807	ZINC02148665	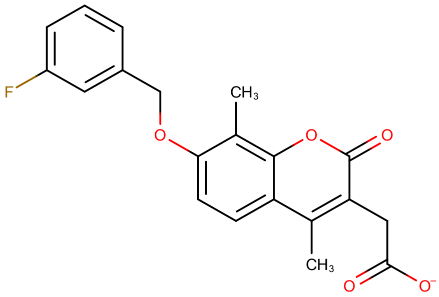	5.5	6.7	6.1

808	ZINC30881020	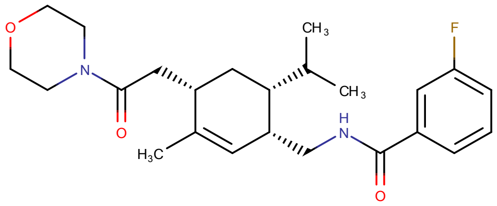	6.2	5.9	6.05
809	ZINC02095349	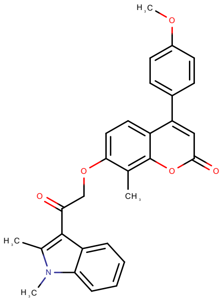	5.9	6.2	6.05
810	MolPort-000-814-627	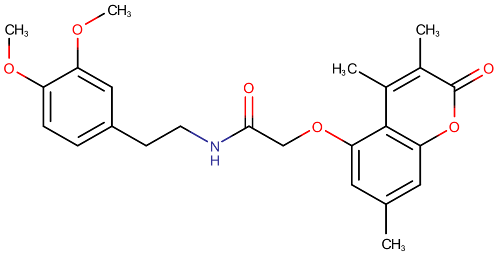	5.9	6.2	6.05
811	MolPort-039-057-703	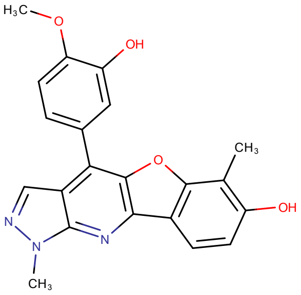	5.9	6.2	6.05
812	MolPort-008-348-717	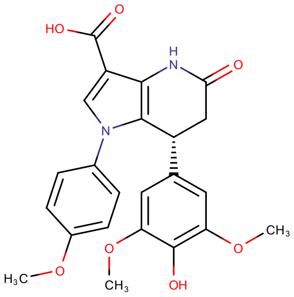	5.7	6.4	6.05

813	MolPort-000-792-600	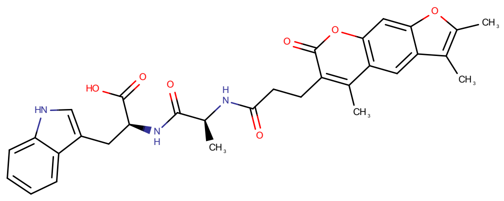	5.7	6.4	6.05
814	ZINC04023594	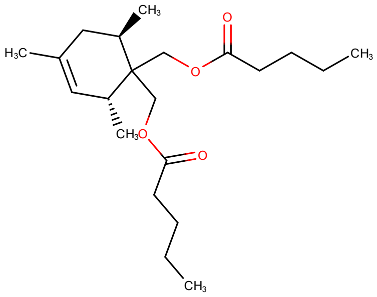	6.1	6	6.05
815	MolPort-002-518-223	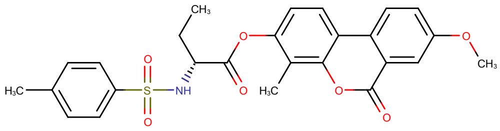	6	6.1	6.05
816	MolPort-002-523-304	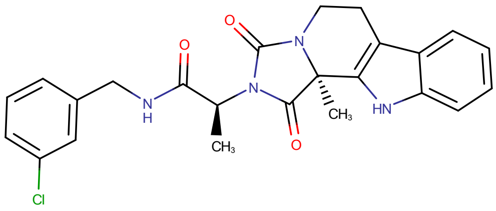	6	6.1	6.05
817	SN00243902	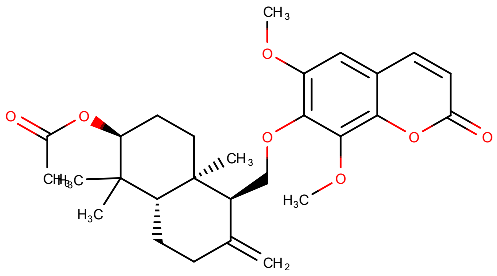	6	6.1	6.05

818	149378-57-4	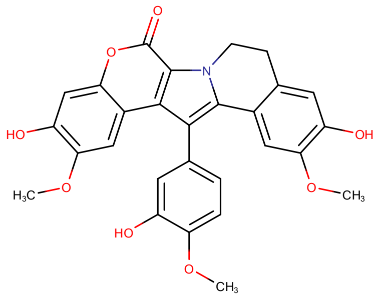	6	6.1	6.05
819	ZINC35466060	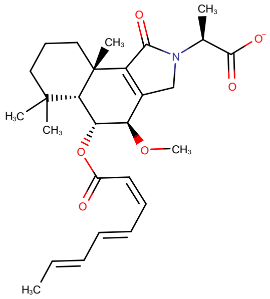	5.8	6.3	6.05
820	9574	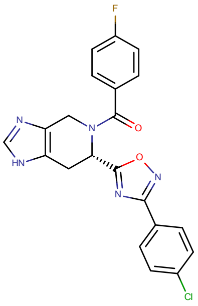	5.8	6.3	6.05
821	ZINC02125058	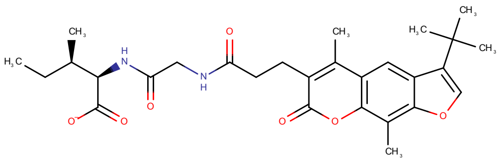	6.1	5.9	6
822	ZINC38143767	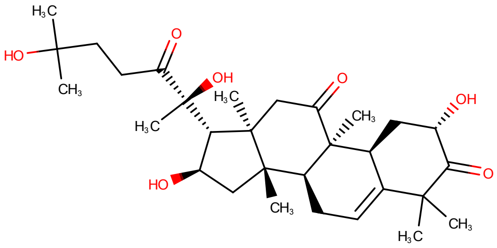	6.1	5.9	6

823	MolPort-002-518-797	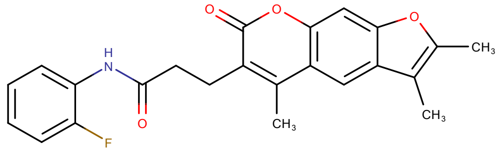	5.9	6.1	6
824	ZINC12899873	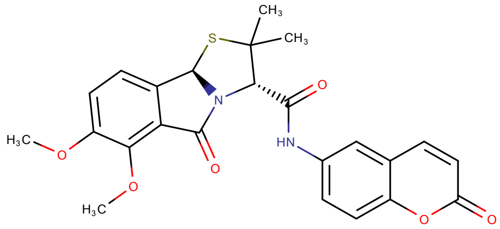	5.9	6.1	6
825	SN00344408	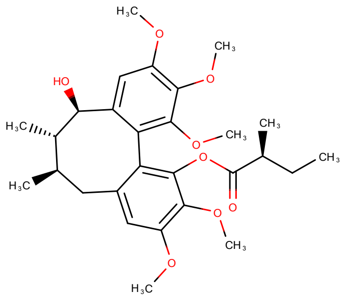	5.9	6.1	6
826	ZINC06167362	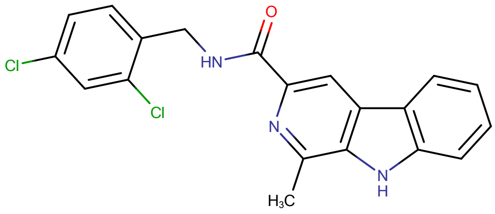	5.8	6.2	6
827	SN00335313	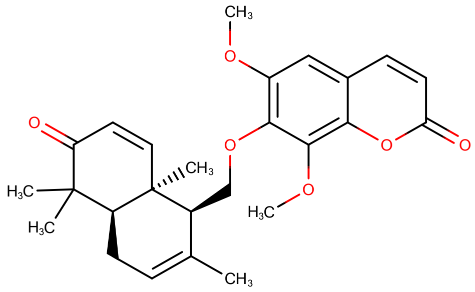	5.7	6.3	6

828	152273-84-2	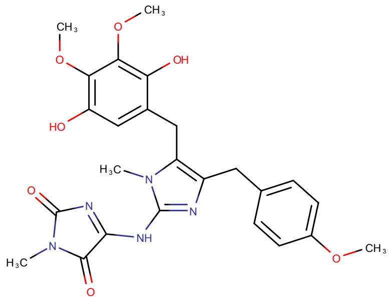	5.7	6.3	6
829	ZINC02123590	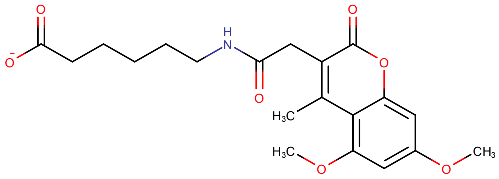	5.5	6.5	6
830	MolPort-002-518-554	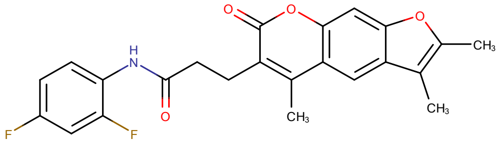	5.9	6	5.95
831	MolPort-038-386-405	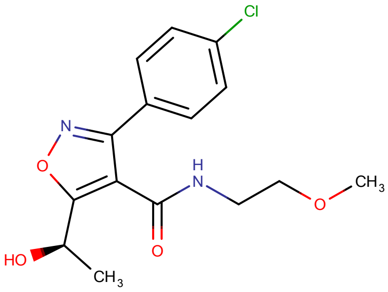	5.9	6	5.95
832	ZINC02095346	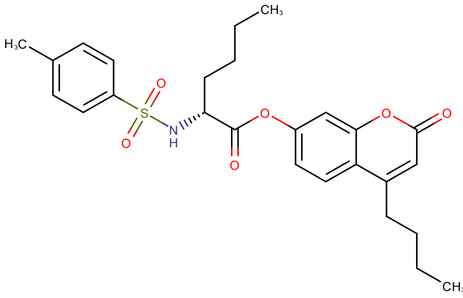	5.9	6	5.95

833	ZINC02667597	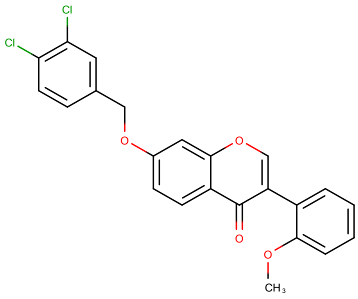	5.9	6	5.95
834	ZINC17146927	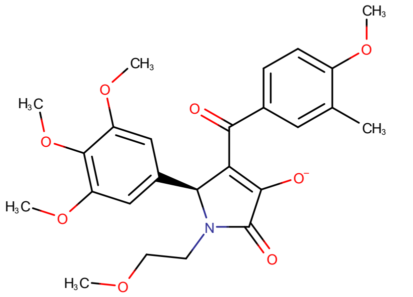	5.7	6.2	5.95
835	ZINC02157897	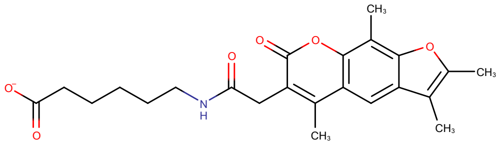	5.5	6.4	5.95
836	MolPort-039-194-689	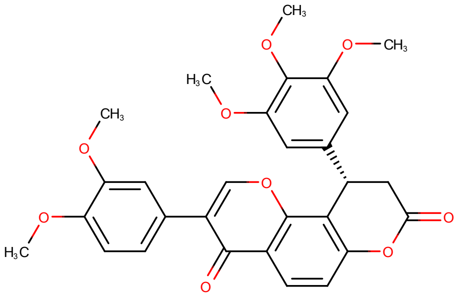	5.5	6.4	5.95
837	ZINC08790722	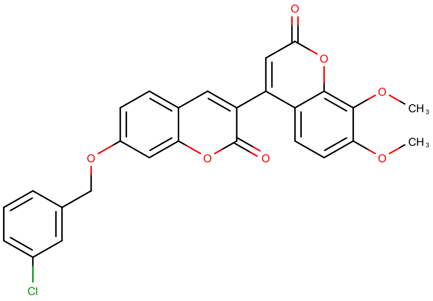	5.5	6.4	5.95

838	ZINC02117069	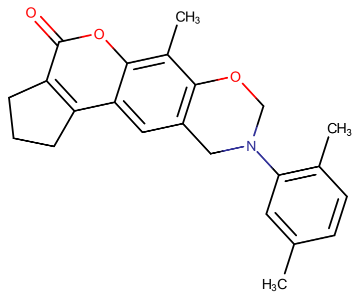	5.8	6.1	5.95
839	ZINC02125535	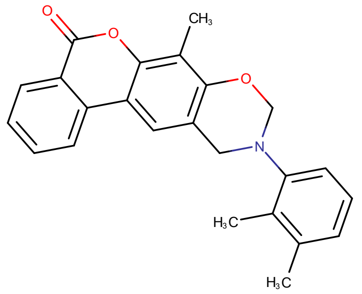	5.8	6.1	5.95
840	ZINC79192837	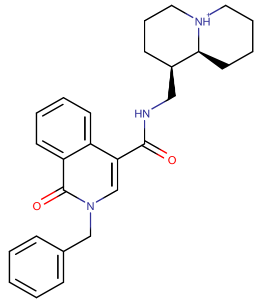	5.8	6.1	5.95
841	MolPort-009-649-144	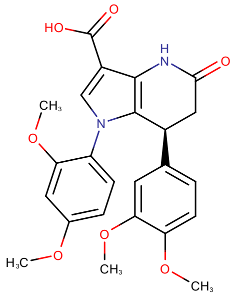	5.6	6.3	5.95
842	SN00051121	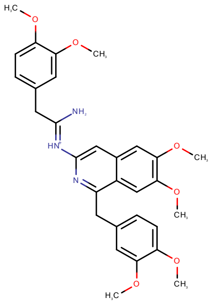	6	5.8	5.9

843	ZINC31168056	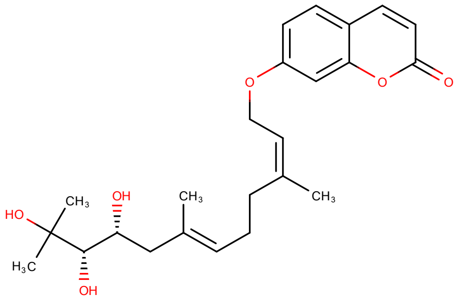	5.8	6	5.9
844	MolPort-039-194-968	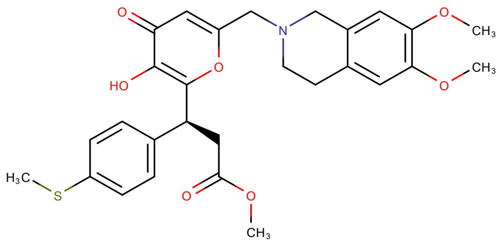	5.7	6.1	5.9
845	ZINC12296372	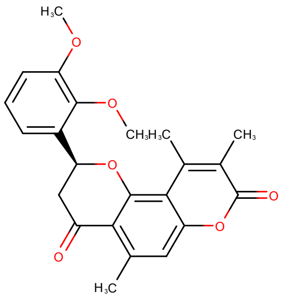	5.7	6.1	5.9
846	ZINC02123120	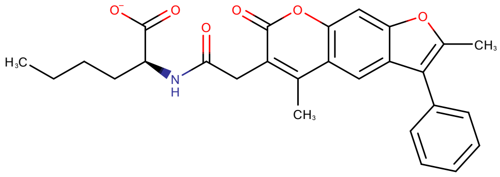	5.7	6.1	5.9
847	ZINC68589300	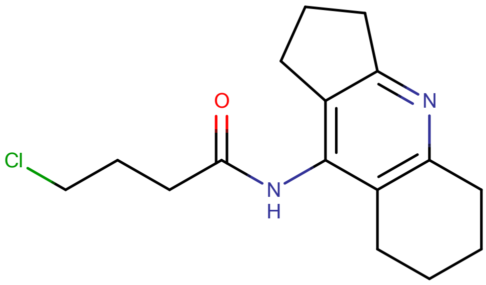	5.6	6.2	5.9

848	SN00102579	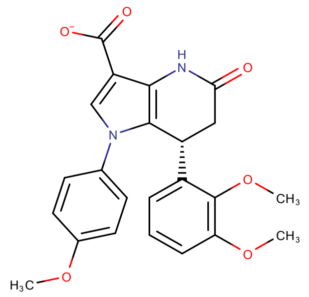	5.5	6.3	5.9
849	SN00305462	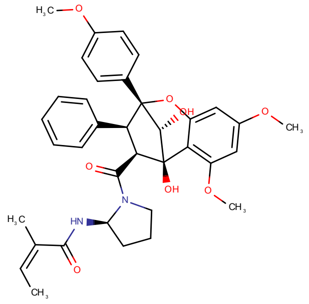	5.4	6.4	5.9
850	MolPort-007-733-445	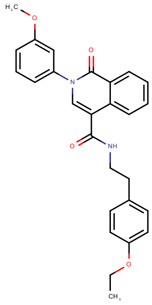	5.9	5.8	5.85
851	17917	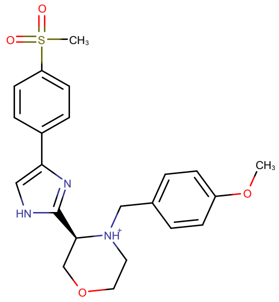	5.8	5.9	5.85
852	MolPort-001-999-765	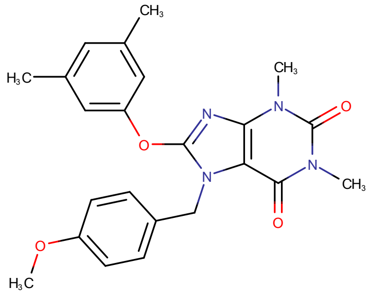	5.8	5.9	5.85

853	SN00103065	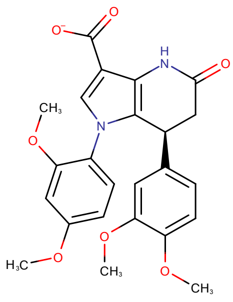	5.7	6	5.85
854	ZINC38143767	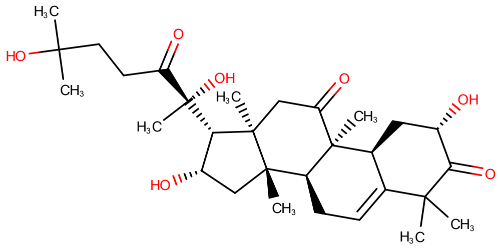	5.7	6	5.85
855	SN00254960	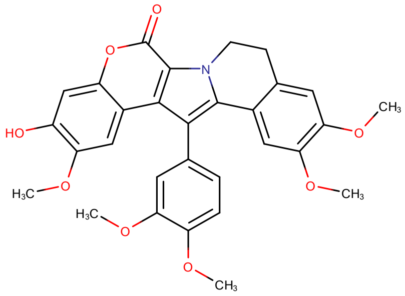	5.7	6	5.85
856	SN00103017	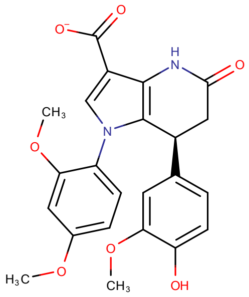	5.6	6.1	5.85
857	MolPort-044-810-666	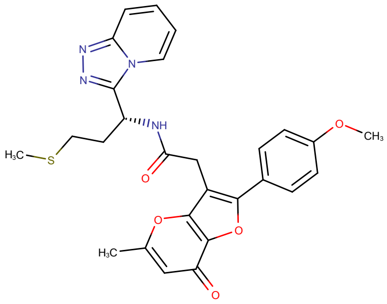	5.5	6.2	5.85

858	MolPort-042-674-422	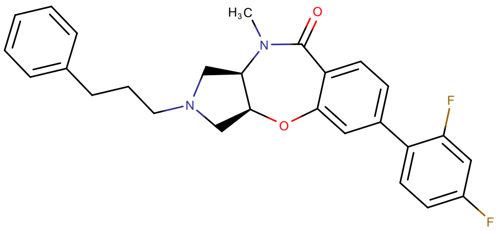	5.9	5.7	5.8
859	MolPort-007-787-617	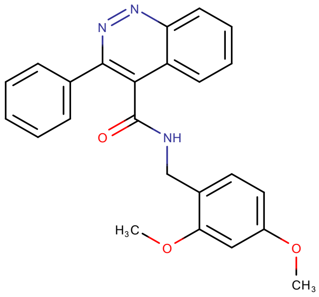	5.7	5.9	5.8
860	ZINC30883812	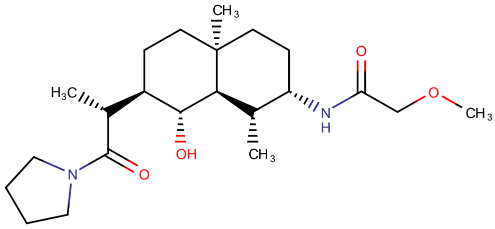	5.7	5.9	5.8
861	MolPort-044-544-159	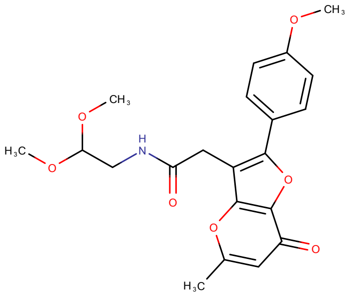	5.4	6.2	5.8
862	MolPort-002-533-223	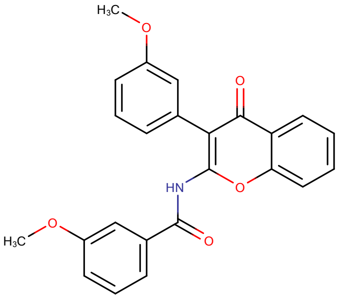	5.4	6.2	5.8

863	ZINC05783367	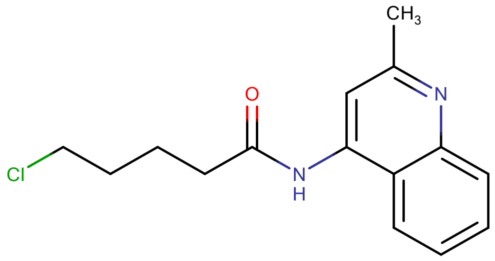	5.6	6	5.8
864	MolPort-002-184-761	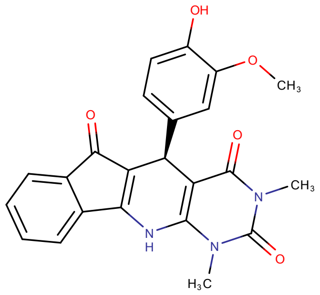	5.6	6	5.8
865	ZINC02095836	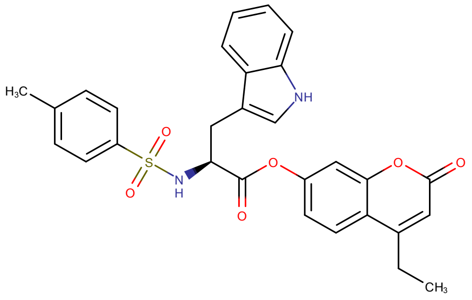	5.6	6	5.8
866	MolPort-001-683-688	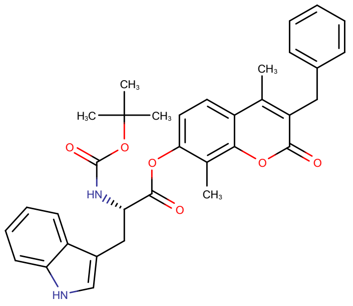	5.6	6	5.8
867	200936-84-1	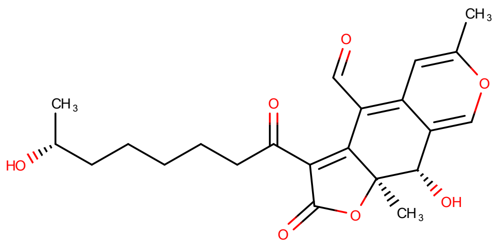	5.5	6.1	5.8

868	MolPort-001-906-348	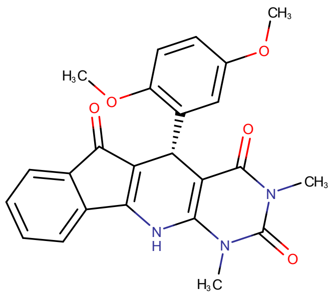	5.5	6.1	5.8
869	ZINC02114872	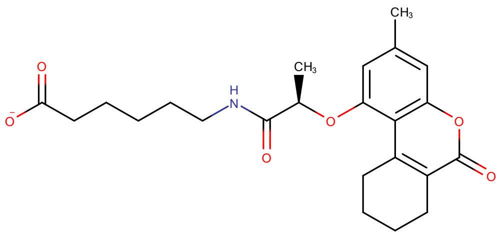	5.3	6.3	5.8
870	189083-80-5	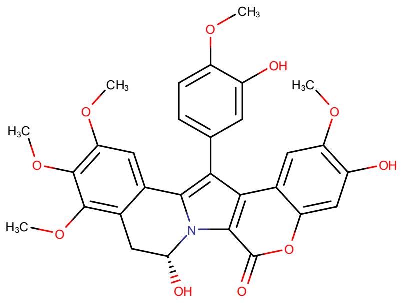	5.5	6	5.75
871	ZINC70687176	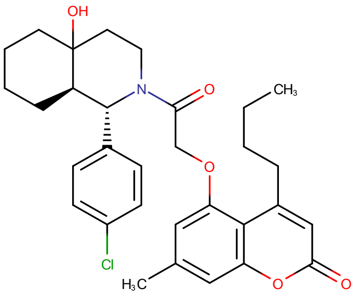	5.4	6.1	5.75
872	SN00061023	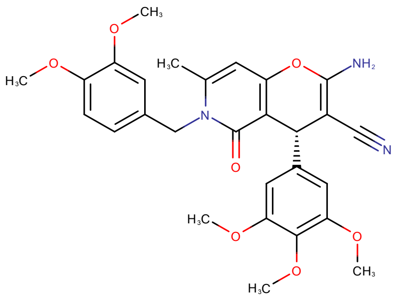	5.4	6.1	5.75

873	MolPort-008-348-069	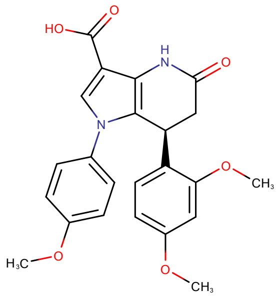	5.5	5.9	5.7
874	19310	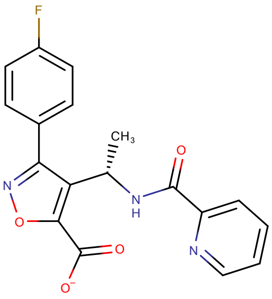	5.2	6.2	5.7
875	MolPort-001-728-081	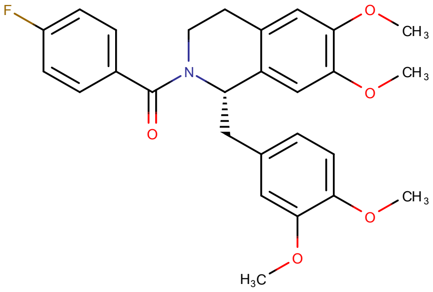	5.6	5.8	5.7
876	MolPort-008-348-079	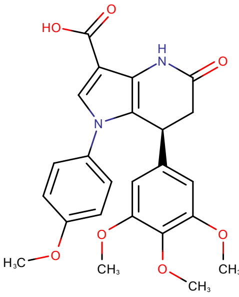	5.6	5.8	5.7
877	MolPort-044-543-703	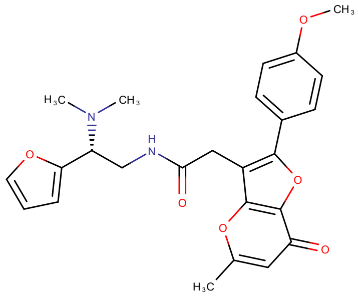	5.3	6.1	5.7

878	MolPort-009-649-107	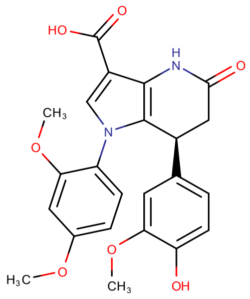	5.3	6.1	5.7
879	ZINC70705416	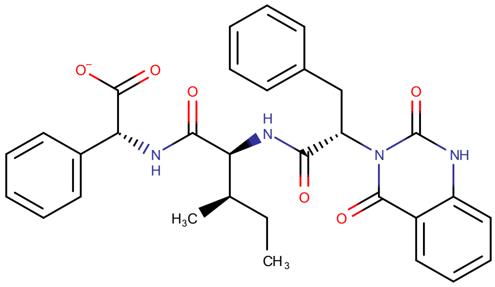	5.3	6.1	5.7
880	MolPort-001-907-854	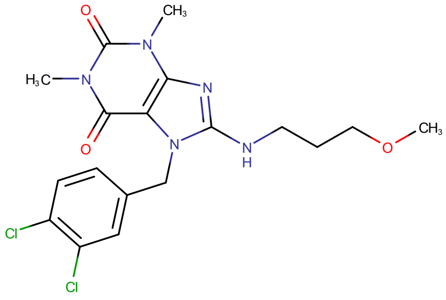	5.3	6.1	5.7
881	ZINC02125325	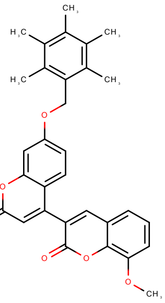	5.3	6.1	5.7
882	MolPort-002-277-679	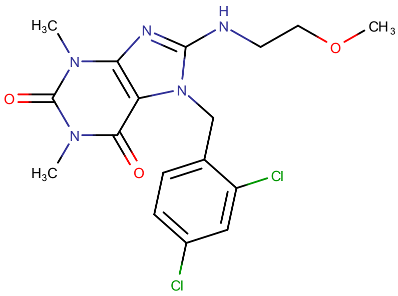	5.5	5.8	5.65

883	ZINC02115373	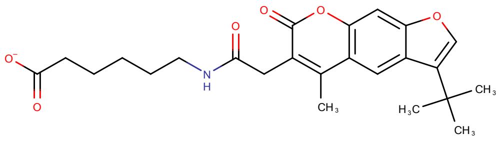	5.3	6	5.65
884	ZINC04046886	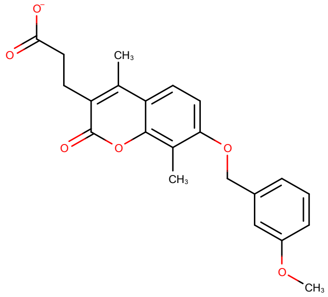	5.2	6.1	5.65
885	ZINC85879780	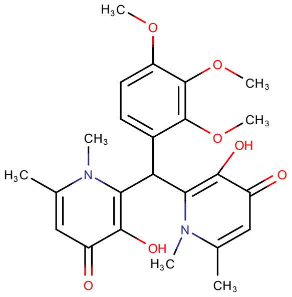	5.4	5.8	5.6
886	SN00063749	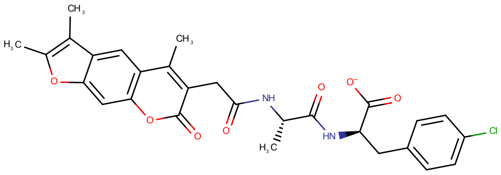	4.9	6.3	5.6
887	SN00098390	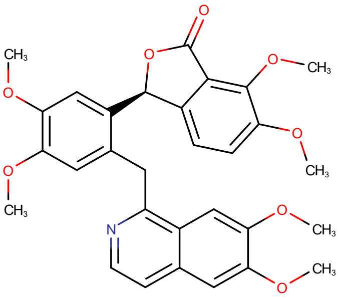	5.4	5.7	5.55

888	MolPort-009-649-139	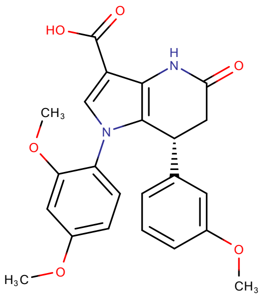	5.4	5.7	5.55
889	MolPort-002-510-470	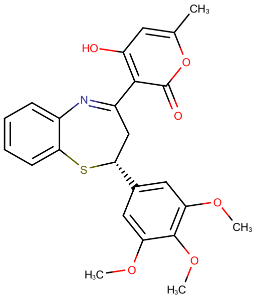	5.2	5.9	5.55
890	MolPort-005-307-707	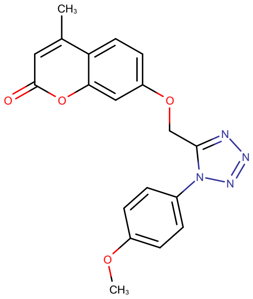	5.2	5.9	5.55
891	MolPort-008-348-309	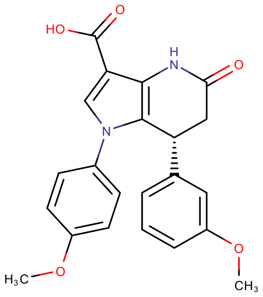	5.3	5.8	5.55
892	SN00102409	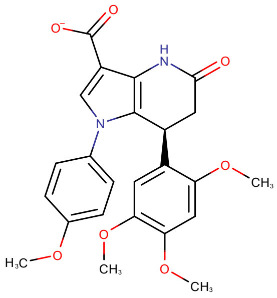	5.1	6	5.55

893	SN00103067	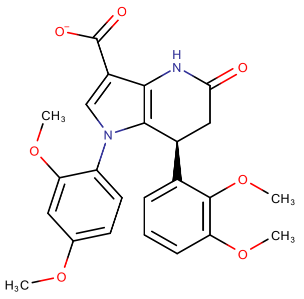	5	6.1	5.55
894	ZINC05452024	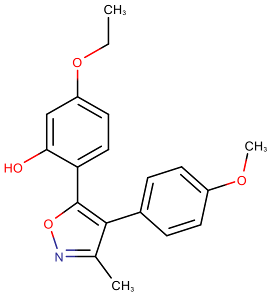	5.3	5.7	5.5
895	MolPort-007-796-366	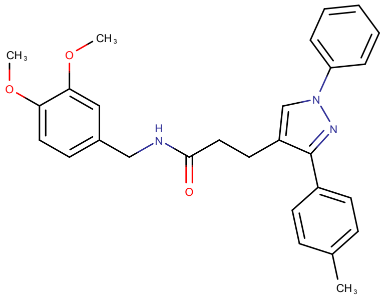	5.2	5.8	5.5
896	MolPort-001-973-554	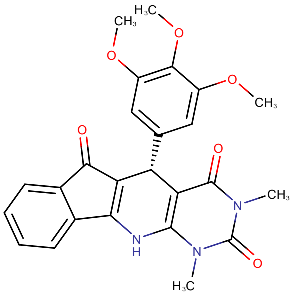	5.1	5.8	5.45
897	SN00102531	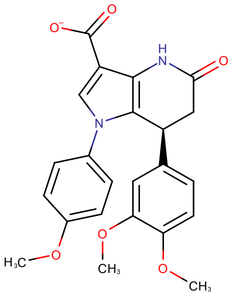	4.9	5.9	5.4

898	MolPort-008-348-587	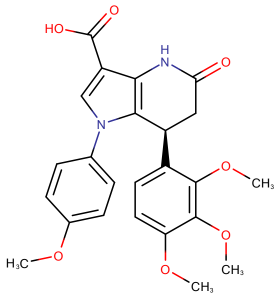	5.2	5.5	5.35
899	ZINC06786347	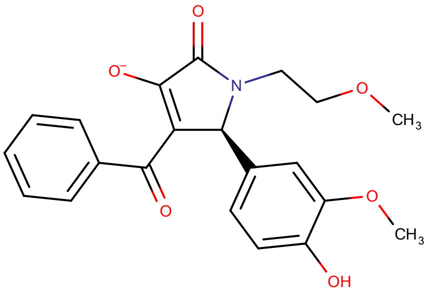	4.9	5.8	5.35
900	MolPort-035-701-663	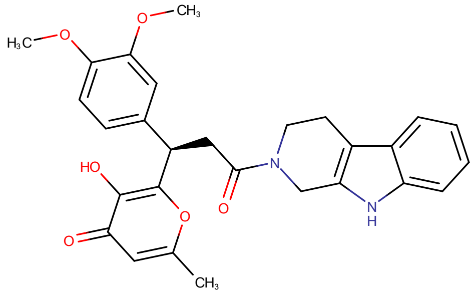	5.1	5.5	5.3
901	MolPort-007-846-702	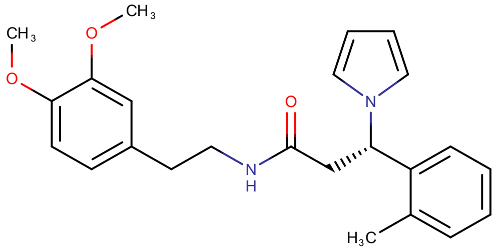	5.1	5.5	5.3
902	MolPort-002-524-175	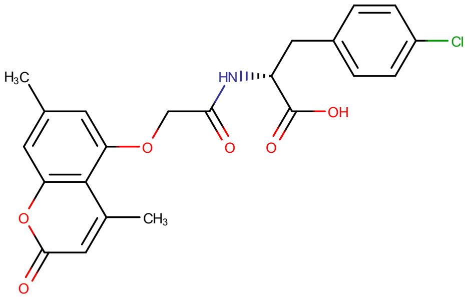	4.8	5.8	5.3

903	MolPort-002-528-359	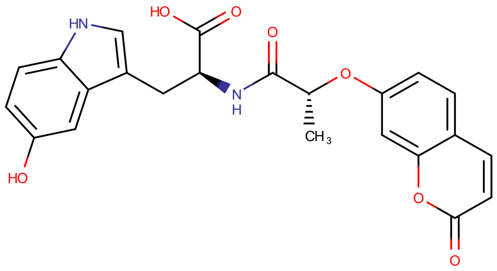	4.9	5.6	5.25
904	SN00102042	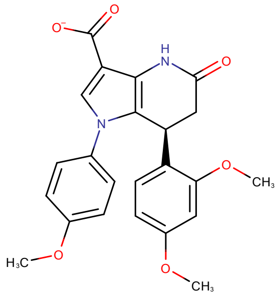	4.6	5.8	5.2
905	MolPort-002-571-308	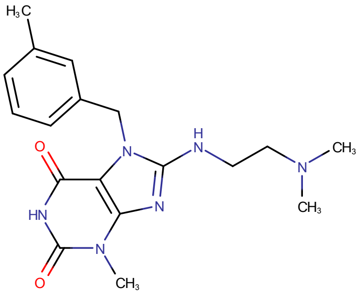	4.7	5.4	5.05
906	ZINC13409784	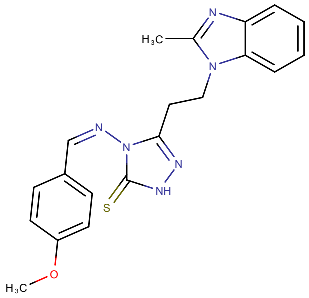	4.5	5.2	4.85
907	SN00102051	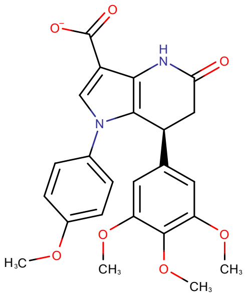	4.2	5.5	4.85

908	SN00102958	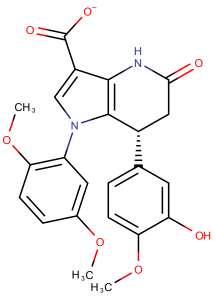	4.1	5.6	4.85
909	ZINC20467021	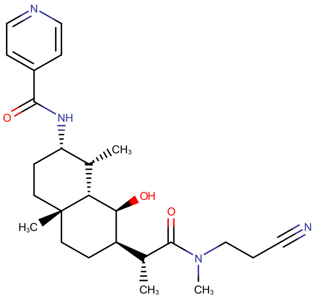	4.5	5	4.75
910	SN00086102	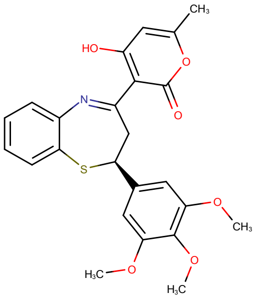	4	5.3	4.65

References

1 Pottie, E., Cannaert, A. & Stove, C. P. In vitro structure–activity relationship determination of 30 psychedelic new psychoactive substances by means of β-arrestin 2 recruitment to the serotonin 2A receptor. *Archives of Toxicology* **94**, 3449-3460, doi:10.1007/s00204-020-02836-w (2020).
